# Environmental Predictors of Ice Seal Presence in the Bering Sea

**DOI:** 10.1371/journal.pone.0106998

**Published:** 2014-09-17

**Authors:** Jennifer L. Miksis-Olds, Laura E. Madden

**Affiliations:** Applied Research Laboratory, The Pennsylvania State University, State College, Pennsylvania, United States of America; University of Auckland, New Zealand

## Abstract

Ice seals overwintering in the Bering Sea are challenged with foraging, finding mates, and maintaining breathing holes in a dark and ice covered environment. Due to the difficulty of studying these species in their natural environment, very little is known about how the seals navigate under ice. Here we identify specific environmental parameters, including components of the ambient background sound, that are predictive of ice seal presence in the Bering Sea. Multi-year mooring deployments provided synoptic time series of acoustic and oceanographic parameters from which environmental parameters predictive of species presence were identified through a series of mixed models. Ice cover and 10 kHz sound level were significant predictors of seal presence, with 40 kHz sound and prey presence (combined with ice cover) as potential predictors as well. Ice seal presence showed a strong positive correlation with ice cover and a negative association with 10 kHz environmental sound. On average, there was a 20–30 dB difference between sound levels during solid ice conditions compared to open water or melting conditions, providing a salient acoustic gradient between open water and solid ice conditions by which ice seals could orient. By constantly assessing the acoustic environment associated with the seasonal ice movement in the Bering Sea, it is possible that ice seals could utilize aspects of the soundscape to gauge their safe distance to open water or the ice edge by orienting in the direction of higher sound levels indicative of open water, especially in the frequency range above 1 kHz. In rapidly changing Arctic and sub-Arctic environments, the seasonal ice conditions and soundscapes are likely to change which may impact the ability of animals using ice presence and cues to successfully function during the winter breeding season.

## Introduction

Ribbon (*Histriophoca fasciata*) and bearded seal (*Erignathus barbatus*) vocalizations are salient vocalizations recorded seasonally in the Bering Sea from January-June when sea ice is present [1]–[2]. These calls are most likely produced by males as a display to attract females and establish territory during the mating season [3]–[8]. During the winter breeding season, the Bering Sea is cold, dark, and ice covered; consequently, these aquatically mating species must locate potential mates while also maintaining positions within the ice sheets where breathing holes can be maintained or where they have access to open water at the ice edge or within polynyas. How they navigate in the low visibility conditions of the dynamic ice sheets to locate potential mates and maintain access to open water for breathing and mating is not fully known. Artificially introduced acoustic cues were shown to be extremely important for a blindfolded spotted seal (*Phoca largha*) in navigating under ice to locate breathing holes [9]; therefore, it is not unreasonable to hypothesize that ribbon and bearded seals may also be using soundscape cues to orient under the ice. The goal of this work was to determine the strongest environmental predictors of ice seal vocal presence in the Bering Sea. Multiple environmental (ice and prey) and acoustic variables were considered in predictive models of ribbon and bearded seal presence during the winter in the Bering Sea, and it was the modeling results that provided insight as to the potential role the soundscape may play in under-ice navigation of these ice seals.

It is widely known that animals use sound to navigate through the environment. Bats and dolphins actively probe the environment with echolocation [10], and non-visual communication signals from conspecifics and heterospecifics guide animals in acquiring mates, foraging, and defense [11]. Over the past decade, there have been an increasing number of studies that explore how animals use information from the overall environmental “soundscape”, the combination of biologic (biophony), abiotic (i.e. wind, rain, and other geologic sounds referred to as geophony), and man-made (anthrophony) sounds, gained via passive listening for orientation and navigation [12]–[15]. The concept of using ambient or reflected sounds (as opposed to specific communication signals) to direct movement or identify appropriate habitats has recently been identified as a new field of study referred to as soundscape orientation, and the concept is also included within the broader field of soundscape ecology in the scientific literature [14], [16]–[17].

In the marine environment where visual signals do not propagate very far, animals rely on sound as their primary means of obtaining information over any significant distance. It has been speculated that large baleen whales use ambient acoustic cues or acoustic landmarks to guide their migration [18]–[22]. Laboratory and field studies have demonstrated that both invertebrates (oyster and crab) and fish use soundscape cues for orientation and localization of appropriate settlement habitat. The commonality between all the soundscape orientation experiments conducted in the marine environment, and select terrestrial habitats, is that the soundscapes identified as having an impact on animal behavior originated from areas of high species diversity in association with reefs or rainforests [12], [15], [23]–[27]. Habitats with greater biodiversity are associated with richer acoustic soundscapes compared to low diversity habitats, which in itself may be an important cue for animal orientation [16], [28]–[30]. This author is only aware of two studies that identify specific acoustic characteristics of the soundscape that are predictors of behavioral response. Stanley et al. (2011) [31] measured the sound intensity level required to elicit settlement and metamorphosis in several species of crab larvae, and Simpson et al. (2008) [32] discovered that coral reef fish responded more strongly to the higher frequency components (>570 Hz) of the reef soundscape.

It has been impossible to truly assess specific predictors, acoustic or otherwise, of behavioral response for ice seals living in conditions unhospitable to direct observation. However, advances in remote sensing technology and capabilities have provided the means to begin identifying potentially important parameters that are deserving of more in-depth study. This study used multiple, synoptically sampled time series from remotely deployed sensors to gain a better understanding of the environmental parameters most likely influencing ice seal behavior during the breeding season in the Bering Sea.

## Methods

### Moorings

**Figure 1:**
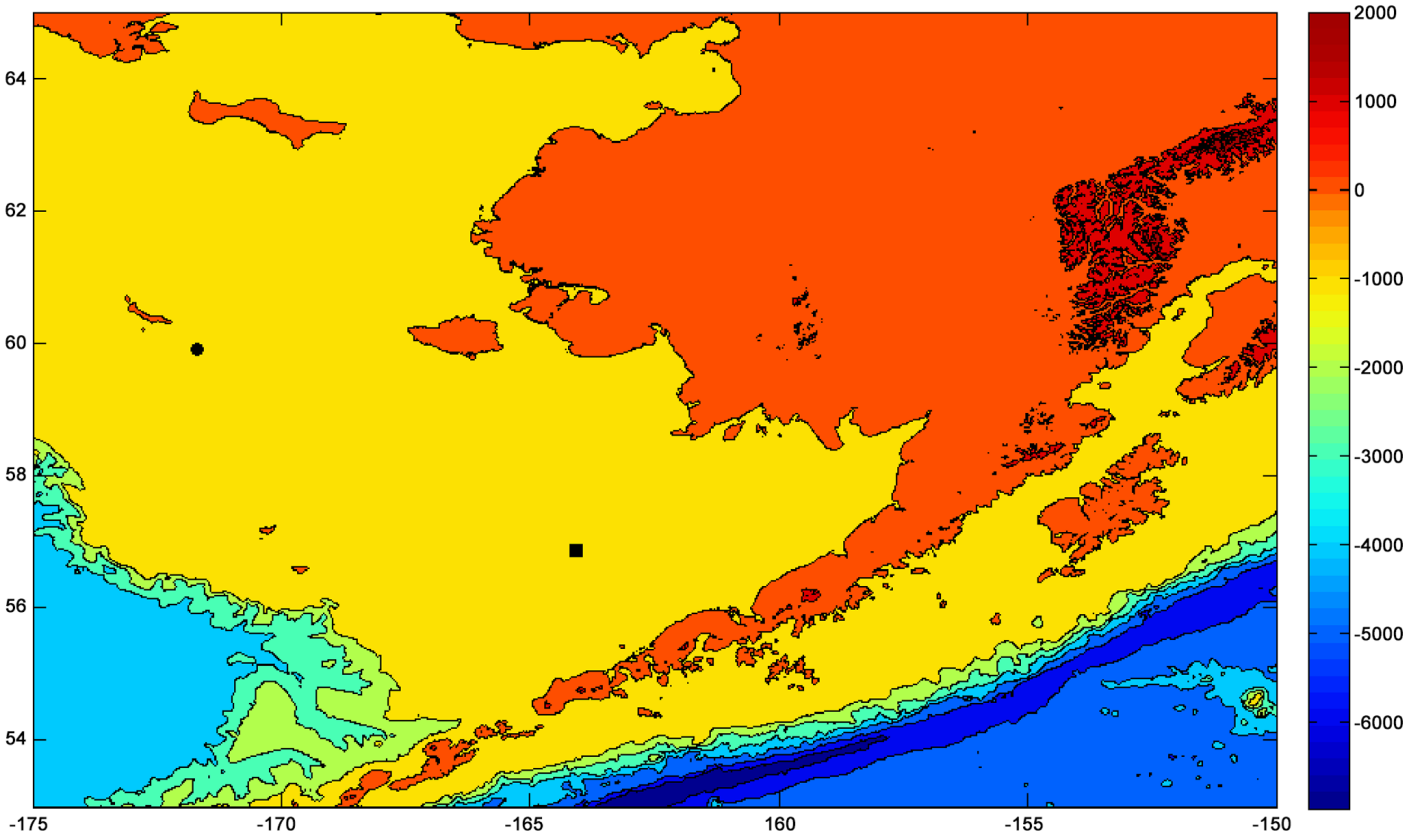
Mooring site locations in the Bering Sea.

Active and passive acoustic sensors were incorporated into subsurface acoustic moorings deployed at two locations on the 70-m isobath of the eastern Bering Sea shelf at sites M2 (56° 51.570′N, 164° 3.801′W) and M5 (59° 54.285′N, 171° 42.285′W) in 2009 and 2007, respectively. Moorings at these locations have been deployed and maintained as part of the NOAA Ecosystems and Fisheries-Oceanography Coordinated Investigations (EcoFOCI) Program (http://www.ecofoci.noaa.gov) since 1995 and 2004, respectively for the M2 and M5 moorings [33] (Figure 1). The acoustic sensors were integrated into the NOAA-deployed, observational moorings under a NOAA Request for Blanket Scientific Research Permit and did not require a specific permit for remote sensing. Moorings were deployed subsurface to prevent entanglement in seasonal sea ice and were serviced in Spring (April/May) and Fall (September/October) each year depending on weather conditions. The acoustic sensors were deployed on a separate, short mooring in conjunction with oceanographic moorings at each location. The oceanographic and acoustic moorings were separated by a distance of approximately 1 km to minimize noise produced by the oceanographic mooring hardware and sensors in the acoustic recordings. The data used in this study comes from acoustic data acquired 27 Sep 2009–19 May 2011 at location M2 and 26 Sep 2008–20 May 2011 at location M5.

The acoustic mooring consisted of a series of active and passive sensors including a 300 kHz RDI ADCP, three-frequency (125 kHz, 200 kHz, and 460 kHz) scientific echosounder system of Acoustic Water Column Profilers (AWCPs: ASL Environmental Sciences, Inc, Sidney, BC), Passive Aquatic Listener (PAL) recorder, and an AURAL (Autonomous Underwater Recorder for Acoustic Listening) (Multi-Électronique (MTE) Inc., Québec, Canada). The mooring was constructed in the following order from top (approximately 60–62 m) to bottom (approximately 70 m): 36″ floatation, 300 kHz RDI ADCP, 30″ floatation, AURAL, AWCP system, PAL, and acoustic release. The AWCP system was mounted in an upward-looking direction 15° off vertical to eliminate interference from flotation and instruments in the mooring line directly above the active acoustic system.

This study utilized data from the PAL and AWCP system. AWCPs record acoustic backscatter to monitor the presence and location of acoustic scatterers such as zooplankton and fish within the water column [34]–[35]. The transducers of the three different frequencies were positioned in the mooring cage so that the beam patterns were aligned to sample the same parcel of water nearly simultaneously. The echosounders sampled the water column for 5 min. each half hour. During each 5 min. sampling period, acoustic backscatter measurements were recorded every 2 s with 20 cm range bins from approximately 0.75 m above the transducer face to the water surface. Zooplankton net tows were conducted during mooring maintenance activities and on separate research cruises in the area using either a 25-cm diameter CalVET system (CalCOFI Vertical Egg Tow; [36]) having 0.15 mm mesh nets or double-oblique tows of paired bongo frames (60-cm frame with 0.333 mm mesh and 20-cm frame with 0.150 mm mesh) [2]. Data from the net tows provided information on dominant species, species composition, and numerical density to aid in defining size classes and interpretation of the acoustic data.

The PAL is an event detector, or adaptive sub-sampling acoustic recorder, with a temporal sampling strategy designed to allow the instrument to record data for up to one year [37]–[40]. The default sampling strategy was to record a 4.5 sec acoustic time series, or soundbite, at a sampling rate of 100 kHz every 5 minutes corresponding to a 1.5% duty cycle. When sampling in the default mode, onboard processing algorithms sub-sampled the 4.5 sec soundbite eight times and generated a power spectrum for each sub-sample. A preliminary detection algorithm identified signals of interest when a temporal feature of the sub-sampled power spectra in a soundbite exceeded one of three threshold criteria: 1) the matching of spectrum characteristics to known spectra, 2) exceeding a 12 dB threshold level between sequential samples indicating a transient source, or 3) the matching of predefined peaks (e.g. 300 Hz–3 kHz) indicating possible tonal or click vocalizations from marine mammals. If no signals of interest were detected, the spectra were averaged, and a single spectrum was saved to the hard disk. The soundbite time series was discarded in the default sampling mode. During periods of increased acoustic activity where signals of interest triggered a modified sampling protocol, the sampling interval was decreased to 2 minute intervals corresponding to a 4% duty cycle. In the modified sampling mode, individual spectra and the soundbites were saved to the hard disk. The PAL continued to operate in the modified sampling mode until no signals of interest were detected. The PAL then returned to the default sampling mode. Details on the adaptive sampling algorithms of the PAL are found in Miksis-Olds et al. (2010) [39].

Field data was collected under Observational Institutional Animal Care and Use Committee (IACUC) #36003 “Characterizing Biological Scatter and Its Implications for Marine Mammals in the Bering Sea” from The Pennsylvania State University. There was no direct interaction with any vertebrates in this study, as all data from marine mammals were obtained remotely through passive acoustic listening.

### Ice Data

Daily mean ice cover (or percent cover in this specific region) and ice thickness data were obtained from the images produced by the NOAA Ice Desk at the National Weather Service in Anchorage, Alaska. The images are posted on http://pafc.arh.noaa.gov/ice.php. Ice conditions surrounding the mooring locations were estimated within an approximate 20 km^2^ around the mooring.

### Data Processing

Ribbon and bearded seal presence was determined from the PAL soundbites with the understanding that detection of vocalizations indicates seal presence, and the lack of acoustic detection does not imply animal absence. Soundbites were reviewed by a human classifier and verified by a second independent human classifier blind to the results of the first reviewer. Sound sources present in the soundbites were identified from spectrograms (1024 point FFT, Hamming window, 87.5% overlap) made from the original 100 kHz recordings downsampled to 48 kHz using Adobe Audition 3.0 (Adobe Systems Incorporated). These settings provided a bandwidth of 61 Hz, with a frequency resolution of 47 Hz, and a time resolution of 2.7 ms. Marine mammal vocalizations were classified aurally and visually from the spectrograms by species (bowhead (*Balaena mysticetus*), gray whale (*Eschrichtius robustus*), killer whale (*Orcinus orca*), beluga whale (*Delphinapterus leucas*), walrus (*Odobenus rosmarus*), ribbon, and bearded seals). Ribbon seal grunts, roars, and downsweeps were used to indicate presence [1], [41]–[42]. Bearded seal vocal presence was determined by the identification of trills, the most salient of the bearded seal vocalizations [6]–[7]. The adaptive sampling protocol and low sampling duty cycle of the PAL prevented calculations of the daily detection rate or overall number of seal vocalizations per day.

Analysis of PAL spectra included examination of spectral shape and levels. Temporal clusters of similarly distinctive sound spectra lasting tens of minutes to hours were manually identified and classified. Sound levels were computed from the time series of spectra. Each spectrum was computed from 1024 point samples of the 4.5 s time series. This resulted in a 513 point power spectral density with each of the bins covering 97 Hz of the 50 kHz usable bandwidth. The spectra were then reduced from 513 points to 64 points by averaging spectra levels over two bins below 3 kHz and over ten bins from 3 to 50 kHz. The resulting power spectral density, relative to µ1Pa^2^/Hz, represents energy from the complete 50 kHz bandwidth with variable frequency resolution. To compute the sound level from these spectra, the values were converted to linear power spectral density and multiplied by the frequency resolution of the bins and then summed. The unit of the full bandwidth average is a sound pressure level, re 1 µPa. Processing of power spectral density was conducted for five frequencies over seven octaves (500 Hz, 2 kHz, 10 kHz, 20 kHz, 40 kHz) with units of dB re 1 µPa^2^/Hz.

To assess prey parameters related to zooplankton/fish abundance and community composition, the AWCP data were processed in 5 m vertical depth bins. Daily mean volume backscatter coefficient (mean S_v_ in units m^2^/m^3^) was calculated from 24 hour integrations over each 5 m depth layer using EchoView software (Myriax, Tasmania). Targets within each depth and time bin were classified as to the likely source of the scattering based on differences in scattering amplitude between the three frequencies. Analyses using this dB-difference approach [2], [43]–[45] are typically groundtruthed with information from net tows or video observations. However, given the low level of direct sampling of the water column in this study, a different approach was used and was consistent with Miksis-Olds et al. (2013) [2] summarized here. If scattering assemblages were monospecific, then the dB-difference for a single scatterer type and an aggregation of scatterers of this type would be identical, although the volume backscattering at each frequency would be different. Theoretical scattering curves for four different types of individual scatterers were generated and dB-differences between the three AWCP frequencies were calculated. Scattering amplitudes (and the subsequent dB differences) were generated using a Stochastic Distorted Wave Born Approximation model [46] for the following scatterers: 1) small scatterers such as neritic copepods (lengths of 1–5 mm) (*Pseudocalanus* spp., *Acartia longiremis, Oithona* spp. and *Calanus*), 2) medium scatterers (lengths of 5–15 mm) which includes juvenile krill, chaetognaths, and amphipods, 3) large scatterers such as adult euphausiids (lengths of 15–30 mm), 4) resonant scatterers which represents an organism with a gas-inclusion such as a swim-bladdered fish or siphonophore, and 5) unknown. The acoustic system was not able to detect the weak scattering strengths of scatterers less than approximately 5 mm in length unless they were present in extremely dense aggregations. Aggregations were classified as belonging to one of the five categories (small, medium, or large scatterer; resonant; or unknown) by determining the shortest geometric distance between the three dB differences calculated for the aggregation and that of the theoretical scatterers. If the closest geometric distance was more than 12 dB (an arbitrarily chosen value), then the aggregation was classified as unknown.

#### Modeling

Daily presence-absence data for ribbon and bearded seals identified in the passive acoustic recordings was the response variable in the generalized linear and generalized additive models (GLM and GAM) designed to identify predictor variables of ice seal presence (Table S1). There is a high degree of temporal overlap between ribbon and bearded seal detections in the Bering Sea [1]–[2], so daily presence-absence data for the two species was combined into a single ice seal response variable to increase statistical power. Initial models included the following predictor variables: ice thickness, % ice cover, 200 kHz Sv, % prey composition (small, medium, large, and resonant scatterers), and mean daily sound level (500 Hz, 2 kHz, 10 kHz, 20 kHz, 40 kHz) (Table S1 and Table S2). Data was first explored to identify potential outliers and evaluate distribution and collinearity among predictor variables and also with marine mammal presence using functions of the AED package in R [47]–[48]. Explanatory variables were centered to allow better model convergence and interpretation, with the exception of ice cover and thickness. Ice cover and ice thickness showed a zero-inflated distribution and transformations failed to sufficiently address the skewed distributions. Zero values are meaningful for these measurements and thus these variables were not truncated or transformed. High collinearity was found between environmental sound level variables with the correlation highest between close frequencies. Ice cover and ice thickness were also highly collinear, although one or both of these variables were removed from the models during the selection process so this collinearity did not pose a problem. Final models including multiple noise variables were checked for collinearity using the *corvif* function from the R package *AED* [47]–[48]. All variables included in final models had VIFs well below 10 (the maximum VIF was 2.32), indicating sufficiently low collinearity [49].

Generalized linear models (GLMs) and generalized additive models (GAMs) allow model fitting to describe relationships between variables without constraints of linear regression models [50]. Generalized additive mixed models (GAMMs) and generalized linear mixed models (GLMMs) extend GAMs and GLMs to include random effects and correlation structures to deal with violations of independence that are often present in observational and time series data and are becoming popular in the analysis of ecological data (for example: Friedlaender et al., 2006 [51]; Wagner & Sweka, 2011 [52]). GLMs and GLMMs with a binomial distribution and logit link function were fit using a backward stepwise approach. Variables were selected for removal using the *drop1* command from the basic *stats* package in R (version 2.14.1; [53]) to apply an analysis of deviance test following a Chi-square distribution. Variables were removed based on a significance criteria of p<0.01 until all variables in the model were considered significant. Significance tests and p-values for analysis of deviance are approximate, thus a selection criteria below the standard 95% significance level was used to avoid inclusion of unnecessary terms. GLMMs were fit using the *glmmPQL* function from the *MASS* package in R [54]. This approach allowed the inclusion of a random effect for site to allow inference beyond the two stations sampled and a temporal correlation structure to address the lack of independence due to repeated sampling at each site. Convergence problems were frequently encountered when a temporal correlation structure for date grouped by site was included. Auto-correlation in the model residuals was examined to determine whether the temporal correlation structure was needed, as including a random effect for site imposes an implicit compound symmetry correlation structure that assumes a constant correlation within data points from the same site. GAMs and GAMMs with a binomial distribution and logit link function were fit using the same procedure described above with the *gamm* function from the *mgcv* package in R [55]–[56] to explore potential non-linear relationships.

GLMM and GAMM techniques are on the “frontier of statistical research” and as such model selection and validation for generalized models on absence-presence response data is difficult [47]. Standardized residuals were extracted and plotted against predictor variables and fitted values to look for patterns. Greater variation in residuals at zero ice coverage was discovered, likely due to the large number of zero values for ice cover. A new data set, zero-truncated for ice cover, was then used to fit the final models to explore the potential for zero values to interfere with model function and selection.

## Results

**Figure 2:**
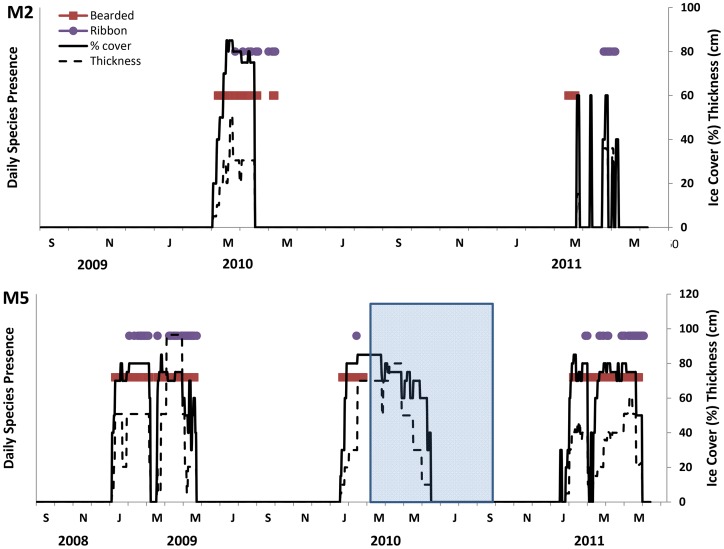
Time series of daily ice and ice seal presence over the M2 and M5 moorings where data exist from 2008–2011. Acoustic presence of species does not correspond to a numerical value on the y axis. The species-specific symbols reflect daily acoustic presence and are separated spatially for easy visualization. The blue box indicates a period of time where no acoustic data were available from the PAL.

Seasonal ice was present at both mooring locations in the Bering Sea during each winter of the study (Figure 2). The ice cover over M5 on the central shelf was thicker and present longer compared to M2 on the southeastern shelf. Bearded seals were detected on 340 days, and ribbon seals were detected on 161 days over the study period from Sep 2008-May 2011. Seals were detected on fewer days at the southern mooring (M2) most likely due to the less persistent and shorter duration of ice cover compared to M5, but the proportion of daily detections for each species was similar at both mooring locations (39 days (63%) bearded and 23 days (37%) ribbon detected at M2; 301 days (68%) bearded and 138 days (32%) ribbon at M5) (Figure 2).

**Figure 3:**
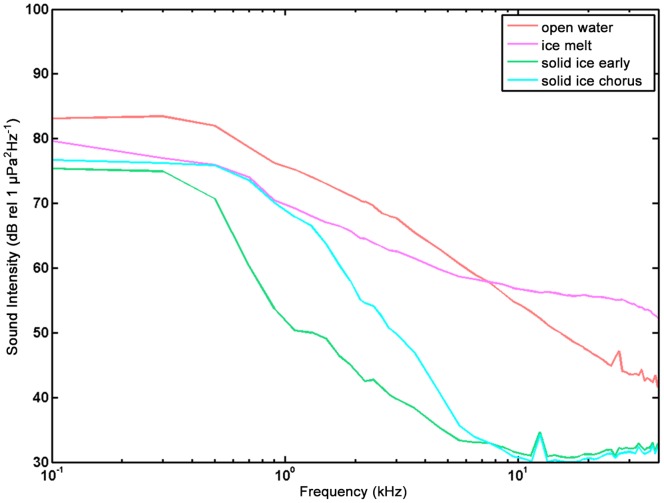
Representative spectra from the Bering Sea under different surface conditions. The Solid Ice Early spectrum represents the acoustic environment prior to the seasonal arrival of chorusing ice seals. The Solid Ice Chorus spectrum captures the acoustic environment when ice seals were observed to be chorusing in the acoustic record.

**Figure 4:**
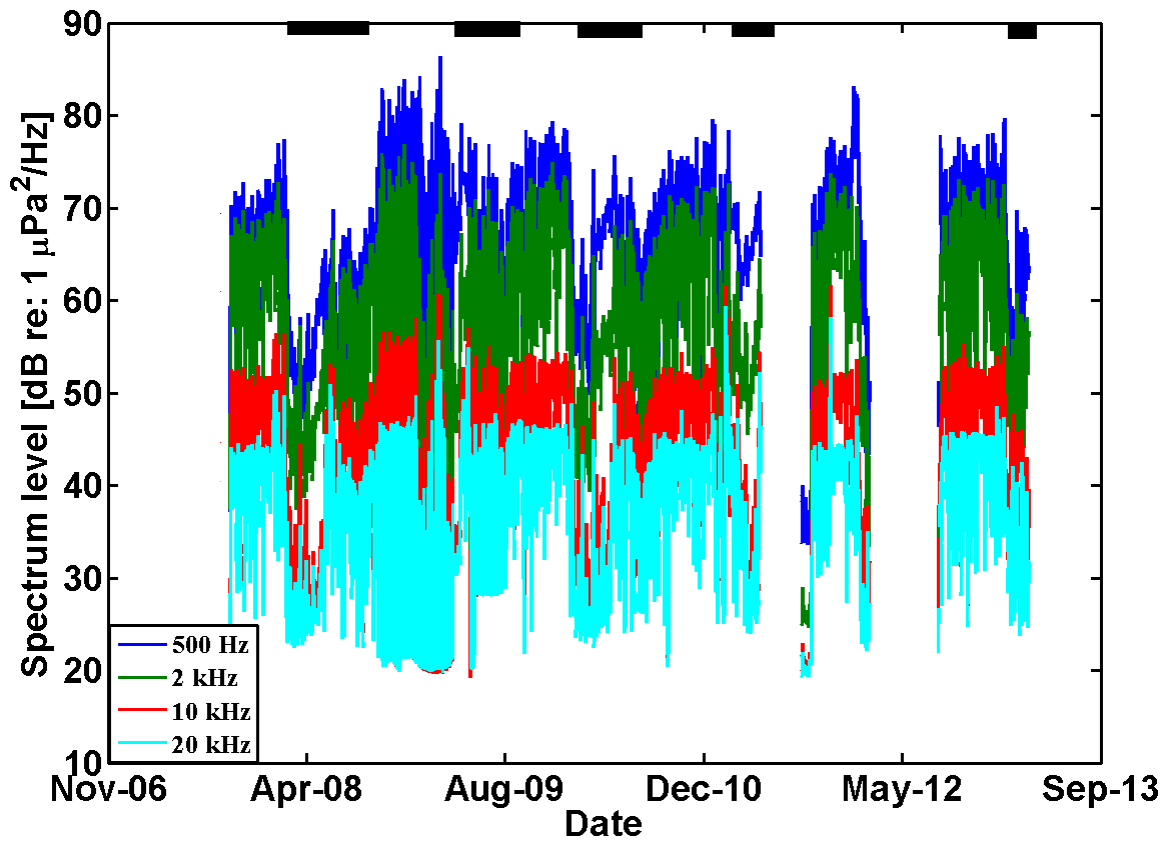
Sound pressure levels at four frequencies from the M5 location in the Bering Sea over a 4 year time period. Gaps in the data are periods when no data was available from the PAL. The black bars across the top of the figure indicate presence of regional ice cover.

The grouping of PAL spectra identified four general sea surface conditions (open water, freeze up, solid ice, seasonal melting) (Figure 3). Validation of sea ice conditions from the passive acoustic data was inferred from the satellite ice thickness and mean ice cover calculations, seasonality, and recorded soundbites of physical processes. Overall sound levels during open water conditions were generally greater than when ice was present for frequencies of 1–10 kHz, which was consistent with previous studies (Figures 3 and 4) [2]. Above 10 kHz, melting conditions produced the greatest sound intensity. For frequencies less than 1 kHz, open water and initial freeze-up conditions had the greatest sound intensity. When solid ice was present above the moorings, sound levels were observed to be the lowest across the full frequency spectrum, with extremely low level intensity (<40 dB re 1 µPa^2^/Hz) above 5 kHz. The internal noise floor of the recorder was likely a limiting factor for sound levels below approximately 32 dB.

The initial models to determine predictors of ice seal presence included ice thickness, % ice cover, 200 kHz Sv, four size categories of % prey composition (small, medium, large, and resonant scatterers), and five sound levels (500 Hz, 2 kHz, 10 kHz, 20 kHz, 40 kHz). The final GLM and GLMM model both included ice cover, 10 kHz sound, 40 kHz sound, and an interaction between ice cover and large crustaceans as significant predictors of ice seal presence (Table 1). Ice seal presence showed a strong positive correlation with ice cover and a negative association with 10 kHz environmental sound levels (Table 1). Prey alone was not a good predictor of seal presence and an interaction between ice cover and large crustaceans indicates a negative relationship with seal presence, likely due to the somewhat non-linear relationship between ice seals and ice cover at M5 (discussed below with GAMM models).

**Table 1:** GLM and GLMM final model results.

Variable	Parameter Estimate	Std. Error	DF	p-value
**GLM**				
Intercept	–4.353	0.380	902	<0.001
Ice cover	8.253	0.865	902	<0.001
*c* 10 kHz sound	–0.146	0.039	902	<0.001
*c* 40 kHz sound	0.130	0.045	902	0.004
*c* Large crustacean	0.036	0.017	902	0.031
Ice cover: *c* Large crustacean	–0.104	0.034	902	0.002
**GLMM**				
* Random effect: site*				
Intercept	–4.353	0.332	904	<0.001
Ice cover	8.254	0.756	904	<0.001
*c* 10 kHz noise	–0.146	0.034	904	<0.001
*c* 40 kHz noise	0.130	0.039	904	0.001
*c* Large crustacean	0.036	0.015	904	0.014
Ice cover: *c* Large crustacean	–0.104	0.030	904	<0.001
**GLMM**				
* Random effect: site*				
* Correlation: CAR1*				
Intercept	–3.700	0.314	1315	<0.001
Ice cover	5.947	0.482	1315	<0.001
*c* 10 kHz noise	–0.071	0.016	1315	<0.001

The letter *c* denotes centered variables. Ice cover is given as a fraction of cover from 0 to 1, large crustacean represents a percent composition, and both 10 kHz and 40 kHz are given in dB re 1 µPa^2^/Hz. The random intercept for site in the GLMM has a standard error of 0.0001 and residual standard error of 0.871. The explanatory variable large crustacean does not meet significance selection criteria (p<0.01), however is included due to the significance of its interaction term with ice cover.

**Figure 5:**
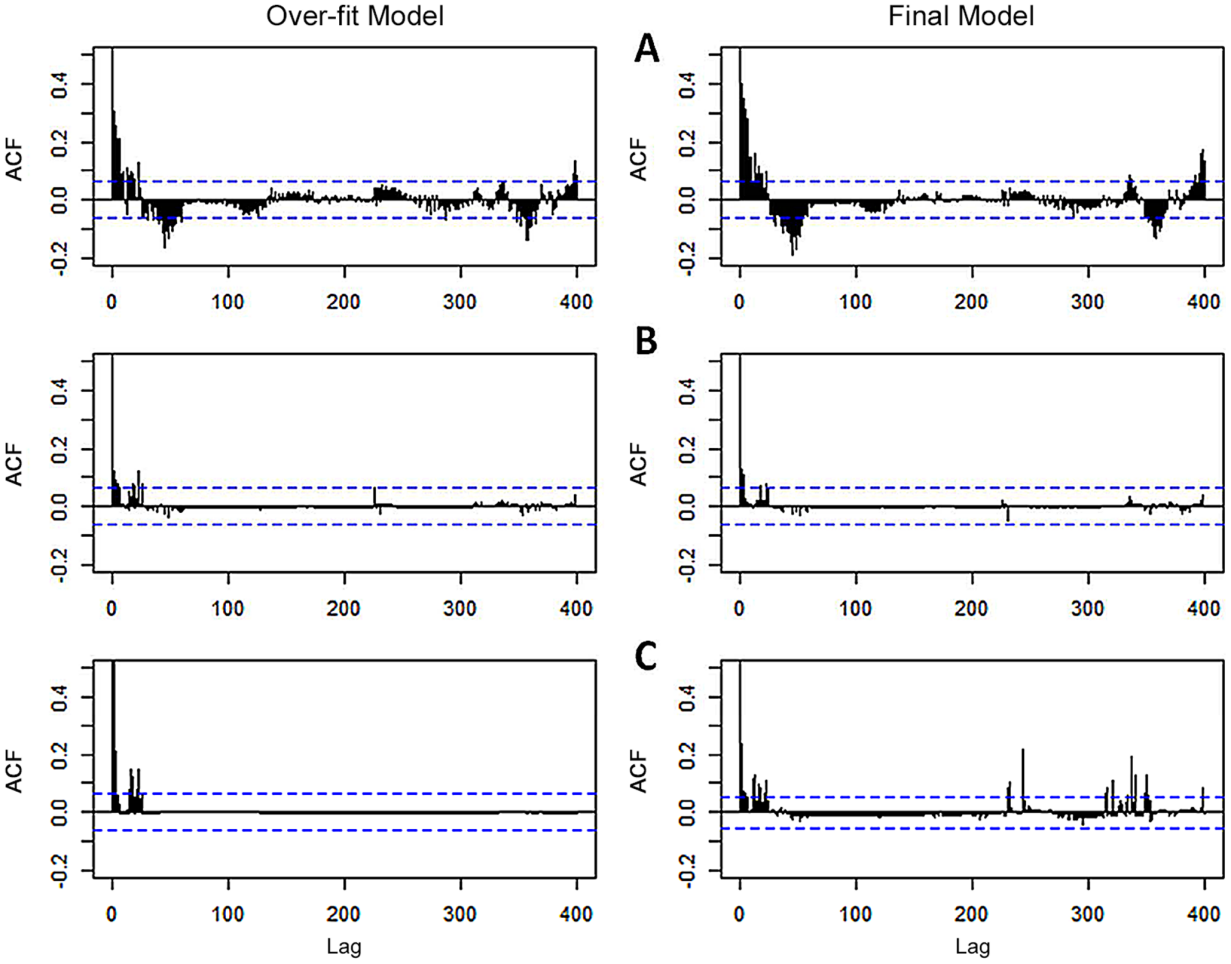
Auto-correlation function (ACF) of model residuals. Plots show auto-correlation of model residuals to 400 lags (400 days) for A) GLM with no random effects or temporal correlation structure, B) GLMM with random site effect and no temporal correlation structure and C) GLMM with random site effect and continuous AR-1 correlation structure. Over-fit models include all explanatory variables and interactions under consideration. Final models include only significant predictor variables after model selection.

Parameters estimated by both the GLM and GLMM are nearly identical (Table 1), suggesting little difference between M2 and M5. However, the inclusion of a random site effect in the GLMM was highly effective in addressing residual autocorrelation (Figure 5B). The inclusion of a temporal correlation structure in the GLMM reduced numerical stability (increased non-convergence problems) and captured less of the residual auto-correlation in the final model (Figure 5C). The GLMM with a random site effect and no temporal correlation structure (Figure 5B) was selected as the optimal model.

**Figure 6:**
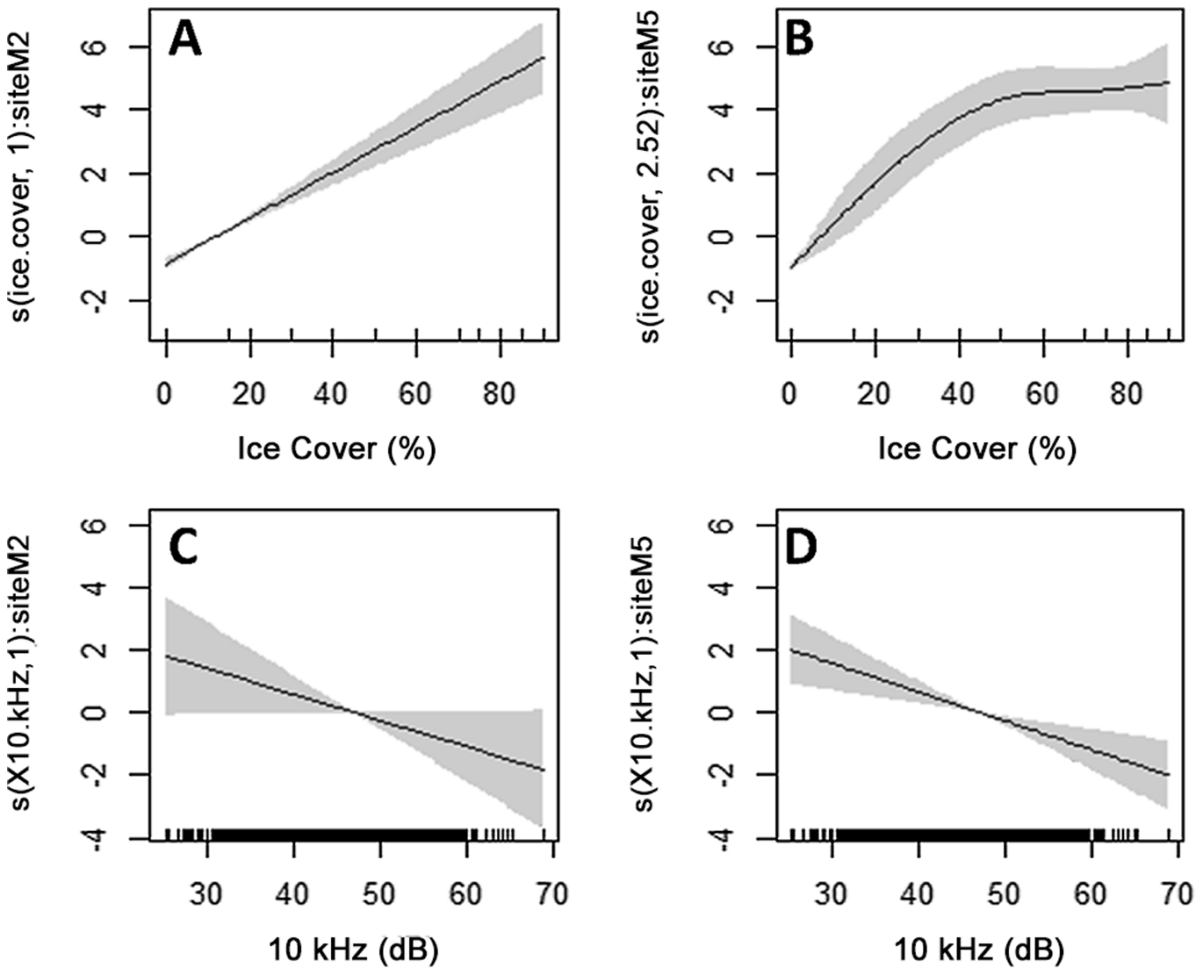
GAMM comparison of smooth functions by site for (A–B) percent ice cover and (C–D) 10 kHz sound. Shaded areas denote 95% confidence intervals. The smooth for M2 on 10 kHz sound was not significant (C). Increase in ice seal presence slows beyond 50% ice cover at M5 (B), although the relationship is still generally linear.

GAMM models showed primarily linear relationships between seal presence and ice cover and 10 kHz sound, with the exception of ice cover at M5 (Figure 6B, Table 2). The plateau in the seal-ice cover smoother seen at M5 may explain the negative slope of the ice cover: large crustacean interaction terms in Table 1. The final GAMM model included smooth terms for ice cover and 10 kHz sound with a random smoother for ice cover (Table 2). Including random smoothers for ice cover and 10 kHz sound resulted in neither 10 kHz smoother being significant. All GAMM models with 40 kHz sound failed to converge.

**Table 2:** Final GAMM model parameters including a random smoother by site for ice cover.

Variable	edf	Std. Error	p-value
**GAMM**			
Intercept	–3.394	0.225	<0.001
s(ice cover): M2	1.000	–	0.003
s(ice cover): M5	2.522	–	<0.001
s(10 kHz)	1.000	–	<0.001

The estimated degrees of freedom (edf) indicate the “curviness” of the smooth terms with 1.00 representing a straight line. A linear relationship is indicated for 10 kHz sound (both sites) and ice cover at M2.

Figure 6B suggests a non-linear effect of ice cover on seal presence as the increasing trend in the smoother levels near 50 percent cover. However, 2.52 degrees of freedom alone is not strong evidence against a GLMM [47]. This relationship must also be regarded with caution as non-convergence issues disallowed inclusion of all predictor variables of interest in the fitting of this model. As a result, the GLMM with a random site effect was selected as the optimal model for this data. Ice cover and 10 kHz sound level appear to be significant predictors of seal presence, with 40 kHz sound and prey presence (combined with ice cover) as potential predictors as well.

## Discussion

Sea ice and 10 kHz sound levels were the strongest predictors of ice seal vocal presence during the winter breeding season in the Bering Sea. The results indicate that as 10 kHz (and to a lesser extend 40 kHz) sound levels increased, the detection of ice seal vocalizations decreased. Neither ribbon seals nor bearded seals have a significant amount of energy in their vocalizations above 10 kHz [1], [6], but if the underwater hearing capabilities of ribbon and bearded seals are comparable to other phylogenetically related, ice-dependent species (e.g. spotted seal (*Phoca largha*), ring seal (*Pusa hispida*), harbor seal (*Phoca vitulina*) [57]) then they are capable of hearing sound above 70 kHz [58]–[61]. The 10 kHz frequency falls directly within the frequency range of best hearing for related phocid species [58]–[61], so it is appropriate to conclude that ribbon and bearded seals can both detect and respond to sound signals in the 10–40 kHz range. Although it is known that ice seals hear and vocalize underwater, there is little direct evidence about how they use or rely on sound to direct their movements and behavior.

The modeling results directed a more detailed examination of the acoustic environment that ice seals encounter on an annual basis. Open water conditions are the loudest up to approximately 8 kHz (Figure 3). Above 8 kHz, conditions associated with the process of ice melting were loudest. Conversely, acoustic conditions associated with solid ice cover were the quietest over the entire spectrum from <5 Hz to 50 kHz. On average, there was a 20–30 dB difference between sound levels during solid ice conditions compared to open water or melting conditions. This difference provides a salient acoustic gradient between open water and solid ice conditions by which ice seals could orient to maintain their horizontal position within the ice sheet or proximity to the ice edge so that access to open water for breathing is preserved. By constantly assessing the acoustic environment to navigate along with the seasonal ice movement in the Bering Sea, it is possible that ice seals can gauge their safe distance to open water or the ice edge through the soundscape in dark, ice covered surroundings by orienting in the direction of higher sound levels, especially in the frequency range above 1 kHz.

This observational study was not able to establish a cause-effect relationship or identify a specific threshold or optimal sound level range that ribbon and bearded seals may employ to navigate under ice. Long-term tagging studies with acoustic dosimeters and GPS location capabilities will be needed to confirm this theory and provide direct evidence of the mechanisms of under-ice navigation in ice seals. It will also be useful to investigate this relationship across locations and regions in both the Arctic and Antarctic to assess whether this concept can be generalized to all ice-dependent species required to navigate under ice. This work presents a particularly timely observation, as the sea ice and acoustic conditions of the oceans, the strongest predictors of ice seal vocal presence during the winter breeding season, are changing due to climate change and industrialization related to shipping and energy exploration/production [62]–[63]. In order to fully access the risk this poses to ice seals, it is critical to gain a better understanding of how the seals use and rely on ice presence and its associated sound to survive in their extreme environments.

## Supporting Information

Table S1 **Response and predictor variables used in the GLM and GAM modeling at the M2 (A) and M5 (B) locations.** Bearded and ribbon seal acoustic presence is a binary response: 0 for absent, 1 for present. The 200 kHz Sv are daily mean values, and the scatterer percent composition is reflective of the daily mean values within each size category. Ice cover % and ice thickness are daily assessment values.
(DOCX)

Table S2 **Daily mean sound levels at M2 (A) and M5 (B) used in the GLM and GAM modeling. Sound level units are dB re 1 μPa^2^/Hz.**
(DOCX)
