# Supplementary material for: Environmental Predictors of Ice Seal Presence in the Bering Sea
Source: PLoS One. 2014 Sep 17;9(9):e106998. doi: 10.1371/journal.pone.0106998 (PMC4167550; doi:10.1371/journal.pone.0106998)
Supplement: Table S1 — Response and predictor variables used in the GLM and GAM modeling at the M2 (A) and M5 (B) locations. Bearded and ribbon seal acoustic presence is a binary response: 0 for absent, 1 for present. The 200 kHz Sv are daily mean values, and the scatterer percent composition is reflective of the daily mean values within each size category. Ice cover % and ice thickness are daily assessment values. (DOCX) [file pone.0106998.s001.docx]

**Table S1 Response and predictor variables used in the GLM and GAM modeling at the M2 (A) and M(5) locations.** Bearded and ribbon seal acoustic presence is a binary response: 0 for absent, 1 for present. The 200 kHz Sv are daily mean values, and the scatterer percent composition is reflective of the daily mean values within each size category.

A)

| **Date** | **Bearded seal presence** | **Ribbon seal presence** | **Ice cover (%)** | **Ice thickness (cm)** | **200 kHz Sv (dB)** | **Small scatter % comp** | **Medium scatter % comp** | **Large scatter % comp** | **Weak resonator % comp** | **Strong resonator % comp** | **Unclass % comp** |
| --- | --- | --- | --- | --- | --- | --- | --- | --- | --- | --- | --- |
| 9/27/2009 | 0 | 0 | 0 | 0 | -60.4 | 0 | 25 | 75 | 0 | 0 | 0 |
| 9/28/2009 | 0 | 0 | 0 | 0 | -57.3 | 0 | 21 | 79 | 0 | 0 | 0 |
| 9/29/2009 | 0 | 0 | 0 | 0 | -68.1 | 0 | 9 | 91 | 0 | 0 | 0 |
| 9/30/2009 | 0 | 0 | 0 | 0 | -68.1 | 0 | 6 | 94 | 0 | 0 | 0 |
| 10/1/2009 | 0 | 0 | 0 | 0 | -64.7 | 0 | 18 | 82 | 0 | 0 | 0 |
| 10/2/2009 | 0 | 0 | 0 | 0 | -62.8 | 0 | 13 | 87 | 0 | 0 | 0 |
| 10/3/2009 | 0 | 0 | 0 | 0 | -66.3 | 0 | 15 | 85 | 0 | 0 | 0 |
| 10/4/2009 | 0 | 0 | 0 | 0 | -69.3 | 0 | 12 | 88 | 0 | 0 | 0 |
| 10/5/2009 | 0 | 0 | 0 | 0 | -66.6 | 0 | 15 | 85 | 0 | 0 | 0 |
| 10/6/2009 | 0 | 0 | 0 | 0 | -62.9 | 0 | 18 | 82 | 0 | 0 | 0 |
| 10/7/2009 | 0 | 0 | 0 | 0 | -65.4 | 0 | 14 | 86 | 0 | 0 | 0 |
| 10/8/2009 | 0 | 0 | 0 | 0 | -69.3 | 0 | 13 | 87 | 0 | 0 | 0 |
| 10/9/2009 | 0 | 0 | 0 | 0 | -66.3 | 0 | 19 | 81 | 0 | 0 | 0 |
| 10/10/2009 | 0 | 0 | 0 | 0 | -65.5 | 0 | 12 | 88 | 0 | 0 | 0 |
| 10/11/2009 | 0 | 0 | 0 | 0 | -68.0 | 0 | 13 | 87 | 0 | 0 | 0 |
| 10/12/2009 | 0 | 0 | 0 | 0 | -68.5 | 0 | 15 | 85 | 0 | 0 | 0 |
| 10/13/2009 | 0 | 0 | 0 | 0 | -70.0 | 0 | 13 | 87 | 0 | 0 | 0 |
| 10/14/2009 | 0 | 0 | 0 | 0 | -65.9 | 0 | 15 | 85 | 0 | 0 | 0 |
| 10/15/2009 | 0 | 0 | 0 | 0 | -58.7 | 0 | 28 | 72 | 0 | 0 | 0 |
| 10/16/2009 | 0 | 0 | 0 | 0 | -59.2 | 0 | 27 | 73 | 0 | 0 | 0 |
| 10/17/2009 | 0 | 0 | 0 | 0 | -61.9 | 0 | 26 | 74 | 0 | 0 | 0 |
| 10/18/2009 | 0 | 0 | 0 | 0 | -63.0 | 0 | 18 | 82 | 0 | 0 | 0 |
| 10/19/2009 | 0 | 0 | 0 | 0 | -64.6 | 0 | 23 | 77 | 0 | 0 | 0 |
| 10/20/2009 | 0 | 0 | 0 | 0 | -62.0 | 0 | 25 | 75 | 0 | 0 | 0 |
| 10/21/2009 | 0 | 0 | 0 | 0 | -62.1 | 0 | 20 | 80 | 0 | 0 | 0 |
| 10/22/2009 | 0 | 0 | 0 | 0 | -65.6 | 0 | 13 | 87 | 0 | 0 | 0 |
| 10/23/2009 | 0 | 0 | 0 | 0 | -64.0 | 0 | 11 | 89 | 0 | 0 | 0 |
| 10/24/2009 | 0 | 0 | 0 | 0 | -66.9 | 0 | 8 | 92 | 0 | 0 | 0 |
| 10/25/2009 | 0 | 0 | 0 | 0 | -65.7 | 0 | 11 | 89 | 0 | 0 | 0 |
| 10/26/2009 | 0 | 0 | 0 | 0 | -63.7 | 0 | 17 | 83 | 0 | 0 | 0 |
| 10/27/2009 | 0 | 0 | 0 | 0 | -63.0 | 0 | 21 | 79 | 0 | 0 | 0 |
| 10/28/2009 | 0 | 0 | 0 | 0 | -60.9 | 0 | 27 | 73 | 0 | 0 | 0 |
| 10/29/2009 | 0 | 0 | 0 | 0 | -61.5 | 0 | 14 | 86 | 0 | 0 | 1 |
| 10/30/2009 | 0 | 0 | 0 | 0 | -59.1 | 0 | 14 | 86 | 0 | 0 | 0 |
| 10/31/2009 | 0 | 0 | 0 | 0 | -56.6 | 0 | 24 | 75 | 0 | 0 | 1 |
| 11/1/2009 | 0 | 0 | 0 | 0 | -65.0 | 0 | 12 | 88 | 0 | 0 | 0 |
| 11/2/2009 | 0 | 0 | 0 | 0 | -64.8 | 0 | 14 | 85 | 0 | 0 | 0 |
| 11/3/2009 | 0 | 0 | 0 | 0 | -61.5 | 0 | 17 | 83 | 0 | 0 | 1 |
| 11/4/2009 | 0 | 0 | 0 | 0 | -60.7 | 0 | 19 | 80 | 0 | 0 | 1 |
| 11/5/2009 | 0 | 0 | 0 | 0 | -58.2 | 0 | 18 | 79 | 0 | 0 | 4 |
| 11/6/2009 | 0 | 0 | 0 | 0 | -60.5 | 0 | 11 | 84 | 0 | 0 | 5 |
| 11/7/2009 | 0 | 0 | 0 | 0 | -54.1 | 0 | 17 | 83 | 0 | 0 | 0 |
| 11/8/2009 | 0 | 0 | 0 | 0 | -56.3 | 0 | 18 | 82 | 0 | 0 | 0 |
| 11/9/2009 | 0 | 0 | 0 | 0 | -61.4 | 0 | 20 | 80 | 0 | 0 | 0 |
| 11/10/2009 | 0 | 0 | 0 | 0 | -60.9 | 0 | 20 | 80 | 0 | 0 | 0 |
| 11/11/2009 | 0 | 0 | 0 | 0 | -59.0 | 0 | 18 | 80 | 0 | 0 | 2 |
| 11/12/2009 | 0 | 0 | 0 | 0 | -67.8 | 0 | 11 | 82 | 0 | 0 | 7 |
| 11/13/2009 | 0 | 0 | 0 | 0 | -64.6 | 0 | 14 | 83 | 0 | 0 | 3 |
| 11/14/2009 | 0 | 0 | 0 | 0 | -61.4 | 0 | 15 | 78 | 0 | 0 | 7 |
| 11/15/2009 | 0 | 0 | 0 | 0 | -58.1 | 0 | 19 | 79 | 0 | 0 | 3 |
| 11/16/2009 | 0 | 0 | 0 | 0 | -58.2 | 0 | 21 | 79 | 0 | 0 | 0 |
| 11/17/2009 | 0 | 0 | 0 | 0 | -60.2 | 0 | 21 | 79 | 0 | 0 | 0 |
| 11/18/2009 | 0 | 0 | 0 | 0 | -55.2 | 0 | 16 | 84 | 0 | 0 | 0 |
| 11/19/2009 | 0 | 0 | 0 | 0 | -57.8 | 0 | 18 | 82 | 0 | 0 | 0 |
| 11/20/2009 | 0 | 0 | 0 | 0 | -60.5 | 0 | 20 | 80 | 0 | 0 | 0 |
| 11/21/2009 | 0 | 0 | 0 | 0 | -65.8 | 0 | 15 | 79 | 0 | 0 | 5 |
| 11/22/2009 | 0 | 0 | 0 | 0 | -61.3 | 0 | 21 | 79 | 0 | 0 | 0 |
| 11/23/2009 | 0 | 0 | 0 | 0 | -61.8 | 0 | 17 | 81 | 0 | 0 | 2 |
| 11/24/2009 | 0 | 0 | 0 | 0 | -60.8 | 0 | 18 | 80 | 0 | 0 | 2 |
| 11/25/2009 | 0 | 0 | 0 | 0 | -64.6 | 0 | 19 | 78 | 0 | 0 | 3 |
| 11/26/2009 | 0 | 0 | 0 | 0 | -65.6 | 0 | 17 | 80 | 0 | 0 | 3 |
| 11/27/2009 | 0 | 0 | 0 | 0 | -67.3 | 0 | 13 | 82 | 0 | 0 | 5 |
| 11/28/2009 | 0 | 0 | 0 | 0 | -63.9 | 0 | 18 | 82 | 0 | 0 | 0 |
| 11/29/2009 | 0 | 0 | 0 | 0 | -63.8 | 0 | 20 | 78 | 0 | 0 | 2 |
| 11/30/2009 | 0 | 0 | 0 | 0 | -62.1 | 0 | 16 | 81 | 0 | 0 | 3 |
| 12/1/2009 | 0 | 0 | 0 | 0 | -63.2 | 0 | 27 | 73 | 0 | 0 | 0 |
| 12/2/2009 | 0 | 0 | 0 | 0 | -63.2 | 0 | 29 | 71 | 0 | 0 | 1 |
| 12/3/2009 | 0 | 0 | 0 | 0 | -64.2 | 0 | 28 | 72 | 0 | 0 | 0 |
| 12/4/2009 | 0 | 0 | 0 | 0 | -59.7 | 0 | 24 | 76 | 0 | 0 | 0 |
| 12/5/2009 | 0 | 0 | 0 | 0 | -64.5 | 0 | 18 | 82 | 0 | 0 | 0 |
| 12/6/2009 | 0 | 0 | 0 | 0 | -58.5 | 0 | 29 | 71 | 0 | 0 | 0 |
| 12/7/2009 | 0 | 0 | 0 | 0 | -58.1 | 0 | 34 | 66 | 0 | 0 | 1 |
| 12/8/2009 | 0 | 0 | 0 | 0 | -59.0 | 0 | 28 | 72 | 0 | 0 | 0 |
| 12/9/2009 | 0 | 0 | 0 | 0 | -60.0 | 0 | 21 | 79 | 0 | 0 | 0 |
| 12/10/2009 | 0 | 0 | 0 | 0 | -60.1 | 0 | 20 | 80 | 0 | 0 | 0 |
| 12/11/2009 | 0 | 0 | 0 | 0 | -61.2 | 0 | 24 | 76 | 0 | 0 | 0 |
| 12/12/2009 | 0 | 0 | 0 | 0 | -61.4 | 0 | 22 | 78 | 0 | 0 | 0 |
| 12/13/2009 | 0 | 0 | 0 | 0 | -64.1 | 0 | 14 | 86 | 0 | 0 | 0 |
| 12/14/2009 | 0 | 0 | 0 | 0 | -62.6 | 0 | 20 | 80 | 0 | 0 | 0 |
| 12/15/2009 | 0 | 0 | 0 | 0 | -57.8 | 0 | 18 | 82 | 0 | 0 | 0 |
| 12/16/2009 | 0 | 0 | 0 | 0 | -59.3 | 0 | 16 | 83 | 0 | 0 | 0 |
| 12/17/2009 | 0 | 0 | 0 | 0 | -58.1 | 0 | 24 | 76 | 0 | 0 | 0 |
| 12/18/2009 | 0 | 0 | 0 | 0 | -60.3 | 0 | 20 | 80 | 0 | 0 | 0 |
| 12/19/2009 | 0 | 0 | 0 | 0 | -61.6 | 0 | 22 | 78 | 0 | 0 | 0 |
| 12/20/2009 | 0 | 0 | 0 | 0 | -61.0 | 0 | 22 | 77 | 0 | 0 | 1 |
| 12/21/2009 | 0 | 0 | 0 | 0 | -63.9 | 0 | 16 | 84 | 0 | 0 | 0 |
| 12/22/2009 | 0 | 0 | 0 | 0 | -62.6 | 0 | 22 | 78 | 0 | 0 | 0 |
| 12/23/2009 | 0 | 0 | 0 | 0 | -65.7 | 0 | 17 | 83 | 0 | 0 | 0 |
| 12/24/2009 | 0 | 0 | 0 | 0 | -64.2 | 0 | 19 | 81 | 0 | 0 | 0 |
| 12/25/2009 | 0 | 0 | 0 | 0 | -69.7 | 0 | 11 | 89 | 0 | 0 | 0 |
| 12/26/2009 | 0 | 0 | 0 | 0 | -65.2 | 0 | 20 | 80 | 0 | 0 | 0 |
| 12/27/2009 | 0 | 0 | 0 | 0 | -66.4 | 0 | 19 | 81 | 0 | 0 | 0 |
| 12/28/2009 | 0 | 0 | 0 | 0 | -65.6 | 0 | 13 | 87 | 0 | 0 | 0 |
| 12/29/2009 | 0 | 0 | 0 | 0 | -65.2 | 0 | 15 | 85 | 0 | 0 | 0 |
| 12/30/2009 | 0 | 0 | 0 | 0 | -65.4 | 0 | 21 | 79 | 0 | 0 | 0 |
| 12/31/2009 | 0 | 0 | 0 | 0 | -69.3 | 0 | 10 | 90 | 0 | 0 | 0 |
| 1/1/2010 | 0 | 0 | 0 | 0 | -69.1 | 0 | 10 | 90 | 0 | 0 | 0 |
| 1/2/2010 | 0 | 0 | 0 | 0 | -68.0 | 0 | 10 | 90 | 0 | 0 | 0 |
| 1/3/2010 | 0 | 0 | 0 | 0 | -62.1 | 0 | 23 | 77 | 0 | 0 | 0 |
| 1/4/2010 | 0 | 0 | 0 | 0 | -60.6 | 0 | 26 | 74 | 0 | 0 | 0 |
| 1/5/2010 | 0 | 0 | 0 | 0 | -60.2 | 0 | 25 | 75 | 0 | 0 | 0 |
| 1/6/2010 | 0 | 0 | 0 | 0 | -60.4 | 0 | 20 | 80 | 0 | 0 | 0 |
| 1/7/2010 | 0 | 0 | 0 | 0 | -61.3 | 0 | 22 | 78 | 0 | 0 | 0 |
| 1/8/2010 | 0 | 0 | 0 | 0 | -62.5 | 0 | 18 | 82 | 0 | 0 | 0 |
| 1/9/2010 | 0 | 0 | 0 | 0 | -61.3 | 0 | 20 | 80 | 0 | 0 | 0 |
| 1/10/2010 | 0 | 0 | 0 | 0 | -60.4 | 0 | 24 | 76 | 0 | 0 | 0 |
| 1/11/2010 | 0 | 0 | 0 | 0 | -61.4 | 0 | 18 | 82 | 0 | 0 | 0 |
| 1/12/2010 | 0 | 0 | 0 | 0 | -59.3 | 0 | 20 | 80 | 0 | 0 | 0 |
| 1/13/2010 | 0 | 0 | 0 | 0 | -60.1 | 0 | 23 | 77 | 0 | 0 | 0 |
| 1/14/2010 | 0 | 0 | 0 | 0 | -62.1 | 0 | 17 | 83 | 0 | 0 | 0 |
| 1/15/2010 | 0 | 0 | 0 | 0 | -62.4 | 0 | 19 | 81 | 0 | 0 | 0 |
| 1/16/2010 | 0 | 0 | 0 | 0 | -64.0 | 0 | 21 | 79 | 0 | 0 | 0 |
| 1/17/2010 | 0 | 0 | 0 | 0 | -61.3 | 0 | 21 | 79 | 0 | 0 | 0 |
| 1/18/2010 | 0 | 0 | 0 | 0 | -59.7 | 0 | 18 | 81 | 0 | 0 | 1 |
| 1/19/2010 | 0 | 0 | 0 | 0 | -57.7 | 0 | 23 | 76 | 0 | 0 | 1 |
| 1/20/2010 | 0 | 0 | 0 | 0 | -64.0 | 0 | 12 | 88 | 0 | 0 | 0 |
| 1/21/2010 | 0 | 0 | 0 | 0 | -63.4 | 0 | 13 | 87 | 0 | 0 | 0 |
| 1/22/2010 | 0 | 0 | 0 | 0 | -58.9 | 0 | 24 | 75 | 0 | 0 | 0 |
| 1/23/2010 | 0 | 0 | 0 | 0 | -67.6 | 0 | 14 | 86 | 0 | 0 | 0 |
| 1/24/2010 | 0 | 0 | 0 | 0 | -64.4 | 0 | 20 | 80 | 0 | 0 | 0 |
| 1/25/2010 | 0 | 0 | 0 | 0 | -62.0 | 0 | 24 | 76 | 0 | 0 | 0 |
| 1/26/2010 | 0 | 0 | 0 | 0 | -59.8 | 0 | 20 | 80 | 0 | 0 | 0 |
| 1/27/2010 | 0 | 0 | 0 | 0 | -57.7 | 0 | 22 | 78 | 0 | 0 | 0 |
| 1/28/2010 | 0 | 0 | 0 | 0 | -57.5 | 0 | 23 | 75 | 0 | 0 | 2 |
| 1/29/2010 | 0 | 0 | 0 | 0 | -67.8 | 0 | 15 | 85 | 0 | 0 | 0 |
| 1/30/2010 | 0 | 0 | 0 | 0 | -65.6 | 0 | 23 | 77 | 0 | 0 | 0 |
| 1/31/2010 | 0 | 0 | 0 | 0 | -64.0 | 0 | 22 | 78 | 0 | 0 | 0 |
| 2/1/2010 | 0 | 0 | 0 | 0 | -58.1 | 0 | 15 | 85 | 0 | 0 | 0 |
| 2/2/2010 | 0 | 0 | 0 | 0 | -60.6 | 0 | 17 | 83 | 0 | 0 | 0 |
| 2/3/2010 | 0 | 0 | 0 | 0 | -59.0 | 0 | 19 | 81 | 0 | 0 | 0 |
| 2/4/2010 | 0 | 0 | 0 | 0 | -55.8 | 0 | 17 | 82 | 0 | 0 | 1 |
| 2/5/2010 | 0 | 0 | 0 | 0 | -50.5 | 0 | 20 | 80 | 0 | 0 | 0 |
| 2/6/2010 | 0 | 0 | 0 | 0 | -58.3 | 0 | 12 | 87 | 0 | 0 | 1 |
| 2/7/2010 | 0 | 0 | 0 | 0 | -59.5 | 0 | 19 | 81 | 0 | 0 | 0 |
| 2/8/2010 | 0 | 0 | 0 | 0 | -57.0 | 0 | 17 | 82 | 0 | 0 | 1 |
| 2/9/2010 | 0 | 0 | 0 | 0 | -65.6 | 0 | 9 | 85 | 0 | 0 | 6 |
| 2/10/2010 | 0 | 0 | 0 | 0 | -61.3 | 0 | 12 | 84 | 0 | 0 | 4 |
| 2/11/2010 | 0 | 0 | 0 | 0 | -62.2 | 0 | 14 | 85 | 0 | 0 | 0 |
| 2/12/2010 | 0 | 0 | 0 | 0 | -67.0 | 0 | 11 | 85 | 0 | 0 | 5 |
| 2/13/2010 | 0 | 0 | 0 | 0 | -69.6 | 0 | 9 | 89 | 0 | 0 | 2 |
| 2/14/2010 | 0 | 0 | 0 | 0 | -62.6 | 0 | 17 | 83 | 0 | 0 | 0 |
| 2/15/2010 | 0 | 0 | 0 | 0 | -60.7 | 0 | 20 | 80 | 0 | 0 | 0 |
| 2/16/2010 | 0 | 0 | 0 | 0 | -62.5 | 0 | 17 | 83 | 0 | 0 | 0 |
| 2/17/2010 | 0 | 0 | 0 | 0 | -63.3 | 0 | 16 | 83 | 0 | 0 | 1 |
| 2/18/2010 | 0 | 0 | 0 | 0 | -61.7 | 0 | 18 | 82 | 0 | 0 | 0 |
| 2/19/2010 | 0 | 0 | 0 | 0 | -62.6 | 0 | 18 | 81 | 0 | 0 | 2 |
| 2/20/2010 | 0 | 0 | 0 | 0 | -64.8 | 0 | 14 | 83 | 0 | 0 | 3 |
| 2/21/2010 | 0 | 0 | 0 | 0 | -61.2 | 0 | 22 | 78 | 0 | 0 | 1 |
| 2/22/2010 | 0 | 0 | 0 | 0 | -58.5 | 0 | 24 | 76 | 0 | 0 | 0 |
| 2/23/2010 | 0 | 0 | 0 | 0 | -58.6 | 0 | 23 | 77 | 0 | 0 | 0 |
| 2/24/2010 | 0 | 0 | 0 | 0 | -58.4 | 0 | 24 | 76 | 0 | 0 | 0 |
| 2/25/2010 | 0 | 0 | 0 | 0 | -58.5 | 0 | 21 | 79 | 0 | 0 | 0 |
| 2/26/2010 | 0 | 0 | 0 | 0 | -56.7 | 0 | 20 | 80 | 0 | 0 | 0 |
| 2/27/2010 | 0 | 0 | 0 | 0 | -52.8 | 0 | 15 | 85 | 0 | 0 | 0 |
| 2/28/2010 | 0 | 0 | 0 | 0 | -58.0 | 0 | 15 | 83 | 0 | 0 | 0 |
| 3/1/2010 | 0 | 0 | 20 | 5 | -60.1 | NA | NA | NA | NA | NA | NA |
| 3/2/2010 | 0 | 0 | 20 | 5 | -61.4 | NA | NA | NA | NA | NA | NA |
| 3/3/2010 | 0 | 0 | 20 | 5 | -61.0 | NA | NA | NA | NA | NA | NA |
| 3/4/2010 | 0 | 0 | 20 | 5 | -58.2 | NA | NA | NA | NA | NA | NA |
| 3/5/2010 | 0 | 0 | 40 | 10 | -56.8 | NA | NA | NA | NA | NA | NA |
| 3/6/2010 | 0 | 0 | 40 | 10 | -58.8 | NA | NA | NA | NA | NA | NA |
| 3/7/2010 | 1 | 0 | 40 | 10 | -60.7 | NA | NA | NA | NA | NA | NA |
| 3/8/2010 | 1 | 0 | 50 | 20 | -60.5 | NA | NA | NA | NA | NA | NA |
| 3/9/2010 | 1 | 0 | 50 | 20 | -61.3 | NA | NA | NA | NA | NA | NA |
| 3/10/2010 | 1 | 0 | 50 | 20 | -63.0 | NA | NA | NA | NA | NA | NA |
| 3/11/2010 | 1 | 0 | 50 | 20 | -63.1 | NA | NA | NA | NA | NA | NA |
| 3/12/2010 | 1 | 1 | 70 | 30 | -62.7 | NA | NA | NA | NA | NA | NA |
| 3/13/2010 | 1 | 0 | 70 | 30 | -62.0 | NA | NA | NA | NA | NA | NA |
| 3/14/2010 | 1 | 0 | 70 | 30 | -63.7 | NA | NA | NA | NA | NA | NA |
| 3/15/2010 | 1 | 1 | 85 | 20 | -65.2 | NA | NA | NA | NA | NA | NA |
| 3/16/2010 | 1 | 0 | 85 | 20 | -64.8 | NA | NA | NA | NA | NA | NA |
| 3/17/2010 | 1 | 0 | 80 | 30 | -67.8 | NA | NA | NA | NA | NA | NA |
| 3/18/2010 | 1 | 0 | 85 | 30 | -62.9 | NA | NA | NA | NA | NA | NA |
| 3/19/2010 | 1 | 0 | 85 | 51 | -69.6 | NA | NA | NA | NA | NA | NA |
| 3/20/2010 | 1 | 0 | 85 | 51 | -65.9 | NA | NA | NA | NA | NA | NA |
| 3/21/2010 | 1 | 0 | 85 | 51 | -63.9 | NA | NA | NA | NA | NA | NA |
| 3/22/2010 | 0 | 0 | 80 | 30 | -67.1 | NA | NA | NA | NA | NA | NA |
| 3/23/2010 | 1 | 0 | 80 | 30 | -63.4 | NA | NA | NA | NA | NA | NA |
| 3/24/2010 | 1 | 1 | 80 | 30 | -64.3 | NA | NA | NA | NA | NA | NA |
| 3/25/2010 | 1 | 0 | 80 | 30 | -66.9 | NA | NA | NA | NA | NA | NA |
| 3/26/2010 | 0 | 0 | 80 | 30 | -68.1 | NA | NA | NA | NA | NA | NA |
| 3/27/2010 | 0 | 0 | 80 | 30 | -64.8 | NA | NA | NA | NA | NA | NA |
| 3/28/2010 | 1 | 0 | 80 | 30 | -72.3 | NA | NA | NA | NA | NA | NA |
| 3/29/2010 | 1 | 0 | 80 | 20 | -72.6 | NA | NA | NA | NA | NA | NA |
| 3/30/2010 | 1 | 0 | 80 | 20 | -77.3 | NA | NA | NA | NA | NA | NA |
| 3/31/2010 | 1 | 0 | 75 | 30 | -72.6 | NA | NA | NA | NA | NA | NA |
| 4/1/2010 | 1 | 1 | 75 | 30 | -75.0 | NA | NA | NA | NA | NA | NA |
| 4/2/2010 | 1 | 0 | 75 | 30 | -77.1 | NA | NA | NA | NA | NA | NA |
| 4/3/2010 | 1 | 0 | 75 | 30 | -70.7 | NA | NA | NA | NA | NA | NA |
| 4/4/2010 | 1 | 0 | 75 | 30 | -73.2 | NA | NA | NA | NA | NA | NA |
| 4/5/2010 | 1 | 0 | 75 | 30 | -71.6 | NA | NA | NA | NA | NA | NA |
| 4/6/2010 | 1 | 0 | 75 | 30 | -71.7 | NA | NA | NA | NA | NA | NA |
| 4/7/2010 | 1 | 1 | 80 | 30 | -66.6 | NA | NA | NA | NA | NA | NA |
| 4/8/2010 | 1 | 0 | 80 | 30 | -65.5 | NA | NA | NA | NA | NA | NA |
| 4/9/2010 | 1 | 1 | 75 | 30 | -66.8 | NA | NA | NA | NA | NA | NA |
| 4/10/2010 | 1 | 1 | 75 | 30 | -73.1 | NA | NA | NA | NA | NA | NA |
| 4/11/2010 | 0 | 0 | 75 | 30 | -62.1 | NA | NA | NA | NA | NA | NA |
| 4/12/2010 | 0 | 0 | 75 | 30 | -55.9 | NA | NA | NA | NA | NA | NA |
| 4/13/2010 | 0 | 0 | 75 | 30 | -62.1 | NA | NA | NA | NA | NA | NA |
| 4/14/2010 | 0 | 0 | 0 | 0 | -63.0 | NA | NA | NA | NA | NA | NA |
| 4/15/2010 | 0 | 1 | 0 | 0 | -63.0 | NA | NA | NA | NA | NA | NA |
| 4/16/2010 | 1 | 0 | 0 | 0 | -62.5 | NA | NA | NA | NA | NA | NA |
| 4/17/2010 | 0 | 1 | 0 | 0 | -69.0 | NA | NA | NA | NA | NA | NA |
| 4/18/2010 | 0 | 0 | 0 | 0 | -64.5 | NA | NA | NA | NA | NA | NA |
| 4/19/2010 | 0 | 0 | 0 | 0 | -59.3 | NA | NA | NA | NA | NA | NA |
| 4/20/2010 | 0 | 0 | 0 | 0 | -62.1 | NA | NA | NA | NA | NA | NA |
| 4/21/2010 | 0 | 0 | 0 | 0 | -65.5 | NA | NA | NA | NA | NA | NA |
| 4/22/2010 | 0 | 0 | 0 | 0 | -62.8 | NA | NA | NA | NA | NA | NA |
| 4/23/2010 | 0 | 0 | 0 | 0 | -64.2 | NA | NA | NA | NA | NA | NA |
| 4/24/2010 | 0 | 0 | 0 | 0 | -62.0 | NA | NA | NA | NA | NA | NA |
| 4/25/2010 | 0 | 0 | 0 | 0 | -62.0 | NA | NA | NA | NA | NA | NA |
| 4/26/2010 | 0 | 0 | 0 | 0 | -61.9 | NA | NA | NA | NA | NA | NA |
| 4/27/2010 | 0 | 0 | 0 | 0 | -59.2 | NA | NA | NA | NA | NA | NA |
| 4/28/2010 | 0 | 1 | 0 | 0 | -52.7 | NA | NA | NA | NA | NA | NA |
| 4/29/2010 | 0 | 0 | 0 | 0 | -74.0 | NA | NA | NA | NA | NA | NA |
| 4/30/2010 | 0 | 0 | 40 | 20 | -72.9 | NA | NA | NA | NA | NA | NA |
| 5/1/2010 | 0 | 0 | 40 | 20 | -73.8 | NA | NA | NA | NA | NA | NA |
| 5/2/2010 | 0 | 1 | 40 | 20 | -72.0 | NA | NA | NA | NA | NA | NA |
| 5/3/2010 | 1 | 1 | 60 | 30 | -72.5 | NA | NA | NA | NA | NA | NA |
| 5/4/2010 | 1 | 1 | 60 | 30 | -70.8 | NA | NA | NA | NA | NA | NA |
| 5/5/2010 | 0 | 1 | 60 | 30 | -71.0 | NA | NA | NA | NA | NA | NA |
| 5/6/2010 | 0 | 0 | 60 | 30 | -70.9 | NA | NA | NA | NA | NA | NA |
| 5/7/2010 | 0 | 0 | 0 | 0 | -70.7 | NA | NA | NA | NA | NA | NA |
| 5/8/2010 | 0 | 0 | 0 | 0 | -70.6 | NA | NA | NA | NA | NA | NA |
| 5/9/2010 | 0 | 0 | 0 | 0 | -73.7 | NA | NA | NA | NA | NA | NA |
| 5/10/2010 | 0 | 0 | 0 | 0 | -72.4 | NA | NA | NA | NA | NA | NA |
| 5/11/2010 | 0 | 0 | 0 | 0 | -72.0 | NA | NA | NA | NA | NA | NA |
| 5/12/2010 | 0 | 0 | 0 | 0 | -74.0 | NA | NA | NA | NA | NA | NA |
| 5/13/2010 | 0 | 0 | 0 | 0 | -75.0 | NA | NA | NA | NA | NA | NA |
| 5/14/2010 | 0 | 0 | 0 | 0 | -73.2 | NA | NA | NA | NA | NA | NA |
| 5/15/2010 | 0 | 0 | 0 | 0 | -68.9 | NA | NA | NA | NA | NA | NA |
| 5/16/2010 | 0 | 0 | 0 | 0 | -73.3 | NA | NA | NA | NA | NA | NA |
| 5/17/2010 | 0 | 0 | 0 | 0 | -75.9 | NA | NA | NA | NA | NA | NA |
| 5/18/2010 | 0 | 0 | 0 | 0 | -76.3 | NA | NA | NA | NA | NA | NA |
| 5/19/2010 | 0 | 0 | 0 | 0 | -77.7 | NA | NA | NA | NA | NA | NA |
| 5/20/2010 | 0 | 0 | 0 | 0 | -74.4 | NA | NA | NA | NA | NA | NA |
| 5/21/2010 | 0 | 0 | 0 | 0 | -79.1 | NA | NA | NA | NA | NA | NA |
| 5/22/2010 | 0 | 0 | 0 | 0 | -79.7 | NA | NA | NA | NA | NA | NA |
| 5/23/2010 | 0 | 0 | 0 | 0 | -79.1 | NA | NA | NA | NA | NA | NA |
| 5/24/2010 | 0 | 0 | 0 | 0 | -80.3 | NA | NA | NA | NA | NA | NA |
| 5/25/2010 | 0 | 0 | 0 | 0 | -80.2 | NA | NA | NA | NA | NA | NA |
| 5/26/2010 | 0 | 0 | 0 | 0 | -77.1 | NA | NA | NA | NA | NA | NA |
| 5/27/2010 | 0 | 0 | 0 | 0 | -72.0 | NA | NA | NA | NA | NA | NA |
| 5/28/2010 | 0 | 0 | 0 | 0 | -73.3 | NA | NA | NA | NA | NA | NA |
| 5/29/2010 | 0 | 0 | 0 | 0 | -78.8 | NA | NA | NA | NA | NA | NA |
| 5/30/2010 | 0 | 0 | 0 | 0 | -79.9 | NA | NA | NA | NA | NA | NA |
| 5/31/2010 | 0 | 0 | 0 | 0 | -79.8 | NA | NA | NA | NA | NA | NA |
| 6/1/2010 | 0 | 0 | 0 | 0 | -77.2 | NA | NA | NA | NA | NA | NA |
| 6/2/2010 | 0 | 0 | 0 | 0 | -75.0 | NA | NA | NA | NA | NA | NA |
| 6/3/2010 | 0 | 0 | 0 | 0 | -76.4 | NA | NA | NA | NA | NA | NA |
| 6/4/2010 | 0 | 0 | 0 | 0 | -75.5 | NA | NA | NA | NA | NA | NA |
| 6/5/2010 | 0 | 0 | 0 | 0 | -76.2 | NA | NA | NA | NA | NA | NA |
| 6/6/2010 | 0 | 0 | 0 | 0 | -75.3 | NA | NA | NA | NA | NA | NA |
| 6/7/2010 | 0 | 0 | 0 | 0 | -74.7 | NA | NA | NA | NA | NA | NA |
| 6/8/2010 | 0 | 0 | 0 | 0 | -77.3 | NA | NA | NA | NA | NA | NA |
| 6/9/2010 | 0 | 0 | 0 | 0 | -75.4 | NA | NA | NA | NA | NA | NA |
| 6/10/2010 | 0 | 0 | 0 | 0 | -68.5 | NA | NA | NA | NA | NA | NA |
| 6/11/2010 | 0 | 0 | 0 | 0 | -72.1 | NA | NA | NA | NA | NA | NA |
| 6/12/2010 | 0 | 0 | 0 | 0 | -75.0 | NA | NA | NA | NA | NA | NA |
| 6/13/2010 | 0 | 0 | 0 | 0 | -74.3 | NA | NA | NA | NA | NA | NA |
| 6/14/2010 | 0 | 0 | 0 | 0 | -70.4 | NA | NA | NA | NA | NA | NA |
| 6/15/2010 | 0 | 0 | 0 | 0 | -67.3 | NA | NA | NA | NA | NA | NA |
| 6/16/2010 | 0 | 0 | 0 | 0 | -70.7 | NA | NA | NA | NA | NA | NA |
| 6/17/2010 | 0 | 0 | 0 | 0 | -68.0 | NA | NA | NA | NA | NA | NA |
| 6/18/2010 | 0 | 0 | 0 | 0 | -69.5 | NA | NA | NA | NA | NA | NA |
| 6/19/2010 | 0 | 0 | 0 | 0 | -69.4 | NA | NA | NA | NA | NA | NA |
| 6/20/2010 | 0 | 0 | 0 | 0 | -70.5 | NA | NA | NA | NA | NA | NA |
| 6/21/2010 | 0 | 0 | 0 | 0 | -72.1 | NA | NA | NA | NA | NA | NA |
| 6/22/2010 | 0 | 0 | 0 | 0 | -71.6 | NA | NA | NA | NA | NA | NA |
| 6/23/2010 | 0 | 0 | 0 | 0 | -71.1 | NA | NA | NA | NA | NA | NA |
| 6/24/2010 | 0 | 0 | 0 | 0 | -72.2 | NA | NA | NA | NA | NA | NA |
| 6/25/2010 | 0 | 0 | 0 | 0 | -72.8 | NA | NA | NA | NA | NA | NA |
| 6/26/2010 | 0 | 0 | 0 | 0 | -72.6 | NA | NA | NA | NA | NA | NA |
| 6/27/2010 | 0 | 0 | 0 | 0 | -72.1 | NA | NA | NA | NA | NA | NA |
| 6/28/2010 | 0 | 0 | 0 | 0 | -70.4 | NA | NA | NA | NA | NA | NA |
| 6/29/2010 | 0 | 0 | 0 | 0 | -68.1 | NA | NA | NA | NA | NA | NA |
| 6/30/2010 | 0 | 0 | 0 | 0 | -70.5 | NA | NA | NA | NA | NA | NA |
| 7/1/2010 | 0 | 0 | 0 | 0 | -68.8 | NA | NA | NA | NA | NA | NA |
| 7/2/2010 | 0 | 0 | 0 | 0 | -68.8 | NA | NA | NA | NA | NA | NA |
| 7/3/2010 | 0 | 0 | 0 | 0 | -68.8 | NA | NA | NA | NA | NA | NA |
| 7/4/2010 | 0 | 0 | 0 | 0 | -68.8 | NA | NA | NA | NA | NA | NA |
| 7/5/2010 | 0 | 0 | 0 | 0 | -68.8 | NA | NA | NA | NA | NA | NA |
| 7/6/2010 | 0 | 0 | 0 | 0 | -68.8 | NA | NA | NA | NA | NA | NA |
| 7/7/2010 | 0 | 0 | 0 | 0 | -68.8 | NA | NA | NA | NA | NA | NA |
| 7/8/2010 | 0 | 0 | 0 | 0 | -68.8 | NA | NA | NA | NA | NA | NA |
| 7/9/2010 | 0 | 0 | 0 | 0 | -68.1 | NA | NA | NA | NA | NA | NA |
| 7/10/2010 | 0 | 0 | 0 | 0 | -68.7 | NA | NA | NA | NA | NA | NA |
| 7/11/2010 | 0 | 0 | 0 | 0 | -68.8 | NA | NA | NA | NA | NA | NA |
| 7/12/2010 | 0 | 0 | 0 | 0 | -68.8 | NA | NA | NA | NA | NA | NA |
| 7/13/2010 | 0 | 0 | 0 | 0 | -68.8 | NA | NA | NA | NA | NA | NA |
| 7/14/2010 | 0 | 0 | 0 | 0 | -68.8 | NA | NA | NA | NA | NA | NA |
| 7/15/2010 | 0 | 0 | 0 | 0 | -68.8 | NA | NA | NA | NA | NA | NA |
| 7/16/2010 | 0 | 0 | 0 | 0 | -68.7 | NA | NA | NA | NA | NA | NA |
| 7/17/2010 | 0 | 0 | 0 | 0 | -68.8 | NA | NA | NA | NA | NA | NA |
| 7/18/2010 | 0 | 0 | 0 | 0 | -68.8 | NA | NA | NA | NA | NA | NA |
| 7/19/2010 | 0 | 0 | 0 | 0 | -68.7 | NA | NA | NA | NA | NA | NA |
| 7/20/2010 | 0 | 0 | 0 | 0 | -68.7 | NA | NA | NA | NA | NA | NA |
| 7/21/2010 | 0 | 0 | 0 | 0 | -68.6 | NA | NA | NA | NA | NA | NA |
| 7/22/2010 | 0 | 0 | 0 | 0 | -68.7 | NA | NA | NA | NA | NA | NA |
| 7/23/2010 | 0 | 0 | 0 | 0 | -68.5 | NA | NA | NA | NA | NA | NA |
| 7/24/2010 | 0 | 0 | 0 | 0 | -68.6 | NA | NA | NA | NA | NA | NA |
| 7/25/2010 | 0 | 0 | 0 | 0 | -68.7 | NA | NA | NA | NA | NA | NA |
| 7/26/2010 | 0 | 0 | 0 | 0 | -68.7 | NA | NA | NA | NA | NA | NA |
| 7/27/2010 | 0 | 0 | 0 | 0 | -68.8 | NA | NA | NA | NA | NA | NA |
| 7/28/2010 | 0 | 0 | 0 | 0 | -68.8 | NA | NA | NA | NA | NA | NA |
| 7/29/2010 | 0 | 0 | 0 | 0 | -68.8 | NA | NA | NA | NA | NA | NA |
| 7/30/2010 | 0 | 0 | 0 | 0 | -68.7 | NA | NA | NA | NA | NA | NA |
| 7/31/2010 | 0 | 0 | 0 | 0 | -68.7 | NA | NA | NA | NA | NA | NA |
| 8/1/2010 | 0 | 0 | 0 | 0 | -75.4 | NA | NA | NA | NA | NA | NA |
| 8/2/2010 | 0 | 0 | 0 | 0 | -73.8 | NA | NA | NA | NA | NA | NA |
| 8/3/2010 | 0 | 0 | 0 | 0 | -74.4 | NA | NA | NA | NA | NA | NA |
| 8/4/2010 | 0 | 0 | 0 | 0 | -75.4 | NA | NA | NA | NA | NA | NA |
| 8/5/2010 | 0 | 0 | 0 | 0 | -75.0 | NA | NA | NA | NA | NA | NA |
| 8/6/2010 | 0 | 0 | 0 | 0 | -72.9 | NA | NA | NA | NA | NA | NA |
| 8/7/2010 | 0 | 0 | 0 | 0 | -71.7 | NA | NA | NA | NA | NA | NA |
| 8/8/2010 | 0 | 0 | 0 | 0 | -72.8 | NA | NA | NA | NA | NA | NA |
| 8/9/2010 | 0 | 0 | 0 | 0 | -73.9 | NA | NA | NA | NA | NA | NA |
| 8/10/2010 | 0 | 0 | 0 | 0 | -75.6 | NA | NA | NA | NA | NA | NA |
| 8/11/2010 | 0 | 0 | 0 | 0 | -76.0 | NA | NA | NA | NA | NA | NA |
| 8/12/2010 | 0 | 0 | 0 | 0 | -74.4 | NA | NA | NA | NA | NA | NA |
| 8/13/2010 | 0 | 0 | 0 | 0 | -74.4 | NA | NA | NA | NA | NA | NA |
| 8/14/2010 | 0 | 0 | 0 | 0 | -74.4 | NA | NA | NA | NA | NA | NA |
| 8/15/2010 | 0 | 0 | 0 | 0 | -75.6 | NA | NA | NA | NA | NA | NA |
| 8/16/2010 | 0 | 0 | 0 | 0 | -74.3 | NA | NA | NA | NA | NA | NA |
| 8/17/2010 | 0 | 0 | 0 | 0 | -73.4 | NA | NA | NA | NA | NA | NA |
| 8/18/2010 | 0 | 0 | 0 | 0 | -74.9 | NA | NA | NA | NA | NA | NA |
| 8/19/2010 | 0 | 0 | 0 | 0 | -74.8 | NA | NA | NA | NA | NA | NA |
| 8/20/2010 | 0 | 0 | 0 | 0 | -74.5 | NA | NA | NA | NA | NA | NA |
| 8/21/2010 | 0 | 0 | 0 | 0 | -73.3 | NA | NA | NA | NA | NA | NA |
| 8/22/2010 | 0 | 0 | 0 | 0 | -73.3 | NA | NA | NA | NA | NA | NA |
| 8/23/2010 | 0 | 0 | 0 | 0 | -74.2 | NA | NA | NA | NA | NA | NA |
| 8/24/2010 | 0 | 0 | 0 | 0 | -74.8 | NA | NA | NA | NA | NA | NA |
| 8/25/2010 | 0 | 0 | 0 | 0 | -75.1 | NA | NA | NA | NA | NA | NA |
| 8/26/2010 | 0 | 0 | 0 | 0 | -75.0 | NA | NA | NA | NA | NA | NA |
| 8/27/2010 | 0 | 0 | 0 | 0 | -74.6 | NA | NA | NA | NA | NA | NA |
| 8/28/2010 | 0 | 0 | 0 | 0 | -75.1 | NA | NA | NA | NA | NA | NA |
| 8/29/2010 | 0 | 0 | 0 | 0 | -75.4 | NA | NA | NA | NA | NA | NA |
| 8/30/2010 | 0 | 0 | 0 | 0 | -75.7 | NA | NA | NA | NA | NA | NA |
| 8/31/2010 | 0 | 0 | 0 | 0 | -75.6 | NA | NA | NA | NA | NA | NA |
| 9/1/2010 | 0 | 0 | 0 | 0 | -68.7 | NA | NA | NA | NA | NA | NA |
| 9/2/2010 | 0 | 0 | 0 | 0 | -68.7 | NA | NA | NA | NA | NA | NA |
| 9/3/2010 | 0 | 0 | 0 | 0 | -68.5 | NA | NA | NA | NA | NA | NA |
| 9/4/2010 | 0 | 0 | 0 | 0 | -68.6 | NA | NA | NA | NA | NA | NA |
| 9/5/2010 | 0 | 0 | 0 | 0 | -68.7 | NA | NA | NA | NA | NA | NA |
| 9/6/2010 | 0 | 0 | 0 | 0 | -68.6 | NA | NA | NA | NA | NA | NA |
| 9/7/2010 | 0 | 0 | 0 | 0 | -68.3 | NA | NA | NA | NA | NA | NA |
| 9/8/2010 | 0 | 0 | 0 | 0 | -67.9 | NA | NA | NA | NA | NA | NA |
| 9/9/2010 | 0 | 0 | 0 | 0 | -68.6 | NA | NA | NA | NA | NA | NA |
| 9/10/2010 | 0 | 0 | 0 | 0 | -68.7 | NA | NA | NA | NA | NA | NA |
| 9/11/2010 | 0 | 0 | 0 | 0 | -68.7 | NA | NA | NA | NA | NA | NA |
| 9/12/2010 | 0 | 0 | 0 | 0 | -68.7 | NA | NA | NA | NA | NA | NA |
| 9/13/2010 | 0 | 0 | 0 | 0 | -68.5 | NA | NA | NA | NA | NA | NA |
| 9/14/2010 | 0 | 0 | 0 | 0 | -68.6 | NA | NA | NA | NA | NA | NA |
| 9/15/2010 | 0 | 0 | 0 | 0 | -68.7 | NA | NA | NA | NA | NA | NA |
| 9/16/2010 | 0 | 0 | 0 | 0 | -68.7 | NA | NA | NA | NA | NA | NA |
| 9/17/2010 | 0 | 0 | 0 | 0 | -68.6 | NA | NA | NA | NA | NA | NA |
| 9/18/2010 | 0 | 0 | 0 | 0 | -68.6 | NA | NA | NA | NA | NA | NA |
| 9/19/2010 | 0 | 0 | 0 | 0 | -68.7 | NA | NA | NA | NA | NA | NA |
| 9/20/2010 | 0 | 0 | 0 | 0 | -68.7 | NA | NA | NA | NA | NA | NA |
| 9/21/2010 | 0 | 0 | 0 | 0 | -68.6 | NA | NA | NA | NA | NA | NA |
| 9/22/2010 | 0 | 0 | 0 | 0 | -68.7 | NA | NA | NA | NA | NA | NA |
| 9/23/2010 | 0 | 0 | 0 | 0 | -68.2 | NA | NA | NA | NA | NA | NA |
| 9/24/2010 | 0 | 0 | 0 | 0 | -68.3 | NA | NA | NA | NA | NA | NA |
| 9/25/2010 | 0 | 0 | 0 | 0 | -67.6 | NA | NA | NA | NA | NA | NA |
| 9/26/2010 | 0 | 0 | 0 | 0 | -67.5 | NA | NA | NA | NA | NA | NA |
| 9/27/2010 | 0 | 0 | 0 | 0 | -68.5 | NA | NA | NA | NA | NA | NA |
| 9/28/2010 | 0 | 0 | 0 | 0 | -68.3 | NA | NA | NA | NA | NA | NA |
| 9/29/2010 | 0 | 0 | 0 | 0 | -66.8 | NA | NA | NA | NA | NA | NA |
| 9/30/2010 | 0 | 0 | 0 | 0 | -67.2 | NA | NA | NA | NA | NA | NA |
| 10/1/2010 | 0 | 0 | 0 | 0 | NA | NA | NA | NA | NA | NA | NA |
| 10/2/2010 | 0 | 0 | 0 | 0 | NA | NA | NA | NA | NA | NA | NA |
| 10/3/2010 | 0 | 0 | 0 | 0 | NA | NA | NA | NA | NA | NA | NA |
| 10/4/2010 | 0 | 0 | 0 | 0 | NA | NA | NA | NA | NA | NA | NA |
| 10/5/2010 | 0 | 0 | 0 | 0 | -61.8 | 0 | 37 | 32 | 30 | 0 | 0 |
| 10/6/2010 | 0 | 0 | 0 | 0 | -63.0 | 0 | 32 | 40 | 28 | 0 | 0 |
| 10/7/2010 | 0 | 0 | 0 | 0 | -62.6 | 0 | 29 | 41 | 30 | 0 | 0 |
| 10/8/2010 | 0 | 0 | 0 | 0 | -62.2 | 0 | 31 | 35 | 34 | 0 | 0 |
| 10/9/2010 | 0 | 0 | 0 | 0 | -61.9 | 1 | 32 | 35 | 32 | 0 | 0 |
| 10/10/2010 | 0 | 0 | 0 | 0 | -61.5 | 1 | 22 | 38 | 39 | 0 | 0 |
| 10/11/2010 | 0 | 0 | 0 | 0 | -61.6 | 0 | 18 | 34 | 48 | 0 | 0 |
| 10/12/2010 | 0 | 0 | 0 | 0 | -59.7 | 1 | 21 | 26 | 53 | 0 | 0 |
| 10/13/2010 | 0 | 0 | 0 | 0 | -59.8 | 0 | 26 | 28 | 45 | 0 | 0 |
| 10/14/2010 | 0 | 0 | 0 | 0 | -58.7 | 0 | 22 | 27 | 50 | 0 | 0 |
| 10/15/2010 | 0 | 0 | 0 | 0 | -50.6 | 1 | 26 | 10 | 63 | 0 | 0 |
| 10/16/2010 | 0 | 0 | 0 | 0 | -51.2 | 2 | 34 | 13 | 51 | 0 | 0 |
| 10/17/2010 | 0 | 0 | 0 | 0 | -57.5 | 0 | 35 | 12 | 53 | 1 | 0 |
| 10/18/2010 | 0 | 0 | 0 | 0 | -58.1 | 0 | 54 | 8 | 38 | 0 | 0 |
| 10/19/2010 | 0 | 0 | 0 | 0 | -58.7 | 1 | 43 | 22 | 34 | 0 | 0 |
| 10/20/2010 | 0 | 0 | 0 | 0 | -57.6 | 8 | 32 | 22 | 38 | 0 | 0 |
| 10/21/2010 | 0 | 0 | 0 | 0 | -56.8 | 1 | 41 | 17 | 41 | 0 | 0 |
| 10/22/2010 | 0 | 0 | 0 | 0 | -56.5 | 0 | 40 | 21 | 39 | 0 | 0 |
| 10/23/2010 | 0 | 0 | 0 | 0 | -56.5 | 2 | 44 | 18 | 36 | 0 | 0 |
| 10/24/2010 | 0 | 0 | 0 | 0 | -57.9 | 1 | 48 | 19 | 32 | 0 | 0 |
| 10/25/2010 | 0 | 0 | 0 | 0 | -57.3 | 0 | 45 | 23 | 31 | 0 | 0 |
| 10/26/2010 | 0 | 0 | 0 | 0 | -57.9 | 1 | 42 | 17 | 40 | 0 | 0 |
| 10/27/2010 | 0 | 0 | 0 | 0 | -57.3 | 0 | 41 | 19 | 40 | 0 | 0 |
| 10/28/2010 | 0 | 0 | 0 | 0 | -57.4 | 0 | 40 | 15 | 45 | 0 | 0 |
| 10/29/2010 | 0 | 0 | 0 | 0 | -57.1 | 0 | 46 | 10 | 43 | 0 | 0 |
| 10/30/2010 | 0 | 0 | 0 | 0 | -55.6 | 0 | 50 | 18 | 32 | 0 | 0 |
| 10/31/2010 | 0 | 0 | 0 | 0 | -55.8 | 0 | 51 | 22 | 26 | 0 | 0 |
| 11/1/2010 | 0 | 0 | 0 | 0 | -56.7 | 0 | 51 | 15 | 33 | 0 | 0 |
| 11/2/2010 | 0 | 0 | 0 | 0 | -57.7 | 0 | 52 | 11 | 37 | 0 | 0 |
| 11/3/2010 | 0 | 0 | 0 | 0 | -57.2 | 0 | 52 | 15 | 33 | 0 | 0 |
| 11/4/2010 | 0 | 0 | 0 | 0 | -58.0 | 1 | 56 | 14 | 29 | 0 | 0 |
| 11/5/2010 | 0 | 0 | 0 | 0 | -57.7 | 0 | 54 | 15 | 30 | 0 | 0 |
| 11/6/2010 | 0 | 0 | 0 | 0 | -57.5 | 0 | 51 | 13 | 36 | 0 | 0 |
| 11/7/2010 | 0 | 0 | 0 | 0 | -57.3 | 0 | 45 | 21 | 34 | 0 | 0 |
| 11/8/2010 | 0 | 0 | 0 | 0 | -57.0 | 0 | 39 | 23 | 37 | 0 | 0 |
| 11/9/2010 | 0 | 0 | 0 | 0 | -57.0 | 0 | 28 | 22 | 50 | 0 | 0 |
| 11/10/2010 | 0 | 0 | 0 | 0 | -55.9 | 0 | 28 | 11 | 61 | 0 | 0 |
| 11/11/2010 | 0 | 0 | 0 | 0 | -55.0 | 0 | 32 | 14 | 53 | 0 | 0 |
| 11/12/2010 | 0 | 0 | 0 | 0 | -54.2 | 0 | 34 | 14 | 52 | 0 | 0 |
| 11/13/2010 | 0 | 0 | 0 | 0 | -55.3 | 0 | 42 | 13 | 44 | 0 | 0 |
| 11/14/2010 | 0 | 0 | 0 | 0 | -55.7 | 0 | 39 | 14 | 46 | 0 | 0 |
| 11/15/2010 | 0 | 0 | 0 | 0 | -56.7 | 0 | 23 | 28 | 49 | 0 | 0 |
| 11/16/2010 | 0 | 0 | 0 | 0 | -56.1 | 0 | 12 | 30 | 58 | 0 | 0 |
| 11/17/2010 | 0 | 0 | 0 | 0 | -55.9 | 0 | 22 | 25 | 53 | 0 | 0 |
| 11/18/2010 | 0 | 0 | 0 | 0 | -55.5 | 0 | 18 | 19 | 62 | 0 | 0 |
| 11/19/2010 | 0 | 0 | 0 | 0 | -55.4 | 0 | 22 | 20 | 57 | 0 | 0 |
| 11/20/2010 | 0 | 0 | 0 | 0 | -56.5 | 0 | 30 | 23 | 47 | 0 | 0 |
| 11/21/2010 | 0 | 0 | 0 | 0 | -55.5 | 0 | 29 | 18 | 52 | 1 | 0 |
| 11/22/2010 | 0 | 0 | 0 | 0 | -55.6 | 0 | 34 | 17 | 48 | 0 | 0 |
| 11/23/2010 | 0 | 0 | 0 | 0 | -55.9 | 0 | 28 | 17 | 55 | 0 | 0 |
| 11/24/2010 | 0 | 0 | 0 | 0 | -55.4 | 0 | 37 | 16 | 47 | 0 | 0 |
| 11/25/2010 | 0 | 0 | 0 | 0 | -56.6 | 0 | 24 | 18 | 58 | 0 | 0 |
| 11/26/2010 | 0 | 0 | 0 | 0 | -58.0 | 0 | 23 | 13 | 63 | 0 | 0 |
| 11/27/2010 | 0 | 0 | 0 | 0 | -56.8 | 0 | 26 | 17 | 57 | 0 | 0 |
| 11/28/2010 | 0 | 0 | 0 | 0 | -59.6 | 1 | 25 | 21 | 53 | 1 | 0 |
| 11/29/2010 | 0 | 0 | 0 | 0 | -58.4 | 2 | 27 | 23 | 48 | 0 | 0 |
| 11/30/2010 | 0 | 0 | 0 | 0 | -58.2 | 0 | 33 | 23 | 43 | 0 | 0 |
| 12/1/2010 | 0 | 0 | 0 | 0 | -58.5 | 0 | 30 | 22 | 48 | 0 | 0 |
| 12/2/2010 | 0 | 0 | 0 | 0 | -58.2 | 0 | 26 | 24 | 50 | 0 | 0 |
| 12/3/2010 | 0 | 0 | 0 | 0 | -56.8 | 0 | 47 | 8 | 44 | 0 | 0 |
| 12/4/2010 | 0 | 0 | 0 | 0 | -55.7 | 4 | 72 | 1 | 23 | 0 | 0 |
| 12/5/2010 | 0 | 0 | 0 | 0 | -54.9 | 16 | 68 | 0 | 17 | 0 | 0 |
| 12/6/2010 | 0 | 0 | 0 | 0 | -56.9 | 5 | 81 | 0 | 13 | 0 | 0 |
| 12/7/2010 | 0 | 0 | 0 | 0 | -57.6 | 3 | 78 | 1 | 19 | 0 | 0 |
| 12/8/2010 | 0 | 0 | 0 | 0 | -55.3 | 1 | 73 | 1 | 24 | 0 | 0 |
| 12/9/2010 | 0 | 0 | 0 | 0 | -55.6 | 1 | 69 | 1 | 29 | 0 | 0 |
| 12/10/2010 | 0 | 0 | 0 | 0 | -55.9 | 0 | 58 | 1 | 41 | 0 | 0 |
| 12/11/2010 | 0 | 0 | 0 | 0 | -57.4 | 0 | 37 | 9 | 53 | 0 | 0 |
| 12/12/2010 | 0 | 0 | 0 | 0 | -57.4 | 0 | 27 | 10 | 63 | 0 | 0 |
| 12/13/2010 | 0 | 0 | 0 | 0 | -60.9 | 0 | 15 | 25 | 60 | 0 | 0 |
| 12/14/2010 | 0 | 0 | 0 | 0 | -61.6 | 0 | 18 | 22 | 60 | 0 | 0 |
| 12/15/2010 | 0 | 0 | 0 | 0 | -59.6 | 0 | 16 | 21 | 63 | 0 | 0 |
| 12/16/2010 | 0 | 0 | 0 | 0 | -57.7 | 1 | 20 | 13 | 67 | 0 | 0 |
| 12/17/2010 | 0 | 0 | 0 | 0 | -61.2 | 3 | 15 | 26 | 56 | 0 | 0 |
| 12/18/2010 | 0 | 0 | 0 | 0 | -61.3 | 1 | 20 | 25 | 54 | 0 | 0 |
| 12/19/2010 | 0 | 0 | 0 | 0 | -62.0 | 0 | 13 | 20 | 67 | 0 | 0 |
| 12/20/2010 | 0 | 0 | 0 | 0 | -61.3 | 0 | 13 | 21 | 66 | 0 | 0 |
| 12/21/2010 | 0 | 0 | 0 | 0 | -58.8 | 1 | 14 | 14 | 71 | 0 | 0 |
| 12/22/2010 | 0 | 0 | 0 | 0 | -55.4 | 0 | 30 | 10 | 60 | 0 | 0 |
| 12/23/2010 | 0 | 0 | 0 | 0 | -61.7 | 0 | 30 | 22 | 47 | 0 | 0 |
| 12/24/2010 | 0 | 0 | 0 | 0 | -62.5 | 0 | 16 | 20 | 64 | 0 | 0 |
| 12/25/2010 | 0 | 0 | 0 | 0 | -60.2 | 0 | 19 | 19 | 61 | 1 | 0 |
| 12/26/2010 | 0 | 0 | 0 | 0 | -60.8 | 0 | 20 | 21 | 58 | 0 | 0 |
| 12/27/2010 | 0 | 0 | 0 | 0 | -63.9 | 0 | 18 | 29 | 53 | 0 | 0 |
| 12/28/2010 | 0 | 0 | 0 | 0 | -64.5 | 0 | 12 | 33 | 54 | 0 | 0 |
| 12/29/2010 | 0 | 0 | 0 | 0 | -63.9 | 0 | 10 | 35 | 55 | 0 | 0 |
| 12/30/2010 | 0 | 0 | 0 | 0 | -65.0 | 0 | 10 | 41 | 49 | 0 | 0 |
| 12/31/2010 | 0 | 0 | 0 | 0 | -63.7 | 0 | 14 | 33 | 53 | 0 | 0 |
| 1/1/2011 | 0 | 0 | 0 | 0 | -63.0 | 0 | 11 | 30 | 59 | 0 | 0 |
| 1/2/2011 | 0 | 0 | 0 | 0 | -64.6 | 0 | 13 | 40 | 47 | 0 | 0 |
| 1/3/2011 | 0 | 0 | 0 | 0 | -65.2 | 1 | 10 | 42 | 47 | 0 | 0 |
| 1/4/2011 | 0 | 0 | 0 | 0 | -48.9 | 8 | 10 | 29 | 53 | 0 | 0 |
| 1/5/2011 | 0 | 0 | 0 | 0 | -48.7 | 6 | 35 | 27 | 32 | 0 | 0 |
| 1/6/2011 | 0 | 0 | 0 | 0 | -64.8 | 0 | 14 | 38 | 48 | 0 | 0 |
| 1/7/2011 | 0 | 0 | 0 | 0 | -64.0 | 2 | 13 | 31 | 53 | 1 | 0 |
| 1/8/2011 | 0 | 0 | 0 | 0 | -62.3 | 0 | 19 | 26 | 55 | 0 | 0 |
| 1/9/2011 | 0 | 0 | 0 | 0 | -61.9 | 0 | 18 | 28 | 54 | 0 | 0 |
| 1/10/2011 | 0 | 0 | 0 | 0 | -62.7 | 0 | 13 | 30 | 57 | 0 | 1 |
| 1/11/2011 | 0 | 0 | 0 | 0 | -63.9 | 0 | 17 | 31 | 52 | 0 | 0 |
| 1/12/2011 | 0 | 0 | 0 | 0 | -62.1 | 0 | 17 | 28 | 54 | 0 | 1 |
| 1/13/2011 | 0 | 0 | 0 | 0 | -61.5 | 0 | 11 | 33 | 56 | 0 | 0 |
| 1/14/2011 | 0 | 0 | 0 | 0 | -63.1 | 0 | 16 | 31 | 53 | 0 | 0 |
| 1/15/2011 | 0 | 0 | 0 | 0 | -60.4 | 0 | 17 | 29 | 54 | 0 | 0 |
| 1/16/2011 | 0 | 0 | 0 | 0 | -58.4 | 0 | 26 | 24 | 49 | 0 | 0 |
| 1/17/2011 | 0 | 0 | 0 | 0 | -58.1 | 0 | 19 | 35 | 46 | 0 | 0 |
| 1/18/2011 | 0 | 0 | 0 | 0 | -56.5 | 6 | 14 | 19 | 59 | 2 | 0 |
| 1/19/2011 | 0 | 0 | 0 | 0 | -48.2 | 6 | 20 | 27 | 47 | 1 | 0 |
| 1/20/2011 | 0 | 0 | 0 | 0 | -62.4 | 0 | 20 | 38 | 42 | 0 | 0 |
| 1/21/2011 | 0 | 0 | 0 | 0 | -62.7 | 0 | 12 | 37 | 52 | 0 | 0 |
| 1/22/2011 | 0 | 0 | 0 | 0 | -60.1 | 1 | 13 | 30 | 53 | 2 | 1 |
| 1/23/2011 | 0 | 0 | 0 | 0 | -51.3 | 9 | 14 | 21 | 55 | 1 | 0 |
| 1/24/2011 | 0 | 0 | 0 | 0 | -59.1 | 0 | 33 | 31 | 36 | 1 | 0 |
| 1/25/2011 | 0 | 0 | 0 | 0 | -61.2 | 1 | 20 | 39 | 40 | 0 | 0 |
| 1/26/2011 | 0 | 0 | 0 | 0 | -62.0 | 0 | 26 | 38 | 35 | 1 | 0 |
| 1/27/2011 | 0 | 0 | 0 | 0 | -56.5 | 8 | 16 | 22 | 54 | 1 | 0 |
| 1/28/2011 | 0 | 0 | 0 | 0 | -53.9 | 2 | 22 | 30 | 45 | 1 | 0 |
| 1/29/2011 | 0 | 0 | 0 | 0 | -62.9 | 2 | 25 | 33 | 39 | 0 | 0 |
| 1/30/2011 | 0 | 0 | 0 | 0 | -63.9 | 0 | 23 | 37 | 40 | 0 | 0 |
| 1/31/2011 | 0 | 0 | 0 | 0 | -63.6 | 0 | 19 | 39 | 41 | 0 | 0 |
| 2/1/2011 | 0 | 0 | 0 | 0 | -56.1 | 1 | 18 | 27 | 51 | 3 | 0 |
| 2/2/2011 | 0 | 0 | 0 | 0 | -53.8 | 0 | 20 | 40 | 38 | 3 | 0 |
| 2/3/2011 | 0 | 0 | 0 | 0 | -55.5 | 0 | 14 | 47 | 37 | 2 | 0 |
| 2/4/2011 | 0 | 0 | 0 | 0 | -62.1 | 0 | 13 | 42 | 45 | 1 | 0 |
| 2/5/2011 | 0 | 0 | 0 | 0 | -61.0 | 6 | 7 | 40 | 46 | 0 | 0 |
| 2/6/2011 | 0 | 0 | 0 | 0 | -64.8 | 1 | 9 | 56 | 33 | 0 | 0 |
| 2/7/2011 | 0 | 0 | 0 | 0 | -64.1 | 0 | 17 | 39 | 44 | 0 | 0 |
| 2/8/2011 | 0 | 0 | 0 | 0 | -64.2 | 0 | 12 | 46 | 42 | 0 | 0 |
| 2/9/2011 | 0 | 0 | 0 | 0 | -61.9 | 3 | 8 | 44 | 46 | 0 | 0 |
| 2/10/2011 | 0 | 0 | 0 | 0 | -57.0 | 4 | 6 | 40 | 47 | 3 | 0 |
| 2/11/2011 | 0 | 0 | 0 | 0 | -57.4 | 0 | 10 | 52 | 33 | 5 | 0 |
| 2/12/2011 | 0 | 0 | 0 | 0 | -61.0 | 0 | 8 | 55 | 36 | 1 | 0 |
| 2/13/2011 | 0 | 0 | 0 | 0 | -61.6 | 6 | 6 | 39 | 47 | 2 | 0 |
| 2/14/2011 | 0 | 0 | 0 | 0 | -63.2 | 1 | 9 | 48 | 42 | 1 | 0 |
| 2/15/2011 | 0 | 0 | 0 | 0 | -64.0 | 0 | 7 | 54 | 38 | 1 | 0 |
| 2/16/2011 | 0 | 0 | 0 | 0 | -63.0 | 1 | 11 | 44 | 43 | 2 | 0 |
| 2/17/2011 | 0 | 0 | 0 | 0 | -63.9 | 1 | 14 | 40 | 45 | 0 | 0 |
| 2/18/2011 | 0 | 0 | 0 | 0 | -64.0 | 0 | 16 | 53 | 31 | 0 | 0 |
| 2/19/2011 | 0 | 0 | 0 | 0 | -63.7 | 1 | 12 | 51 | 36 | 1 | 0 |
| 2/20/2011 | 0 | 0 | 0 | 0 | -63.4 | 3 | 10 | 46 | 40 | 0 | 0 |
| 2/21/2011 | 0 | 0 | 0 | 0 | -64.4 | 0 | 20 | 49 | 31 | 0 | 0 |
| 2/22/2011 | 0 | 0 | 0 | 0 | -62.2 | 0 | 12 | 50 | 37 | 2 | 0 |
| 2/23/2011 | 0 | 0 | 0 | 0 | -61.0 | 1 | 13 | 41 | 43 | 2 | 0 |
| 2/24/2011 | 0 | 0 | 0 | 0 | -57.3 | 8 | 17 | 31 | 43 | 1 | 0 |
| 2/25/2011 | 0 | 0 | 0 | 0 | -61.5 | 0 | 11 | 45 | 44 | 0 | 0 |
| 2/26/2011 | 0 | 0 | 0 | 0 | -65.3 | 1 | 17 | 43 | 39 | 0 | 0 |
| 2/27/2011 | 0 | 0 | 0 | 0 | -63.1 | 0 | 7 | 52 | 41 | 1 | 0 |
| 2/28/2011 | 0 | 0 | 0 | 0 | -60.8 | 0 | 9 | 55 | 33 | 2 | 0 |
| 3/1/2011 | 0 | 0 | 0 | 0 | -62.1 | 0 | 11 | 59 | 30 | 0 | 0 |
| 3/2/2011 | 0 | 0 | 0 | 0 | -61.3 | 0 | 5 | 49 | 44 | 2 | 0 |
| 3/3/2011 | 0 | 0 | 0 | 0 | -61.0 | 0 | 6 | 47 | 45 | 1 | 1 |
| 3/4/2011 | 0 | 0 | 0 | 0 | -64.1 | 0 | 2 | 54 | 43 | 1 | 0 |
| 3/5/2011 | 0 | 0 | 0 | 0 | -64.0 | 0 | 10 | 56 | 34 | 1 | 0 |
| 3/6/2011 | 0 | 0 | 0 | 0 | -64.5 | 0 | 3 | 58 | 38 | 1 | 0 |
| 3/7/2011 | 0 | 0 | 0 | 0 | -66.6 | 0 | 1 | 61 | 37 | 0 | 0 |
| 3/8/2011 | 0 | 0 | 0 | 0 | -65.5 | 0 | 13 | 65 | 21 | 1 | 0 |
| 3/9/2011 | 1 | 0 | 0 | 0 | -65.4 | 0 | 9 | 66 | 24 | 0 | 0 |
| 3/10/2011 | 0 | 0 | 0 | 0 | -66.6 | 0 | 11 | 69 | 20 | 0 | 0 |
| 3/11/2011 | 1 | 0 | 0 | 0 | -66.0 | 0 | 11 | 71 | 18 | 0 | 0 |
| 3/12/2011 | 0 | 0 | 0 | 0 | -65.9 | 0 | 3 | 66 | 30 | 0 | 0 |
| 3/13/2011 | 0 | 0 | 0 | 0 | -65.5 | 0 | 11 | 66 | 23 | 0 | 0 |
| 3/14/2011 | 0 | 0 | 0 | 0 | -65.4 | 0 | 10 | 68 | 22 | 0 | 0 |
| 3/15/2011 | 0 | 0 | 0 | 0 | -64.9 | 0 | 8 | 70 | 22 | 0 | 0 |
| 3/16/2011 | 1 | 0 | 0 | 0 | -65.6 | 0 | 13 | 69 | 19 | 0 | 0 |
| 3/17/2011 | 0 | 0 | NA | NA | -67.4 | 0 | 10 | 74 | 16 | 0 | 0 |
| 3/18/2011 | 0 | 0 | 60 | 15 | -64.6 | 0 | 4 | 66 | 30 | 0 | 0 |
| 3/19/2011 | 1 | 0 | NA | NA | -63.1 | 0 | 8 | 58 | 33 | 1 | 0 |
| 3/20/2011 | 0 | 0 | NA | NA | -56.0 | 0 | 11 | 46 | 41 | 1 | 0 |
| 3/21/2011 | 0 | 0 | 0 | 0 | -64.1 | 0 | 14 | 47 | 38 | 1 | 0 |
| 3/22/2011 | 0 | 0 | NA | NA | -64.6 | 0 | 15 | 50 | 35 | 0 | 0 |
| 3/23/2011 | 0 | 0 | 0 | 0 | -64.7 | 0 | 18 | 55 | 24 | 2 | 0 |
| 3/24/2011 | 0 | 0 | NA | NA | -63.0 | 0 | 13 | 59 | 27 | 1 | 0 |
| 3/25/2011 | 0 | 0 | 0 | 0 | -64.5 | 0 | 14 | 60 | 25 | 0 | 0 |
| 3/26/2011 | 0 | 0 | NA | NA | -64.1 | 0 | 15 | 51 | 34 | 0 | 0 |
| 3/27/2011 | 0 | 0 | NA | NA | -64.1 | 0 | 7 | 61 | 32 | 0 | 0 |
| 3/28/2011 | 0 | 0 | 0 | 0 | -64.4 | 0 | 4 | 69 | 27 | 1 | 0 |
| 3/29/2011 | 0 | 0 | NA | NA | -62.3 | 0 | 8 | 56 | 34 | 2 | 0 |
| 3/30/2011 | 0 | 0 | NA | NA | -60.7 | 0 | 9 | 47 | 42 | 2 | 0 |
| 3/31/2011 | 0 | 0 | 0 | 0 | -60.9 | 0 | 7 | 56 | 37 | 0 | 0 |
| 4/1/2011 | 0 | 0 | 60 | 36 | -63.0 | 0 | 7 | 64 | 27 | 1 | 0 |
| 4/2/2011 | 0 | 0 | NA | NA | -63.0 | 0 | 11 | 60 | 29 | 0 | 0 |
| 4/3/2011 | 0 | 0 | NA | NA | -61.8 | 0 | 17 | 47 | 34 | 1 | 0 |
| 4/4/2011 | 0 | 0 | 0 | 0 | -64.2 | 0 | 6 | 57 | 36 | 1 | 0 |
| 4/5/2011 | 0 | 0 | NA | NA | -63.1 | 0 | 13 | 47 | 40 | 1 | 0 |
| 4/6/2011 | 0 | 0 | 0 | 0 | -63.2 | 0 | 14 | 44 | 41 | 0 | 0 |
| 4/7/2011 | 0 | 0 | NA | NA | -58.0 | 7 | 61 | 7 | 24 | 1 | 0 |
| 4/8/2011 | 0 | 0 | 0 | 0 | -60.3 | 11 | 77 | 1 | 11 | 0 | 0 |
| 4/9/2011 | 0 | 0 | NA | NA | -59.1 | 8 | 75 | 3 | 14 | 0 | 0 |
| 4/10/2011 | 0 | 0 | NA | NA | -64.6 | 0 | 42 | 30 | 28 | 0 | 0 |
| 4/11/2011 | 0 | 0 | 0 | 0 | -64.3 | 0 | 13 | 42 | 44 | 0 | 0 |
| 4/12/2011 | 0 | 0 | NA | NA | -63.7 | 0 | 9 | 58 | 32 | 0 | 1 |
| 4/13/2011 | 0 | 0 | 0 | 0 | -64.6 | 0 | 26 | 47 | 26 | 1 | 0 |
| 4/14/2011 | 0 | 0 | NA | NA | -63.1 | 0 | 14 | 45 | 41 | 0 | 0 |
| 4/15/2011 | 0 | 1 | 40 | 36 | -64.3 | 0 | 15 | 41 | 44 | 0 | 0 |
| 4/16/2011 | 0 | 1 | NA | NA | -65.2 | 0 | 18 | 37 | 45 | 0 | 0 |
| 4/17/2011 | 0 | 1 | NA | NA | -64.8 | 0 | 14 | 41 | 44 | 0 | 1 |
| 4/18/2011 | 0 | 0 | 60 | 36 | -64.4 | 0 | 18 | 39 | 42 | 1 | 0 |
| 4/19/2011 | 0 | 1 | NA | NA | -65.3 | 0 | 27 | 41 | 32 | 0 | 0 |
| 4/20/2011 | 0 | 1 | 0 | 0 | -66.2 | 0 | 24 | 53 | 23 | 0 | 0 |
| 4/21/2011 | 0 | 1 | NA | NA | -65.9 | 0 | 10 | 57 | 33 | 0 | 0 |
| 4/22/2011 | 0 | 1 | 0 | 0 | -65.6 | 0 | 7 | 58 | 35 | 0 | 0 |
| 4/23/2011 | 0 | 0 | NA | NA | -64.0 | 0 | 8 | 54 | 38 | 0 | 0 |
| 4/24/2011 | 0 | 0 | NA | NA | -61.1 | 0 | 15 | 46 | 38 | 1 | 0 |
| 4/25/2011 | 0 | 1 | 30 | 36 | -64.4 | 0 | 26 | 51 | 23 | 0 | 0 |
| 4/26/2011 | 0 | 0 | NA | NA | -66.3 | 0 | 9 | 64 | 27 | 0 | 0 |
| 4/27/2011 | 0 | 1 | 0 | 0 | -65.1 | 0 | 11 | 56 | 32 | 1 | 0 |
| 4/28/2011 | 0 | 0 | NA | NA | -66.8 | 0 | 9 | 65 | 26 | 0 | 0 |
| 4/29/2011 | 0 | 0 | 40 | 30 | -67.4 | 0 | 13 | 58 | 28 | 0 | 0 |
| 4/30/2011 | 0 | 0 | NA | NA | -67.5 | 0 | 11 | 62 | 27 | 0 | 0 |
| 5/1/2011 | 0 | 0 | NA | NA | -66.8 | 0 | 11 | 59 | 30 | 1 | 0 |
| 5/2/2011 | 0 | 0 | 0 | 0 | -68.6 | 0 | 4 | 72 | 24 | 0 | 0 |
| 5/3/2011 | 0 | 0 | NA | NA | -68.8 | 0 | 7 | 66 | 28 | 0 | 0 |
| 5/4/2011 | 0 | 0 | 0 | 0 | -67.5 | 0 | 14 | 61 | 26 | 0 | 0 |
| 5/5/2011 | 0 | 0 | 0 | 0 | -64.4 | 0 | 15 | 53 | 31 | 0 | 0 |
| 5/6/2011 | 0 | 0 | 0 | 0 | -64.1 | 0 | 16 | 50 | 34 | 1 | 0 |
| 5/7/2011 | 0 | 0 | 0 | 0 | -61.6 | 0 | 20 | 51 | 27 | 1 | 0 |
| 5/8/2011 | 0 | 0 | 0 | 0 | -62.0 | 0 | 21 | 50 | 29 | 1 | 0 |
| 5/9/2011 | 0 | 0 | 0 | 0 | -63.9 | 0 | 16 | 58 | 26 | 0 | 0 |
| 5/10/2011 | 0 | 0 | 0 | 0 | -60.2 | 0 | 12 | 49 | 38 | 0 | 0 |
| 5/11/2011 | 0 | 0 | 0 | 0 | -60.9 | 0 | 14 | 48 | 36 | 2 | 0 |
| 5/12/2011 | 0 | 0 | 0 | 0 | -53.7 | 0 | 8 | 44 | 46 | 2 | 0 |
| 5/13/2011 | 0 | 0 | 0 | 0 | -56.4 | 0 | 9 | 41 | 47 | 3 | 0 |
| 5/14/2011 | 0 | 0 | 0 | 0 | -56.4 | 0 | 14 | 32 | 51 | 3 | 0 |
| 5/15/2011 | 0 | 0 | 0 | 0 | -58.3 | 0 | 17 | 31 | 50 | 2 | 0 |
| 5/16/2011 | 0 | 0 | 0 | 0 | -58.5 | 0 | 19 | 31 | 49 | 1 | 0 |
| 5/17/2011 | 0 | 0 | 0 | 0 | -58.3 | 0 | 24 | 34 | 40 | 1 | 0 |
| 5/18/2011 | 0 | 0 | 0 | 0 | -57.3 | 0 | 17 | 32 | 35 | 2 | 0 |
| 5/19/2011 | 0 | 0 | 0 | 0 | NA | NA | NA | NA | NA | NA | NA |

B)

| **Date** | **Bearded seal presence** | **Ribbon seal presence** | **Ice cover (%)** | **Ice thickness (cm)** | **200 kHz Sv (dB)** | **Small scatter % comp** | **Medium scatter % comp** | **Large scatter % comp** | **Weak resonator % comp** | **Strong resonator % comp** | **Unclass % comp** |
| --- | --- | --- | --- | --- | --- | --- | --- | --- | --- | --- | --- |
| 9/26/2008 | NA | NA | 0 | 0 | -68.7 | 0 | 14 | 68 | 17 | 0 | 0 |
| 9/27/2008 | 0 | 0 | 0 | 0 | -68.4 | 0 | 17 | 65 | 18 | 0 | 0 |
| 9/28/2008 | 0 | 0 | 0 | 0 | -67.8 | 0 | 10 | 54 | 30 | 0 | 0 |
| 9/29/2008 | 0 | 0 | 0 | 0 | -68.8 | 0 | 16 | 61 | 21 | 0 | 0 |
| 9/30/2008 | 0 | 0 | 0 | 0 | -68.3 | 0 | 19 | 57 | 23 | 0 | 0 |
| 10/1/2008 | 0 | 0 | 0 | 0 | -68.0 | 0 | 15 | 52 | 27 | 0 | 0 |
| 10/2/2008 | 0 | 0 | 0 | 0 | -67.9 | 0 | 12 | 47 | 35 | 0 | 1 |
| 10/3/2008 | 0 | 0 | 0 | 0 | -68.1 | 0 | 12 | 41 | 35 | 0 | 0 |
| 10/4/2008 | 0 | 0 | 0 | 0 | -64.5 | 0 | 10 | 39 | 42 | 0 | 0 |
| 10/5/2008 | 0 | 0 | 0 | 0 | -68.4 | 0 | 12 | 37 | 41 | 0 | 0 |
| 10/6/2008 | 0 | 0 | 0 | 0 | -68.5 | 0 | 14 | 55 | 23 | 0 | 0 |
| 10/7/2008 | 0 | 0 | 0 | 0 | -68.7 | 0 | 15 | 59 | 20 | 0 | 0 |
| 10/8/2008 | 0 | 0 | 0 | 0 | -68.3 | 0 | 18 | 58 | 22 | 0 | 0 |
| 10/9/2008 | 0 | 0 | 0 | 0 | -65.5 | 0 | 11 | 29 | 34 | 0 | 0 |
| 10/10/2008 | 0 | 0 | 0 | 0 | -64.8 | 0 | 12 | 27 | 40 | 0 | 0 |
| 10/11/2008 | 0 | 0 | 0 | 0 | -68.4 | 0 | 11 | 41 | 38 | 0 | 0 |
| 10/12/2008 | 0 | 0 | 0 | 0 | -68.8 | 0 | 14 | 53 | 32 | 0 | 0 |
| 10/13/2008 | 0 | 0 | 0 | 0 | -67.9 | 0 | 13 | 38 | 33 | 0 | 0 |
| 10/14/2008 | 0 | 0 | 0 | 0 | -69.0 | 0 | 12 | 43 | 37 | 0 | 0 |
| 10/15/2008 | 0 | 0 | 0 | 0 | -64.7 | 2 | 12 | 43 | 37 | 0 | 0 |
| 10/16/2008 | 0 | 0 | 0 | 0 | -67.1 | 0 | 31 | 42 | 28 | 0 | 0 |
| 10/17/2008 | 0 | 0 | 0 | 0 | -67.3 | 0 | 13 | 47 | 35 | 0 | 0 |
| 10/18/2008 | 0 | 0 | 0 | 0 | -69.4 | 0 | 11 | 49 | 31 | 0 | 0 |
| 10/19/2008 | 0 | 0 | 0 | 0 | -68.1 | 0 | 9 | 51 | 31 | 0 | 0 |
| 10/20/2008 | 0 | 0 | 0 | 0 | -68.6 | 0 | 16 | 60 | 23 | 0 | 0 |
| 10/21/2008 | 0 | 0 | 0 | 0 | -68.1 | 0 | 11 | 54 | 30 | 0 | 0 |
| 10/22/2008 | 0 | 0 | 0 | 0 | -68.0 | 0 | 14 | 49 | 32 | 0 | 0 |
| 10/23/2008 | 0 | 0 | 0 | 0 | -68.4 | 0 | 10 | 49 | 24 | 0 | 0 |
| 10/24/2008 | 0 | 0 | 0 | 0 | -68.4 | 0 | 13 | 54 | 28 | 0 | 0 |
| 10/25/2008 | 0 | 0 | 0 | 0 | -67.8 | 0 | 16 | 54 | 28 | 0 | 0 |
| 10/26/2008 | 0 | 0 | 0 | 0 | -66.4 | 1 | 20 | 53 | 27 | 0 | 0 |
| 10/27/2008 | 0 | 0 | 0 | 0 | -64.3 | 0 | 15 | 49 | 28 | 0 | 0 |
| 10/28/2008 | 0 | 0 | 0 | 0 | -66.0 | 0 | 19 | 46 | 22 | 0 | 0 |
| 10/29/2008 | 0 | 0 | 0 | 0 | -69.2 | 0 | 14 | 45 | 33 | 0 | 0 |
| 10/30/2008 | 0 | 0 | 0 | 0 | -68.5 | 0 | 11 | 46 | 35 | 0 | 0 |
| 10/31/2008 | 0 | 0 | 0 | 0 | -63.4 | 0 | 11 | 45 | 32 | 0 | 0 |
| 11/1/2008 | 0 | 0 | 0 | 0 | -64.3 | 0 | 12 | 46 | 36 | 0 | 0 |
| 11/2/2008 | 0 | 0 | 0 | 0 | -63.8 | 0 | 16 | 47 | 34 | 0 | 0 |
| 11/3/2008 | 0 | 0 | 0 | 0 | -63.1 | 0 | 14 | 42 | 40 | 0 | 0 |
| 11/4/2008 | 0 | 0 | 0 | 0 | -63.9 | 2 | 8 | 40 | 40 | 0 | 0 |
| 11/5/2008 | 0 | 0 | 0 | 0 | -61.4 | 0 | 12 | 38 | 40 | 0 | 0 |
| 11/6/2008 | 0 | 0 | 0 | 0 | -61.5 | 0 | 11 | 40 | 34 | 0 | 0 |
| 11/7/2008 | 0 | 0 | 0 | 0 | -59.8 | 0 | 10 | 44 | 40 | 0 | 0 |
| 11/8/2008 | 0 | 0 | 0 | 0 | -62.2 | 0 | 10 | 44 | 32 | 0 | 0 |
| 11/9/2008 | 0 | 0 | 0 | 0 | -53.3 | 0 | 9 | 47 | 32 | 2 | 1 |
| 11/10/2008 | 0 | 0 | 0 | 0 | -55.9 | 0 | 9 | 45 | 36 | 0 | 1 |
| 11/11/2008 | 0 | 0 | 0 | 0 | -60.0 | 0 | 11 | 44 | 37 | 0 | 0 |
| 11/12/2008 | 0 | 0 | 0 | 0 | -60.3 | 0 | 9 | 42 | 37 | 0 | 0 |
| 11/13/2008 | 0 | 0 | 0 | 0 | -61.8 | 0 | 15 | 43 | 37 | 0 | 0 |
| 11/14/2008 | 0 | 0 | 0 | 0 | -62.0 | 0 | 16 | 43 | 41 | 0 | 0 |
| 11/15/2008 | 0 | 0 | 0 | 0 | -58.0 | 0 | 15 | 34 | 45 | 0 | 0 |
| 11/16/2008 | 0 | 0 | 0 | 0 | -63.9 | 0 | 15 | 31 | 40 | 1 | 0 |
| 11/17/2008 | 0 | 0 | 0 | 0 | -61.7 | 0 | 11 | 31 | 44 | 0 | 0 |
| 11/18/2008 | 0 | 0 | 0 | 0 | -59.9 | 0 | 10 | 40 | 44 | 0 | 0 |
| 11/19/2008 | 0 | 0 | 0 | 0 | -60.8 | 0 | 15 | 40 | 45 | 0 | 0 |
| 11/20/2008 | 0 | 0 | 0 | 0 | -59.9 | 1 | 11 | 39 | 38 | 1 | 0 |
| 11/21/2008 | 0 | 0 | 0 | 0 | -61.4 | 0 | 18 | 24 | 36 | 0 | 0 |
| 11/22/2008 | 0 | 0 | 0 | 0 | -59.6 | 1 | 51 | 15 | 21 | 0 | 0 |
| 11/23/2008 | 0 | 0 | 0 | 0 | -62.0 | 0 | 34 | 23 | 36 | 0 | 0 |
| 11/24/2008 | 0 | 0 | 0 | 0 | -62.5 | 0 | 20 | 34 | 33 | 0 | 0 |
| 11/25/2008 | 0 | 0 | 0 | 0 | -64.3 | 0 | 12 | 37 | 43 | 0 | 0 |
| 11/26/2008 | 0 | 0 | 0 | 0 | -64.5 | 0 | 12 | 35 | 40 | 0 | 0 |
| 11/27/2008 | 0 | 0 | 0 | 0 | -65.7 | 0 | 10 | 41 | 41 | 0 | 0 |
| 11/28/2008 | 0 | 0 | 0 | 0 | -62.3 | 0 | 12 | 42 | 34 | 0 | 0 |
| 11/29/2008 | 0 | 0 | 0 | 0 | -63.2 | 0 | 10 | 48 | 37 | 0 | 0 |
| 11/30/2008 | 0 | 0 | 0 | 0 | -61.8 | 0 | 13 | 42 | 41 | 0 | 0 |
| 12/1/2008 | 0 | 0 | 0 | 0 | -63.2 | 0 | 13 | 37 | 37 | 0 | 0 |
| 12/2/2008 | 0 | 0 | 0 | 0 | -65.1 | 0 | 11 | 36 | 31 | 0 | 0 |
| 12/3/2008 | 0 | 0 | 0 | 0 | -65.1 | 0 | 15 | 31 | 31 | 0 | 0 |
| 12/4/2008 | 0 | 0 | 0 | 0 | -64.5 | 0 | 30 | 17 | 22 | 1 | 0 |
| 12/5/2008 | 0 | 0 | 0 | 0 | -66.5 | 8 | 56 | 13 | 6 | 0 | 0 |
| 12/6/2008 | 0 | 0 | 0 | 0 | -63.3 | 1 | 50 | 16 | 17 | 0 | 0 |
| 12/7/2008 | 0 | 0 | 0 | 0 | -59.0 | 3 | 16 | 34 | 44 | 0 | 0 |
| 12/8/2008 | 0 | 0 | 0 | 0 | -61.8 | 1 | 14 | 35 | 45 | 0 | 0 |
| 12/9/2008 | 0 | 0 | 0 | 0 | -68.2 | 0 | 10 | 42 | 39 | 0 | 0 |
| 12/10/2008 | 0 | 0 | 0 | 0 | -67.8 | 0 | 10 | 49 | 26 | 0 | 0 |
| 12/11/2008 | 0 | 0 | 0 | 0 | -67.0 | 0 | 12 | 48 | 32 | 0 | 0 |
| 12/12/2008 | 0 | 0 | 0 | 0 | -69.0 | 0 | 16 | 41 | 33 | 0 | 0 |
| 12/13/2008 | 0 | 0 | 0 | 0 | -66.2 | 3 | 41 | 28 | 18 | 0 | 0 |
| 12/14/2008 | 0 | 0 | 0 | 0 | -68.0 | 0 | 35 | 34 | 21 | 0 | 0 |
| 12/15/2008 | 0 | 0 | 0 | 0 | -69.5 | 0 | 16 | 37 | 39 | 0 | 0 |
| 12/16/2008 | 0 | 0 | 0 | 0 | -67.4 | 0 | 20 | 33 | 36 | 0 | 0 |
| 12/17/2008 | 0 | 0 | 0 | 0 | -67.5 | 0 | 20 | 30 | 39 | 0 | 0 |
| 12/18/2008 | 0 | 0 | 0 | 0 | -66.3 | 0 | 26 | 28 | 42 | 0 | 0 |
| 12/19/2008 | 0 | 0 | 0 | 0 | -66.3 | 0 | 18 | 30 | 46 | 0 | 0 |
| 12/20/2008 | 0 | 0 | 0 | 0 | -67.1 | 0 | 21 | 38 | 41 | 0 | 0 |
| 12/21/2008 | 0 | 0 | 0 | 0 | -68.7 | 0 | 12 | 45 | 38 | 0 | 0 |
| 12/22/2008 | 0 | 0 | 0 | 0 | -68.2 | 0 | 13 | 42 | 38 | 0 | 0 |
| 12/23/2008 | 0 | 0 | 0 | 0 | -67.3 | 0 | 16 | 30 | 34 | 1 | 1 |
| 12/24/2008 | 0 | 0 | 0 | 0 | -66.9 | 0 | 29 | 25 | 34 | 0 | 0 |
| 12/25/2008 | 0 | 0 | 0 | 0 | -66.0 | 1 | 33 | 21 | 30 | 0 | 0 |
| 12/26/2008 | 0 | 0 | 0 | 0 | -67.2 | 0 | 29 | 40 | 21 | 0 | 0 |
| 12/27/2008 | 0 | 0 | 0 | 0 | -69.2 | 0 | 12 | 59 | 15 | 0 | 0 |
| 12/28/2008 | 0 | 0 | 0 | 0 | -70.4 | 0 | 10 | 64 | 17 | 0 | 0 |
| 12/29/2008 | 0 | 0 | 0 | 0 | -68.8 | 0 | 11 | 42 | 36 | 0 | 0 |
| 12/30/2008 | 0 | 0 | 0 | 0 | -67.8 | 0 | 13 | 33 | 48 | 0 | 0 |
| 12/31/2008 | 0 | 0 | 0 | 0 | -68.9 | 0 | 10 | 38 | 48 | 0 | 0 |
| 1/1/2009 | 0 | 0 | NA | NA | -68.4 | 0 | 10 | 47 | 42 | 0 | 0 |
| 1/2/2009 | 0 | 0 | 40 | 5 | -68.8 | 0 | 10 | 47 | 34 | 0 | 0 |
| 1/3/2009 | 0 | 0 | NA | NA | -67.7 | 0 | 10 | 40 | 42 | 0 | 0 |
| 1/4/2009 | 0 | 0 | NA | NA | -68.7 | 0 | 11 | 42 | 43 | 0 | 0 |
| 1/5/2009 | 0 | 0 | 50 | 20 | -68.9 | 0 | 12 | 45 | 42 | 0 | 0 |
| 1/6/2009 | 0 | 0 | NA | NA | -70.5 | 0 | 9 | 64 | 28 | 0 | 0 |
| 1/7/2009 | 1 | 0 | 70 | 50 | -70.5 | 0 | 9 | 69 | 22 | 0 | 0 |
| 1/8/2009 | 0 | 0 | NA | NA | -70.9 | 0 | 9 | 75 | 14 | 0 | 0 |
| 1/9/2009 | 0 | 0 | 70 | 50 | -71.1 | 0 | 10 | 83 | 7 | 0 | 0 |
| 1/10/2009 | 0 | 0 | NA | NA | -71.1 | 0 | 11 | 72 | 15 | 0 | 0 |
| 1/11/2009 | 0 | 0 | NA | NA | -71.2 | 0 | 9 | 75 | 16 | 0 | 0 |
| 1/12/2009 | 0 | 0 | 70 | 50 | -71.4 | 0 | 8 | 83 | 5 | 0 | 0 |
| 1/13/2009 | 0 | 0 | NA | NA | -71.5 | 0 | 9 | 84 | 7 | 0 | 0 |
| 1/14/2009 | 0 | 0 | 70 | 50 | -70.9 | 0 | 12 | 81 | 6 | 0 | 0 |
| 1/15/2009 | 0 | 0 | NA | NA | -71.2 | 0 | 10 | 84 | 5 | 0 | 0 |
| 1/16/2009 | 1 | 0 | 80 | 50 | -70.9 | 0 | 11 | 81 | 4 | 0 | 0 |
| 1/17/2009 | 0 | 0 | NA | NA | -70.4 | 0 | 18 | 76 | 5 | 0 | 0 |
| 1/18/2009 | 0 | 0 | NA | NA | -71.1 | 0 | 11 | 85 | 4 | 0 | 1 |
| 1/19/2009 | 1 | 0 | 70 | 20 | -71.6 | 0 | 10 | 88 | 2 | 0 | 0 |
| 1/20/2009 | 0 | 0 | NA | NA | -71.5 | 0 | 9 | 87 | 1 | 0 | 0 |
| 1/21/2009 | 0 | 0 | 70 | 20 | -71.3 | 0 | 10 | 88 | 2 | 0 | 0 |
| 1/22/2009 | 0 | 0 | NA | NA | -71.0 | 0 | 11 | 86 | 4 | 0 | 0 |
| 1/23/2009 | 0 | 0 | 70 | 20 | -70.6 | 0 | 10 | 83 | 4 | 0 | 0 |
| 1/24/2009 | 0 | 0 | NA | NA | -69.8 | 0 | 11 | 81 | 6 | 0 | 0 |
| 1/25/2009 | 0 | 0 | NA | NA | -71.5 | 0 | 9 | 90 | 1 | 0 | 0 |
| 1/26/2009 | 0 | 0 | 75 | 50 | -71.1 | 0 | 9 | 83 | 7 | 0 | 0 |
| 1/27/2009 | 0 | 0 | NA | NA | -69.5 | 0 | 10 | 81 | 9 | 0 | 0 |
| 1/28/2009 | 0 | 0 | 75 | 50 | -70.7 | 0 | 10 | 80 | 7 | 0 | 1 |
| 1/29/2009 | 0 | 0 | NA | NA | -70.6 | 0 | 10 | 83 | 5 | 0 | 0 |
| 1/30/2009 | 1 | 1 | 80 | 50 | -70.3 | 0 | 10 | 78 | 11 | 0 | 0 |
| 1/31/2009 | 1 | 0 | NA | NA | -70.5 | 0 | 12 | 77 | 9 | 0 | 0 |
| 2/1/2009 | 0 | 0 | NA | NA | -69.9 | 0 | 10 | 75 | 10 | 0 | 0 |
| 2/2/2009 | 0 | 1 | 80 | 50 | -70.2 | 0 | 11 | 68 | 16 | 0 | 0 |
| 2/3/2009 | 1 | 0 | NA | NA | -71.0 | 0 | 10 | 82 | 6 | 0 | 0 |
| 2/4/2009 | 0 | 0 | 80 | 50 | -69.2 | 0 | 16 | 75 | 3 | 0 | 0 |
| 2/5/2009 | 1 | 0 | NA | NA | -70.4 | 0 | 11 | 79 | 4 | 0 | 0 |
| 2/6/2009 | 1 | 0 | 80 | 50 | -70.5 | 0 | 14 | 73 | 10 | 0 | 0 |
| 2/7/2009 | 1 | 1 | NA | NA | -68.7 | 0 | 16 | 67 | 10 | 0 | 1 |
| 2/8/2009 | 1 | 0 | NA | NA | -70.0 | 0 | 11 | 63 | 17 | 0 | 1 |
| 2/9/2009 | 1 | 1 | 80 | 50 | -70.1 | 0 | 10 | 64 | 18 | 0 | 0 |
| 2/10/2009 | 0 | 0 | NA | NA | -70.9 | 0 | 11 | 69 | 13 | 0 | 0 |
| 2/11/2009 | 0 | 0 | 80 | 50 | -69.5 | 0 | 10 | 71 | 10 | 0 | 0 |
| 2/12/2009 | 1 | 0 | 80 | 50 | -70.1 | 0 | 14 | 78 | 6 | 0 | 0 |
| 2/13/2009 | 0 | 1 | 80 | 50 | -65.5 | 0 | 13 | 72 | 11 | 0 | 1 |
| 2/14/2009 | 1 | 0 | NA | NA | -70.5 | 0 | 10 | 79 | 6 | 0 | 0 |
| 2/15/2009 | 1 | 1 | NA | NA | -70.1 | 0 | 10 | 78 | 7 | 0 | 0 |
| 2/16/2009 | 1 | 1 | 80 | 50 | -67.1 | 1 | 11 | 65 | 15 | 0 | 0 |
| 2/17/2009 | 1 | 1 | NA | NA | -68.6 | 0 | 13 | 74 | 10 | 0 | 0 |
| 2/18/2009 | 1 | 1 | 80 | 50 | -69.7 | 0 | 11 | 73 | 6 | 0 | 1 |
| 2/19/2009 | 1 | 1 | NA | NA | -68.2 | 1 | 10 | 77 | 5 | 0 | 0 |
| 2/20/2009 | 1 | 1 | 80 | 50 | -69.0 | 0 | 10 | 74 | 6 | 0 | 1 |
| 2/21/2009 | 1 | 1 | NA | NA | -69.7 | 0 | 13 | 79 | 3 | 0 | 0 |
| 2/22/2009 | 1 | 1 | NA | NA | -69.8 | 0 | 12 | 78 | 6 | 0 | 0 |
| 2/23/2009 | 0 | 1 | 80 | 50 | -69.6 | 0 | 13 | 72 | 10 | 0 | 0 |
| 2/24/2009 | 1 | 1 | NA | NA | -71.1 | 0 | 12 | 72 | 8 | 0 | 0 |
| 2/25/2009 | 0 | 0 | 80 | 50 | -69.3 | 0 | 17 | 73 | 5 | 0 | 0 |
| 2/26/2009 | 0 | 0 | NA | NA | -71.9 | 0 | 13 | 72 | 6 | 0 | 0 |
| 2/27/2009 | 0 | 1 | 80 | 50 | -69.2 | 0 | 26 | 62 | 10 | 0 | 0 |
| 2/28/2009 | 1 | 0 | NA | NA | -66.2 | 2 | 50 | 36 | 4 | 0 | 0 |
| 3/1/2009 | 0 | 1 | NA | NA | -68.0 | 0 | 26 | 65 | 8 | 0 | 0 |
| 3/2/2009 | 1 | 1 | 80 | 20 | -67.5 | 0 | 25 | 59 | 11 | 1 | 0 |
| 3/3/2009 | 1 | 0 | NA | NA | -67.9 | 0 | 19 | 63 | 16 | 0 | 0 |
| 3/4/2009 | 0 | 0 | 60 | 20 | -68.2 | 0 | 17 | 63 | 20 | 0 | 0 |
| 3/5/2009 | 0 | 0 | NA | NA | -66.5 | 8 | 32 | 33 | 10 | 0 | 1 |
| 3/6/2009 | 0 | 0 | 0 | 0 | -67.3 | 8 | 76 | 13 | 0 | 0 | 0 |
| 3/7/2009 | 0 | 0 | NA | NA | -67.7 | 0 | 20 | 60 | 14 | 0 | 0 |
| 3/8/2009 | 0 | 0 | NA | NA | -66.1 | 5 | 43 | 26 | 18 | 0 | 0 |
| 3/9/2009 | 0 | 0 | 0 | 0 | -68.2 | 0 | 21 | 58 | 18 | 0 | 0 |
| 3/10/2009 | 0 | 0 | 0 | 0 | -68.2 | 0 | 21 | 54 | 23 | 0 | 0 |
| 3/11/2009 | 0 | 0 | 0 | 0 | -69.1 | 0 | 20 | 58 | 17 | 0 | 0 |
| 3/12/2009 | 0 | 0 | NA | NA | -69.9 | 0 | 12 | 67 | 22 | 0 | 0 |
| 3/13/2009 | 0 | 0 | 0 | 0 | -69.6 | 0 | 15 | 62 | 23 | 0 | 0 |
| 3/14/2009 | 0 | 0 | NA | NA | -68.6 | 1 | 30 | 55 | 13 | 0 | 0 |
| 3/15/2009 | 0 | 0 | NA | NA | -69.6 | 0 | 22 | 62 | 16 | 0 | 0 |
| 3/16/2009 | 0 | 0 | 20 | 5 | -69.1 | 0 | 19 | 60 | 21 | 0 | 0 |
| 3/17/2009 | 1 | 1 | 60 | 20 | -69.8 | 0 | 14 | 58 | 28 | 0 | 0 |
| 3/18/2009 | 1 | 0 | 70 | 5 | -69.6 | 0 | 14 | 56 | 28 | 0 | 0 |
| 3/19/2009 | 1 | 0 | NA | NA | -68.0 | 0 | 16 | 52 | 26 | 0 | 0 |
| 3/20/2009 | 1 | 0 | 75 | 5 | -69.5 | 0 | 12 | 60 | 24 | 0 | 0 |
| 3/21/2009 | 1 | 0 | NA | NA | -70.3 | 0 | 10 | 72 | 12 | 0 | 0 |
| 3/22/2009 | 1 | 0 | NA | NA | -70.5 | 0 | 11 | 75 | 10 | 0 | 0 |
| 3/23/2009 | 1 | 0 | 85 | 50 | -69.9 | 0 | 14 | 67 | 15 | 0 | 1 |
| 3/24/2009 | 1 | 0 | NA | NA | -69.0 | 0 | 15 | 66 | 12 | 0 | 0 |
| 3/25/2009 | 1 | 0 | 75 | 50 | -70.0 | 0 | 14 | 73 | 10 | 0 | 0 |
| 3/26/2009 | 1 | 0 | NA | NA | -69.9 | 0 | 15 | 74 | 7 | 0 | 0 |
| 3/27/2009 | 1 | 0 | 75 | 50 | -70.0 | 0 | 13 | 63 | 20 | 0 | 1 |
| 3/28/2009 | 1 | 0 | NA | NA | -68.4 | 0 | 10 | 70 | 16 | 0 | 0 |
| 3/29/2009 | 1 | 1 | NA | NA | -69.2 | 0 | 19 | 64 | 15 | 0 | 0 |
| 3/30/2009 | 1 | 0 | 75 | 50 | -69.0 | 0 | 13 | 64 | 20 | 0 | 1 |
| 3/31/2009 | 1 | 0 | NA | NA | -70.0 | 0 | 12 | 70 | 12 | 1 | 1 |
| 4/1/2009 | 1 | 0 | 75 | 97 | -70.6 | 0 | 13 | 77 | 6 | 0 | 1 |
| 4/2/2009 | 1 | 0 | NA | NA | -70.7 | 0 | 12 | 68 | 14 | 0 | 2 |
| 4/3/2009 | 1 | 0 | 70 | 97 | -71.0 | 0 | 11 | 77 | 9 | 1 | 1 |
| 4/4/2009 | 1 | 0 | NA | NA | -68.5 | 3 | 10 | 76 | 9 | 0 | 1 |
| 4/5/2009 | 1 | 1 | NA | NA | -70.2 | 0 | 12 | 79 | 4 | 0 | 2 |
| 4/6/2009 | 1 | 1 | 70 | 97 | -67.7 | 0 | 10 | 72 | 12 | 0 | 1 |
| 4/7/2009 | 1 | 1 | NA | NA | -69.8 | 0 | 12 | 79 | 7 | 0 | 1 |
| 4/8/2009 | 1 | 1 | 70 | 97 | -59.1 | 2 | 11 | 79 | 6 | 0 | 1 |
| 4/9/2009 | 1 | 1 | NA | NA | -69.9 | 0 | 13 | 76 | 8 | 0 | 1 |
| 4/10/2009 | 1 | 0 | 70 | 97 | -70.7 | 0 | 13 | 77 | 7 | 0 | 1 |
| 4/11/2009 | 1 | 0 | NA | NA | -70.2 | 0 | 14 | 75 | 7 | 0 | 2 |
| 4/12/2009 | 1 | 0 | NA | NA | -70.8 | 0 | 10 | 75 | 10 | 0 | 2 |
| 4/13/2009 | 1 | 0 | 70 | 97 | -70.6 | 0 | 11 | 71 | 9 | 0 | 3 |
| 4/14/2009 | 1 | 0 | NA | NA | -71.0 | 0 | 12 | 74 | 8 | 0 | 1 |
| 4/15/2009 | 1 | 0 | 75 | 97 | -70.0 | 0 | 12 | 75 | 7 | 1 | 2 |
| 4/16/2009 | 1 | 1 | NA | NA | -70.9 | 0 | 12 | 76 | 7 | 0 | 2 |
| 4/17/2009 | 1 | 0 | 75 | 97 | -70.4 | 0 | 11 | 72 | 8 | 0 | 2 |
| 4/18/2009 | 1 | 0 | NA | NA | -70.4 | 0 | 12 | 76 | 4 | 2 | 2 |
| 4/19/2009 | 1 | 1 | NA | NA | -70.5 | 0 | 11 | 73 | 6 | 0 | 2 |
| 4/20/2009 | 1 | 0 | 75 | 97 | -69.9 | 0 | 11 | 71 | 8 | 0 | 3 |
| 4/21/2009 | 1 | 0 | NA | NA | -69.9 | 0 | 11 | 73 | 10 | 1 | 2 |
| 4/22/2009 | 1 | 1 | 75 | 97 | -70.9 | 0 | 11 | 73 | 10 | 0 | 1 |
| 4/23/2009 | 1 | 0 | NA | NA | -70.9 | 0 | 11 | 74 | 10 | 0 | 2 |
| 4/24/2009 | 1 | 1 | 75 | 97 | -71.2 | 0 | 10 | 79 | 8 | 0 | 2 |
| 4/25/2009 | 1 | 1 | NA | NA | -70.7 | 0 | 11 | 75 | 10 | 0 | 1 |
| 4/26/2009 | 1 | 1 | NA | NA | -69.9 | 0 | 13 | 76 | 8 | 0 | 1 |
| 4/27/2009 | 1 | 1 | 60 | 50 | -70.2 | 0 | 10 | 83 | 5 | 1 | 0 |
| 4/28/2009 | 1 | 1 | NA | NA | -68.9 | 0 | 10 | 86 | 3 | 0 | 0 |
| 4/29/2009 | 1 | 1 | 60 | 50 | -71.0 | 0 | 10 | 87 | 2 | 0 | 0 |
| 4/30/2009 | 1 | 1 | NA | NA | -70.7 | 0 | 11 | 77 | 6 | 0 | 1 |
| 5/1/2009 | 1 | 1 | 50 | 20 | -69.5 | 0 | 9 | 81 | 8 | 1 | 0 |
| 5/2/2009 | 1 | 1 | NA | NA | -70.2 | 0 | 9 | 76 | 13 | 0 | 0 |
| 5/3/2009 | 1 | 1 | NA | NA | -69.7 | 0 | 9 | 75 | 15 | 1 | 0 |
| 5/4/2009 | 1 | 1 | 50 | 5 | -68.9 | 0 | 9 | 72 | 17 | 1 | 0 |
| 5/5/2009 | 1 | 1 | NA | NA | -68.3 | 0 | 14 | 68 | 17 | 0 | 0 |
| 5/6/2009 | 1 | 1 | 40 | 20 | -70.4 | 0 | 11 | 71 | 18 | 0 | 0 |
| 5/7/2009 | 1 | 1 | NA | NA | -69.7 | 0 | 9 | 78 | 11 | 2 | 0 |
| 5/8/2009 | 1 | 1 | 70 | 20 | -71.0 | 0 | 9 | 82 | 8 | 0 | 0 |
| 5/9/2009 | 1 | 1 | NA | NA | -69.7 | 1 | 9 | 78 | 8 | 1 | 0 |
| 5/10/2009 | 1 | 1 | NA | NA | -69.8 | 0 | 12 | 80 | 8 | 0 | 0 |
| 5/11/2009 | 1 | 1 | 30 | 20 | -71.3 | 0 | 9 | 81 | 9 | 0 | 0 |
| 5/12/2009 | 0 | 1 | NA | NA | -70.2 | 0 | 9 | 73 | 17 | 0 | 0 |
| 5/13/2009 | 0 | 1 | 50 | 20 | -69.5 | 0 | 10 | 73 | 12 | 0 | 0 |
| 5/14/2009 | 1 | 1 | NA | NA | -69.5 | 0 | 11 | 75 | 14 | 0 | 0 |
| 5/15/2009 | 1 | 1 | 60 | 20 | -67.3 | 0 | 13 | 73 | 13 | 0 | 0 |
| 5/16/2009 | 1 | 1 | NA | NA | -68.9 | 0 | 10 | 69 | 16 | 1 | 0 |
| 5/17/2009 | 0 | 0 | NA | NA | -68.3 | 0 | 12 | 55 | 19 | 1 | 0 |
| 5/18/2009 | 0 | 0 | 40 | 20 | -67.5 | 0 | 10 | 57 | 25 | 0 | 0 |
| 5/19/2009 | 0 | 0 | NA | NA | -68.5 | 0 | 9 | 53 | 27 | 2 | 0 |
| 5/20/2009 | 0 | 1 | 0 | 0 | -67.8 | 0 | 11 | 59 | 24 | 0 | 0 |
| 5/21/2009 | 0 | 0 | NA | NA | -68.6 | 0 | 10 | 53 | 29 | 1 | 0 |
| 5/22/2009 | 0 | 0 | 0 | 0 | -66.4 | 0 | 10 | 48 | 29 | 2 | 0 |
| 5/23/2009 | 1 | 0 | NA | NA | -69.6 | 0 | 13 | 57 | 28 | 1 | 0 |
| 5/24/2009 | 0 | 0 | NA | NA | -69.5 | 0 | 12 | 58 | 25 | 0 | 0 |
| 5/25/2009 | 0 | 0 | 0 | 0 | -67.1 | 0 | 16 | 56 | 21 | 0 | 0 |
| 5/26/2009 | 0 | 0 | NA | NA | -65.9 | 0 | 12 | 59 | 18 | 1 | 0 |
| 5/27/2009 | 0 | 0 | 0 | 0 | -69.2 | 0 | 13 | 68 | 16 | 0 | 0 |
| 5/28/2009 | 0 | 0 | NA | NA | -67.4 | 0 | 13 | 69 | 16 | 0 | 0 |
| 5/29/2009 | 0 | 0 | 0 | 0 | -70.3 | 0 | 14 | 64 | 9 | 0 | 0 |
| 5/30/2009 | 0 | 0 | NA | NA | -67.6 | 0 | 7 | 63 | 10 | 0 | 0 |
| 5/31/2009 | 0 | 0 | 0 | 0 | -66.8 | 0 | 6 | 60 | 13 | 0 | 0 |
| 6/1/2009 | 0 | 0 | NA | NA | -70.4 | 0 | 18 | 72 | 10 | 0 | 0 |
| 6/2/2009 | 0 | 0 | 0 | 0 | -71.2 | 0 | 11 | 58 | 14 | 0 | 0 |
| 6/3/2009 | 0 | 0 | NA | NA | -68.1 | 0 | 9 | 67 | 12 | 0 | 0 |
| 6/4/2009 | 0 | 0 | 0 | 0 | -70.0 | 0 | 8 | 59 | 11 | 0 | 0 |
| 6/5/2009 | 0 | 0 | NA | NA | -69.3 | 0 | 22 | 69 | 7 | 0 | 0 |
| 6/6/2009 | 0 | 0 | 0 | 0 | -71.9 | 0 | 11 | 62 | 7 | 0 | 0 |
| 6/7/2009 | 0 | 0 | NA | NA | -73.2 | 0 | 10 | 80 | 6 | 0 | 0 |
| 6/8/2009 | 0 | 0 | 0 | 0 | -73.3 | 0 | 6 | 61 | 8 | 0 | 0 |
| 6/9/2009 | 0 | 0 | NA | NA | -73.6 | 0 | 17 | 79 | 2 | 0 | 0 |
| 6/10/2009 | 0 | 0 | 0 | 0 | -72.9 | 0 | 9 | 59 | 6 | 0 | 0 |
| 6/11/2009 | 0 | 0 | 0 | 0 | -69.5 | 0 | 11 | 75 | 15 | 0 | 0 |
| 6/12/2009 | 0 | 0 | 0 | 0 | -72.2 | 0 | 9 | 63 | 6 | 0 | 0 |
| 6/13/2009 | 0 | 0 | 0 | 0 | -73.7 | 0 | 14 | 76 | 3 | 0 | 0 |
| 6/14/2009 | 0 | 0 | 0 | 0 | -71.8 | 0 | 15 | 61 | 5 | 0 | 0 |
| 6/15/2009 | 0 | 0 | 0 | 0 | -71.6 | 0 | 24 | 71 | 4 | 0 | 0 |
| 6/16/2009 | 0 | 0 | 0 | 0 | -73.1 | 0 | 26 | 53 | 3 | 0 | 0 |
| 6/17/2009 | 0 | 0 | 0 | 0 | -69.6 | 0 | 18 | 60 | 11 | 0 | 0 |
| 6/18/2009 | 0 | 0 | 0 | 0 | -73.1 | 0 | 5 | 73 | 8 | 0 | 0 |
| 6/19/2009 | 0 | 0 | 0 | 0 | -67.5 | 0 | 5 | 81 | 14 | 0 | 0 |
| 6/20/2009 | 0 | 0 | 0 | 0 | -71.2 | 0 | 9 | 56 | 14 | 0 | 0 |
| 6/21/2009 | 0 | 0 | 0 | 0 | -72.2 | 0 | 9 | 60 | 12 | 0 | 0 |
| 6/22/2009 | 0 | 0 | 0 | 0 | -72.1 | 0 | 6 | 72 | 16 | 0 | 0 |
| 6/23/2009 | 0 | 0 | 0 | 0 | -71.9 | 0 | 11 | 67 | 21 | 0 | 0 |
| 6/24/2009 | 0 | 0 | 0 | 0 | -71.4 | 0 | 6 | 63 | 10 | 0 | 0 |
| 6/25/2009 | 0 | 0 | 0 | 0 | -70.8 | 0 | 3 | 69 | 11 | 0 | 0 |
| 6/26/2009 | 0 | 0 | 0 | 0 | -72.9 | 0 | 5 | 85 | 9 | 0 | 0 |
| 6/27/2009 | 0 | 0 | 0 | 0 | -72.3 | 0 | 6 | 86 | 8 | 0 | 0 |
| 6/28/2009 | 0 | 0 | 0 | 0 | -72.8 | 0 | 5 | 64 | 5 | 0 | 0 |
| 6/29/2009 | 0 | 0 | 0 | 0 | -71.2 | 0 | 9 | 60 | 11 | 0 | 0 |
| 6/30/2009 | 0 | 0 | 0 | 0 | -70.1 | 0 | 10 | 75 | 15 | 0 | 0 |
| 7/1/2009 | 0 | 0 | 0 | 0 | -72.1 | 0 | 4 | 77 | 14 | 0 | 0 |
| 7/2/2009 | 0 | 0 | 0 | 0 | -72.9 | 0 | 3 | 63 | 9 | 0 | 0 |
| 7/3/2009 | 0 | 0 | 0 | 0 | -70.9 | 0 | 31 | 33 | 10 | 0 | 0 |
| 7/4/2009 | 0 | 0 | 0 | 0 | -72.8 | 0 | 27 | 62 | 7 | 0 | 0 |
| 7/5/2009 | 0 | 0 | 0 | 0 | -74.4 | 0 | 29 | 62 | 7 | 0 | 0 |
| 7/6/2009 | 0 | 0 | 0 | 0 | -73.6 | 0 | 20 | 46 | 7 | 0 | 0 |
| 7/7/2009 | 0 | 0 | 0 | 0 | -72.2 | 0 | 20 | 41 | 9 | 0 | 0 |
| 7/8/2009 | 0 | 0 | 0 | 0 | -73.4 | 0 | 5 | 83 | 8 | 0 | 0 |
| 7/9/2009 | 0 | 0 | 0 | 0 | -75.0 | 0 | 5 | 91 | 3 | 0 | 0 |
| 7/10/2009 | 0 | 0 | 0 | 0 | -74.7 | 0 | 2 | 55 | 5 | 0 | 0 |
| 7/11/2009 | 0 | 0 | 0 | 0 | -73.4 | 0 | 6 | 75 | 9 | 0 | 0 |
| 7/12/2009 | 0 | 0 | 0 | 0 | -71.0 | 0 | 1 | 76 | 16 | 1 | 0 |
| 7/13/2009 | 0 | 0 | 0 | 0 | -72.0 | 0 | 1 | 79 | 13 | 1 | 0 |
| 7/14/2009 | 0 | 0 | 0 | 0 | -72.4 | 0 | 1 | 50 | 11 | 0 | 0 |
| 7/15/2009 | 0 | 0 | 0 | 0 | -73.9 | 0 | 0 | 76 | 19 | 0 | 0 |
| 7/16/2009 | 0 | 0 | 0 | 0 | -70.7 | 0 | 0 | 70 | 23 | 0 | 0 |
| 7/17/2009 | 0 | 0 | 0 | 0 | -72.2 | 0 | 0 | 73 | 21 | 0 | 0 |
| 7/18/2009 | 0 | 0 | 0 | 0 | -73.6 | 0 | 1 | 43 | 8 | 0 | 0 |
| 7/19/2009 | 0 | 0 | 0 | 0 | -74.5 | 0 | 1 | 81 | 16 | 0 | 0 |
| 7/20/2009 | 0 | 0 | 0 | 0 | -74.0 | 0 | 4 | 80 | 15 | 0 | 0 |
| 7/21/2009 | 0 | 0 | 0 | 0 | -72.9 | 0 | 4 | 72 | 19 | 0 | 0 |
| 7/22/2009 | 0 | 0 | 0 | 0 | -71.9 | 0 | 1 | 41 | 17 | 0 | 0 |
| 7/23/2009 | 0 | 0 | 0 | 0 | -72.9 | 0 | 3 | 75 | 21 | 0 | 0 |
| 7/24/2009 | 0 | 0 | 0 | 0 | -74.2 | 0 | 5 | 71 | 24 | 0 | 0 |
| 7/25/2009 | 0 | 0 | 0 | 0 | -73.7 | 0 | 6 | 65 | 25 | 0 | 0 |
| 7/26/2009 | 0 | 0 | 0 | 0 | -72.4 | 0 | 11 | 36 | 20 | 0 | 0 |
| 7/27/2009 | 0 | 0 | 0 | 0 | -72.1 | 0 | 10 | 62 | 28 | 0 | 0 |
| 7/28/2009 | 0 | 0 | 0 | 0 | -73.1 | 0 | 8 | 68 | 24 | 0 | 0 |
| 7/29/2009 | 0 | 0 | 0 | 0 | -73.5 | 0 | 9 | 65 | 16 | 0 | 0 |
| 7/30/2009 | 0 | 0 | 0 | 0 | -73.9 | 0 | 4 | 57 | 11 | 0 | 0 |
| 7/31/2009 | 0 | 0 | 0 | 0 | -74.1 | 0 | 4 | 74 | 22 | 0 | 0 |
| 8/1/2009 | 0 | 0 | 0 | 0 | -72.8 | 0 | 4 | 70 | 26 | 0 | 0 |
| 8/2/2009 | 0 | 0 | 0 | 0 | -73.8 | 0 | 5 | 63 | 16 | 1 | 0 |
| 8/3/2009 | 0 | 0 | 0 | 0 | -72.6 | 0 | 3 | 44 | 23 | 0 | 0 |
| 8/4/2009 | 0 | 0 | 0 | 0 | -70.9 | 0 | 1 | 57 | 42 | 0 | 0 |
| 8/5/2009 | 0 | 0 | 0 | 0 | -71.4 | 0 | 4 | 53 | 43 | 0 | 0 |
| 8/6/2009 | 0 | 0 | 0 | 0 | -72.0 | 0 | 3 | 48 | 30 | 0 | 0 |
| 8/7/2009 | 0 | 0 | 0 | 0 | -72.7 | 0 | 3 | 49 | 29 | 0 | 0 |
| 8/8/2009 | 0 | 0 | 0 | 0 | -73.0 | 0 | 6 | 65 | 29 | 0 | 0 |
| 8/9/2009 | 0 | 0 | 0 | 0 | -73.2 | 0 | 5 | 64 | 31 | 0 | 0 |
| 8/10/2009 | 0 | 0 | 0 | 0 | -72.8 | 0 | 3 | 49 | 26 | 0 | 0 |
| 8/11/2009 | 0 | 0 | 0 | 0 | -73.5 | 0 | 3 | 59 | 22 | 0 | 0 |
| 8/12/2009 | 0 | 0 | 0 | 0 | -73.3 | 0 | 1 | 71 | 28 | 0 | 0 |
| 8/13/2009 | 0 | 0 | 0 | 0 | -73.5 | 0 | 2 | 68 | 27 | 0 | 0 |
| 8/14/2009 | 0 | 0 | 0 | 0 | -73.2 | 0 | 3 | 57 | 21 | 0 | 0 |
| 8/15/2009 | 0 | 0 | 0 | 0 | -73.1 | 0 | 2 | 52 | 22 | 0 | 0 |
| 8/16/2009 | 0 | 0 | 0 | 0 | -72.8 | 0 | 3 | 56 | 41 | 0 | 0 |
| 8/17/2009 | 0 | 0 | 0 | 0 | -72.5 | 0 | 2 | 60 | 38 | 0 | 0 |
| 8/18/2009 | 0 | 0 | 0 | 0 | -72.4 | 0 | 2 | 50 | 32 | 0 | 0 |
| 8/19/2009 | 0 | 0 | 0 | 0 | -72.4 | 0 | 3 | 51 | 27 | 0 | 0 |
| 8/20/2009 | 0 | 0 | 0 | 0 | -71.4 | 0 | 0 | 57 | 42 | 0 | 0 |
| 8/21/2009 | 0 | 0 | 0 | 0 | -71.0 | 0 | 1 | 53 | 45 | 0 | 0 |
| 8/22/2009 | 0 | 0 | 0 | 0 | -71.7 | 0 | 0 | 43 | 41 | 0 | 0 |
| 8/23/2009 | 0 | 0 | 0 | 0 | -72.0 | 0 | 4 | 49 | 28 | 0 | 0 |
| 8/24/2009 | 0 | 0 | 0 | 0 | -72.6 | 0 | 3 | 61 | 36 | 0 | 0 |
| 8/25/2009 | 0 | 0 | 0 | 0 | -71.4 | 0 | 6 | 45 | 38 | 1 | 0 |
| 8/26/2009 | 0 | 0 | 0 | 0 | -70.9 | 0 | 7 | 45 | 36 | 1 | 0 |
| 8/27/2009 | 0 | 0 | 0 | 0 | -69.9 | 0 | 8 | 41 | 31 | 0 | 0 |
| 8/28/2009 | 0 | 0 | 0 | 0 | -70.8 | 0 | 10 | 62 | 28 | 0 | 0 |
| 8/29/2009 | 0 | 0 | 0 | 0 | -71.0 | 0 | 6 | 50 | 36 | 0 | 0 |
| 8/30/2009 | 0 | 0 | 0 | 0 | -72.0 | 0 | 1 | 59 | 27 | 0 | 0 |
| 8/31/2009 | 0 | 0 | 0 | 0 | -70.4 | 0 | 3 | 46 | 25 | 1 | 0 |
| 9/1/2009 | 0 | 0 | 0 | 0 | -71.6 | 0 | 5 | 59 | 35 | 0 | 0 |
| 9/2/2009 | 0 | 0 | 0 | 0 | -68.6 | 0 | 4 | 46 | 34 | 0 | 0 |
| 9/3/2009 | 0 | 0 | 0 | 0 | -71.9 | 0 | 4 | 73 | 18 | 0 | 0 |
| 9/4/2009 | 0 | 0 | 0 | 0 | -73.6 | 0 | 6 | 63 | 10 | 0 | 0 |
| 9/5/2009 | 0 | 0 | 0 | 0 | -71.6 | 0 | 10 | 70 | 18 | 0 | 0 |
| 9/6/2009 | 0 | 0 | 0 | 0 | -73.2 | 0 | 3 | 60 | 17 | 1 | 0 |
| 9/7/2009 | 0 | 0 | 0 | 0 | -71.4 | 0 | 1 | 60 | 30 | 0 | 0 |
| 9/8/2009 | 0 | 0 | 0 | 0 | -71.2 | 0 | 4 | 53 | 18 | 0 | 0 |
| 9/9/2009 | 0 | 0 | 0 | 0 | -71.3 | 0 | 2 | 65 | 31 | 0 | 0 |
| 9/10/2009 | 0 | 0 | 0 | 0 | -71.5 | 0 | 0 | 57 | 25 | 1 | 0 |
| 9/11/2009 | 0 | 0 | 0 | 0 | -73.5 | 0 | 3 | 68 | 29 | 0 | 0 |
| 9/12/2009 | 0 | 0 | 0 | 0 | -72.6 | 0 | 0 | 50 | 26 | 0 | 0 |
| 9/13/2009 | 0 | 0 | 0 | 0 | -71.0 | 0 | 1 | 60 | 35 | 0 | 0 |
| 9/14/2009 | 0 | 0 | 0 | 0 | -72.7 | 0 | 4 | 57 | 24 | 0 | 0 |
| 9/15/2009 | 0 | 0 | 0 | 0 | -72.2 | 0 | 4 | 64 | 32 | 0 | 0 |
| 9/16/2009 | 0 | 0 | 0 | 0 | -72.7 | 0 | 4 | 51 | 25 | 0 | 0 |
| 9/17/2009 | 0 | 0 | 0 | 0 | -71.2 | 0 | 1 | 52 | 41 | 0 | 0 |
| 9/18/2009 | 0 | 0 | 0 | 0 | -70.9 | 0 | 2 | 42 | 33 | 0 | 0 |
| 9/19/2009 | 0 | 0 | 0 | 0 | -70.1 | 0 | 5 | 51 | 39 | 0 | 0 |
| 9/20/2009 | 0 | 0 | 0 | 0 | -68.7 | 0 | 6 | 44 | 27 | 1 | 0 |
| 9/21/2009 | 0 | 0 | 0 | 0 | -71.8 | 0 | 8 | 54 | 39 | 0 | 0 |
| 9/22/2009 | 0 | 0 | 0 | 0 | -71.2 | 0 | 7 | 41 | 34 | 0 | 0 |
| 9/23/2009 | 0 | 0 | 0 | 0 | -70.2 | 0 | 7 | 45 | 41 | 0 | 0 |
| 9/24/2009 | 0 | 0 | 0 | 0 | -70.1 | 0 | 6 | 43 | 34 | 0 | 0 |
| 9/25/2009 | 0 | 0 | 0 | 0 | -70.9 | 1 | 10 | 45 | 42 | 0 | 0 |
| 9/26/2009 | 0 | 0 | 0 | 0 | -69.9 | 0 | 8 | 45 | 34 | 0 | 0 |
| 9/27/2009 | 0 | 0 | 0 | 0 | -69.9 | 0 | 10 | 40 | 45 | 0 | 0 |
| 9/28/2009 | 0 | 0 | 0 | 0 | -69.9 | 0 | 12 | 44 | 30 | 0 | 0 |
| 9/29/2009 | 0 | 0 | 0 | 0 | -66.1 | 0 | 16 | 35 | 43 | 0 | 0 |
| 9/30/2009 | 0 | 0 | 0 | 0 | -64.8 | 0 | 23 | 25 | 51 | 0 | 0 |
| 10/1/2009 | 0 | 0 | 0 | 0 | -65.2 | 0 | 18 | 25 | 51 | 1 | 0 |
| 10/2/2009 | 0 | 0 | 0 | 0 | -62.7 | 0 | 16 | 26 | 48 | 0 | 0 |
| 10/3/2009 | 0 | 0 | 0 | 0 | -65.4 | 0 | 16 | 25 | 49 | 2 | 0 |
| 10/4/2009 | 0 | 0 | 0 | 0 | -64.8 | 0 | 10 | 30 | 51 | 0 | 0 |
| 10/5/2009 | 0 | 0 | 0 | 0 | -64.5 | 0 | 17 | 31 | 49 | 0 | 0 |
| 10/6/2009 | 0 | 0 | 0 | 0 | -64.1 | 0 | 12 | 25 | 49 | 1 | 1 |
| 10/7/2009 | 0 | 0 | 0 | 0 | -62.3 | 0 | 13 | 28 | 52 | 0 | 0 |
| 10/8/2009 | 0 | 0 | 0 | 0 | -65.4 | 0 | 13 | 30 | 52 | 0 | 0 |
| 10/9/2009 | 0 | 0 | 0 | 0 | -66.1 | 1 | 15 | 30 | 49 | 0 | 0 |
| 10/10/2009 | 0 | 0 | 0 | 0 | -63.7 | 1 | 18 | 17 | 48 | 1 | 0 |
| 10/11/2009 | 0 | 0 | 0 | 0 | -61.3 | 1 | 22 | 18 | 52 | 0 | 0 |
| 10/12/2009 | 0 | 0 | 0 | 0 | -65.4 | 0 | 23 | 32 | 44 | 1 | 0 |
| 10/13/2009 | 0 | 0 | 0 | 0 | -66.3 | 0 | 24 | 35 | 41 | 0 | 0 |
| 10/14/2009 | 0 | 0 | 0 | 0 | -65.8 | 0 | 24 | 32 | 43 | 0 | 0 |
| 10/15/2009 | 0 | 0 | 0 | 0 | -60.8 | 1 | 21 | 33 | 44 | 0 | 0 |
| 10/16/2009 | 0 | 0 | 0 | 0 | -58.9 | 0 | 18 | 34 | 46 | 0 | 0 |
| 10/17/2009 | 0 | 0 | 0 | 0 | -65.1 | 1 | 19 | 39 | 42 | 0 | 0 |
| 10/18/2009 | 0 | 0 | 0 | 0 | -66.5 | 0 | 16 | 35 | 46 | 0 | 0 |
| 10/19/2009 | 0 | 0 | 0 | 0 | -63.1 | 0 | 13 | 36 | 43 | 0 | 0 |
| 10/20/2009 | 0 | 0 | 0 | 0 | -63.8 | 0 | 18 | 26 | 47 | 1 | 0 |
| 10/21/2009 | 0 | 0 | 0 | 0 | -63.8 | 1 | 17 | 28 | 42 | 0 | 0 |
| 10/22/2009 | 0 | 0 | 0 | 0 | -63.4 | 0 | 14 | 31 | 37 | 1 | 1 |
| 10/23/2009 | 0 | 0 | 0 | 0 | -64.7 | 0 | 16 | 35 | 40 | 0 | 0 |
| 10/24/2009 | 0 | 0 | 0 | 0 | -58.7 | 1 | 26 | 36 | 38 | 0 | 1 |
| 10/25/2009 | 0 | 0 | 0 | 0 | -57.5 | 1 | 17 | 32 | 46 | 0 | 2 |
| 10/26/2009 | 0 | 0 | 0 | 0 | -61.0 | 0 | 21 | 36 | 42 | 0 | 0 |
| 10/27/2009 | 0 | 0 | 0 | 0 | -60.3 | 2 | 20 | 41 | 38 | 0 | 1 |
| 10/28/2009 | 0 | 0 | 0 | 0 | -59.0 | 0 | 20 | 36 | 43 | 0 | 0 |
| 10/29/2009 | 0 | 0 | 0 | 0 | -56.6 | 0 | 15 | 32 | 46 | 0 | 0 |
| 10/30/2009 | 0 | 0 | 0 | 0 | -60.3 | 0 | 18 | 35 | 45 | 0 | 0 |
| 10/31/2009 | 0 | 0 | 0 | 0 | -61.0 | 0 | 19 | 36 | 43 | 0 | 0 |
| 11/1/2009 | 0 | 0 | 0 | 0 | -57.6 | 0 | 19 | 26 | 54 | 0 | 1 |
| 11/2/2009 | 0 | 0 | 0 | 0 | -57.7 | 0 | 15 | 22 | 56 | 0 | 1 |
| 11/3/2009 | 0 | 0 | 0 | 0 | -58.8 | 0 | 15 | 22 | 54 | 0 | 0 |
| 11/4/2009 | 0 | 0 | 0 | 0 | -59.2 | 0 | 14 | 20 | 53 | 0 | 1 |
| 11/5/2009 | 0 | 0 | 0 | 0 | -53.7 | 0 | 20 | 15 | 51 | 1 | 1 |
| 11/6/2009 | 0 | 0 | 0 | 0 | -52.6 | 0 | 18 | 14 | 55 | 1 | 1 |
| 11/7/2009 | 0 | 0 | 0 | 0 | -55.1 | 0 | 17 | 27 | 51 | 0 | 1 |
| 11/8/2009 | 0 | 0 | 0 | 0 | -51.2 | 4 | 17 | 27 | 49 | 1 | 1 |
| 11/9/2009 | 0 | 0 | 0 | 0 | -49.8 | 0 | 17 | 27 | 53 | 0 | 1 |
| 11/10/2009 | 0 | 0 | 0 | 0 | -53.5 | 0 | 14 | 29 | 51 | 0 | 0 |
| 11/11/2009 | 0 | 0 | 0 | 0 | -51.1 | 0 | 10 | 31 | 48 | 1 | 0 |
| 11/12/2009 | 0 | 0 | 0 | 0 | -56.1 | 0 | 14 | 39 | 45 | 0 | 0 |
| 11/13/2009 | 0 | 0 | 0 | 0 | -54.3 | 0 | 9 | 33 | 47 | 1 | 1 |
| 11/14/2009 | 0 | 0 | 0 | 0 | -52.9 | 0 | 9 | 37 | 46 | 1 | 0 |
| 11/15/2009 | 0 | 0 | 0 | 0 | -54.7 | 0 | 6 | 37 | 46 | 1 | 1 |
| 11/16/2009 | 0 | 0 | 0 | 0 | -53.7 | 0 | 12 | 47 | 38 | 1 | 1 |
| 11/17/2009 | 0 | 0 | 0 | 0 | -55.2 | 0 | 13 | 40 | 45 | 0 | 1 |
| 11/18/2009 | 0 | 0 | 0 | 0 | -56.2 | 0 | 9 | 38 | 52 | 0 | 0 |
| 11/19/2009 | 0 | 0 | 0 | 0 | -55.9 | 0 | 7 | 31 | 46 | 1 | 1 |
| 11/20/2009 | 0 | 0 | 0 | 0 | -55.2 | 0 | 64 | 12 | 13 | 0 | 0 |
| 11/21/2009 | 0 | 0 | 0 | 0 | -56.7 | 0 | 27 | 26 | 35 | 1 | 0 |
| 11/22/2009 | 0 | 0 | 0 | 0 | -55.0 | 0 | 32 | 12 | 42 | 1 | 1 |
| 11/23/2009 | 0 | 0 | 0 | 0 | -55.8 | 0 | 57 | 15 | 19 | 0 | 0 |
| 11/24/2009 | 0 | 0 | 0 | 0 | -56.9 | 0 | 24 | 41 | 36 | 0 | 0 |
| 11/25/2009 | 0 | 0 | 0 | 0 | -57.4 | 0 | 13 | 41 | 44 | 0 | 0 |
| 11/26/2009 | 0 | 0 | 0 | 0 | -52.3 | 0 | 3 | 33 | 55 | 1 | 0 |
| 11/27/2009 | 0 | 0 | 0 | 0 | -58.9 | 0 | 8 | 36 | 47 | 1 | 0 |
| 11/28/2009 | 0 | 0 | 0 | 0 | -60.4 | 0 | 11 | 34 | 49 | 0 | 1 |
| 11/29/2009 | 0 | 0 | 0 | 0 | -58.4 | 0 | 13 | 33 | 41 | 1 | 1 |
| 11/30/2009 | 0 | 0 | 0 | 0 | -52.6 | 0 | 37 | 12 | 25 | 0 | 0 |
| 12/1/2009 | 0 | 0 | 0 | 0 | -57.5 | 0 | 72 | 1 | 15 | 0 | 0 |
| 12/2/2009 | 0 | 0 | 0 | 0 | -59.7 | 0 | 34 | 19 | 38 | 0 | 0 |
| 12/3/2009 | 0 | 0 | 0 | 0 | -58.0 | 0 | 21 | 32 | 42 | 0 | 0 |
| 12/4/2009 | 0 | 0 | 0 | 0 | -58.9 | 0 | 31 | 15 | 35 | 1 | 1 |
| 12/5/2009 | 0 | 0 | 0 | 0 | -57.2 | 17 | 57 | 0 | 13 | 0 | 0 |
| 12/6/2009 | 0 | 0 | 0 | 0 | -63.1 | 0 | 72 | 4 | 13 | 0 | 0 |
| 12/7/2009 | 0 | 0 | 0 | 0 | -58.6 | 4 | 65 | 1 | 14 | 0 | 0 |
| 12/8/2009 | 0 | 0 | 0 | 0 | -59.2 | 7 | 72 | 8 | 8 | 0 | 0 |
| 12/9/2009 | 0 | 0 | 0 | 0 | -62.5 | 0 | 50 | 28 | 15 | 0 | 0 |
| 12/10/2009 | 0 | 0 | 0 | 0 | -62.7 | 0 | 48 | 30 | 18 | 0 | 0 |
| 12/11/2009 | 0 | 0 | 0 | 0 | -65.5 | 0 | 23 | 47 | 27 | 0 | 0 |
| 12/12/2009 | 0 | 0 | 0 | 0 | -65.2 | 0 | 26 | 42 | 32 | 0 | 0 |
| 12/13/2009 | 0 | 0 | 0 | 0 | -65.4 | 0 | 13 | 49 | 30 | 2 | 0 |
| 12/14/2009 | 0 | 0 | 0 | 0 | -62.7 | 0 | 12 | 46 | 33 | 1 | 0 |
| 12/15/2009 | 0 | 0 | 0 | 0 | -63.8 | 0 | 15 | 53 | 26 | 1 | 0 |
| 12/16/2009 | 0 | 0 | 0 | 0 | -62.5 | 0 | 17 | 47 | 32 | 0 | 0 |
| 12/17/2009 | 0 | 0 | 0 | 0 | -64.7 | 0 | 14 | 49 | 32 | 0 | 0 |
| 12/18/2009 | 0 | 0 | 0 | 0 | -64.3 | 0 | 8 | 42 | 40 | 0 | 1 |
| 12/19/2009 | 0 | 0 | 0 | 0 | -63.4 | 0 | 17 | 33 | 33 | 1 | 1 |
| 12/20/2009 | 0 | 0 | 0 | 0 | -62.9 | 0 | 29 | 36 | 25 | 1 | 0 |
| 12/21/2009 | 0 | 0 | 0 | 0 | -65.6 | 0 | 11 | 54 | 25 | 1 | 1 |
| 12/22/2009 | 0 | 0 | 0 | 0 | -66.5 | 0 | 15 | 42 | 36 | 1 | 0 |
| 12/23/2009 | 0 | 0 | 0 | 0 | -67.7 | 0 | 15 | 53 | 32 | 0 | 0 |
| 12/24/2009 | 0 | 0 | 0 | 0 | -67.7 | 0 | 11 | 55 | 28 | 1 | 0 |
| 12/25/2009 | 0 | 0 | 0 | 0 | -64.7 | 0 | 9 | 59 | 22 | 2 | 1 |
| 12/26/2009 | 0 | 0 | 0 | 0 | -65.6 | 0 | 7 | 55 | 28 | 1 | 0 |
| 12/27/2009 | 0 | 0 | 0 | 0 | -66.0 | 0 | 12 | 47 | 36 | 1 | 0 |
| 12/28/2009 | 0 | 0 | 0 | 0 | -67.5 | 0 | 11 | 40 | 45 | 0 | 0 |
| 12/29/2009 | 0 | 0 | 0 | 0 | -65.1 | 0 | 10 | 40 | 40 | 0 | 1 |
| 12/30/2009 | 0 | 0 | 0 | 0 | -64.7 | 0 | 10 | 36 | 45 | 1 | 0 |
| 12/31/2009 | 0 | 0 | 0 | 0 | -65.2 | 0 | 17 | 42 | 39 | 0 | 0 |
| 1/1/2010 | 0 | 0 | 0 | 0 | -66.4 | 0 | 15 | 45 | 35 | 1 | 0 |
| 1/2/2010 | 0 | 0 | 0 | 0 | -63.1 | 0 | 12 | 44 | 35 | 1 | 0 |
| 1/3/2010 | 0 | 0 | 0 | 0 | -62.1 | 0 | 13 | 41 | 33 | 1 | 1 |
| 1/4/2010 | 0 | 0 | 0 | 0 | -59.7 | 0 | 10 | 30 | 39 | 2 | 2 |
| 1/5/2010 | 0 | 0 | 0 | 0 | -60.8 | 0 | 36 | 45 | 18 | 0 | 0 |
| 1/6/2010 | 0 | 0 | 0 | 0 | -64.9 | 0 | 38 | 32 | 21 | 1 | 2 |
| 1/7/2010 | 0 | 0 | 0 | 0 | -62.6 | 0 | 14 | 44 | 29 | 1 | 1 |
| 1/8/2010 | 0 | 0 | 0 | 0 | -60.6 | 0 | 17 | 35 | 36 | 1 | 3 |
| 1/9/2010 | 0 | 0 | 0 | 0 | -62.4 | 0 | 12 | 51 | 24 | 1 | 1 |
| 1/10/2010 | 0 | 0 | 0 | 0 | -62.5 | 0 | 14 | 31 | 43 | 1 | 1 |
| 1/11/2010 | 0 | 0 | 15 | 5 | -63.4 | 0 | 16 | 40 | 41 | 0 | 0 |
| 1/12/2010 | 0 | 0 | NA | NA | -64.8 | 0 | 13 | 51 | 36 | 0 | 0 |
| 1/13/2010 | 1 | 0 | 30 | 10 | -68.0 | 0 | 10 | 44 | 46 | 0 | 0 |
| 1/14/2010 | 0 | 0 | NA | NA | -63.8 | 0 | 16 | 47 | 35 | 1 | 0 |
| 1/15/2010 | 0 | 0 | 30 | 10 | -61.9 | 0 | 18 | 57 | 21 | 1 | 0 |
| 1/16/2010 | 0 | 0 | NA | NA | -61.8 | 0 | 13 | 69 | 17 | 0 | 0 |
| 1/17/2010 | 1 | 0 | NA | NA | -64.3 | 0 | 15 | 45 | 36 | 0 | 1 |
| 1/18/2010 | 1 | 0 | 60 | 20 | -64.9 | 0 | 23 | 47 | 29 | 0 | 0 |
| 1/19/2010 | 1 | 0 | NA | NA | -65.0 | 0 | 19 | 50 | 29 | 1 | 0 |
| 1/20/2010 | 1 | 0 | 80 | 20 | -68.3 | 0 | 11 | 74 | 15 | 0 | 0 |
| 1/21/2010 | 1 | 0 | NA | NA | -68.6 | 0 | 11 | 63 | 26 | 0 | 0 |
| 1/22/2010 | 1 | 0 | 80 | 20 | -64.6 | 0 | 13 | 54 | 27 | 1 | 0 |
| 1/23/2010 | 0 | 0 | NA | NA | -66.3 | 0 | 25 | 62 | 13 | 0 | 0 |
| 1/24/2010 | 1 | 0 | NA | NA | -66.7 | 0 | 29 | 52 | 18 | 0 | 0 |
| 1/25/2010 | 0 | 0 | 80 | 30 | -66.6 | 0 | 28 | 50 | 21 | 0 | 0 |
| 1/26/2010 | 1 | 0 | NA | NA | -67.5 | 0 | 20 | 39 | 40 | 0 | 0 |
| 1/27/2010 | 1 | 0 | 80 | 30 | -67.4 | 0 | 19 | 36 | 44 | 0 | 0 |
| 1/28/2010 | 1 | 0 | NA | NA | -64.4 | 0 | 16 | 32 | 51 | 0 | 0 |
| 1/29/2010 | 1 | 0 | 80 | 30 | -64.9 | 0 | 15 | 39 | 45 | 1 | 0 |
| 1/30/2010 | 1 | 0 | NA | NA | -65.9 | 0 | 15 | 44 | 40 | 1 | 0 |
| 1/31/2010 | 1 | 0 | NA | NA | -65.0 | 0 | 21 | 32 | 44 | 1 | 0 |
| 2/1/2010 | 1 | 0 | 80 | 30 | -67.2 | 0 | 18 | 37 | 43 | 1 | 0 |
| 2/2/2010 | 1 | 0 | NA | NA | -67.1 | 0 | 20 | 40 | 39 | 0 | 0 |
| 2/3/2010 | 1 | 0 | 80 | 30 | -67.0 | 0 | 19 | 41 | 37 | 0 | 0 |
| 2/4/2010 | 1 | 0 | NA | NA | -66.1 | 1 | 17 | 40 | 41 | 0 | 0 |
| 2/5/2010 | 1 | 0 | 80 | 30 | -65.4 | 0 | 21 | 36 | 40 | 1 | 0 |
| 2/6/2010 | 1 | 0 | NA | NA | -66.2 | 1 | 22 | 38 | 37 | 1 | 0 |
| 2/7/2010 | 1 | 0 | NA | NA | -65.6 | 0 | 18 | 39 | 41 | 1 | 0 |
| 2/8/2010 | 1 | 0 | 85 | 71 | -67.7 | 0 | 17 | 38 | 41 | 2 | 0 |
| 2/9/2010 | 1 | 0 | NA | NA | -65.8 | 0 | 14 | 48 | 35 | 1 | 0 |
| 2/10/2010 | 1 | 0 | 85 | 71 | -66.0 | 0 | 14 | 54 | 31 | 0 | 0 |
| 2/11/2010 | 1 | 0 | NA | NA | -65.9 | 0 | 19 | 40 | 39 | 0 | 0 |
| 2/12/2010 | 1 | 0 | 85 | 71 | -67.8 | 0 | 15 | 58 | 27 | 0 | 0 |
| 2/13/2010 | 1 | 0 | NA | NA | -67.8 | 1 | 19 | 64 | 14 | 0 | 0 |
| 2/14/2010 | 1 | 0 | NA | NA | -65.3 | 0 | 16 | 67 | 15 | 1 | 0 |
| 2/15/2010 | 1 | 0 | 85 | 71 | -67.7 | 1 | 13 | 73 | 11 | 1 | 0 |
| 2/16/2010 | 1 | 0 | NA | NA | -68.7 | 0 | 13 | 77 | 8 | 1 | 0 |
| 2/17/2010 | NA | NA | 85 | 71 | -68.9 | 0 | 12 | 77 | 9 | 1 | 0 |
| 2/18/2010 | NA | NA | NA | NA | -59.2 | 0 | 15 | 72 | 11 | 1 | 0 |
| 2/19/2010 | NA | NA | 85 | 71 | -67.0 | 0 | 16 | 52 | 29 | 1 | 0 |
| 2/20/2010 | NA | NA | NA | NA | -65.5 | 0 | 17 | 52 | 29 | 1 | 0 |
| 2/21/2010 | NA | NA | NA | NA | -68.2 | 0 | 16 | 55 | 28 | 1 | 0 |
| 2/22/2010 | NA | NA | 85 | 71 | -64.5 | 0 | 16 | 51 | 30 | 1 | 0 |
| 2/23/2010 | NA | NA | NA | NA | -65.3 | 1 | 15 | 49 | 32 | 1 | 0 |
| 2/24/2010 | NA | NA | 85 | 71 | -57.8 | 2 | 12 | 50 | 31 | 1 | 1 |
| 2/25/2010 | NA | NA | NA | NA | -66.7 | 1 | 15 | 55 | 27 | 1 | 0 |
| 2/26/2010 | NA | NA | 85 | 71 | -65.3 | 0 | 14 | 52 | 33 | 1 | 0 |
| 2/27/2010 | NA | NA | NA | NA | -67.2 | 0 | 13 | 49 | 34 | 0 | 0 |
| 2/28/2010 | NA | NA | NA | NA | -65.3 | 0 | 12 | 50 | 31 | 2 | 0 |
| 3/1/2010 | NA | NA | 85 | 71 | -61.3 | 1 | 15 | 51 | 29 | 1 | 1 |
| 3/2/2010 | NA | NA | NA | NA | -66.2 | 0 | 18 | 61 | 19 | 2 | 0 |
| 3/3/2010 | NA | NA | 85 | 71 | -67.2 | 0 | 15 | 53 | 28 | 1 | 0 |
| 3/4/2010 | NA | NA | NA | NA | -63.9 | 0 | 15 | 54 | 26 | 1 | 1 |
| 3/5/2010 | NA | NA | 85 | 71 | -63.1 | 0 | 14 | 59 | 20 | 2 | 0 |
| 3/6/2010 | NA | NA | NA | NA | -63.8 | 1 | 16 | 53 | 23 | 1 | 0 |
| 3/7/2010 | NA | NA | NA | NA | -62.0 | 1 | 15 | 52 | 24 | 1 | 0 |
| 3/8/2010 | NA | NA | 85 | 71 | -63.4 | 1 | 13 | 48 | 30 | 1 | 1 |
| 3/9/2010 | NA | NA | NA | NA | -63.5 | 0 | 14 | 37 | 37 | 2 | 1 |
| 3/10/2010 | NA | NA | 85 | 71 | -64.3 | 0 | 17 | 29 | 45 | 2 | 1 |
| 3/11/2010 | NA | NA | NA | NA | -63.7 | 0 | 13 | 39 | 45 | 2 | 0 |
| 3/12/2010 | NA | NA | 85 | 71 | -62.3 | 0 | 13 | 61 | 26 | 0 | 0 |
| 3/13/2010 | NA | NA | NA | NA | -64.8 | 0 | 16 | 71 | 9 | 1 | 1 |
| 3/14/2010 | NA | NA | NA | NA | -65.7 | 0 | 13 | 76 | 11 | 1 | 0 |
| 3/15/2010 | NA | NA | 85 | 71 | -64.5 | 1 | 13 | 78 | 7 | 1 | 1 |
| 3/16/2010 | NA | NA | NA | NA | -59.6 | 1 | 14 | 75 | 6 | 1 | 1 |
| 3/17/2010 | NA | NA | 85 | 71 | -65.5 | 1 | 12 | 80 | 6 | 1 | 0 |
| 3/18/2010 | NA | NA | 75 | 71 | -62.8 | 1 | 13 | 71 | 12 | 1 | 0 |
| 3/19/2010 | NA | NA | 70 | 50 | -64.3 | 0 | 13 | 69 | 17 | 0 | 0 |
| 3/20/2010 | NA | NA | NA | NA | -66.5 | 0 | 17 | 64 | 18 | 1 | 0 |
| 3/21/2010 | NA | NA | NA | NA | -63.9 | 0 | 12 | 66 | 18 | 1 | 0 |
| 3/22/2010 | NA | NA | 70 | 71 | -64.7 | 0 | 15 | 56 | 26 | 0 | 0 |
| 3/23/2010 | NA | NA | NA | NA | -63.4 | 1 | 10 | 62 | 27 | 1 | 0 |
| 3/24/2010 | NA | NA | 80 | 71 | -62.7 | 0 | 9 | 65 | 25 | 0 | 0 |
| 3/25/2010 | NA | NA | NA | NA | -64.7 | 0 | 14 | 69 | 16 | 1 | 0 |
| 3/26/2010 | NA | NA | 80 | 71 | -64.2 | 1 | 10 | 68 | 20 | 1 | 0 |
| 3/27/2010 | NA | NA | NA | NA | -65.7 | 0 | 9 | 67 | 22 | 1 | 0 |
| 3/28/2010 | NA | NA | NA | NA | -68.3 | 1 | 8 | 59 | 29 | 3 | 0 |
| 3/29/2010 | NA | NA | 75 | 71 | -65.8 | 0 | 9 | 56 | 32 | 2 | 1 |
| 3/30/2010 | NA | NA | NA | NA | -66.1 | 0 | 9 | 60 | 30 | 1 | 0 |
| 3/31/2010 | NA | NA | 75 | 81 | -66.3 | 0 | 10 | 60 | 26 | 2 | 0 |
| 4/1/2010 | NA | NA | NA | NA | -66.7 | 0 | 10 | 57 | 30 | 1 | 0 |
| 4/2/2010 | NA | NA | 75 | 81 | -68.6 | 0 | 13 | 59 | 25 | 3 | 0 |
| 4/3/2010 | NA | NA | NA | NA | -66.2 | 0 | 12 | 50 | 34 | 3 | 0 |
| 4/4/2010 | NA | NA | NA | NA | -66.4 | 0 | 11 | 60 | 26 | 2 | 0 |
| 4/5/2010 | NA | NA | 75 | 81 | -67.2 | 0 | 13 | 58 | 26 | 2 | 0 |
| 4/6/2010 | NA | NA | NA | NA | -67.7 | 0 | 13 | 52 | 32 | 3 | 0 |
| 4/7/2010 | NA | NA | 75 | 81 | -66.6 | 0 | 13 | 47 | 35 | 3 | 0 |
| 4/8/2010 | NA | NA | NA | NA | -68.7 | 0 | 14 | 53 | 29 | 2 | 0 |
| 4/9/2010 | NA | NA | 75 | 81 | -67.0 | 0 | 12 | 48 | 35 | 5 | 0 |
| 4/10/2010 | NA | NA | NA | NA | -68.8 | 0 | 9 | 53 | 36 | 1 | 0 |
| 4/11/2010 | NA | NA | NA | NA | -66.9 | 0 | 11 | 48 | 40 | 1 | 0 |
| 4/12/2010 | NA | NA | 75 | 81 | -67.7 | 0 | 15 | 45 | 39 | 1 | 0 |
| 4/13/2010 | NA | NA | NA | NA | -66.6 | 0 | 17 | 43 | 36 | 5 | 0 |
| 4/14/2010 | NA | NA | 75 | 81 | -66.0 | 1 | 12 | 54 | 32 | 1 | 0 |
| 4/15/2010 | NA | NA | NA | NA | -67.5 | 0 | 13 | 57 | 29 | 1 | 1 |
| 4/16/2010 | NA | NA | 75 | 81 | -67.3 | 0 | 10 | 58 | 30 | 2 | 0 |
| 4/17/2010 | NA | NA | NA | NA | -68.7 | 0 | 11 | 49 | 38 | 1 | 0 |
| 4/18/2010 | NA | NA | NA | NA | -68.8 | 0 | 11 | 59 | 29 | 0 | 0 |
| 4/19/2010 | NA | NA | 70 | 81 | -67.6 | 0 | 14 | 50 | 34 | 2 | 0 |
| 4/20/2010 | NA | NA | NA | NA | -68.4 | 0 | 14 | 46 | 36 | 4 | 0 |
| 4/21/2010 | NA | NA | 60 | 50 | -66.7 | 0 | 17 | 36 | 45 | 2 | 0 |
| 4/22/2010 | NA | NA | NA | NA | -67.6 | 0 | 17 | 43 | 38 | 2 | 0 |
| 4/23/2010 | NA | NA | 60 | 50 | -67.7 | 0 | 16 | 40 | 41 | 2 | 0 |
| 4/24/2010 | NA | NA | NA | NA | -68.6 | 0 | 13 | 43 | 42 | 1 | 0 |
| 4/25/2010 | NA | NA | NA | NA | -67.8 | 0 | 16 | 39 | 43 | 1 | 0 |
| 4/26/2010 | NA | NA | 70 | 50 | -67.2 | 0 | 19 | 34 | 43 | 4 | 0 |
| 4/27/2010 | NA | NA | NA | NA | -67.1 | 2 | 16 | 34 | 46 | 3 | 0 |
| 4/28/2010 | NA | NA | 70 | 50 | -62.9 | 0 | 16 | 35 | 44 | 3 | 1 |
| 4/29/2010 | NA | NA | NA | NA | -63.7 | 0 | 13 | 48 | 36 | 1 | 1 |
| 4/30/2010 | NA | NA | 75 | 50 | -65.9 | 0 | 14 | 54 | 29 | 2 | 0 |
| 5/1/2010 | NA | NA | NA | NA | -68.2 | 0 | 11 | 52 | 35 | 1 | 0 |
| 5/2/2010 | NA | NA | NA | NA | -66.7 | 0 | 11 | 55 | 33 | 1 | 0 |
| 5/3/2010 | NA | NA | 75 | 50 | -68.5 | 0 | 15 | 49 | 35 | 2 | 0 |
| 5/4/2010 | NA | NA | NA | NA | -68.4 | 0 | 15 | 36 | 44 | 2 | 0 |
| 5/5/2010 | NA | NA | 60 | 50 | -68.5 | 0 | 16 | 43 | 39 | 1 | 0 |
| 5/6/2010 | NA | NA | NA | NA | -62.1 | 0 | 13 | 55 | 28 | 1 | 1 |
| 5/7/2010 | NA | NA | 60 | 50 | -65.6 | 0 | 11 | 58 | 31 | 0 | 0 |
| 5/8/2010 | NA | NA | NA | NA | -69.7 | 0 | 9 | 60 | 30 | 1 | 0 |
| 5/9/2010 | NA | NA | NA | NA | -67.8 | 0 | 8 | 64 | 27 | 0 | 0 |
| 5/10/2010 | NA | NA | 70 | 30 | -71.0 | 0 | 9 | 66 | 25 | 0 | 0 |
| 5/11/2010 | NA | NA | NA | NA | -69.8 | 0 | 11 | 51 | 31 | 0 | 2 |
| 5/12/2010 | NA | NA | 70 | 30 | -69.8 | 0 | 10 | 60 | 27 | 1 | 1 |
| 5/13/2010 | NA | NA | NA | NA | -68.4 | 0 | 11 | 56 | 31 | 2 | 0 |
| 5/14/2010 | NA | NA | 70 | 30 | -64.3 | 1 | 9 | 53 | 34 | 2 | 1 |
| 5/15/2010 | NA | NA | NA | NA | -65.3 | 0 | 11 | 61 | 27 | 1 | 0 |
| 5/16/2010 | NA | NA | NA | NA | -68.4 | 0 | 9 | 55 | 35 | 1 | 0 |
| 5/17/2010 | NA | NA | 70 | 30 | -68.5 | 0 | 8 | 58 | 33 | 1 | 0 |
| 5/18/2010 | NA | NA | NA | NA | -69.0 | 0 | 9 | 58 | 31 | 2 | 0 |
| 5/19/2010 | NA | NA | 70 | 30 | -68.8 | 0 | 10 | 59 | 30 | 1 | 0 |
| 5/20/2010 | NA | NA | NA | NA | -70.1 | 0 | 10 | 58 | 32 | 1 | 0 |
| 5/21/2010 | NA | NA | 60 | 30 | -69.9 | 0 | 9 | 62 | 29 | 0 | 0 |
| 5/22/2010 | NA | NA | NA | NA | -68.5 | 0 | 12 | 63 | 25 | 1 | 0 |
| 5/23/2010 | NA | NA | NA | NA | -68.7 | 0 | 12 | 61 | 27 | 1 | 0 |
| 5/24/2010 | NA | NA | 60 | 10 | -68.2 | 0 | 15 | 63 | 20 | 1 | 1 |
| 5/25/2010 | NA | NA | NA | NA | -67.0 | 0 | 15 | 63 | 21 | 1 | 0 |
| 5/26/2010 | NA | NA | 60 | 10 | -63.6 | 0 | 13 | 57 | 30 | 0 | 0 |
| 5/27/2010 | NA | NA | NA | NA | -69.6 | 0 | 13 | 61 | 26 | 0 | 0 |
| 5/28/2010 | NA | NA | 60 | 10 | -68.1 | 0 | 13 | 64 | 23 | 0 | 0 |
| 5/29/2010 | NA | NA | NA | NA | -68.3 | 0 | 18 | 62 | 19 | 1 | 0 |
| 5/30/2010 | NA | NA | NA | NA | -66.4 | 0 | 20 | 58 | 20 | 2 | 0 |
| 5/31/2010 | NA | NA | 60 | 10 | -66.4 | 0 | 18 | 51 | 26 | 2 | 0 |
| 6/1/2010 | NA | NA | NA | NA | -66.7 | 0 | 17 | 55 | 27 | 2 | 0 |
| 6/2/2010 | NA | NA | 30 | 10 | -68.1 | 0 | 24 | 51 | 24 | 1 | 0 |
| 6/3/2010 | NA | NA | NA | NA | -67.0 | 0 | 34 | 43 | 22 | 1 | 0 |
| 6/4/2010 | NA | NA | 40 | 10 | -66.1 | 0 | 35 | 45 | 20 | 1 | 0 |
| 6/5/2010 | NA | NA | NA | NA | -60.9 | 0 | 50 | 40 | 9 | 1 | 0 |
| 6/6/2010 | NA | NA | NA | NA | -66.0 | 1 | 60 | 34 | 6 | 0 | 0 |
| 6/7/2010 | NA | NA | 0 | 0 | -68.6 | 0 | 45 | 40 | 15 | 1 | 0 |
| 6/8/2010 | NA | NA | NA | NA | -68.9 | 0 | 24 | 60 | 16 | 0 | 0 |
| 6/9/2010 | NA | NA | 0 | 0 | -69.8 | 0 | 14 | 69 | 17 | 1 | 0 |
| 6/10/2010 | NA | NA | NA | NA | -69.4 | 0 | 12 | 64 | 22 | 3 | 0 |
| 6/11/2010 | NA | NA | 0 | 0 | -69.7 | 0 | 13 | 68 | 17 | 2 | 0 |
| 6/12/2010 | NA | NA | 0 | 0 | -70.3 | 0 | 11 | 70 | 19 | 1 | 0 |
| 6/13/2010 | NA | NA | 0 | 0 | -69.4 | 0 | 14 | 57 | 23 | 2 | 0 |
| 6/14/2010 | NA | NA | 0 | 0 | -67.9 | 0 | 19 | 47 | 32 | 3 | 0 |
| 6/15/2010 | NA | NA | 0 | 0 | -68.5 | 0 | 18 | 45 | 34 | 2 | 0 |
| 6/16/2010 | NA | NA | 0 | 0 | -68.9 | 0 | 18 | 56 | 26 | 1 | 0 |
| 6/17/2010 | NA | NA | 0 | 0 | -69.2 | 0 | 14 | 59 | 22 | 2 | 0 |
| 6/18/2010 | NA | NA | 0 | 0 | -69.2 | 0 | 14 | 62 | 22 | 3 | 0 |
| 6/19/2010 | NA | NA | 0 | 0 | -67.6 | 0 | 14 | 59 | 24 | 3 | 0 |
| 6/20/2010 | NA | NA | 0 | 0 | -68.3 | 0 | 12 | 52 | 26 | 4 | 1 |
| 6/21/2010 | NA | NA | 0 | 0 | -68.2 | 0 | 11 | 55 | 26 | 4 | 0 |
| 6/22/2010 | NA | NA | 0 | 0 | -69.1 | 0 | 13 | 51 | 34 | 1 | 0 |
| 6/23/2010 | NA | NA | 0 | 0 | -69.1 | 0 | 15 | 48 | 36 | 0 | 0 |
| 6/24/2010 | NA | NA | 0 | 0 | -69.2 | 0 | 16 | 56 | 27 | 1 | 0 |
| 6/25/2010 | NA | NA | 0 | 0 | -69.6 | 0 | 19 | 46 | 33 | 1 | 0 |
| 6/26/2010 | NA | NA | 0 | 0 | -69.1 | 0 | 15 | 54 | 26 | 1 | 1 |
| 6/27/2010 | NA | NA | 0 | 0 | -69.7 | 0 | 18 | 59 | 23 | 0 | 0 |
| 6/28/2010 | NA | NA | 0 | 0 | -68.5 | 0 | 14 | 52 | 28 | 1 | 0 |
| 6/29/2010 | NA | NA | 0 | 0 | -68.8 | 0 | 12 | 73 | 14 | 1 | 0 |
| 6/30/2010 | NA | NA | 0 | 0 | -70.9 | 0 | 12 | 68 | 19 | 0 | 0 |
| 7/1/2010 | NA | NA | 0 | 0 | -69.8 | 0 | 15 | 60 | 24 | 1 | 0 |
| 7/2/2010 | NA | NA | 0 | 0 | -71.2 | 0 | 11 | 80 | 9 | 0 | 0 |
| 7/3/2010 | NA | NA | 0 | 0 | -71.5 | 0 | 8 | 73 | 18 | 0 | 0 |
| 7/4/2010 | NA | NA | 0 | 0 | -70.9 | 0 | 9 | 76 | 14 | 0 | 0 |
| 7/5/2010 | NA | NA | 0 | 0 | -70.9 | 0 | 14 | 73 | 13 | 1 | 0 |
| 7/6/2010 | NA | NA | 0 | 0 | -70.2 | 0 | 14 | 76 | 10 | 0 | 0 |
| 7/7/2010 | NA | NA | 0 | 0 | -70.2 | 0 | 13 | 75 | 12 | 0 | 0 |
| 7/8/2010 | NA | NA | 0 | 0 | -65.3 | 0 | 13 | 64 | 22 | 1 | 0 |
| 7/9/2010 | NA | NA | 0 | 0 | -70.8 | 0 | 11 | 70 | 17 | 0 | 0 |
| 7/10/2010 | NA | NA | 0 | 0 | -70.4 | 0 | 12 | 70 | 17 | 1 | 0 |
| 7/11/2010 | NA | NA | 0 | 0 | -70.9 | 0 | 9 | 77 | 14 | 0 | 0 |
| 7/12/2010 | NA | NA | 0 | 0 | -70.7 | 0 | 10 | 66 | 24 | 1 | 0 |
| 7/13/2010 | NA | NA | 0 | 0 | -70.5 | 0 | 13 | 65 | 22 | 1 | 0 |
| 7/14/2010 | NA | NA | 0 | 0 | -70.5 | 0 | 13 | 66 | 21 | 0 | 0 |
| 7/15/2010 | NA | NA | 0 | 0 | -70.7 | 0 | 10 | 70 | 20 | 0 | 0 |
| 7/16/2010 | NA | NA | 0 | 0 | -70.8 | 0 | 12 | 64 | 24 | 0 | 0 |
| 7/17/2010 | NA | NA | 0 | 0 | -70.7 | 0 | 12 | 62 | 27 | 0 | 0 |
| 7/18/2010 | NA | NA | 0 | 0 | -70.8 | 0 | 10 | 67 | 23 | 0 | 0 |
| 7/19/2010 | NA | NA | 0 | 0 | -70.9 | 0 | 9 | 68 | 23 | 0 | 0 |
| 7/20/2010 | NA | NA | 0 | 0 | -71.1 | 0 | 9 | 70 | 20 | 0 | 0 |
| 7/21/2010 | NA | NA | 0 | 0 | -70.9 | 0 | 12 | 60 | 28 | 0 | 0 |
| 7/22/2010 | NA | NA | 0 | 0 | -70.1 | 0 | 12 | 46 | 35 | 3 | 0 |
| 7/23/2010 | NA | NA | 0 | 0 | -69.3 | 0 | 14 | 56 | 30 | 1 | 0 |
| 7/24/2010 | NA | NA | 0 | 0 | -69.7 | 0 | 18 | 53 | 29 | 1 | 0 |
| 7/25/2010 | NA | NA | 0 | 0 | -69.7 | 0 | 12 | 61 | 27 | 0 | 0 |
| 7/26/2010 | NA | NA | 0 | 0 | -69.9 | 0 | 12 | 58 | 29 | 0 | 0 |
| 7/27/2010 | NA | NA | 0 | 0 | -69.8 | 0 | 11 | 51 | 38 | 0 | 0 |
| 7/28/2010 | NA | NA | 0 | 0 | -70.3 | 0 | 14 | 55 | 30 | 2 | 0 |
| 7/29/2010 | NA | NA | 0 | 0 | -69.2 | 0 | 11 | 56 | 33 | 1 | 0 |
| 7/30/2010 | NA | NA | 0 | 0 | -69.6 | 0 | 17 | 60 | 23 | 1 | 0 |
| 7/31/2010 | NA | NA | 0 | 0 | -69.5 | 0 | 12 | 64 | 22 | 1 | 0 |
| 8/1/2010 | NA | NA | 0 | 0 | -69.6 | 0 | 13 | 64 | 21 | 2 | 0 |
| 8/2/2010 | NA | NA | 0 | 0 | -68.5 | 0 | 17 | 57 | 23 | 2 | 0 |
| 8/3/2010 | NA | NA | 0 | 0 | -68.7 | 0 | 17 | 59 | 23 | 0 | 0 |
| 8/4/2010 | NA | NA | 0 | 0 | -69.6 | 0 | 15 | 57 | 25 | 2 | 0 |
| 8/5/2010 | NA | NA | 0 | 0 | -68.9 | 0 | 17 | 52 | 31 | 1 | 0 |
| 8/6/2010 | NA | NA | 0 | 0 | -68.9 | 0 | 13 | 57 | 25 | 1 | 0 |
| 8/7/2010 | NA | NA | 0 | 0 | -69.5 | 0 | 14 | 62 | 18 | 2 | 0 |
| 8/8/2010 | NA | NA | 0 | 0 | -68.8 | 0 | 14 | 63 | 21 | 1 | 1 |
| 8/9/2010 | NA | NA | 0 | 0 | -68.9 | 0 | 14 | 61 | 24 | 1 | 0 |
| 8/10/2010 | NA | NA | 0 | 0 | -69.1 | 0 | 12 | 67 | 21 | 1 | 0 |
| 8/11/2010 | NA | NA | 0 | 0 | -69.1 | 0 | 15 | 65 | 18 | 3 | 0 |
| 8/12/2010 | NA | NA | 0 | 0 | -69.1 | 0 | 15 | 58 | 27 | 1 | 0 |
| 8/13/2010 | NA | NA | 0 | 0 | -68.9 | 0 | 13 | 59 | 27 | 1 | 0 |
| 8/14/2010 | NA | NA | 0 | 0 | -68.6 | 0 | 15 | 51 | 26 | 2 | 0 |
| 8/15/2010 | NA | NA | 0 | 0 | -67.7 | 0 | 14 | 45 | 30 | 3 | 1 |
| 8/16/2010 | NA | NA | 0 | 0 | -68.6 | 0 | 16 | 49 | 35 | 1 | 0 |
| 8/17/2010 | NA | NA | 0 | 0 | -68.0 | 0 | 15 | 48 | 32 | 2 | 0 |
| 8/18/2010 | NA | NA | 0 | 0 | -68.3 | 0 | 19 | 60 | 22 | 0 | 0 |
| 8/19/2010 | NA | NA | 0 | 0 | -68.2 | 0 | 18 | 51 | 27 | 2 | 0 |
| 8/20/2010 | NA | NA | 0 | 0 | -67.2 | 0 | 11 | 47 | 32 | 2 | 1 |
| 8/21/2010 | NA | NA | 0 | 0 | -67.5 | 0 | 19 | 50 | 29 | 1 | 0 |
| 8/22/2010 | NA | NA | 0 | 0 | -68.3 | 0 | 10 | 70 | 20 | 0 | 0 |
| 8/23/2010 | NA | NA | 0 | 0 | -69.7 | 0 | 16 | 61 | 21 | 1 | 0 |
| 8/24/2010 | NA | NA | 0 | 0 | -67.8 | 0 | 8 | 55 | 26 | 2 | 0 |
| 8/25/2010 | NA | NA | 0 | 0 | -68.0 | 0 | 9 | 55 | 26 | 2 | 0 |
| 8/26/2010 | NA | NA | 0 | 0 | -68.9 | 0 | 14 | 55 | 28 | 2 | 0 |
| 8/27/2010 | NA | NA | 0 | 0 | -68.4 | 0 | 15 | 55 | 26 | 3 | 0 |
| 8/28/2010 | NA | NA | 0 | 0 | -68.5 | 0 | 19 | 54 | 26 | 2 | 0 |
| 8/29/2010 | NA | NA | 0 | 0 | -67.7 | 0 | 14 | 57 | 28 | 1 | 0 |
| 8/30/2010 | NA | NA | 0 | 0 | -68.6 | 0 | 13 | 55 | 30 | 2 | 0 |
| 8/31/2010 | NA | NA | 0 | 0 | -68.6 | 0 | 15 | 52 | 29 | 2 | 0 |
| 9/1/2010 | NA | NA | 0 | 0 | -68.3 | 0 | 14 | 53 | 27 | 4 | 0 |
| 9/2/2010 | NA | NA | 0 | 0 | -67.0 | 0 | 13 | 50 | 30 | 3 | 1 |
| 9/3/2010 | NA | NA | 0 | 0 | -67.9 | 0 | 13 | 53 | 29 | 2 | 0 |
| 9/4/2010 | NA | NA | 0 | 0 | -68.5 | 0 | 17 | 55 | 28 | 1 | 0 |
| 9/5/2010 | NA | NA | 0 | 0 | -68.5 | 0 | 13 | 52 | 30 | 2 | 0 |
| 9/6/2010 | NA | NA | 0 | 0 | -66.5 | 0 | 12 | 46 | 31 | 2 | 1 |
| 9/7/2010 | NA | NA | 0 | 0 | -69.0 | 0 | 9 | 48 | 33 | 1 | 0 |
| 9/8/2010 | NA | NA | 0 | 0 | -69.1 | 0 | 13 | 46 | 35 | 2 | 0 |
| 9/9/2010 | NA | NA | 0 | 0 | -68.3 | 0 | 15 | 52 | 32 | 1 | 0 |
| 9/10/2010 | NA | NA | 0 | 0 | -68.4 | 0 | 16 | 49 | 33 | 1 | 0 |
| 9/11/2010 | NA | NA | 0 | 0 | -68.5 | 0 | 14 | 49 | 32 | 1 | 0 |
| 9/12/2010 | NA | NA | 0 | 0 | -67.2 | 0 | 11 | 42 | 29 | 5 | 1 |
| 9/13/2010 | NA | NA | 0 | 0 | -68.7 | 0 | 17 | 47 | 33 | 2 | 0 |
| 9/14/2010 | NA | NA | 0 | 0 | -67.5 | 0 | 15 | 46 | 37 | 2 | 0 |
| 9/15/2010 | NA | NA | 0 | 0 | -68.2 | 0 | 16 | 47 | 36 | 1 | 0 |
| 9/16/2010 | NA | NA | 0 | 0 | -67.7 | 0 | 16 | 43 | 40 | 2 | 0 |
| 9/17/2010 | NA | NA | 0 | 0 | -67.7 | 0 | 17 | 35 | 45 | 2 | 0 |
| 9/18/2010 | NA | NA | 0 | 0 | -66.5 | 0 | 12 | 38 | 42 | 2 | 0 |
| 9/19/2010 | NA | NA | 0 | 0 | -68.1 | 0 | 10 | 43 | 35 | 3 | 0 |
| 9/20/2010 | NA | NA | 0 | 0 | -67.2 | 0 | 13 | 40 | 39 | 1 | 0 |
| 9/21/2010 | NA | NA | 0 | 0 | -63.7 | 0 | 8 | 43 | 39 | 2 | 0 |
| 9/22/2010 | NA | NA | 0 | 0 | -63.5 | 0 | 15 | 43 | 41 | 1 | 0 |
| 9/23/2010 | NA | NA | 0 | 0 | -68.3 | 0 | 14 | 42 | 41 | 4 | 0 |
| 9/24/2010 | NA | NA | 0 | 0 | -66.7 | 0 | 12 | 41 | 38 | 5 | 0 |
| 9/25/2010 | NA | NA | 0 | 0 | -66.7 | 0 | 9 | 41 | 38 | 3 | 1 |
| 9/26/2010 | NA | NA | 0 | 0 | -61.4 | 0 | 6 | 39 | 41 | 2 | 2 |
| 9/27/2010 | NA | NA | 0 | 0 | -68.0 | 0 | 0 | 57 | 35 | 0 | 2 |
| 9/28/2010 | 0 | 0 | 0 | 0 | -66.9 | 0 | 0 | 67 | 33 | 0 | 0 |
| 9/29/2010 | 0 | 0 | 0 | 0 | -67.4 | 0 | 0 | 66 | 32 | 0 | 1 |
| 9/30/2010 | 0 | 0 | 0 | 0 | -70.1 | 0 | 0 | 63 | 35 | 0 | 1 |
| 10/1/2010 | 0 | 0 | 0 | 0 | -70.8 | 0 | 0 | 60 | 39 | 0 | 0 |
| 10/2/2010 | 0 | 0 | 0 | 0 | -66.1 | 0 | 0 | 58 | 42 | 0 | 0 |
| 10/3/2010 | 0 | 0 | 0 | 0 | -69.5 | 0 | 1 | 73 | 27 | 0 | 0 |
| 10/4/2010 | 0 | 0 | 0 | 0 | -73.4 | 0 | 1 | 74 | 25 | 0 | 0 |
| 10/5/2010 | 0 | 0 | 0 | 0 | -74.1 | 0 | 0 | 77 | 23 | 0 | 0 |
| 10/6/2010 | 0 | 0 | 0 | 0 | -74.0 | 0 | 0 | 80 | 21 | 0 | 0 |
| 10/7/2010 | 0 | 0 | 0 | 0 | -74.6 | 0 | 1 | 88 | 9 | 0 | 0 |
| 10/8/2010 | 0 | 0 | 0 | 0 | -76.9 | 0 | 0 | 90 | 10 | 0 | 0 |
| 10/9/2010 | 0 | 0 | 0 | 0 | -75.4 | 0 | 0 | 73 | 26 | 0 | 0 |
| 10/10/2010 | 0 | 0 | 0 | 0 | -70.8 | 0 | 0 | 69 | 31 | 0 | 0 |
| 10/11/2010 | 0 | 0 | 0 | 0 | -69.2 | 0 | 1 | 68 | 29 | 0 | 0 |
| 10/12/2010 | 0 | 0 | 0 | 0 | -74.6 | 0 | 0 | 81 | 19 | 0 | 0 |
| 10/13/2010 | 0 | 0 | 0 | 0 | -75.1 | 0 | 1 | 74 | 17 | 0 | 1 |
| 10/14/2010 | 0 | 0 | 0 | 0 | -74.0 | 0 | 1 | 72 | 26 | 0 | 0 |
| 10/15/2010 | 0 | 0 | 0 | 0 | -73.2 | 0 | 0 | 67 | 33 | 0 | 0 |
| 10/16/2010 | 0 | 0 | 0 | 0 | -68.3 | 0 | 0 | 45 | 51 | 0 | 0 |
| 10/17/2010 | 0 | 0 | 0 | 0 | -66.1 | 0 | 0 | 56 | 44 | 0 | 0 |
| 10/18/2010 | 0 | 0 | 0 | 0 | -67.6 | 0 | 0 | 52 | 44 | 0 | 0 |
| 10/19/2010 | 0 | 0 | 0 | 0 | -72.1 | 0 | 0 | 75 | 25 | 0 | 0 |
| 10/20/2010 | 0 | 0 | 0 | 0 | -71.8 | 0 | 0 | 53 | 45 | 0 | 0 |
| 10/21/2010 | 0 | 0 | 0 | 0 | -68.0 | 0 | 0 | 55 | 43 | 0 | 0 |
| 10/22/2010 | 0 | 0 | 0 | 0 | -67.8 | 0 | 0 | 60 | 38 | 0 | 0 |
| 10/23/2010 | 0 | 0 | 0 | 0 | -70.5 | 0 | 0 | 65 | 35 | 0 | 0 |
| 10/24/2010 | 0 | 0 | 0 | 0 | -72.5 | 0 | 0 | 69 | 31 | 0 | 0 |
| 10/25/2010 | 0 | 0 | 0 | 0 | -70.2 | 0 | 0 | 59 | 39 | 2 | 0 |
| 10/26/2010 | 0 | 0 | 0 | 0 | -71.2 | 0 | 0 | 57 | 43 | 0 | 0 |
| 10/27/2010 | 0 | 0 | 0 | 0 | -64.8 | 0 | 0 | 40 | 60 | 0 | 0 |
| 10/28/2010 | 0 | 0 | 0 | 0 | -65.7 | 0 | 0 | 63 | 37 | 0 | 0 |
| 10/29/2010 | 0 | 0 | 0 | 0 | -69.6 | 0 | 0 | 67 | 30 | 0 | 0 |
| 10/30/2010 | 0 | 0 | 0 | 0 | -71.0 | 0 | 0 | 70 | 27 | 0 | 0 |
| 10/31/2010 | 0 | 0 | 0 | 0 | -69.6 | 0 | 0 | 58 | 34 | 0 | 0 |
| 11/1/2010 | 0 | 0 | 0 | 0 | -70.7 | 0 | 0 | 56 | 40 | 0 | 0 |
| 11/2/2010 | 0 | 0 | 0 | 0 | -67.3 | 0 | 0 | 71 | 29 | 0 | 0 |
| 11/3/2010 | 0 | 0 | 0 | 0 | -71.2 | 0 | 0 | 77 | 23 | 0 | 0 |
| 11/4/2010 | 0 | 0 | 0 | 0 | -73.2 | 0 | 0 | 75 | 25 | 0 | 0 |
| 11/5/2010 | 0 | 0 | 0 | 0 | -69.5 | 0 | 0 | 65 | 35 | 0 | 0 |
| 11/6/2010 | 0 | 0 | 0 | 0 | -67.7 | 0 | 1 | 63 | 36 | 0 | 0 |
| 11/7/2010 | 0 | 0 | 0 | 0 | -71.6 | 0 | 0 | 72 | 28 | 0 | 0 |
| 11/8/2010 | 0 | 0 | 0 | 0 | -72.8 | 0 | 0 | 70 | 30 | 0 | 0 |
| 11/9/2010 | 0 | 0 | 0 | 0 | -70.3 | 0 | 0 | 75 | 26 | 0 | 0 |
| 11/10/2010 | 0 | 0 | 0 | 0 | -69.5 | 0 | 0 | 76 | 24 | 0 | 0 |
| 11/11/2010 | 0 | 0 | 0 | 0 | -69.1 | 0 | 0 | 57 | 39 | 0 | 1 |
| 11/12/2010 | 0 | 0 | 0 | 0 | -67.0 | 0 | 0 | 58 | 39 | 0 | 0 |
| 11/13/2010 | 0 | 0 | 0 | 0 | -69.6 | 0 | 0 | 67 | 33 | 0 | 0 |
| 11/14/2010 | 0 | 0 | 0 | 0 | -73.1 | 0 | 0 | 70 | 30 | 0 | 0 |
| 11/15/2010 | 0 | 0 | 0 | 0 | -74.0 | 0 | 0 | 68 | 32 | 0 | 0 |
| 11/16/2010 | 0 | 0 | 0 | 0 | -70.0 | 0 | 0 | 52 | 47 | 0 | 1 |
| 11/17/2010 | 0 | 0 | 0 | 0 | -66.4 | 0 | 0 | 52 | 48 | 0 | 0 |
| 11/18/2010 | 0 | 0 | 0 | 0 | -67.4 | 0 | 0 | 53 | 47 | 0 | 0 |
| 11/19/2010 | 0 | 0 | 0 | 0 | -68.1 | 0 | 0 | 53 | 45 | 2 | 0 |
| 11/20/2010 | 0 | 0 | 0 | 0 | -67.7 | 0 | 0 | 57 | 43 | 0 | 0 |
| 11/21/2010 | 0 | 0 | 0 | 0 | -67.3 | 0 | 2 | 63 | 35 | 0 | 0 |
| 11/22/2010 | 0 | 0 | 0 | 0 | -68.1 | 0 | 1 | 53 | 45 | 0 | 0 |
| 11/23/2010 | 0 | 0 | 0 | 0 | -64.5 | 0 | 0 | 43 | 51 | 0 | 0 |
| 11/24/2010 | 0 | 0 | 0 | 0 | -66.7 | 0 | 2 | 55 | 43 | 0 | 0 |
| 11/25/2010 | 0 | 0 | 0 | 0 | -70.1 | 0 | 0 | 51 | 47 | 0 | 0 |
| 11/26/2010 | 0 | 0 | 0 | 0 | -69.3 | 0 | 0 | 45 | 55 | 0 | 0 |
| 11/27/2010 | 0 | 0 | 0 | 0 | -69.0 | 0 | 2 | 48 | 50 | 0 | 0 |
| 11/28/2010 | 0 | 0 | 0 | 0 | -71.2 | 0 | 2 | 54 | 41 | 0 | 0 |
| 11/29/2010 | 0 | 0 | 0 | 0 | -66.9 | 0 | 0 | 42 | 56 | 0 | 0 |
| 11/30/2010 | 0 | 0 | 0 | 0 | -68.3 | 0 | 2 | 52 | 44 | 0 | 0 |
| 12/1/2010 | 0 | 0 | 0 | 0 | -71.4 | 0 | 1 | 55 | 45 | 0 | 0 |
| 12/2/2010 | 0 | 0 | 0 | 0 | -68.7 | 0 | 0 | 53 | 47 | 0 | 0 |
| 12/3/2010 | 0 | 0 | 0 | 0 | -70.0 | 0 | 6 | 59 | 31 | 0 | 0 |
| 12/4/2010 | 0 | 0 | 0 | 0 | -65.9 | 0 | 58 | 28 | 9 | 0 | 0 |
| 12/5/2010 | 0 | 0 | 0 | 0 | -66.2 | 0 | 29 | 59 | 12 | 0 | 0 |
| 12/6/2010 | 0 | 0 | 0 | 0 | -72.5 | 0 | 2 | 67 | 31 | 0 | 0 |
| 12/7/2010 | 0 | 0 | 0 | 0 | -71.4 | 0 | 1 | 64 | 35 | 0 | 0 |
| 12/8/2010 | 0 | 0 | 0 | 0 | -68.7 | 0 | 0 | 76 | 24 | 0 | 0 |
| 12/9/2010 | 0 | 0 | 0 | 0 | -72.1 | 0 | 1 | 61 | 32 | 0 | 0 |
| 12/10/2010 | 0 | 0 | 0 | 0 | -71.1 | 0 | 1 | 62 | 37 | 0 | 0 |
| 12/11/2010 | 0 | 0 | 0 | 0 | -71.6 | 0 | 0 | 47 | 53 | 0 | 0 |
| 12/12/2010 | 0 | 0 | 0 | 0 | -70.1 | 0 | 0 | 53 | 47 | 0 | 0 |
| 12/13/2010 | 0 | 0 | 0 | 0 | -69.5 | 0 | 0 | 53 | 46 | 0 | 0 |
| 12/14/2010 | 0 | 0 | 0 | 0 | -71.1 | 0 | 0 | 63 | 38 | 0 | 0 |
| 12/15/2010 | 0 | 0 | 0 | 0 | -72.1 | 0 | 2 | 74 | 23 | 0 | 0 |
| 12/16/2010 | 0 | 0 | 0 | 0 | -74.3 | 0 | 1 | 78 | 21 | 0 | 0 |
| 12/17/2010 | 0 | 0 | 0 | 0 | -74.8 | 0 | 0 | 83 | 17 | 0 | 0 |
| 12/18/2010 | 0 | 0 | 0 | 0 | -72.2 | 0 | 0 | 69 | 31 | 0 | 0 |
| 12/19/2010 | 0 | 0 | 0 | 0 | -69.9 | 0 | 0 | 70 | 28 | 1 | 0 |
| 12/20/2010 | 0 | 0 | 0 | 0 | -73.0 | 0 | 0 | 62 | 37 | 0 | 0 |
| 12/21/2010 | 0 | 0 | 0 | 0 | -68.8 | 0 | 1 | 67 | 31 | 0 | 0 |
| 12/22/2010 | 0 | 0 | 0 | 0 | -70.4 | 0 | 1 | 76 | 23 | 0 | 0 |
| 12/23/2010 | 0 | 0 | 0 | 0 | -70.4 | 0 | 0 | 80 | 20 | 0 | 0 |
| 12/24/2010 | 0 | 0 | 0 | 0 | -67.7 | 0 | 0 | 65 | 32 | 0 | 0 |
| 12/25/2010 | 0 | 0 | 0 | 0 | -66.8 | 0 | 0 | 63 | 29 | 1 | 0 |
| 12/26/2010 | 0 | 0 | 0 | 0 | -66.3 | 0 | 29 | 54 | 11 | 0 | 0 |
| 12/27/2010 | 0 | 0 | 0 | 0 | -67.4 | 0 | 3 | 86 | 11 | 0 | 0 |
| 12/28/2010 | 0 | 0 | 0 | 0 | -69.6 | 0 | 0 | 83 | 17 | 0 | 0 |
| 12/29/2010 | 0 | 0 | 0 | 0 | -67.4 | 0 | 0 | 74 | 24 | 0 | 0 |
| 12/30/2010 | 0 | 0 | 0 | 0 | -66.7 | 0 | 1 | 85 | 15 | 0 | 0 |
| 12/31/2010 | 0 | 0 | 0 | 0 | -74.3 | 0 | 1 | 78 | 17 | 0 | 0 |
| 1/1/2011 | 0 | 0 | 0 | 0 | -68.4 | 0 | 0 | 74 | 22 | 0 | 0 |
| 1/2/2011 | 0 | 0 | 0 | 0 | -69.4 | 0 | 1 | 67 | 30 | 0 | 0 |
| 1/3/2011 | 0 | 0 | 0 | 0 | -71.3 | 0 | 0 | 74 | 25 | 0 | 0 |
| 1/4/2011 | 0 | 0 | NA | NA | -69.3 | 0 | 1 | 81 | 19 | 0 | 0 |
| 1/5/2011 | 0 | 0 | 30 | 15 | -73.8 | 0 | 1 | 83 | 16 | 0 | 0 |
| 1/6/2011 | 0 | 0 | NA | NA | -71.1 | 0 | 0 | 61 | 35 | 0 | 0 |
| 1/7/2011 | 0 | 0 | 0 | 0 | -66.7 | 0 | 0 | 59 | 39 | 0 | 0 |
| 1/8/2011 | 0 | 0 | NA | NA | -66.7 | 0 | 0 | 36 | 62 | 0 | 0 |
| 1/9/2011 | 0 | 0 | NA | NA | -66.2 | 0 | 0 | 50 | 48 | 0 | 3 |
| 1/10/2011 | 0 | 0 | 0 | 0 | -67.8 | 0 | 0 | 64 | 36 | 0 | 0 |
| 1/11/2011 | 0 | 0 | NA | NA | -69.0 | 0 | 0 | 72 | 27 | 0 | 0 |
| 1/12/2011 | 0 | 0 | 0 | 0 | -71.9 | 0 | 0 | 64 | 36 | 0 | 0 |
| 1/13/2011 | 0 | 0 | NA | NA | -67.7 | 0 | 0 | 64 | 34 | 1 | 0 |
| 1/14/2011 | 0 | 0 | 20 | 5 | -66.7 | 0 | 0 | 65 | 35 | 0 | 0 |
| 1/15/2011 | 0 | 0 | NA | NA | -70.9 | 0 | 1 | 67 | 31 | 0 | 1 |
| 1/16/2011 | 0 | 0 | NA | NA | -71.6 | 0 | 0 | 67 | 33 | 0 | 0 |
| 1/17/2011 | 0 | 0 | 30 | 5 | -74.7 | 0 | 0 | 74 | 24 | 0 | 1 |
| 1/18/2011 | 0 | 0 | NA | NA | -69.9 | 0 | 0 | 73 | 26 | 0 | 0 |
| 1/19/2011 | 0 | 0 | 60 | 30 | -67.9 | 0 | 0 | 80 | 19 | 0 | 0 |
| 1/20/2011 | 0 | 0 | NA | NA | -70.4 | 0 | 1 | 82 | 16 | 0 | 1 |
| 1/21/2011 | 0 | 0 | 70 | 30 | -72.6 | 0 | 0 | 82 | 18 | 0 | 0 |
| 1/22/2011 | 0 | 0 | NA | NA | -74.0 | 0 | 1 | 84 | 15 | 0 | 0 |
| 1/23/2011 | 0 | 0 | NA | NA | -69.4 | 0 | 5 | 83 | 11 | 0 | 0 |
| 1/24/2011 | 0 | 0 | 80 | 41 | -72.7 | 0 | 2 | 95 | 2 | 0 | 0 |
| 1/25/2011 | 1 | 0 | NA | NA | -72.8 | 0 | 1 | 82 | 4 | 0 | 0 |
| 1/26/2011 | 0 | 0 | 85 | 41 | -71.2 | 0 | 1 | 92 | 2 | 0 | 1 |
| 1/27/2011 | 1 | 0 | NA | NA | -72.4 | 0 | 1 | 89 | 6 | 0 | 1 |
| 1/28/2011 | 0 | 0 | 85 | 41 | -70.6 | 0 | 2 | 95 | 3 | 0 | 1 |
| 1/29/2011 | 0 | 0 | NA | NA | -75.6 | 0 | 1 | 86 | 13 | 0 | 0 |
| 1/30/2011 | 0 | 0 | NA | NA | -75.3 | 0 | 1 | 87 | 12 | 0 | 0 |
| 1/31/2011 | 0 | 0 | 70 | 41 | -75.0 | 0 | 2 | 84 | 13 | 0 | 0 |
| 2/1/2011 | 1 | 0 | NA | NA | -73.0 | 0 | 3 | 86 | 11 | 0 | 0 |
| 2/2/2011 | 0 | 0 | 70 | 41 | -75.2 | 0 | 0 | 89 | 10 | 0 | 0 |
| 2/3/2011 | 1 | 0 | NA | NA | -74.5 | 0 | 2 | 92 | 5 | 0 | 0 |
| 2/4/2011 | 0 | 0 | 75 | 41 | -78.4 | 0 | 0 | 93 | 6 | 0 | 0 |
| 2/5/2011 | 0 | 0 | NA | NA | -77.3 | 0 | 1 | 92 | 7 | 0 | 0 |
| 2/6/2011 | 0 | 0 | NA | NA | -76.0 | 0 | 0 | 97 | 3 | 0 | 0 |
| 2/7/2011 | 0 | 0 | 70 | 36 | -79.4 | 0 | 1 | 96 | 3 | 0 | 0 |
| 2/8/2011 | 0 | 0 | NA | NA | -75.3 | 0 | 2 | 94 | 3 | 0 | 0 |
| 2/9/2011 | 0 | 0 | 80 | 41 | -75.1 | 0 | 2 | 95 | 2 | 0 | 0 |
| 2/10/2011 | 1 | 0 | NA | NA | -75.4 | 0 | 1 | 98 | 1 | 0 | 0 |
| 2/11/2011 | 0 | 0 | 80 | 41 | -71.6 | 0 | 2 | 94 | 4 | 0 | 0 |
| 2/12/2011 | 1 | 0 | NA | NA | -72.5 | 0 | 3 | 87 | 9 | 0 | 0 |
| 2/13/2011 | 0 | 0 | NA | NA | -70.7 | 0 | 2 | 93 | 4 | 0 | 0 |
| 2/14/2011 | 0 | 1 | 80 | 41 | -71.1 | 0 | 1 | 95 | 1 | 0 | 1 |
| 2/15/2011 | 0 | 0 | NA | NA | -77.1 | 0 | 0 | 93 | 6 | 0 | 0 |
| 2/16/2011 | 1 | 0 | 80 | 41 | -76.2 | 0 | 9 | 89 | 2 | 0 | 0 |
| 2/17/2011 | 0 | 1 | NA | NA | -72.5 | 0 | 38 | 62 | 0 | 0 | 0 |
| 2/18/2011 | 0 | 0 | 20 | 10 | -74.1 | 0 | 63 | 37 | 0 | 0 | 0 |
| 2/19/2011 | 1 | 0 | NA | NA | -75.1 | 0 | 12 | 88 | 1 | 0 | 0 |
| 2/20/2011 | 0 | 0 | NA | NA | -68.7 | 0 | 36 | 62 | 2 | 0 | 0 |
| 2/21/2011 | 0 | 0 | 0 | 0 | -70.7 | 0 | 1 | 99 | 0 | 0 | 1 |
| 2/22/2011 | 0 | 0 | NA | NA | -70.2 | 0 | 19 | 76 | 3 | 0 | 0 |
| 2/23/2011 | 0 | 0 | 40 | 15 | -67.9 | 0 | 1 | 74 | 19 | 1 | 0 |
| 2/24/2011 | 0 | 0 | NA | NA | -66.2 | 0 | 30 | 50 | 10 | 0 | 0 |
| 2/25/2011 | 0 | 0 | 0 | 0 | -70.4 | 0 | 1 | 97 | 3 | 0 | 0 |
| 2/26/2011 | 0 | 0 | NA | NA | -78.0 | 0 | 0 | 100 | 0 | 0 | 0 |
| 2/27/2011 | 0 | 0 | NA | NA | -78.8 | 0 | 0 | 93 | 6 | 0 | 0 |
| 2/28/2011 | 1 | 0 | 40 | 15 | -78.2 | 0 | 0 | 94 | 4 | 0 | 0 |
| 3/1/2011 | 1 | 0 | 40 | 15 | -78.3 | 0 | 0 | 92 | 8 | 0 | 0 |
| 3/2/2011 | 1 | 0 | 60 | 15 | -78.4 | 0 | 0 | 92 | 8 | 0 | 0 |
| 3/3/2011 | 1 | 0 | NA | NA | -78.1 | 0 | 0 | 89 | 9 | 0 | 0 |
| 3/4/2011 | 1 | 0 | 60 | 15 | -78.1 | 0 | 1 | 93 | 6 | 0 | 0 |
| 3/5/2011 | 1 | 0 | NA | NA | -78.5 | 0 | 0 | 95 | 5 | 0 | 0 |
| 3/6/2011 | 1 | 0 | NA | NA | -79.0 | 0 | 3 | 96 | 1 | 0 | 0 |
| 3/7/2011 | 1 | 0 | 70 | 20 | -77.1 | 0 | 3 | 94 | 2 | 0 | 0 |
| 3/8/2011 | 1 | 0 | NA | NA | -78.5 | 0 | 2 | 96 | 2 | 0 | 0 |
| 3/9/2011 | 0 | 1 | 75 | 20 | -79.0 | 0 | 2 | 98 | 1 | 0 | 0 |
| 3/10/2011 | 0 | 1 | NA | NA | -78.4 | 0 | 0 | 93 | 7 | 0 | 1 |
| 3/11/2011 | 0 | 0 | 75 | 20 | -78.1 | 0 | 1 | 95 | 4 | 0 | 0 |
| 3/12/2011 | 1 | 0 | NA | NA | -78.5 | 0 | 2 | 94 | 4 | 0 | 0 |
| 3/13/2011 | 1 | 0 | NA | NA | -78.9 | 0 | 2 | 96 | 2 | 0 | 0 |
| 3/14/2011 | 1 | 0 | 75 | 20 | -78.9 | 0 | 2 | 97 | 0 | 0 | 0 |
| 3/15/2011 | 1 | 1 | NA | NA | -79.0 | 0 | 4 | 77 | 0 | 0 | 0 |
| 3/16/2011 | 1 | 0 | 80 | 36 | -77.4 | 0 | 3 | 93 | 0 | 0 | 1 |
| 3/17/2011 | 1 | 0 | NA | NA | -76.8 | 0 | 2 | 94 | 1 | 0 | 2 |
| 3/18/2011 | 1 | 0 | 80 | 36 | -75.9 | 0 | 0 | 93 | 4 | 0 | 3 |
| 3/19/2011 | 1 | 0 | NA | NA | -76.4 | 0 | 1 | 91 | 3 | 0 | 5 |
| 3/20/2011 | 1 | 0 | NA | NA | -75.5 | 0 | 1 | 91 | 3 | 0 | 3 |
| 3/21/2011 | 1 | 1 | 75 | 36 | -75.8 | 0 | 1 | 95 | 2 | 0 | 0 |
| 3/22/2011 | 1 | 0 | NA | NA | -76.1 | 0 | 1 | 95 | 2 | 0 | 1 |
| 3/23/2011 | 1 | 1 | 80 | 41 | -75.8 | 0 | 3 | 92 | 2 | 0 | 3 |
| 3/24/2011 | 1 | 0 | NA | NA | -77.6 | 0 | 3 | 95 | 1 | 0 | 1 |
| 3/25/2011 | 1 | 0 | 80 | 41 | -77.8 | 0 | 1 | 96 | 2 | 0 | 0 |
| 3/26/2011 | 1 | 0 | NA | NA | -77.2 | 0 | 2 | 97 | 1 | 0 | 0 |
| 3/27/2011 | 1 | 0 | NA | NA | -78.6 | 0 | 1 | 97 | 1 | 0 | 0 |
| 3/28/2011 | 1 | 0 | 75 | 36 | -77.8 | 0 | 2 | 96 | 2 | 0 | 0 |
| 3/29/2011 | 1 | 0 | NA | NA | -77.4 | 0 | 2 | 92 | 4 | 0 | 0 |
| 3/30/2011 | 1 | 0 | NA | NA | -78.3 | 0 | 1 | 96 | 2 | 0 | 0 |
| 3/31/2011 | 1 | 0 | 75 | 41 | -78.1 | 0 | 3 | 93 | 2 | 0 | 1 |
| 4/1/2011 | 1 | 0 | 75 | 41 | -78.4 | 0 | 4 | 92 | 3 | 0 | 0 |
| 4/2/2011 | 1 | 0 | NA | NA | -75.1 | 0 | 3 | 94 | 2 | 0 | 0 |
| 4/3/2011 | 1 | 0 | NA | NA | -75.9 | 0 | 2 | 87 | 2 | 0 | 0 |
| 4/4/2011 | 1 | 0 | 75 | 41 | -76.3 | 0 | 3 | 95 | 2 | 0 | 0 |
| 4/5/2011 | 1 | 0 | NA | NA | -73.2 | 0 | 0 | 92 | 6 | 0 | 0 |
| 4/6/2011 | 1 | 0 | 70 | 41 | -72.7 | 0 | 3 | 92 | 4 | 0 | 0 |
| 4/7/2011 | 1 | 0 | NA | NA | -74.5 | 0 | 4 | 91 | 6 | 0 | 0 |
| 4/8/2011 | 1 | 0 | 80 | 41 | -72.2 | 0 | 8 | 91 | 1 | 0 | 0 |
| 4/9/2011 | 1 | 0 | NA | NA | -72.6 | 0 | 4 | 95 | 2 | 0 | 0 |
| 4/10/2011 | 1 | 0 | NA | NA | -75.4 | 0 | 2 | 93 | 5 | 0 | 0 |
| 4/11/2011 | 1 | 0 | 70 | 41 | -73.9 | 0 | 3 | 93 | 3 | 1 | 0 |
| 4/12/2011 | 1 | 0 | NA | NA | -73.0 | 0 | 2 | 93 | 3 | 1 | 0 |
| 4/13/2011 | 1 | 0 | 70 | 41 | -74.0 | 0 | 3 | 94 | 2 | 0 | 0 |
| 4/14/2011 | 1 | 1 | NA | NA | -72.8 | 0 | 2 | 93 | 5 | 0 | 0 |
| 4/15/2011 | 1 | 0 | 80 | 41 | -73.1 | 0 | 2 | 91 | 5 | 0 | 1 |
| 4/16/2011 | 1 | 0 | NA | NA | -75.2 | 0 | 1 | 93 | 6 | 0 | 0 |
| 4/17/2011 | 1 | 1 | NA | NA | -74.6 | 0 | 2 | 94 | 4 | 0 | 0 |
| 4/18/2011 | 1 | 0 | 80 | 51 | -76.3 | 0 | 2 | 95 | 3 | 0 | 0 |
| 4/19/2011 | 1 | 1 | NA | NA | -75.2 | 0 | 1 | 95 | 3 | 0 | 0 |
| 4/20/2011 | 1 | 0 | 80 | 51 | -75.0 | 0 | 2 | 96 | 2 | 0 | 0 |
| 4/21/2011 | 1 | 0 | NA | NA | -74.3 | 0 | 3 | 94 | 2 | 0 | 0 |
| 4/22/2011 | 1 | 0 | 75 | 51 | -73.1 | 0 | 1 | 94 | 3 | 0 | 0 |
| 4/23/2011 | 1 | 0 | NA | NA | -72.9 | 0 | 1 | 92 | 4 | 1 | 1 |
| 4/24/2011 | 1 | 0 | NA | NA | -72.7 | 0 | 1 | 96 | 3 | 0 | 0 |
| 4/25/2011 | 1 | 1 | 75 | 51 | -74.1 | 0 | 3 | 93 | 3 | 0 | 1 |
| 4/26/2011 | 1 | 1 | NA | NA | -73.4 | 0 | 2 | 94 | 3 | 0 | 1 |
| 4/27/2011 | 1 | 1 | 75 | 61 | -72.1 | 0 | 2 | 92 | 5 | 0 | 1 |
| 4/28/2011 | 1 | 1 | NA | NA | -74.1 | 0 | 2 | 94 | 3 | 1 | 1 |
| 4/29/2011 | 1 | 0 | 75 | 61 | -74.6 | 0 | 2 | 91 | 6 | 0 | 0 |
| 4/30/2011 | 1 | 1 | NA | NA | -74.3 | 0 | 2 | 91 | 5 | 1 | 0 |
| 5/1/2011 | 1 | 0 | NA | NA | -75.8 | 0 | 1 | 96 | 3 | 0 | 1 |
| 5/2/2011 | 1 | 1 | 75 | 51 | -72.2 | 0 | 1 | 95 | 3 | 1 | 0 |
| 5/3/2011 | 1 | 1 | NA | NA | -75.8 | 0 | 2 | 91 | 0 | 0 | 0 |
| 5/4/2011 | 1 | 1 | 75 | 51 | -76.4 | 0 | 2 | 96 | 1 | 0 | 0 |
| 5/5/2011 | 1 | 1 | 75 | 51 | -75.5 | 0 | 1 | 95 | 1 | 1 | 1 |
| 5/6/2011 | 1 | 1 | 75 | 51 | -75.8 | 0 | 1 | 96 | 0 | 0 | 0 |
| 5/7/2011 | 1 | 1 | NA | NA | -75.0 | 0 | 2 | 96 | 2 | 0 | 0 |
| 5/8/2011 | 1 | 1 | NA | NA | -75.1 | 0 | 1 | 96 | 1 | 1 | 1 |
| 5/9/2011 | 1 | 1 | 50 | 20 | -76.2 | 0 | 1 | 93 | 2 | 0 | 1 |
| 5/10/2011 | 1 | 1 | NA | NA | -75.3 | 0 | 2 | 97 | 1 | 0 | 0 |
| 5/11/2011 | 1 | 1 | 50 | 20 | -74.2 | 0 | 0 | 98 | 2 | 0 | 0 |
| 5/12/2011 | 1 | 1 | NA | NA | -73.6 | 0 | 0 | 93 | 4 | 0 | 1 |
| 5/13/2011 | 0 | 1 | 50 | 20 | -74.1 | 0 | 1 | 96 | 3 | 0 | 1 |
| 5/14/2011 | 0 | 1 | NA | NA | -76.8 | 0 | 2 | 96 | 1 | 0 | 0 |
| 5/15/2011 | 0 | 1 | NA | NA | -78.1 | 0 | 0 | 99 | 1 | 0 | 0 |
| 5/16/2011 | 0 | 1 | 50 | 20 | -78.8 | 0 | 1 | 98 | 0 | 0 | 0 |
| 5/17/2011 | 0 | 1 | NA | NA | -79.5 | 0 | 4 | 96 | 0 | 0 | 0 |
| 5/18/2011 | 0 | 1 | 0 | 0 | -79.1 | 0 | 1 | 93 | 6 | 0 | 0 |
| 5/19/2011 | 0 | 1 | NA | NA | -72.5 | 0 | 0 | 93 | 7 | 0 | 0 |
| 5/20/2011 | 0 | 1 | 0 | 0 | -70.8 | 0 | 0 | 74 | 6 | 0 | 0 |
