# Supplementary material for: Environmental Predictors of Ice Seal Presence in the Bering Sea
Source: PLoS One. 2014 Sep 17;9(9):e106998. doi: 10.1371/journal.pone.0106998 (PMC4167550; doi:10.1371/journal.pone.0106998)
Supplement: Table S2 — Daily mean sound levels at M2 (A) and M5 (B) used in the GLM and GAM modeling. Sound level units are dB re 1 μPa2/Hz. (DOCX) [file pone.0106998.s002.docx]

**Table S2 Daily mean sound levels at M2 (A) and M5 (B) used in the GLM and GAM modeling.** Sound level units are dB re 1μPa^2^/Hz.

A)

| **Date** | **500 Hz** | **2 kHz** | **10 kHz** | **20 kHz** | **40 kHz** |
| --- | --- | --- | --- | --- | --- |
| 9/27/2009 | 66.7 | 62.9 | 47.9 | 42.8 | 37.0 |
| 9/28/2009 | 67.8 | 63.0 | 49.9 | 44.5 | 38.4 |
| 9/29/2009 | 68.2 | 61.7 | 48.1 | 42.7 | 37.2 |
| 9/30/2009 | 69.4 | 65.1 | 50.9 | 44.5 | 37.5 |
| 10/1/2009 | 71.6 | 65.8 | 47.5 | 42.8 | 36.5 |
| 10/2/2009 | 75.7 | 69.3 | 50.7 | 44.6 | 38.7 |
| 10/3/2009 | 72.4 | 64.1 | 45.7 | 41.2 | 37.2 |
| 10/4/2009 | 67.0 | 58.1 | 40.9 | 36.5 | 33.4 |
| 10/5/2009 | 62.0 | 57.1 | 44.6 | 42.4 | 37.3 |
| 10/6/2009 | 69.0 | 65.3 | 51.1 | 43.4 | 37.0 |
| 10/7/2009 | 67.4 | 63.1 | 49.3 | 43.2 | 36.8 |
| 10/8/2009 | 65.9 | 59.6 | 45.6 | 40.7 | 36.1 |
| 10/9/2009 | 67.9 | 62.8 | 49.2 | 44.0 | 37.8 |
| 10/10/2009 | 68.3 | 64.0 | 50.3 | 44.3 | 37.8 |
| 10/11/2009 | 65.1 | 59.6 | 46.1 | 40.9 | 35.9 |
| 10/12/2009 | 64.8 | 55.1 | 28.3 | 27.9 | 30.5 |
| 10/13/2009 | 67.8 | 58.8 | 39.2 | 34.8 | 32.4 |
| 10/14/2009 | 69.3 | 62.6 | 47.7 | 42.4 | 37.2 |
| 10/15/2009 | 68.6 | 63.8 | 50.2 | 44.4 | 38.1 |
| 10/16/2009 | 65.3 | 60.2 | 46.9 | 41.8 | 36.8 |
| 10/17/2009 | 62.5 | 56.9 | 43.8 | 39.3 | 35.5 |
| 10/18/2009 | 61.0 | 54.4 | 39.3 | 35.2 | 33.0 |
| 10/19/2009 | 61.8 | 55.5 | 42.3 | 39.0 | 35.1 |
| 10/20/2009 | 66.9 | 61.3 | 48.1 | 42.9 | 37.4 |
| 10/21/2009 | 69.0 | 64.1 | 50.2 | 44.1 | 37.7 |
| 10/22/2009 | 70.3 | 66.1 | 51.6 | 44.8 | 37.3 |
| 10/23/2009 | 73.6 | 68.3 | 52.3 | 44.2 | 36.9 |
| 10/24/2009 | 70.8 | 65.3 | 50.3 | 44.4 | 38.2 |
| 10/25/2009 | 64.4 | 58.5 | 44.0 | 39.1 | 35.4 |
| 10/26/2009 | 65.0 | 58.1 | 44.3 | 41.6 | 36.8 |
| 10/27/2009 | 58.4 | 52.2 | 39.9 | 41.0 | 36.3 |
| 10/28/2009 | 63.3 | 57.2 | 44.2 | 39.5 | 35.5 |
| 10/29/2009 | 66.9 | 62.1 | 48.7 | 43.3 | 38.1 |
| 10/30/2009 | 76.5 | 69.0 | 53.0 | 49.5 | 40.2 |
| 10/31/2009 | 71.7 | 66.4 | 49.5 | 43.9 | 39.8 |
| 11/1/2009 | 65.8 | 59.0 | 45.0 | 40.5 | 35.8 |
| 11/2/2009 | 74.9 | 64.6 | 41.9 | 38.8 | 33.1 |
| 11/3/2009 | 69.1 | 63.7 | 49.5 | 44.4 | 37.9 |
| 11/4/2009 | 76.8 | 69.5 | 50.1 | 41.9 | 36.7 |
| 11/5/2009 | 77.6 | 69.8 | 52.4 | 43.7 | 36.4 |
| 11/6/2009 | 70.0 | 66.2 | 51.4 | 44.7 | 37.7 |
| 11/7/2009 | 70.9 | 66.7 | 51.8 | 44.8 | 38.1 |
| 11/8/2009 | 69.1 | 64.0 | 49.6 | 43.5 | 37.8 |
| 11/9/2009 | 68.9 | 61.7 | 45.7 | 40.9 | 36.1 |
| 11/10/2009 | 74.8 | 66.8 | 47.7 | 42.1 | 37.2 |
| 11/11/2009 | 73.0 | 67.3 | 51.1 | 44.7 | 38.3 |
| 11/12/2009 | 69.4 | 64.2 | 49.7 | 44.6 | 39.1 |
| 11/13/2009 | 67.4 | 60.1 | 45.5 | 41.7 | 37.0 |
| 11/14/2009 | 81.9 | 73.3 | 58.2 | 48.4 | 42.6 |
| 11/15/2009 | 79.0 | 70.3 | 53.1 | 45.3 | 41.1 |
| 11/16/2009 | 83.1 | 76.2 | 57.3 | 51.5 | 49.7 |
| 11/17/2009 | 79.9 | 71.8 | 49.4 | 41.0 | 36.7 |
| 11/18/2009 | 76.3 | 68.8 | 51.8 | 44.9 | 38.5 |
| 11/19/2009 | 68.8 | 64.7 | 50.0 | 43.8 | 38.4 |
| 11/20/2009 | 68.6 | 64.2 | 50.1 | 44.1 | 38.4 |
| 11/21/2009 | 79.0 | 72.5 | 52.6 | 43.0 | 37.3 |
| 11/22/2009 | 67.0 | 60.8 | 46.5 | 41.3 | 36.2 |
| 11/23/2009 | 81.0 | 72.5 | 52.2 | 44.3 | 40.2 |
| 11/24/2009 | 74.2 | 67.7 | 51.4 | 44.2 | 38.0 |
| 11/25/2009 | 72.5 | 65.2 | 49.5 | 43.1 | 37.8 |
| 11/26/2009 | 69.8 | 59.7 | 44.7 | 37.5 | 33.9 |
| 11/27/2009 | 71.2 | 60.9 | 43.8 | 37.0 | 33.9 |
| 11/28/2009 | 72.2 | 65.3 | 50.2 | 44.0 | 39.2 |
| 11/29/2009 | 67.5 | 62.8 | 48.3 | 41.8 | 36.9 |
| 11/30/2009 | 71.0 | 67.6 | 50.0 | 43.5 | 37.8 |
| 12/1/2009 | 65.0 | 60.4 | 46.9 | 41.8 | 37.4 |
| 12/2/2009 | 70.6 | 66.7 | 51.2 | 44.5 | 37.8 |
| 12/3/2009 | 64.3 | 59.7 | 45.7 | 40.7 | 37.0 |
| 12/4/2009 | 72.2 | 68.3 | 51.7 | 43.8 | 36.8 |
| 12/5/2009 | 72.0 | 68.6 | 51.1 | 43.2 | 36.8 |
| 12/6/2009 | 70.9 | 66.6 | 51.1 | 44.3 | 38.2 |
| 12/7/2009 | 72.6 | 69.3 | 51.4 | 43.1 | 36.5 |
| 12/8/2009 | 68.1 | 64.2 | 49.2 | 43.4 | 38.0 |
| 12/9/2009 | 66.3 | 62.2 | 47.8 | 42.5 | 37.9 |
| 12/10/2009 | 67.3 | 63.0 | 48.5 | 43.0 | 38.3 |
| 12/11/2009 | 65.7 | 60.4 | 46.6 | 41.6 | 37.6 |
| 12/12/2009 | 60.0 | 52.8 | 38.1 | 34.7 | 31.5 |
| 12/13/2009 | 64.6 | 59.0 | 45.1 | 40.5 | 36.5 |
| 12/14/2009 | 64.1 | 57.6 | 44.0 | 39.3 | 36.2 |
| 12/15/2009 | 70.7 | 66.6 | 50.8 | 44.1 | 37.7 |
| 12/16/2009 | 70.3 | 66.1 | 50.7 | 44.1 | 37.7 |
| 12/17/2009 | 70.1 | 65.9 | 50.6 | 44.3 | 38.3 |
| 12/18/2009 | 66.0 | 61.6 | 47.0 | 41.9 | 37.2 |
| 12/19/2009 | 67.5 | 63.1 | 48.8 | 44.1 | 38.5 |
| 12/20/2009 | 69.5 | 65.7 | 50.5 | 44.1 | 37.9 |
| 12/21/2009 | 69.6 | 65.6 | 51.6 | 45.9 | 40.3 |
| 12/22/2009 | 66.9 | 62.6 | 48.4 | 43.3 | 38.0 |
| 12/23/2009 | 62.1 | 57.6 | 43.7 | 39.3 | 34.9 |
| 12/24/2009 | 69.3 | 64.9 | 50.1 | 43.9 | 37.8 |
| 12/25/2009 | 67.9 | 63.6 | 49.1 | 43.8 | 38.2 |
| 12/26/2009 | 65.5 | 61.1 | 47.2 | 42.2 | 37.3 |
| 12/27/2009 | 63.3 | 58.2 | 44.6 | 39.9 | 35.6 |
| 12/28/2009 | 68.4 | 63.9 | 49.2 | 43.5 | 37.5 |
| 12/29/2009 | 68.1 | 63.8 | 48.9 | 43.2 | 37.2 |
| 12/30/2009 | 66.4 | 61.6 | 47.8 | 42.7 | 37.5 |
| 12/31/2009 | 59.4 | 53.5 | 40.3 | 35.9 | 32.1 |
| 1/1/2010 | 63.7 | 58.1 | 44.4 | 39.5 | 35.5 |
| 1/2/2010 | 66.0 | 61.7 | 47.3 | 42.2 | 37.9 |
| 1/3/2010 | 70.6 | 65.9 | 50.1 | 43.7 | 38.0 |
| 1/4/2010 | 71.5 | 67.5 | 51.4 | 44.2 | 36.8 |
| 1/5/2010 | 66.4 | 61.9 | 48.0 | 42.5 | 37.3 |
| 1/6/2010 | 69.9 | 65.6 | 50.5 | 44.3 | 37.6 |
| 1/7/2010 | 68.4 | 64.0 | 49.5 | 44.0 | 38.2 |
| 1/8/2010 | 67.2 | 62.5 | 48.5 | 43.0 | 38.0 |
| 1/9/2010 | 67.4 | 62.5 | 48.5 | 43.1 | 38.1 |
| 1/10/2010 | 68.1 | 63.7 | 49.5 | 43.8 | 38.4 |
| 1/11/2010 | 65.6 | 61.0 | 47.0 | 41.7 | 37.3 |
| 1/12/2010 | 67.5 | 63.1 | 48.1 | 42.2 | 37.4 |
| 1/13/2010 | 66.6 | 62.2 | 47.8 | 42.5 | 38.0 |
| 1/14/2010 | 64.9 | 59.5 | 45.6 | 40.3 | 36.3 |
| 1/15/2010 | 68.8 | 64.5 | 49.6 | 43.8 | 38.1 |
| 1/16/2010 | 68.5 | 64.1 | 49.5 | 43.5 | 37.9 |
| 1/17/2010 | 67.9 | 63.3 | 49.1 | 43.5 | 38.5 |
| 1/18/2010 | 69.7 | 65.4 | 50.6 | 44.7 | 38.5 |
| 1/19/2010 | 71.9 | 68.0 | 52.0 | 44.6 | 36.9 |
| 1/20/2010 | 71.3 | 67.4 | 51.6 | 44.6 | 37.2 |
| 1/21/2010 | 71.6 | 64.8 | 49.4 | 43.7 | 38.9 |
| 1/22/2010 | 69.3 | 62.6 | 48.0 | 42.7 | 38.4 |
| 1/23/2010 | 64.9 | 60.9 | 45.4 | 40.4 | 36.6 |
| 1/24/2010 | 64.7 | 57.6 | 44.1 | 39.3 | 35.4 |
| 1/25/2010 | 65.9 | 59.6 | 45.8 | 40.6 | 36.9 |
| 1/26/2010 | 69.0 | 64.0 | 49.5 | 43.7 | 38.4 |
| 1/27/2010 | 69.9 | 65.5 | 50.7 | 44.6 | 38.7 |
| 1/28/2010 | 69.4 | 64.6 | 50.0 | 44.2 | 38.6 |
| 1/29/2010 | 77.4 | 68.9 | 46.5 | 40.2 | 35.9 |
| 1/30/2010 | 80.8 | 71.8 | 46.8 | 38.7 | 36.6 |
| 1/31/2010 | 81.5 | 71.9 | 47.7 | 42.3 | 38.1 |
| 2/1/2010 | 79.8 | 70.0 | 49.3 | 43.4 | 38.1 |
| 2/2/2010 | 69.7 | 61.1 | 37.3 | 31.8 | 29.8 |
| 2/3/2010 | 70.8 | 62.2 | 46.4 | 41.3 | 36.8 |
| 2/4/2010 | 70.8 | 65.7 | 50.3 | 43.9 | 37.8 |
| 2/5/2010 | 72.7 | 68.5 | 52.4 | 44.5 | 36.4 |
| 2/6/2010 | 72.2 | 67.7 | 52.2 | 44.9 | 37.2 |
| 2/7/2010 | 68.3 | 61.9 | 47.9 | 43.3 | 39.7 |
| 2/8/2010 | 72.3 | 68.1 | 48.7 | 40.6 | 36.7 |
| 2/9/2010 | 69.4 | 65.3 | 48.1 | 41.0 | 35.4 |
| 2/10/2010 | 67.6 | 61.2 | 47.0 | 41.4 | 36.4 |
| 2/11/2010 | 67.4 | 61.2 | 47.1 | 41.4 | 36.5 |
| 2/12/2010 | 66.0 | 57.7 | 43.9 | 38.5 | 34.7 |
| 2/13/2010 | 65.5 | 57.6 | 43.7 | 38.2 | 34.6 |
| 2/14/2010 | 66.9 | 56.9 | 40.5 | 34.6 | 31.9 |
| 2/15/2010 | 66.1 | 59.0 | 45.5 | 40.1 | 37.1 |
| 2/16/2010 | 64.7 | 57.7 | 44.3 | 39.0 | 36.1 |
| 2/17/2010 | 62.9 | 56.1 | 42.7 | 37.2 | 34.1 |
| 2/18/2010 | 61.8 | 48.4 | 37.3 | 33.6 | 34.1 |
| 2/19/2010 | 65.1 | 57.6 | 46.2 | 44.4 | 43.1 |
| 2/20/2010 | 64.2 | 54.2 | 40.1 | 34.6 | 32.4 |
| 2/21/2010 | 65.6 | 55.5 | 43.4 | 40.8 | 38.8 |
| 2/22/2010 | 62.9 | 51.3 | 45.5 | 44.9 | 43.6 |
| 2/23/2010 | 64.1 | 57.3 | 44.7 | 41.6 | 39.3 |
| 2/24/2010 | 67.4 | 61.9 | 48.0 | 42.4 | 37.8 |
| 2/25/2010 | 67.5 | 62.1 | 48.0 | 42.5 | 37.9 |
| 2/26/2010 | 68.4 | 62.9 | 49.0 | 43.3 | 38.2 |
| 2/27/2010 | 73.2 | 68.3 | 54.3 | 47.9 | 43.0 |
| 2/28/2010 | 72.5 | 66.9 | 53.7 | 48.8 | 46.8 |
| 3/1/2010 | 72.8 | 66.2 | 51.0 | 45.3 | 41.7 |
| 3/2/2010 | 68.9 | 59.9 | 44.2 | 40.7 | 39.9 |
| 3/3/2010 | 69.9 | 63.7 | 51.6 | 47.7 | 45.8 |
| 3/4/2010 | 69.2 | 63.0 | 50.6 | 46.7 | 44.7 |
| 3/5/2010 | 73.9 | 67.0 | 50.6 | 43.8 | 39.0 |
| 3/6/2010 | 66.1 | 58.9 | 47.0 | 44.2 | 43.4 |
| 3/7/2010 | 64.9 | 57.2 | 46.9 | 44.2 | 42.5 |
| 3/8/2010 | 62.5 | 54.2 | 38.1 | 34.0 | 34.3 |
| 3/9/2010 | 67.3 | 58.9 | 42.9 | 36.9 | 36.7 |
| 3/10/2010 | 67.8 | 59.3 | 43.6 | 39.4 | 39.3 |
| 3/11/2010 | 71.7 | 62.3 | 39.0 | 33.5 | 32.6 |
| 3/12/2010 | 69.9 | 59.4 | 40.0 | 36.9 | 35.1 |
| 3/13/2010 | 70.9 | 56.6 | 38.7 | 36.4 | 37.7 |
| 3/14/2010 | 65.7 | 55.3 | 38.3 | 33.5 | 31.3 |
| 3/15/2010 | 69.3 | 55.2 | 32.5 | 29.6 | 30.9 |
| 3/16/2010 | 71.3 | 61.1 | 38.2 | 35.6 | 42.7 |
| 3/17/2010 | 69.5 | 58.8 | 43.0 | 41.7 | 40.5 |
| 3/18/2010 | 67.5 | 55.6 | 41.4 | 39.0 | 38.0 |
| 3/19/2010 | 65.7 | 55.9 | 46.2 | 42.7 | 41.5 |
| 3/20/2010 | 62.0 | 53.0 | 44.3 | 42.3 | 41.1 |
| 3/21/2010 | 61.6 | 52.2 | 43.7 | 44.0 | 41.6 |
| 3/22/2010 | 57.5 | 44.3 | 33.1 | 30.7 | 31.7 |
| 3/23/2010 | 56.0 | 46.9 | 36.9 | 34.5 | 35.0 |
| 3/24/2010 | 59.6 | 47.5 | 38.5 | 41.1 | 40.6 |
| 3/25/2010 | 61.2 | 54.8 | 46.2 | 45.2 | 44.6 |
| 3/26/2010 | 57.2 | 48.8 | 41.1 | 40.0 | 39.6 |
| 3/27/2010 | 60.4 | 50.2 | 38.7 | 36.8 | 36.2 |
| 3/28/2010 | 57.6 | 47.1 | 35.2 | 32.7 | 34.2 |
| 3/29/2010 | 62.5 | 52.5 | 40.0 | 36.6 | 36.0 |
| 3/30/2010 | 67.1 | 51.3 | 34.9 | 30.8 | 31.7 |
| 3/31/2010 | 68.5 | 55.2 | 38.2 | 33.6 | 31.0 |
| 4/1/2010 | 69.2 | 57.7 | 42.5 | 38.7 | 37.9 |
| 4/2/2010 | 66.7 | 58.2 | 44.7 | 40.9 | 38.9 |
| 4/3/2010 | 64.4 | 53.9 | 40.8 | 38.1 | 36.8 |
| 4/4/2010 | 63.1 | 53.9 | 43.8 | 40.8 | 39.2 |
| 4/5/2010 | 66.2 | 54.5 | 45.8 | 42.8 | 41.3 |
| 4/6/2010 | 63.2 | 54.3 | 42.7 | 39.6 | 38.0 |
| 4/7/2010 | 65.1 | 55.9 | 40.5 | 37.9 | 36.3 |
| 4/8/2010 | 65.5 | 55.3 | 36.9 | 35.1 | 35.2 |
| 4/9/2010 | 63.8 | 54.4 | 40.5 | 37.9 | 36.8 |
| 4/10/2010 | 69.6 | 64.2 | 55.7 | 53.2 | 50.5 |
| 4/11/2010 | 71.2 | 65.8 | 56.9 | 54.4 | 51.5 |
| 4/12/2010 | 72.5 | 67.5 | 56.4 | 51.9 | 47.8 |
| 4/13/2010 | 66.1 | 60.8 | 46.7 | 41.2 | 36.1 |
| 4/14/2010 | 63.9 | 57.0 | 43.2 | 38.1 | 34.9 |
| 4/15/2010 | 67.2 | 61.3 | 47.0 | 41.2 | 35.8 |
| 4/16/2010 | 66.3 | 60.7 | 46.3 | 39.9 | 34.5 |
| 4/17/2010 | 59.8 | 51.4 | 37.8 | 32.5 | 31.3 |
| 4/18/2010 | 69.4 | 64.6 | 49.8 | 43.4 | 37.2 |
| 4/19/2010 | 66.6 | 61.0 | 47.0 | 41.0 | 35.7 |
| 4/20/2010 | 66.8 | 60.2 | 46.4 | 40.8 | 36.1 |
| 4/21/2010 | 65.5 | 57.7 | 43.7 | 38.4 | 34.7 |
| 4/22/2010 | 64.3 | 56.3 | 42.8 | 37.8 | 35.2 |
| 4/23/2010 | 63.0 | 53.3 | 39.6 | 34.8 | 32.6 |
| 4/24/2010 | 66.9 | 60.3 | 46.1 | 40.4 | 35.6 |
| 4/25/2010 | 66.0 | 59.6 | 45.9 | 40.7 | 36.4 |
| 4/26/2010 | 70.2 | 64.7 | 51.1 | 46.1 | 41.2 |
| 4/27/2010 | 74.0 | 68.5 | 59.6 | 58.1 | 55.8 |
| 4/28/2010 | 81.4 | 73.6 | 59.3 | 55.7 | 52.0 |
| 4/29/2010 | 70.0 | 64.3 | 55.6 | 53.6 | 50.8 |
| 4/30/2010 | 69.8 | 64.2 | 50.2 | 43.2 | 36.8 |
| 5/1/2010 | 78.2 | 72.0 | 53.3 | 48.9 | 47.4 |
| 5/2/2010 | 67.0 | 61.2 | 48.4 | 44.3 | 41.0 |
| 5/3/2010 | 61.3 | 52.1 | 39.6 | 36.7 | 34.1 |
| 5/4/2010 | 60.2 | 44.1 | 32.4 | 33.2 | 32.6 |
| 5/5/2010 | 63.6 | 56.6 | 44.4 | 41.8 | 39.8 |
| 5/6/2010 | 61.5 | 53.2 | 40.1 | 36.1 | 33.7 |
| 5/7/2010 | 66.7 | 60.6 | 47.2 | 41.1 | 36.1 |
| 5/8/2010 | 63.9 | 55.6 | 42.7 | 37.5 | 34.2 |
| 5/9/2010 | 71.8 | 63.4 | 44.1 | 39.6 | 34.9 |
| 5/10/2010 | 64.1 | 52.9 | 39.0 | 35.2 | 31.8 |
| 5/11/2010 | 67.1 | 61.6 | 48.2 | 41.5 | 35.6 |
| 5/12/2010 | 61.5 | 54.0 | 41.4 | 36.1 | 33.8 |
| 5/13/2010 | 64.4 | 58.4 | 45.3 | 39.8 | 41.2 |
| 5/14/2010 | 57.8 | 47.2 | 33.4 | 33.6 | 47.1 |
| 5/15/2010 | 61.1 | 54.9 | 42.2 | 38.0 | 41.4 |
| 5/16/2010 | 70.8 | 63.4 | 49.0 | 42.5 | 37.0 |
| 5/17/2010 | 67.5 | 62.0 | 49.6 | 42.4 | 39.4 |
| 5/18/2010 | 66.8 | 62.0 | 48.8 | 42.3 | 40.8 |
| 5/19/2010 | 58.1 | 52.7 | 39.8 | 35.4 | 45.3 |
| 5/20/2010 | 67.2 | 61.9 | 48.7 | 42.0 | 39.9 |
| 5/21/2010 | 70.7 | 66.0 | 51.4 | 43.0 | 40.3 |
| 5/22/2010 | 63.1 | 53.8 | 39.6 | 34.5 | 32.4 |
| 5/23/2010 | 62.7 | 57.6 | 44.8 | 38.8 | 47.3 |
| 5/24/2010 | 72.5 | 63.8 | 47.0 | 40.3 | 41.6 |
| 5/25/2010 | 61.6 | 56.8 | 44.0 | 38.4 | 44.2 |
| 5/26/2010 | 59.4 | 52.5 | 39.9 | 35.0 | 43.2 |
| 5/27/2010 | 67.5 | 63.3 | 50.4 | 42.9 | 44.6 |
| 5/28/2010 | 68.7 | 64.4 | 51.0 | 43.6 | 41.3 |
| 5/29/2010 | 67.3 | 62.8 | 51.0 | 43.1 | 38.2 |
| 5/30/2010 | 62.8 | 57.8 | 45.2 | 39.5 | 44.4 |
| 5/31/2010 | 64.6 | 58.5 | 45.6 | 40.2 | 45.2 |
| 6/1/2010 | 58.0 | 52.1 | 39.9 | 36.7 | 38.7 |
| 6/2/2010 | 68.0 | 63.8 | 51.2 | 43.2 | 42.4 |
| 6/3/2010 | 68.2 | 64.0 | 51.5 | 42.9 | 39.5 |
| 6/4/2010 | 60.9 | 56.9 | 44.0 | 38.2 | 42.4 |
| 6/5/2010 | 60.8 | 56.5 | 44.2 | 38.3 | 43.0 |
| 6/6/2010 | 70.6 | 63.3 | 43.1 | 37.3 | 45.3 |
| 6/7/2010 | 62.0 | 57.2 | 44.7 | 38.8 | 36.3 |
| 6/8/2010 | 57.7 | 52.8 | 41.2 | 41.9 | 48.1 |
| 6/9/2010 | 55.1 | 50.4 | 38.6 | 33.5 | 46.2 |
| 6/10/2010 | 58.3 | 53.7 | 41.5 | 36.3 | 49.0 |
| 6/11/2010 | 63.2 | 58.6 | 46.2 | 40.3 | 36.1 |
| 6/12/2010 | 62.0 | 57.1 | 45.0 | 39.1 | 35.1 |
| 6/13/2010 | 61.0 | 56.1 | 46.1 | 38.4 | 34.7 |
| 6/14/2010 | 66.5 | 61.9 | 52.1 | 45.9 | 37.8 |
| 6/15/2010 | 64.4 | 60.3 | 51.3 | 41.8 | 37.0 |
| 6/16/2010 | 64.3 | 60.2 | 48.5 | 42.8 | 37.4 |
| 6/17/2010 | 66.5 | 62.5 | 52.8 | 43.3 | 37.8 |
| 6/18/2010 | 63.8 | 59.3 | 51.1 | 41.7 | 37.0 |
| 6/19/2010 | 64.4 | 57.8 | 43.2 | 42.0 | 37.3 |
| 6/20/2010 | 57.6 | 51.9 | 39.0 | 39.0 | 34.3 |
| 6/21/2010 | 68.1 | 60.5 | 44.2 | 38.6 | 35.8 |
| 6/22/2010 | 62.7 | 58.1 | 46.4 | 40.1 | 36.1 |
| 6/23/2010 | 56.9 | 53.2 | 59.7 | 43.2 | 37.8 |
| 6/24/2010 | 54.0 | 48.7 | 37.3 | 32.8 | 33.0 |
| 6/25/2010 | 62.1 | 57.3 | 45.4 | 39.6 | 35.6 |
| 6/26/2010 | 65.1 | 60.3 | 48.1 | 41.7 | 36.9 |
| 6/27/2010 | 64.4 | 60.1 | 48.2 | 41.9 | 37.8 |
| 6/28/2010 | 60.3 | 55.8 | 46.4 | 38.7 | 36.6 |
| 6/29/2010 | 56.0 | 51.3 | 40.9 | 43.1 | 37.2 |
| 6/30/2010 | 58.6 | 53.2 | 40.7 | 38.0 | 33.4 |
| 7/1/2010 | 55.7 | 50.7 | 40.6 | 43.4 | 37.2 |
| 7/2/2010 | 60.0 | 51.3 | 36.9 | 37.0 | 34.3 |
| 7/3/2010 | 51.0 | 42.3 | 31.7 | 30.9 | 30.4 |
| 7/4/2010 | 55.2 | 50.2 | 39.3 | 35.9 | 33.4 |
| 7/5/2010 | 64.8 | 53.8 | 35.7 | 41.0 | 34.8 |
| 7/6/2010 | 60.9 | 54.8 | 42.9 | 37.5 | 34.4 |
| 7/7/2010 | 59.5 | 54.7 | 42.9 | 38.1 | 37.1 |
| 7/8/2010 | 51.3 | 45.9 | 35.2 | 32.5 | 37.1 |
| 7/9/2010 | 63.4 | 58.5 | 46.8 | 40.5 | 36.4 |
| 7/10/2010 | 64.6 | 60.2 | 49.5 | 41.8 | 37.3 |
| 7/11/2010 | 55.3 | 49.7 | 38.4 | 34.2 | 32.5 |
| 7/12/2010 | 60.9 | 50.4 | 39.8 | 35.4 | 38.5 |
| 7/13/2010 | 60.7 | 49.3 | 41.6 | 43.0 | 36.1 |
| 7/14/2010 | 56.3 | 51.5 | 40.0 | 34.6 | 32.3 |
| 7/15/2010 | 54.0 | 49.1 | 38.3 | 33.8 | 33.5 |
| 7/16/2010 | 49.7 | 44.4 | 32.9 | 29.1 | 29.9 |
| 7/17/2010 | 48.4 | 41.2 | 30.6 | 28.8 | 30.4 |
| 7/18/2010 | 60.1 | 50.2 | 34.6 | 35.9 | 31.8 |
| 7/19/2010 | 59.6 | 55.1 | 43.2 | 38.0 | 34.3 |
| 7/20/2010 | 60.1 | 54.2 | 42.5 | 39.9 | 35.4 |
| 7/21/2010 | 64.0 | 59.0 | 48.8 | 41.8 | 37.3 |
| 7/22/2010 | 62.3 | 56.8 | 44.8 | 39.8 | 35.6 |
| 7/23/2010 | 64.1 | 59.7 | 50.8 | 41.7 | 37.2 |
| 7/24/2010 | 66.0 | 61.1 | 53.7 | 43.3 | 38.9 |
| 7/25/2010 | 60.8 | 56.3 | 45.2 | 38.9 | 40.1 |
| 7/26/2010 | 60.2 | 55.8 | 44.2 | 38.1 | 42.0 |
| 7/27/2010 | 55.6 | 50.9 | 39.1 | 33.7 | 38.3 |
| 7/28/2010 | 58.9 | 53.7 | 42.0 | 36.4 | 38.3 |
| 7/29/2010 | 61.1 | 56.9 | 48.1 | 46.2 | 43.5 |
| 7/30/2010 | 56.4 | 51.8 | 41.9 | 43.4 | 40.4 |
| 7/31/2010 | 65.4 | 60.1 | 49.8 | 41.5 | 40.4 |
| 8/1/2010 | 55.1 | 49.8 | 38.3 | 33.6 | 39.9 |
| 8/2/2010 | 56.2 | 50.8 | 39.8 | 39.0 | 43.4 |
| 8/3/2010 | 61.0 | 56.2 | 44.7 | 42.8 | 38.4 |
| 8/4/2010 | 59.7 | 53.7 | 46.9 | 37.6 | 41.8 |
| 8/5/2010 | 62.7 | 48.8 | 33.6 | 30.6 | 43.9 |
| 8/6/2010 | 63.0 | 58.8 | 47.4 | 42.8 | 40.4 |
| 8/7/2010 | 66.4 | 61.6 | 53.2 | 44.0 | 37.9 |
| 8/8/2010 | 64.3 | 58.1 | 49.4 | 44.2 | 42.9 |
| 8/9/2010 | 65.8 | 62.0 | 54.1 | 43.4 | 37.8 |
| 8/10/2010 | 69.8 | 63.5 | 56.5 | 43.7 | 38.8 |
| 8/11/2010 | 65.1 | 60.3 | 49.3 | 42.5 | 38.5 |
| 8/12/2010 | 71.7 | 55.1 | 41.3 | 38.7 | 37.3 |
| 8/13/2010 | 72.0 | 61.1 | 43.0 | 34.9 | 40.8 |
| 8/14/2010 | 67.6 | 62.3 | 54.7 | 44.5 | 44.6 |
| 8/15/2010 | 65.5 | 61.6 | 56.4 | 45.5 | 44.3 |
| 8/16/2010 | 63.9 | 59.1 | 49.6 | 42.6 | 43.9 |
| 8/17/2010 | 66.4 | 61.8 | 53.6 | 48.4 | 46.5 |
| 8/18/2010 | 65.2 | 61.0 | 58.6 | 43.2 | 44.0 |
| 8/19/2010 | 61.2 | 56.6 | 49.6 | 40.4 | 45.4 |
| 8/20/2010 | 57.4 | 52.1 | 45.2 | 35.5 | 40.6 |
| 8/21/2010 | 61.7 | 55.7 | 44.0 | 40.1 | 43.2 |
| 8/22/2010 | 64.0 | 58.1 | 46.1 | 40.3 | 42.0 |
| 8/23/2010 | 63.8 | 59.2 | 47.8 | 41.3 | 42.1 |
| 8/24/2010 | 61.8 | 56.6 | 46.7 | 39.1 | 36.5 |
| 8/25/2010 | 64.7 | 53.8 | 33.3 | 30.4 | 44.1 |
| 8/26/2010 | 64.7 | 55.2 | 41.3 | 40.3 | 36.6 |
| 8/27/2010 | 62.8 | 56.4 | 44.0 | 39.2 | 35.7 |
| 8/28/2010 | 67.9 | 59.9 | 53.1 | 43.2 | 44.3 |
| 8/29/2010 | 59.1 | 53.4 | 41.3 | 36.3 | 45.6 |
| 8/30/2010 | 60.9 | 53.7 | 40.6 | 36.0 | 48.3 |
| 8/31/2010 | 62.6 | 57.7 | 45.3 | 39.5 | 45.9 |
| 9/1/2010 | 64.3 | 59.9 | 47.7 | 41.6 | 46.5 |
| 9/2/2010 | 65.4 | 57.8 | 45.3 | 39.7 | 47.9 |
| 9/3/2010 | 63.1 | 57.9 | 48.5 | 40.1 | 46.1 |
| 9/4/2010 | 66.7 | 61.2 | 51.6 | 43.3 | 41.7 |
| 9/5/2010 | 66.2 | 59.5 | 47.1 | 41.2 | 44.5 |
| 9/6/2010 | 64.0 | 58.4 | 54.8 | 42.9 | 44.9 |
| 9/7/2010 | 64.3 | 57.8 | 48.5 | 45.3 | 45.2 |
| 9/8/2010 | 68.1 | 64.2 | 55.9 | 45.1 | 40.5 |
| 9/9/2010 | 75.3 | 66.2 | 52.3 | 43.3 | 44.5 |
| 9/10/2010 | 71.7 | 59.9 | 40.3 | 33.1 | 47.6 |
| 9/11/2010 | 63.4 | 53.9 | 34.4 | 32.6 | 48.5 |
| 9/12/2010 | 60.6 | 49.0 | 36.2 | 31.8 | 50.7 |
| 9/13/2010 | 64.4 | 59.5 | 47.9 | 41.5 | 45.1 |
| 9/14/2010 | 64.8 | 60.6 | 50.5 | 42.4 | 45.6 |
| 9/15/2010 | 71.6 | 60.2 | 42.8 | 37.4 | 48.2 |
| 9/16/2010 | 61.4 | 52.3 | 38.3 | 33.7 | 48.1 |
| 9/17/2010 | 61.5 | 56.6 | 44.7 | 39.3 | 48.0 |
| 9/18/2010 | 65.4 | 59.0 | 46.7 | 41.0 | 46.4 |
| 9/19/2010 | 66.0 | 60.8 | 53.8 | 43.1 | 43.6 |
| 9/20/2010 | 63.1 | 57.9 | 46.5 | 40.5 | 46.4 |
| 9/21/2010 | 61.0 | 55.9 | 44.1 | 38.8 | 45.3 |
| 9/22/2010 | 61.6 | 54.8 | 42.8 | 37.4 | 47.8 |
| 9/23/2010 | 68.3 | 59.9 | 46.2 | 39.0 | 46.2 |
| 9/24/2010 | 68.6 | 63.3 | 63.6 | 47.4 | 44.6 |
| 9/25/2010 | 67.9 | 62.3 | 60.7 | 44.5 | 44.2 |
| 9/26/2010 | 70.1 | 66.4 | 63.1 | 47.9 | 44.0 |
| 9/27/2010 | 72.2 | 64.2 | 61.3 | 46.4 | 47.7 |
| 9/28/2010 | 75.6 | 69.3 | 60.6 | 47.6 | 48.0 |
| 9/29/2010 | 68.9 | 65.2 | 62.4 | 50.7 | 44.2 |
| 9/30/2010 | 71.1 | 68.1 | 65.4 | 48.2 | 42.9 |
| 10/1/2010 | 70.5 | 67.1 | 57.7 | 47.8 | 42.9 |
| 10/2/2010 | 70.7 | 67.1 | 57.5 | 45.8 | 41.0 |
| 10/3/2010 | 71.4 | 66.1 | 58.9 | 45.6 | 41.5 |
| 10/4/2010 | 67.0 | 61.2 | 56.4 | 43.4 | 50.8 |
| 10/5/2010 | 75.4 | 66.9 | 58.5 | 54.6 | 48.2 |
| 10/6/2010 | 70.6 | 61.7 | 42.1 | 38.4 | 34.6 |
| 10/7/2010 | 69.2 | 63.9 | 42.1 | 38.1 | 34.6 |
| 10/8/2010 | 72.8 | 67.1 | 44.3 | 37.8 | 34.8 |
| 10/9/2010 | 70.3 | 62.7 | 41.7 | 37.0 | 34.6 |
| 10/10/2010 | 69.1 | 59.4 | 39.1 | 35.1 | 33.3 |
| 10/11/2010 | 70.9 | 63.5 | 48.3 | 42.9 | 37.3 |
| 10/12/2010 | 69.8 | 64.0 | 50.1 | 44.6 | 38.0 |
| 10/13/2010 | 69.7 | 62.0 | 46.0 | 41.2 | 36.3 |
| 10/14/2010 | 64.9 | 57.7 | 44.8 | 41.4 | 36.2 |
| 10/15/2010 | 70.3 | 60.4 | 47.1 | 44.2 | 37.7 |
| 10/16/2010 | 72.9 | 65.6 | 52.8 | 46.1 | 37.7 |
| 10/17/2010 | 69.0 | 64.3 | 50.9 | 45.0 | 37.7 |
| 10/18/2010 | 70.2 | 65.3 | 50.9 | 44.7 | 37.6 |
| 10/19/2010 | 76.2 | 70.3 | 51.9 | 46.0 | 39.9 |
| 10/20/2010 | 79.8 | 71.6 | 50.4 | 42.0 | 37.1 |
| 10/21/2010 | 68.5 | 62.8 | 48.5 | 43.5 | 37.6 |
| 10/22/2010 | 67.4 | 61.6 | 47.7 | 42.5 | 36.5 |
| 10/23/2010 | 68.8 | 62.5 | 48.7 | 43.8 | 37.6 |
| 10/24/2010 | 67.8 | 59.2 | 40.7 | 41.0 | 35.0 |
| 10/25/2010 | 70.6 | 62.8 | 41.3 | 34.0 | 32.8 |
| 10/26/2010 | 68.8 | 62.3 | 48.5 | 43.5 | 37.6 |
| 10/27/2010 | 69.3 | 63.4 | 49.6 | 45.7 | 38.7 |
| 10/28/2010 | 69.0 | 63.8 | 49.4 | 44.2 | 38.1 |
| 10/29/2010 | 70.8 | 67.5 | 53.1 | 47.0 | 38.4 |
| 10/30/2010 | 66.7 | 61.1 | 47.1 | 44.4 | 37.9 |
| 10/31/2010 | 70.0 | 61.6 | 45.0 | 39.5 | 35.7 |
| 11/1/2010 | 70.6 | 64.0 | 48.5 | 43.8 | 38.1 |
| 11/2/2010 | 70.3 | 65.0 | 50.4 | 45.1 | 38.5 |
| 11/3/2010 | 69.7 | 63.6 | 49.1 | 44.1 | 38.6 |
| 11/4/2010 | 67.1 | 60.7 | 46.5 | 41.8 | 37.1 |
| 11/5/2010 | 66.4 | 59.4 | 45.1 | 40.7 | 36.4 |
| 11/6/2010 | 70.1 | 64.9 | 50.2 | 44.8 | 38.6 |
| 11/7/2010 | 69.7 | 62.4 | 47.3 | 42.2 | 37.1 |
| 11/8/2010 | 70.1 | 60.6 | 46.0 | 41.7 | 37.1 |
| 11/9/2010 | 71.7 | 61.7 | 46.0 | 42.2 | 37.2 |
| 11/10/2010 | 72.0 | 64.3 | 49.8 | 44.3 | 37.9 |
| 11/11/2010 | 68.8 | 62.9 | 49.0 | 44.1 | 38.2 |
| 11/12/2010 | 66.4 | 52.4 | 35.3 | 31.3 | 32.3 |
| 11/13/2010 | 66.3 | 53.4 | 37.6 | 34.2 | 33.7 |
| 11/14/2010 | 71.8 | 63.7 | 45.1 | 40.0 | 36.0 |
| 11/15/2010 | 66.5 | 57.9 | 43.6 | 39.1 | 35.3 |
| 11/16/2010 | 70.6 | 64.5 | 50.1 | 45.1 | 39.0 |
| 11/17/2010 | 68.3 | 62.0 | 48.0 | 43.1 | 37.8 |
| 11/18/2010 | 70.0 | 62.3 | 48.3 | 43.6 | 38.2 |
| 11/19/2010 | 70.5 | 65.2 | 50.9 | 45.4 | 38.4 |
| 11/20/2010 | 70.6 | 64.2 | 49.9 | 44.3 | 37.7 |
| 11/21/2010 | 69.4 | 63.2 | 49.5 | 44.6 | 38.1 |
| 11/22/2010 | 67.3 | 59.7 | 47.3 | 50.9 | 42.5 |
| 11/23/2010 | 68.9 | 62.0 | 48.8 | 46.2 | 39.8 |
| 11/24/2010 | 69.7 | 65.1 | 49.8 | 45.3 | 39.0 |
| 11/25/2010 | 73.5 | 65.8 | 50.5 | 44.7 | 38.3 |
| 11/26/2010 | 76.7 | 66.6 | 48.8 | 43.9 | 38.4 |
| 11/27/2010 | 78.0 | 68.4 | 46.7 | 42.4 | 37.6 |
| 11/28/2010 | 77.8 | 68.4 | 51.2 | 45.2 | 38.8 |
| 11/29/2010 | 85.2 | 75.1 | 52.2 | 45.3 | 41.0 |
| 11/30/2010 | 70.2 | 62.5 | 45.5 | 40.8 | 36.6 |
| 12/1/2010 | 63.4 | 57.8 | 43.3 | 39.8 | 34.1 |
| 12/2/2010 | 73.6 | 65.4 | 49.2 | 43.6 | 37.5 |
| 12/3/2010 | 75.4 | 68.6 | 52.1 | 45.4 | 38.3 |
| 12/4/2010 | 71.6 | 67.8 | 52.1 | 45.0 | 38.1 |
| 12/5/2010 | 72.8 | 68.9 | 52.1 | 44.4 | 36.9 |
| 12/6/2010 | 71.6 | 67.1 | 51.8 | 45.5 | 38.2 |
| 12/7/2010 | 70.5 | 65.7 | 51.0 | 45.7 | 38.7 |
| 12/8/2010 | 72.8 | 68.8 | 52.0 | 44.4 | 37.0 |
| 12/9/2010 | 72.7 | 69.0 | 52.0 | 44.0 | 36.5 |
| 12/10/2010 | 72.6 | 68.9 | 51.9 | 44.1 | 36.4 |
| 12/11/2010 | 72.3 | 68.2 | 52.0 | 44.4 | 37.4 |
| 12/12/2010 | 72.5 | 68.5 | 51.6 | 44.1 | 38.3 |
| 12/13/2010 | 69.0 | 64.4 | 49.9 | 44.2 | 37.9 |
| 12/14/2010 | 69.9 | 65.7 | 50.6 | 44.9 | 38.2 |
| 12/15/2010 | 72.6 | 68.4 | 52.0 | 44.4 | 36.9 |
| 12/16/2010 | 72.9 | 68.9 | 52.0 | 43.6 | 36.2 |
| 12/17/2010 | 66.9 | 62.0 | 47.7 | 42.1 | 36.5 |
| 12/18/2010 | 64.9 | 55.7 | 41.9 | 37.2 | 34.2 |
| 12/19/2010 | 68.0 | 63.1 | 49.2 | 44.1 | 38.0 |
| 12/20/2010 | 66.2 | 61.0 | 47.0 | 42.0 | 37.1 |
| 12/21/2010 | 71.2 | 66.9 | 50.9 | 44.0 | 37.3 |
| 12/22/2010 | 74.5 | 71.1 | 51.6 | 41.1 | 34.8 |
| 12/23/2010 | 69.0 | 64.6 | 49.4 | 43.4 | 37.7 |
| 12/24/2010 | 70.7 | 66.4 | 51.1 | 44.7 | 37.6 |
| 12/25/2010 | 72.8 | 68.4 | 51.8 | 43.9 | 36.4 |
| 12/26/2010 | 71.9 | 67.7 | 51.6 | 44.4 | 36.7 |
| 12/27/2010 | 68.1 | 63.7 | 49.1 | 43.7 | 37.7 |
| 12/28/2010 | 65.3 | 60.3 | 46.7 | 41.8 | 37.2 |
| 12/29/2010 | 71.0 | 66.3 | 51.4 | 45.0 | 37.8 |
| 12/30/2010 | 70.9 | 65.4 | 52.2 | 48.6 | 44.1 |
| 12/31/2010 | 69.2 | 63.0 | 49.3 | 45.1 | 40.7 |
| 1/1/2011 | 70.7 | 65.1 | 50.8 | 46.3 | 40.3 |
| 1/2/2011 | 67.2 | 62.3 | 47.9 | 42.0 | 36.2 |
| 1/3/2011 | 67.0 | 62.0 | 48.2 | 42.3 | 36.6 |
| 1/4/2011 | 71.2 | 67.1 | 50.6 | 43.1 | 36.6 |
| 1/5/2011 | 72.6 | 68.4 | 51.4 | 43.6 | 36.6 |
| 1/6/2011 | 64.6 | 59.5 | 46.1 | 40.4 | 35.7 |
| 1/7/2011 | 69.3 | 64.6 | 50.4 | 44.2 | 37.5 |
| 1/8/2011 | 67.7 | 62.9 | 49.2 | 43.4 | 37.4 |
| 1/9/2011 | 66.9 | 61.9 | 48.4 | 42.8 | 37.7 |
| 1/10/2011 | 66.5 | 61.7 | 48.3 | 43.9 | 37.6 |
| 1/11/2011 | 62.8 | 57.7 | 45.1 | 39.3 | 35.1 |
| 1/12/2011 | 64.0 | 58.8 | 46.0 | 40.2 | 35.9 |
| 1/13/2011 | 64.3 | 59.1 | 46.3 | 40.5 | 36.3 |
| 1/14/2011 | 67.2 | 62.3 | 48.5 | 42.8 | 38.4 |
| 1/15/2011 | 67.9 | 62.8 | 49.3 | 43.7 | 38.1 |
| 1/16/2011 | 63.1 | 57.6 | 45.2 | 39.2 | 35.5 |
| 1/17/2011 | 60.3 | 54.6 | 43.5 | 36.7 | 34.0 |
| 1/18/2011 | 71.8 | 67.5 | 51.6 | 44.0 | 37.4 |
| 1/19/2011 | 73.5 | 70.1 | 52.0 | 43.4 | 37.9 |
| 1/20/2011 | 64.5 | 59.1 | 46.5 | 40.4 | 35.6 |
| 1/21/2011 | 66.9 | 61.3 | 48.4 | 42.5 | 37.2 |
| 1/22/2011 | 67.3 | 61.8 | 48.4 | 42.2 | 37.7 |
| 1/23/2011 | 74.5 | 70.9 | 51.3 | 41.8 | 35.1 |
| 1/24/2011 | 66.3 | 60.3 | 47.1 | 42.2 | 36.6 |
| 1/25/2011 | 68.7 | 63.3 | 49.4 | 46.3 | 38.5 |
| 1/26/2011 | 68.8 | 63.8 | 49.8 | 43.8 | 37.9 |
| 1/27/2011 | 72.8 | 68.8 | 51.9 | 44.4 | 36.8 |
| 1/28/2011 | 72.1 | 68.1 | 51.8 | 44.5 | 36.8 |
| 1/29/2011 | 69.2 | 64.3 | 50.3 | 44.4 | 38.2 |
| 1/30/2011 | 66.4 | 61.4 | 48.1 | 42.5 | 37.2 |
| 1/31/2011 | 68.0 | 63.1 | 49.2 | 43.6 | 38.0 |
| 2/1/2011 | 69.8 | 65.0 | 50.5 | 44.6 | 38.1 |
| 2/2/2011 | 68.5 | 62.3 | 48.5 | 43.4 | 37.8 |
| 2/3/2011 | 67.3 | 58.1 | 45.1 | 39.3 | 35.4 |
| 2/4/2011 | 67.3 | 59.9 | 46.4 | 40.3 | 35.5 |
| 2/5/2011 | 72.1 | 67.6 | 52.2 | 45.0 | 37.2 |
| 2/6/2011 | 68.7 | 63.5 | 49.5 | 43.8 | 37.5 |
| 2/7/2011 | 66.6 | 59.0 | 46.2 | 40.8 | 36.0 |
| 2/8/2011 | 67.9 | 62.3 | 49.2 | 45.4 | 38.7 |
| 2/9/2011 | 70.4 | 65.8 | 51.0 | 44.8 | 37.8 |
| 2/10/2011 | 71.1 | 66.5 | 51.2 | 44.8 | 37.9 |
| 2/11/2011 | 69.4 | 64.2 | 49.6 | 43.3 | 37.4 |
| 2/12/2011 | 66.9 | 60.3 | 46.9 | 41.4 | 36.8 |
| 2/13/2011 | 72.2 | 67.9 | 52.1 | 44.9 | 37.4 |
| 2/14/2011 | 71.2 | 66.1 | 51.6 | 46.6 | 42.3 |
| 2/15/2011 | 69.6 | 61.9 | 49.7 | 46.1 | 42.1 |
| 2/16/2011 | 70.4 | 63.0 | 48.9 | 43.1 | 37.3 |
| 2/17/2011 | 70.4 | 64.6 | 50.7 | 45.0 | 38.1 |
| 2/18/2011 | 67.8 | 58.4 | 44.9 | 39.5 | 35.2 |
| 2/19/2011 | 71.1 | 62.9 | 48.9 | 43.3 | 37.5 |
| 2/20/2011 | 70.6 | 64.9 | 50.8 | 44.5 | 37.8 |
| 2/21/2011 | 67.3 | 57.0 | 44.1 | 39.0 | 35.1 |
| 2/22/2011 | 70.9 | 63.6 | 49.6 | 43.8 | 37.5 |
| 2/23/2011 | 70.4 | 64.2 | 50.1 | 44.2 | 37.8 |
| 2/24/2011 | 73.1 | 69.0 | 52.9 | 45.4 | 37.8 |
| 2/25/2011 | 66.8 | 61.8 | 48.0 | 42.3 | 36.8 |
| 2/26/2011 | 69.9 | 64.4 | 50.0 | 44.2 | 38.0 |
| 2/27/2011 | 70.1 | 63.9 | 49.7 | 44.2 | 38.4 |
| 2/28/2011 | 70.3 | 58.4 | 44.1 | 39.0 | 35.1 |
| 3/1/2011 | 71.0 | 57.8 | 42.9 | 37.7 | 34.4 |
| 3/2/2011 | 70.9 | 62.4 | 48.3 | 42.8 | 37.2 |
| 3/3/2011 | 73.3 | 67.3 | 52.7 | 47.6 | 43.2 |
| 3/4/2011 | 70.5 | 65.5 | 54.7 | 52.3 | 49.7 |
| 3/5/2011 | 67.5 | 57.4 | 45.0 | 41.7 | 38.8 |
| 3/6/2011 | 70.2 | 63.8 | 51.6 | 48.4 | 44.5 |
| 3/7/2011 | 70.9 | 64.4 | 53.3 | 51.6 | 48.9 |
| 3/8/2011 | 69.1 | 61.2 | 53.5 | 53.0 | 50.3 |
| 3/9/2011 | 66.1 | 51.0 | 40.3 | 38.6 | 38.4 |
| 3/10/2011 | 68.2 | 58.9 | 51.0 | 50.3 | 47.6 |
| 3/11/2011 | 69.4 | 54.6 | 43.4 | 41.4 | 39.3 |
| 3/12/2011 | 70.5 | 62.4 | 49.0 | 45.1 | 39.5 |
| 3/13/2011 | 67.1 | 56.4 | 47.9 | 46.8 | 44.2 |
| 3/14/2011 | 68.2 | 57.7 | 48.8 | 47.4 | 44.9 |
| 3/15/2011 | 68.0 | 59.8 | 50.0 | 48.1 | 45.2 |
| 3/16/2011 | 65.1 | 55.2 | 47.3 | 46.4 | 43.8 |
| 3/17/2011 | 65.2 | 58.0 | 46.6 | 44.1 | 42.6 |
| 3/18/2011 | 73.6 | 68.0 | 58.6 | 56.1 | 50.7 |
| 3/19/2011 | 67.1 | 61.0 | 47.0 | 40.7 | 35.3 |
| 3/20/2011 | 74.0 | 70.1 | 52.6 | 43.9 | 36.2 |
| 3/21/2011 | 66.9 | 61.7 | 48.2 | 42.5 | 36.9 |
| 3/22/2011 | 66.9 | 61.5 | 48.0 | 43.4 | 37.7 |
| 3/23/2011 | 66.1 | 61.3 | 46.7 | 40.9 | 36.0 |
| 3/24/2011 | 65.0 | 57.1 | 44.5 | 38.9 | 35.2 |
| 3/25/2011 | 63.6 | 53.3 | 41.1 | 34.7 | 32.6 |
| 3/26/2011 | 66.8 | 60.6 | 47.0 | 41.6 | 36.8 |
| 3/27/2011 | 67.2 | 61.1 | 47.5 | 42.1 | 37.0 |
| 3/28/2011 | 67.3 | 61.2 | 47.7 | 42.2 | 37.0 |
| 3/29/2011 | 68.9 | 63.6 | 49.6 | 44.1 | 38.2 |
| 3/30/2011 | 72.5 | 67.5 | 54.1 | 50.1 | 43.7 |
| 3/31/2011 | 71.9 | 66.9 | 56.4 | 53.6 | 49.4 |
| 4/1/2011 | 64.0 | 56.8 | 45.9 | 43.2 | 40.6 |
| 4/2/2011 | 70.2 | 65.2 | 56.0 | 55.0 | 49.9 |
| 4/3/2011 | 72.7 | 68.2 | 53.1 | 46.9 | 39.8 |
| 4/4/2011 | 72.2 | 67.3 | 57.9 | 56.7 | 52.5 |
| 4/5/2011 | 69.9 | 64.8 | 50.8 | 44.7 | 37.9 |
| 4/6/2011 | 70.9 | 66.5 | 51.8 | 45.4 | 37.4 |
| 4/7/2011 | 73.9 | 69.9 | 52.9 | 43.4 | 35.9 |
| 4/8/2011 | 72.8 | 68.8 | 52.1 | 43.6 | 35.9 |
| 4/9/2011 | 72.7 | 68.8 | 51.8 | 43.7 | 36.3 |
| 4/10/2011 | 66.9 | 61.3 | 47.6 | 41.8 | 36.5 |
| 4/11/2011 | 70.3 | 65.8 | 51.3 | 45.1 | 38.1 |
| 4/12/2011 | 71.7 | 67.3 | 52.1 | 44.8 | 37.1 |
| 4/13/2011 | 71.4 | 67.0 | 53.9 | 49.6 | 44.8 |
| 4/14/2011 | 66.7 | 61.0 | 51.2 | 48.3 | 43.6 |
| 4/15/2011 | 63.3 | 55.9 | 43.4 | 39.2 | 36.4 |
| 4/16/2011 | 59.5 | 46.9 | 39.2 | 34.5 | 33.7 |
| 4/17/2011 | 61.4 | 52.6 | 43.7 | 42.0 | 40.1 |
| 4/18/2011 | 65.4 | 59.6 | 50.7 | 48.8 | 45.1 |
| 4/19/2011 | 67.8 | 56.5 | 43.8 | 42.1 | 40.3 |
| 4/20/2011 | 63.5 | 54.1 | 43.5 | 41.5 | 40.6 |
| 4/21/2011 | 65.9 | 59.6 | 49.6 | 48.8 | 46.8 |
| 4/22/2011 | 69.6 | 64.3 | 54.6 | 53.8 | 51.3 |
| 4/23/2011 | 72.1 | 66.9 | 55.5 | 53.2 | 47.9 |
| 4/24/2011 | 74.1 | 69.5 | 60.0 | 58.3 | 52.7 |
| 4/25/2011 | 70.8 | 65.6 | 58.0 | 55.3 | 50.4 |
| 4/26/2011 | 71.2 | 64.0 | 54.8 | 52.8 | 49.9 |
| 4/27/2011 | 71.4 | 64.1 | 49.2 | 42.4 | 35.6 |
| 4/28/2011 | 69.0 | 60.7 | 46.6 | 40.9 | 35.4 |
| 4/29/2011 | 64.4 | 53.5 | 41.0 | 35.0 | 32.8 |
| 4/30/2011 | 65.4 | 53.5 | 40.7 | 34.2 | 32.5 |
| 5/1/2011 | 68.5 | 57.0 | 40.8 | 34.1 | 32.5 |
| 5/2/2011 | 79.9 | 68.4 | 52.8 | 46.7 | 45.3 |
| 5/3/2011 | 68.7 | 60.5 | 46.4 | 40.9 | 35.7 |
| 5/4/2011 | 62.4 | 56.7 | 44.2 | 38.5 | 34.1 |
| 5/5/2011 | 64.4 | 59.1 | 46.1 | 40.6 | 35.0 |
| 5/6/2011 | 64.7 | 59.7 | 46.5 | 40.7 | 34.7 |
| 5/7/2011 | 64.1 | 59.4 | 46.0 | 43.0 | 35.7 |
| 5/8/2011 | 62.9 | 48.0 | 37.0 | 34.4 | 31.5 |
| 5/9/2011 | 67.0 | 60.3 | 46.7 | 40.7 | 34.5 |
| 5/10/2011 | 72.9 | 66.0 | 51.0 | 44.6 | 37.1 |
| 5/11/2011 | 66.0 | 57.9 | 42.9 | 37.2 | 33.6 |
| 5/12/2011 | 69.9 | 62.1 | 44.2 | 38.6 | 34.4 |
| 5/13/2011 | 65.9 | 59.2 | 45.5 | 41.4 | 35.9 |
| 5/14/2011 | 69.7 | 64.9 | 51.1 | 45.2 | 37.1 |
| 5/15/2011 | 73.2 | 64.8 | 50.3 | 44.0 | 36.6 |
| 5/16/2011 | 64.3 | 57.8 | 44.7 | 39.1 | 34.0 |
| 5/17/2011 | 52.1 | 42.2 | 35.4 | 29.3 | 29.9 |
| 5/18/2011 | 57.6 | 50.0 | 38.1 | 33.7 | 31.6 |
| 5/19/2011 | NA | NA | NA | NA | NA |

B)

| **Date** | **500 Hz** | **2 kHz** | **10 kHz** | **20 kHz** | **40 kHz** |
| --- | --- | --- | --- | --- | --- |
| 9/26/2008 | NA | NA | NA | NA | NA |
| 9/27/2008 | 68.8 | 63.5 | 46.4 | 42.1 | 37.9 |
| 9/28/2008 | 75.9 | 70.6 | 53.1 | 45.9 | 38.3 |
| 9/29/2008 | 56.8 | 52.6 | 41.6 | 43.4 | 34.4 |
| 9/30/2008 | 54.1 | 51.3 | 39.3 | 39.7 | 32.0 |
| 10/1/2008 | 74.8 | 69.9 | 52.1 | 45.4 | 38.1 |
| 10/2/2008 | 76.0 | 70.5 | 52.8 | 45.0 | 38.5 |
| 10/3/2008 | 78.7 | 73.1 | 54.6 | 46.2 | 40.4 |
| 10/4/2008 | 78.7 | 73.6 | 54.8 | 46.4 | 40.2 |
| 10/5/2008 | 79.6 | 74.3 | 56.5 | 47.7 | 41.4 |
| 10/6/2008 | 77.3 | 71.8 | 54.2 | 46.1 | 39.7 |
| 10/7/2008 | 73.1 | 67.9 | 50.7 | 43.7 | 37.0 |
| 10/8/2008 | 75.0 | 69.5 | 51.5 | 43.4 | 37.2 |
| 10/9/2008 | 83.5 | 78.3 | 53.9 | 43.5 | 40.4 |
| 10/10/2008 | 83.1 | 77.7 | 54.4 | 43.5 | 40.6 |
| 10/11/2008 | 77.9 | 72.2 | 53.6 | 45.1 | 39.2 |
| 10/12/2008 | 70.7 | 65.5 | 48.1 | 40.5 | 34.8 |
| 10/13/2008 | 82.1 | 76.5 | 56.9 | 46.4 | 41.7 |
| 10/14/2008 | 79.6 | 73.5 | 55.2 | 46.7 | 40.7 |
| 10/15/2008 | 76.7 | 71.3 | 53.3 | 45.8 | 39.1 |
| 10/16/2008 | 69.0 | 64.1 | 47.4 | 41.2 | 34.9 |
| 10/17/2008 | 75.4 | 70.3 | 52.7 | 44.6 | 37.6 |
| 10/18/2008 | 78.0 | 72.5 | 54.6 | 46.2 | 39.5 |
| 10/19/2008 | 75.4 | 70.1 | 52.7 | 45.1 | 38.0 |
| 10/20/2008 | 62.9 | 58.3 | 41.9 | 36.3 | 31.9 |
| 10/21/2008 | 75.6 | 70.3 | 52.7 | 44.5 | 38.7 |
| 10/22/2008 | 73.9 | 68.9 | 51.7 | 46.4 | 40.1 |
| 10/23/2008 | 77.7 | 72.2 | 54.2 | 47.7 | 40.7 |
| 10/24/2008 | 76.8 | 71.6 | 53.2 | 44.9 | 39.3 |
| 10/25/2008 | 70.1 | 64.9 | 48.2 | 41.0 | 35.5 |
| 10/26/2008 | 57.2 | 53.1 | 37.1 | 38.0 | 33.9 |
| 10/27/2008 | 78.9 | 73.0 | 54.1 | 44.9 | 39.0 |
| 10/28/2008 | 82.6 | 76.8 | 56.9 | 45.9 | 41.1 |
| 10/29/2008 | 73.9 | 68.5 | 51.5 | 43.9 | 37.4 |
| 10/30/2008 | 72.2 | 66.9 | 50.1 | 42.4 | 37.1 |
| 10/31/2008 | 81.0 | 74.9 | 56.1 | 47.2 | 41.2 |
| 11/1/2008 | 75.6 | 70.0 | 52.6 | 45.4 | 38.9 |
| 11/2/2008 | 67.4 | 62.2 | 45.1 | 38.0 | 33.6 |
| 11/3/2008 | 72.7 | 67.5 | 50.1 | 42.6 | 36.7 |
| 11/4/2008 | 77.1 | 71.7 | 53.9 | 46.2 | 39.8 |
| 11/5/2008 | 78.7 | 73.0 | 54.7 | 46.4 | 40.8 |
| 11/6/2008 | 80.5 | 74.4 | 55.5 | 46.8 | 41.4 |
| 11/7/2008 | 78.4 | 72.6 | 54.6 | 46.1 | 40.1 |
| 11/8/2008 | 79.6 | 73.7 | 55.2 | 46.6 | 41.0 |
| 11/9/2008 | 79.1 | 73.4 | 55.2 | 46.8 | 41.2 |
| 11/10/2008 | 78.9 | 73.1 | 54.7 | 46.2 | 40.8 |
| 11/11/2008 | 74.8 | 69.2 | 51.5 | 44.3 | 38.4 |
| 11/12/2008 | 76.1 | 70.5 | 52.8 | 44.7 | 38.2 |
| 11/13/2008 | 73.8 | 68.0 | 50.8 | 44.4 | 37.4 |
| 11/14/2008 | 66.4 | 59.3 | 42.1 | 35.7 | 32.5 |
| 11/15/2008 | 78.5 | 72.7 | 54.8 | 46.3 | 40.6 |
| 11/16/2008 | 80.2 | 74.3 | 55.3 | 46.6 | 41.1 |
| 11/17/2008 | 81.3 | 75.5 | 56.2 | 47.1 | 42.0 |
| 11/18/2008 | 78.0 | 72.3 | 54.4 | 46.1 | 40.6 |
| 11/19/2008 | 71.7 | 62.1 | 45.3 | 38.1 | 34.6 |
| 11/20/2008 | 80.1 | 74.0 | 55.4 | 46.6 | 41.2 |
| 11/21/2008 | 83.5 | 77.5 | 54.7 | 43.9 | 41.1 |
| 11/22/2008 | 81.7 | 75.5 | 54.4 | 44.3 | 40.4 |
| 11/23/2008 | 78.2 | 71.8 | 53.8 | 45.9 | 40.5 |
| 11/24/2008 | 79.7 | 73.7 | 55.2 | 46.6 | 41.8 |
| 11/25/2008 | 78.7 | 72.6 | 54.6 | 46.3 | 40.8 |
| 11/26/2008 | 80.9 | 74.6 | 54.9 | 45.4 | 41.0 |
| 11/27/2008 | 78.2 | 71.8 | 53.9 | 45.8 | 40.2 |
| 11/28/2008 | 75.5 | 68.8 | 51.2 | 43.3 | 38.2 |
| 11/29/2008 | 78.0 | 70.4 | 52.9 | 44.9 | 40.0 |
| 11/30/2008 | 77.9 | 69.9 | 52.3 | 44.5 | 39.2 |
| 12/1/2008 | 82.8 | 75.5 | 55.6 | 46.6 | 42.1 |
| 12/2/2008 | 83.2 | 77.3 | 55.3 | 44.5 | 40.9 |
| 12/3/2008 | 83.4 | 77.4 | 55.4 | 44.6 | 40.9 |
| 12/4/2008 | 84.3 | 79.0 | 53.0 | 40.7 | 40.3 |
| 12/5/2008 | 82.6 | 76.6 | 55.4 | 45.9 | 41.3 |
| 12/6/2008 | 86.2 | 76.7 | 56.0 | 46.3 | 41.6 |
| 12/7/2008 | 80.8 | 70.3 | 52.2 | 44.9 | 40.4 |
| 12/8/2008 | 79.4 | 69.5 | 52.0 | 44.1 | 38.7 |
| 12/9/2008 | 84.5 | 76.5 | 55.0 | 46.5 | 41.5 |
| 12/10/2008 | 81.7 | 75.4 | 55.2 | 45.7 | 41.2 |
| 12/11/2008 | 79.7 | 73.5 | 54.7 | 46.6 | 40.8 |
| 12/12/2008 | 81.3 | 75.1 | 55.7 | 47.1 | 41.6 |
| 12/13/2008 | 81.6 | 75.4 | 56.2 | 47.0 | 41.9 |
| 12/14/2008 | 79.9 | 73.2 | 55.2 | 46.8 | 41.3 |
| 12/15/2008 | 75.9 | 69.4 | 51.6 | 44.9 | 39.4 |
| 12/16/2008 | 81.1 | 74.6 | 56.3 | 48.0 | 42.4 |
| 12/17/2008 | 80.1 | 73.6 | 55.8 | 47.0 | 41.2 |
| 12/18/2008 | 73.1 | 64.9 | 48.8 | 41.7 | 36.4 |
| 12/19/2008 | 76.0 | 68.3 | 51.8 | 44.1 | 39.0 |
| 12/20/2008 | 71.2 | 59.2 | 43.2 | 37.1 | 35.4 |
| 12/21/2008 | 76.9 | 69.7 | 52.7 | 44.7 | 38.4 |
| 12/22/2008 | 78.5 | 71.7 | 54.0 | 46.2 | 40.8 |
| 12/23/2008 | 83.3 | 77.8 | 54.7 | 45.0 | 40.9 |
| 12/24/2008 | 81.1 | 74.6 | 54.4 | 45.2 | 40.3 |
| 12/25/2008 | 82.6 | 76.2 | 55.7 | 45.9 | 41.1 |
| 12/26/2008 | 80.5 | 74.2 | 55.0 | 46.6 | 41.0 |
| 12/27/2008 | 82.0 | 75.7 | 56.0 | 46.7 | 41.4 |
| 12/28/2008 | 80.7 | 74.6 | 55.5 | 47.5 | 41.9 |
| 12/29/2008 | 81.3 | 74.9 | 56.1 | 47.3 | 41.8 |
| 12/30/2008 | 79.0 | 72.4 | 54.5 | 47.3 | 40.9 |
| 12/31/2008 | 76.2 | 64.7 | 48.1 | 41.6 | 37.0 |
| 1/1/2009 | 78.8 | 71.4 | 53.5 | 45.6 | 39.5 |
| 1/2/2009 | 81.6 | 75.0 | 56.4 | 47.6 | 42.8 |
| 1/3/2009 | 82.3 | 75.9 | 58.5 | 50.7 | 44.8 |
| 1/4/2009 | 80.8 | 73.5 | 56.1 | 48.1 | 42.1 |
| 1/5/2009 | 77.3 | 67.0 | 45.0 | 37.3 | 35.5 |
| 1/6/2009 | 81.8 | 65.8 | 45.7 | 38.4 | 37.1 |
| 1/7/2009 | 82.2 | 62.3 | 42.0 | 39.5 | 36.7 |
| 1/8/2009 | 84.6 | 65.0 | 44.9 | 41.6 | 39.1 |
| 1/9/2009 | 86.6 | 66.2 | 41.3 | 36.9 | 37.9 |
| 1/10/2009 | 85.9 | 65.6 | 38.7 | 41.7 | 39.8 |
| 1/11/2009 | 84.3 | 62.0 | 35.7 | 35.7 | 36.7 |
| 1/12/2009 | 88.2 | 64.5 | 37.4 | 36.1 | 37.2 |
| 1/13/2009 | 92.0 | 75.0 | 52.6 | 42.0 | 39.2 |
| 1/14/2009 | 86.4 | 67.0 | 47.9 | 43.2 | 39.9 |
| 1/15/2009 | 80.8 | 72.2 | 45.6 | 40.1 | 38.9 |
| 1/16/2009 | 81.2 | 62.8 | 43.9 | 42.0 | 38.3 |
| 1/17/2009 | 78.6 | 66.9 | 48.9 | 43.1 | 37.4 |
| 1/18/2009 | 74.7 | 54.0 | 38.3 | 34.5 | 33.3 |
| 1/19/2009 | 76.9 | 67.6 | 45.1 | 40.6 | 37.1 |
| 1/20/2009 | 78.2 | 59.9 | 43.7 | 42.8 | 37.3 |
| 1/21/2009 | 75.8 | 58.5 | 45.0 | 39.7 | 37.0 |
| 1/22/2009 | 75.8 | 62.7 | 47.7 | 42.5 | 39.0 |
| 1/23/2009 | 74.9 | 64.8 | 49.0 | 42.9 | 38.3 |
| 1/24/2009 | 76.7 | 64.1 | 47.2 | 41.7 | 36.6 |
| 1/25/2009 | 72.5 | 54.2 | 35.4 | 35.2 | 37.0 |
| 1/26/2009 | 72.7 | 53.5 | 38.4 | 39.5 | 40.5 |
| 1/27/2009 | 72.5 | 61.8 | 44.2 | 39.4 | 36.5 |
| 1/28/2009 | 79.4 | 55.3 | 38.4 | 33.1 | 33.1 |
| 1/29/2009 | 71.6 | 52.9 | 31.5 | 31.2 | 32.7 |
| 1/30/2009 | 70.4 | 52.3 | 32.8 | 30.3 | 30.6 |
| 1/31/2009 | 70.6 | 54.5 | 33.0 | 31.2 | 31.3 |
| 2/1/2009 | 77.8 | 58.9 | 36.1 | 36.0 | 35.1 |
| 2/2/2009 | 70.3 | 52.1 | 35.9 | 31.6 | 30.9 |
| 2/3/2009 | 73.1 | 59.7 | 52.2 | 42.1 | 32.9 |
| 2/4/2009 | 80.1 | 72.8 | 52.9 | 46.7 | 43.7 |
| 2/5/2009 | 79.0 | 74.3 | 54.2 | 50.5 | 46.5 |
| 2/6/2009 | 70.9 | 61.8 | 45.2 | 41.4 | 38.8 |
| 2/7/2009 | 71.5 | 60.8 | 41.5 | 35.5 | 32.8 |
| 2/8/2009 | 74.7 | 57.5 | 39.6 | 39.1 | 37.2 |
| 2/9/2009 | 76.8 | 72.0 | 53.0 | 41.8 | 37.6 |
| 2/10/2009 | 82.6 | 69.2 | 46.5 | 39.5 | 36.6 |
| 2/11/2009 | 76.7 | 64.2 | 46.6 | 42.8 | 39.8 |
| 2/12/2009 | 80.1 | 70.4 | 54.7 | 49.9 | 45.9 |
| 2/13/2009 | 78.2 | 63.0 | 38.7 | 34.9 | 32.8 |
| 2/14/2009 | 80.5 | 65.7 | 43.7 | 38.8 | 36.2 |
| 2/15/2009 | 73.2 | 62.5 | 36.9 | 33.7 | 32.3 |
| 2/16/2009 | 70.9 | 56.0 | 46.7 | 34.5 | 31.8 |
| 2/17/2009 | 72.1 | 58.6 | 40.5 | 32.7 | 31.1 |
| 2/18/2009 | 73.8 | 68.4 | 52.8 | 41.6 | 34.5 |
| 2/19/2009 | 74.7 | 62.9 | 35.2 | 33.6 | 32.6 |
| 2/20/2009 | 71.7 | 60.3 | 39.5 | 37.0 | 35.6 |
| 2/21/2009 | 75.9 | 68.0 | 52.1 | 48.5 | 45.2 |
| 2/22/2009 | 77.0 | 65.7 | 47.0 | 42.5 | 39.3 |
| 2/23/2009 | 82.2 | 73.0 | 59.2 | 54.8 | 49.9 |
| 2/24/2009 | 80.9 | 69.7 | 58.2 | 53.8 | 48.4 |
| 2/25/2009 | 74.8 | 64.2 | 53.8 | 50.6 | 45.4 |
| 2/26/2009 | 83.9 | 73.1 | 54.0 | 49.8 | 44.5 |
| 2/27/2009 | 76.4 | 68.5 | 55.6 | 51.5 | 46.8 |
| 2/28/2009 | 79.5 | 72.2 | 64.2 | 59.0 | 53.9 |
| 3/1/2009 | 77.2 | 69.2 | 55.9 | 50.4 | 45.0 |
| 3/2/2009 | 82.9 | 74.7 | 59.5 | 52.4 | 48.0 |
| 3/3/2009 | 84.3 | 62.0 | 45.6 | 46.2 | 46.0 |
| 3/4/2009 | 81.9 | 67.5 | 53.9 | 49.0 | 45.7 |
| 3/5/2009 | 92.0 | 77.9 | 64.6 | 58.5 | 51.0 |
| 3/6/2009 | 85.1 | 69.9 | 58.9 | 52.9 | 47.4 |
| 3/7/2009 | 87.1 | 70.2 | 52.9 | 49.6 | 46.7 |
| 3/8/2009 | 84.3 | 72.3 | 57.1 | 51.8 | 47.3 |
| 3/9/2009 | 83.2 | 69.2 | 55.5 | 49.2 | 43.9 |
| 3/10/2009 | 78.3 | 68.2 | 52.0 | 46.0 | 41.4 |
| 3/11/2009 | 79.0 | 58.4 | 46.9 | 46.9 | 40.7 |
| 3/12/2009 | 80.0 | 63.5 | 46.8 | 46.6 | 44.7 |
| 3/13/2009 | 78.6 | 65.1 | 51.7 | 47.7 | 44.3 |
| 3/14/2009 | 77.5 | 65.0 | 51.3 | 47.4 | 44.8 |
| 3/15/2009 | 78.3 | 66.8 | 51.2 | 47.0 | 44.2 |
| 3/16/2009 | 80.2 | 66.4 | 42.9 | 40.7 | 36.5 |
| 3/17/2009 | 82.5 | 68.3 | 40.0 | 37.5 | 34.7 |
| 3/18/2009 | 80.2 | 66.7 | 45.3 | 38.4 | 36.2 |
| 3/19/2009 | 79.3 | 69.1 | 43.8 | 40.0 | 37.2 |
| 3/20/2009 | 78.7 | 66.7 | 38.1 | 39.0 | 36.1 |
| 3/21/2009 | 79.4 | 66.6 | 36.9 | 34.6 | 32.9 |
| 3/22/2009 | 80.7 | 67.5 | 37.1 | 37.0 | 35.6 |
| 3/23/2009 | 84.1 | 73.6 | 49.2 | 42.5 | 37.6 |
| 3/24/2009 | 80.0 | 66.1 | 37.4 | 35.3 | 33.3 |
| 3/25/2009 | 79.9 | 67.3 | 40.9 | 38.1 | 35.1 |
| 3/26/2009 | 81.9 | 67.3 | 48.3 | 42.7 | 38.1 |
| 3/27/2009 | 77.9 | 64.3 | 39.9 | 34.8 | 32.5 |
| 3/28/2009 | 76.9 | 62.7 | 35.3 | 33.6 | 32.1 |
| 3/29/2009 | 75.1 | 66.2 | 49.7 | 43.0 | 38.0 |
| 3/30/2009 | 76.8 | 63.4 | 38.4 | 34.1 | 31.9 |
| 3/31/2009 | 72.8 | 63.4 | 34.1 | 32.2 | 31.4 |
| 4/1/2009 | 72.6 | 60.9 | 41.1 | 35.4 | 31.9 |
| 4/2/2009 | 76.9 | 65.6 | 41.2 | 35.3 | 33.1 |
| 4/3/2009 | 80.3 | 67.1 | 38.1 | 35.0 | 33.4 |
| 4/4/2009 | 78.3 | 64.8 | 42.2 | 40.9 | 38.8 |
| 4/5/2009 | 71.4 | 58.9 | 41.7 | 35.0 | 31.4 |
| 4/6/2009 | 78.4 | 65.7 | 47.4 | 42.9 | 42.0 |
| 4/7/2009 | 79.5 | 65.8 | 39.5 | 41.0 | 37.2 |
| 4/8/2009 | 76.2 | 63.3 | 33.9 | 34.9 | 37.1 |
| 4/9/2009 | 77.5 | 64.9 | 42.1 | 37.3 | 33.9 |
| 4/10/2009 | 75.6 | 65.8 | 38.3 | 36.4 | 34.7 |
| 4/11/2009 | 74.5 | 69.5 | 38.7 | 38.1 | 33.9 |
| 4/12/2009 | 72.3 | 62.3 | 35.4 | 33.7 | 32.7 |
| 4/13/2009 | 74.2 | 62.5 | 34.9 | 32.8 | 31.4 |
| 4/14/2009 | 71.9 | 62.7 | 38.1 | 35.5 | 33.2 |
| 4/15/2009 | 75.3 | 65.8 | 34.9 | 33.7 | 32.4 |
| 4/16/2009 | 73.9 | 62.9 | 33.8 | 32.6 | 31.3 |
| 4/17/2009 | 73.6 | 61.0 | 40.1 | 44.3 | 43.4 |
| 4/18/2009 | 78.0 | 65.3 | 44.4 | 41.0 | 37.1 |
| 4/19/2009 | 79.4 | 66.1 | 40.5 | 43.4 | 40.9 |
| 4/20/2009 | 73.9 | 61.5 | 37.9 | 37.8 | 34.8 |
| 4/21/2009 | 75.6 | 61.5 | 43.0 | 37.4 | 34.1 |
| 4/22/2009 | 76.6 | 65.3 | 40.0 | 36.3 | 32.6 |
| 4/23/2009 | 76.9 | 63.0 | 42.2 | 36.3 | 33.3 |
| 4/24/2009 | 75.0 | 62.5 | 39.5 | 35.2 | 31.7 |
| 4/25/2009 | 79.4 | 71.3 | 59.2 | 54.8 | 49.9 |
| 4/26/2009 | 81.8 | 73.4 | 54.7 | 50.4 | 45.7 |
| 4/27/2009 | 78.8 | 70.1 | 51.4 | 47.8 | 43.2 |
| 4/28/2009 | 77.8 | 69.4 | 46.9 | 44.5 | 40.6 |
| 4/29/2009 | 77.2 | 70.6 | 45.6 | 42.9 | 39.0 |
| 4/30/2009 | 76.9 | 69.1 | 52.4 | 49.4 | 44.8 |
| 5/1/2009 | 76.9 | 69.8 | 53.8 | 50.4 | 45.6 |
| 5/2/2009 | 76.5 | 68.3 | 48.6 | 46.9 | 42.8 |
| 5/3/2009 | 76.4 | 66.9 | 45.5 | 43.2 | 39.8 |
| 5/4/2009 | 76.3 | 66.6 | 42.0 | 41.7 | 40.0 |
| 5/5/2009 | 77.4 | 66.7 | 44.7 | 42.6 | 38.9 |
| 5/6/2009 | 76.2 | 65.2 | 39.0 | 36.7 | 34.7 |
| 5/7/2009 | 75.6 | 65.6 | 39.3 | 40.8 | 37.4 |
| 5/8/2009 | 74.7 | 66.4 | 42.9 | 40.1 | 36.2 |
| 5/9/2009 | 75.5 | 67.1 | 51.4 | 48.9 | 44.8 |
| 5/10/2009 | 76.0 | 69.3 | 50.6 | 49.1 | 45.6 |
| 5/11/2009 | 73.6 | 63.4 | 45.6 | 42.7 | 38.7 |
| 5/12/2009 | 75.8 | 68.1 | 56.1 | 54.1 | 49.5 |
| 5/13/2009 | 76.2 | 67.5 | 57.5 | 55.6 | 51.0 |
| 5/14/2009 | 76.5 | 68.8 | 55.1 | 52.7 | 48.0 |
| 5/15/2009 | 73.5 | 63.8 | 42.6 | 40.2 | 37.3 |
| 5/16/2009 | 78.1 | 72.2 | 54.2 | 47.1 | 40.9 |
| 5/17/2009 | 78.2 | 72.6 | 55.0 | 47.1 | 39.7 |
| 5/18/2009 | 73.8 | 68.4 | 50.9 | 42.5 | 36.0 |
| 5/19/2009 | 72.4 | 67.3 | 49.5 | 42.3 | 36.5 |
| 5/20/2009 | 73.2 | 67.8 | 50.0 | 41.6 | 34.6 |
| 5/21/2009 | 71.0 | 65.8 | 48.7 | 41.2 | 34.4 |
| 5/22/2009 | 76.7 | 71.1 | 53.0 | 43.3 | 36.2 |
| 5/23/2009 | 65.1 | 59.5 | 42.6 | 40.0 | 35.7 |
| 5/24/2009 | 67.9 | 62.8 | 45.6 | 37.6 | 32.7 |
| 5/25/2009 | 72.7 | 67.3 | 49.7 | 40.7 | 33.9 |
| 5/26/2009 | 75.4 | 70.2 | 52.2 | 43.2 | 35.2 |
| 5/27/2009 | 68.0 | 63.5 | 45.9 | 38.8 | 33.6 |
| 5/28/2009 | 67.8 | 63.3 | 46.3 | 38.8 | 33.6 |
| 5/29/2009 | 73.9 | 64.6 | 36.0 | 34.9 | 27.9 |
| 5/30/2009 | 99.9 | 93.7 | 75.8 | 67.3 | 55.7 |
| 5/31/2009 | 73.9 | 68.7 | 51.9 | 46.9 | 43.1 |
| 6/1/2009 | 67.6 | 62.8 | 47.2 | 44.5 | 42.2 |
| 6/2/2009 | 65.4 | 60.9 | 45.6 | 48.2 | 44.7 |
| 6/3/2009 | 71.0 | 65.8 | 50.3 | 46.0 | 41.8 |
| 6/4/2009 | 71.1 | 66.2 | 50.3 | 46.9 | 43.9 |
| 6/5/2009 | 70.6 | 65.9 | 50.1 | 47.4 | 43.1 |
| 6/6/2009 | 66.4 | 61.6 | 46.6 | 46.2 | 43.2 |
| 6/7/2009 | 63.5 | 59.1 | 44.3 | 45.0 | 42.5 |
| 6/8/2009 | 67.6 | 62.8 | 47.5 | 45.8 | 39.8 |
| 6/9/2009 | 58.8 | 54.6 | 41.0 | 46.5 | 41.2 |
| 6/10/2009 | 64.7 | 60.3 | 45.5 | 42.6 | 39.2 |
| 6/11/2009 | 70.8 | 65.9 | 50.3 | 46.8 | 41.5 |
| 6/12/2009 | 69.4 | 64.6 | 49.3 | 48.9 | 45.5 |
| 6/13/2009 | 59.4 | 54.8 | 40.7 | 42.5 | 39.8 |
| 6/14/2009 | 65.9 | 61.1 | 45.9 | 45.9 | 43.7 |
| 6/15/2009 | 58.7 | 54.5 | 40.9 | 45.6 | 42.9 |
| 6/16/2009 | 50.5 | 47.6 | 34.6 | 45.6 | 41.1 |
| 6/17/2009 | 64.3 | 59.5 | 44.7 | 43.0 | 41.2 |
| 6/18/2009 | 61.3 | 56.8 | 42.8 | 43.6 | 39.9 |
| 6/19/2009 | 74.5 | 69.2 | 53.1 | 49.0 | 44.8 |
| 6/20/2009 | 69.2 | 64.0 | 48.8 | 47.5 | 44.1 |
| 6/21/2009 | 73.1 | 66.3 | 46.9 | 47.2 | 43.3 |
| 6/22/2009 | 69.4 | 64.2 | 49.0 | 46.8 | 41.5 |
| 6/23/2009 | 68.8 | 63.7 | 48.6 | 44.6 | 39.3 |
| 6/24/2009 | 67.1 | 62.0 | 47.3 | 46.1 | 42.3 |
| 6/25/2009 | 68.7 | 63.6 | 48.4 | 46.7 | 41.2 |
| 6/26/2009 | 64.3 | 59.3 | 44.7 | 46.8 | 42.1 |
| 6/27/2009 | 47.8 | 46.5 | 34.7 | 46.2 | 43.5 |
| 6/28/2009 | 63.3 | 58.6 | 44.2 | 45.1 | 42.6 |
| 6/29/2009 | 69.5 | 64.4 | 49.5 | 47.4 | 42.5 |
| 6/30/2009 | 71.0 | 65.8 | 50.6 | 47.7 | 43.5 |
| 7/1/2009 | 73.0 | 67.8 | 52.2 | 47.6 | 43.5 |
| 7/2/2009 | 74.6 | 68.0 | 56.6 | 49.2 | 43.4 |
| 7/3/2009 | 77.7 | 72.4 | 55.8 | 48.1 | 44.4 |
| 7/4/2009 | 72.6 | 67.3 | 51.7 | 48.3 | 42.2 |
| 7/5/2009 | 67.3 | 62.3 | 47.4 | 46.5 | 42.9 |
| 7/6/2009 | 71.1 | 65.6 | 50.7 | 49.3 | 43.7 |
| 7/7/2009 | 74.3 | 69.0 | 53.0 | 47.8 | 41.4 |
| 7/8/2009 | 70.6 | 65.3 | 50.0 | 47.2 | 43.2 |
| 7/9/2009 | 58.0 | 54.2 | 39.9 | 47.8 | 44.8 |
| 7/10/2009 | 64.4 | 59.5 | 45.2 | 42.7 | 38.2 |
| 7/11/2009 | 68.9 | 63.9 | 48.7 | 46.3 | 41.4 |
| 7/12/2009 | 74.2 | 68.9 | 52.9 | 47.6 | 41.6 |
| 7/13/2009 | 74.8 | 69.3 | 53.2 | 49.4 | 44.4 |
| 7/14/2009 | 72.9 | 67.7 | 51.9 | 46.4 | 41.2 |
| 7/15/2009 | 67.2 | 62.1 | 47.3 | 46.8 | 43.9 |
| 7/16/2009 | 74.5 | 69.0 | 53.0 | 47.3 | 40.2 |
| 7/17/2009 | 71.5 | 66.1 | 50.8 | 47.0 | 41.3 |
| 7/18/2009 | 66.1 | 60.5 | 46.0 | 48.2 | 43.9 |
| 7/19/2009 | 53.9 | 50.4 | 38.2 | 44.8 | 43.2 |
| 7/20/2009 | 62.0 | 57.4 | 42.8 | 45.6 | 41.9 |
| 7/21/2009 | 72.2 | 67.0 | 51.6 | 50.9 | 45.5 |
| 7/22/2009 | 74.1 | 68.8 | 52.9 | 48.9 | 44.4 |
| 7/23/2009 | 69.7 | 64.4 | 49.1 | 47.8 | 42.5 |
| 7/24/2009 | 61.9 | 57.4 | 43.2 | 45.1 | 41.9 |
| 7/25/2009 | 65.0 | 60.1 | 45.6 | 46.2 | 42.0 |
| 7/26/2009 | 59.3 | 54.5 | 41.1 | 45.0 | 42.5 |
| 7/27/2009 | 57.7 | 53.3 | 40.1 | 44.6 | 43.4 |
| 7/28/2009 | 60.9 | 56.4 | 43.2 | 47.1 | 43.2 |
| 7/29/2009 | 58.2 | 53.5 | 40.0 | 42.8 | 40.1 |
| 7/30/2009 | 58.7 | 54.4 | 41.6 | 46.7 | 44.2 |
| 7/31/2009 | 65.3 | 60.3 | 46.0 | 46.6 | 41.9 |
| 8/1/2009 | 68.5 | 63.5 | 48.6 | 46.8 | 43.3 |
| 8/2/2009 | 60.9 | 56.4 | 42.3 | 43.7 | 40.6 |
| 8/3/2009 | 67.6 | 62.5 | 47.7 | 45.2 | 41.4 |
| 8/4/2009 | 71.2 | 66.2 | 50.7 | 45.9 | 41.0 |
| 8/5/2009 | 70.1 | 64.7 | 49.5 | 47.3 | 41.8 |
| 8/6/2009 | 69.5 | 64.0 | 49.0 | 48.2 | 44.0 |
| 8/7/2009 | 64.9 | 60.1 | 45.6 | 46.3 | 40.9 |
| 8/8/2009 | 50.8 | 47.1 | 37.4 | 46.0 | 44.0 |
| 8/9/2009 | 55.8 | 51.8 | 38.7 | 42.8 | 37.1 |
| 8/10/2009 | 62.0 | 56.9 | 43.1 | 47.8 | 44.2 |
| 8/11/2009 | 56.1 | 51.7 | 38.7 | 44.9 | 41.5 |
| 8/12/2009 | 67.1 | 61.9 | 48.3 | 48.1 | 43.6 |
| 8/13/2009 | 60.6 | 55.9 | 43.1 | 47.8 | 44.5 |
| 8/14/2009 | 47.1 | 45.4 | 36.2 | 45.1 | 42.5 |
| 8/15/2009 | 66.4 | 61.4 | 46.7 | 46.9 | 42.1 |
| 8/16/2009 | 62.9 | 58.1 | 43.8 | 40.1 | 38.5 |
| 8/17/2009 | 60.3 | 55.3 | 42.0 | 46.4 | 43.0 |
| 8/18/2009 | 67.9 | 62.7 | 47.7 | 44.6 | 42.0 |
| 8/19/2009 | 63.8 | 58.9 | 44.7 | 44.9 | 45.4 |
| 8/20/2009 | 71.1 | 65.5 | 50.4 | 46.6 | 43.9 |
| 8/21/2009 | 72.0 | 66.7 | 51.2 | 48.7 | 44.7 |
| 8/22/2009 | 71.4 | 66.0 | 50.6 | 47.7 | 44.9 |
| 8/23/2009 | 68.4 | 63.2 | 48.2 | 48.5 | 43.9 |
| 8/24/2009 | 66.2 | 61.0 | 46.2 | 48.1 | 44.4 |
| 8/25/2009 | 70.9 | 65.8 | 50.5 | 45.9 | 40.9 |
| 8/26/2009 | 68.0 | 63.0 | 47.7 | 48.1 | 45.0 |
| 8/27/2009 | 71.8 | 66.4 | 50.8 | 46.1 | 41.5 |
| 8/28/2009 | 63.5 | 59.0 | 44.7 | 45.8 | 41.1 |
| 8/29/2009 | 67.3 | 62.2 | 47.4 | 45.2 | 41.9 |
| 8/30/2009 | 73.2 | 67.9 | 51.8 | 48.1 | 44.7 |
| 8/31/2009 | 75.7 | 70.5 | 53.8 | 49.0 | 44.0 |
| 9/1/2009 | 70.7 | 65.6 | 50.1 | 48.0 | 43.8 |
| 9/2/2009 | 74.9 | 69.8 | 53.3 | 49.6 | 45.6 |
| 9/3/2009 | 71.1 | 65.9 | 50.2 | 46.1 | 42.7 |
| 9/4/2009 | 62.6 | 57.8 | 43.7 | 44.9 | 42.4 |
| 9/5/2009 | 55.9 | 52.2 | 39.8 | 46.5 | 43.2 |
| 9/6/2009 | 65.5 | 60.7 | 46.2 | 44.3 | 43.0 |
| 9/7/2009 | 74.9 | 69.4 | 53.3 | 48.0 | 42.4 |
| 9/8/2009 | 74.8 | 69.2 | 53.0 | 47.2 | 43.3 |
| 9/9/2009 | 73.4 | 68.1 | 52.3 | 48.4 | 45.3 |
| 9/10/2009 | 73.4 | 68.1 | 52.2 | 48.2 | 42.3 |
| 9/11/2009 | 63.8 | 58.5 | 44.2 | 48.0 | 47.8 |
| 9/12/2009 | 74.1 | 68.8 | 52.6 | 47.2 | 40.5 |
| 9/13/2009 | 75.4 | 70.0 | 53.4 | 48.4 | 43.3 |
| 9/14/2009 | 64.7 | 59.6 | 44.6 | 46.9 | 43.1 |
| 9/15/2009 | 68.5 | 63.3 | 48.6 | 47.4 | 43.9 |
| 9/16/2009 | 67.0 | 61.7 | 46.9 | 47.6 | 44.3 |
| 9/17/2009 | 74.5 | 69.1 | 53.2 | 47.3 | 41.8 |
| 9/18/2009 | 74.8 | 69.4 | 53.5 | 49.9 | 46.9 |
| 9/19/2009 | 74.5 | 69.2 | 53.2 | 48.8 | 44.3 |
| 9/20/2009 | 74.3 | 68.8 | 52.9 | 48.5 | 44.2 |
| 9/21/2009 | 68.0 | 62.8 | 47.8 | 43.4 | 40.5 |
| 9/22/2009 | 71.3 | 66.0 | 50.5 | 49.1 | 45.2 |
| 9/23/2009 | 75.3 | 69.9 | 53.6 | 49.3 | 45.5 |
| 9/24/2009 | 74.8 | 69.4 | 53.3 | 48.4 | 45.4 |
| 9/25/2009 | 70.3 | 65.2 | 49.8 | 44.9 | 41.7 |
| 9/26/2009 | 70.7 | 65.6 | 50.3 | 47.2 | 42.4 |
| 9/27/2009 | 72.4 | 67.2 | 51.1 | 47.4 | 44.8 |
| 9/28/2009 | 73.1 | 67.3 | 51.1 | 46.3 | 41.6 |
| 9/29/2009 | 85.6 | 75.6 | 53.6 | 52.7 | 52.0 |
| 9/30/2009 | 73.3 | 68.3 | 52.0 | 47.0 | 41.0 |
| 10/1/2009 | 66.7 | 61.7 | 46.4 | 40.4 | 34.5 |
| 10/2/2009 | 76.5 | 70.9 | 54.7 | 46.5 | 38.5 |
| 10/3/2009 | 71.0 | 66.0 | 50.4 | 46.0 | 38.6 |
| 10/4/2009 | 74.4 | 69.0 | 53.0 | 46.6 | 39.3 |
| 10/5/2009 | 71.3 | 66.1 | 50.6 | 44.5 | 38.3 |
| 10/6/2009 | 72.4 | 67.5 | 50.4 | 43.3 | 36.3 |
| 10/7/2009 | 77.2 | 71.5 | 54.0 | 46.0 | 38.0 |
| 10/8/2009 | 72.6 | 67.4 | 51.6 | 45.4 | 38.3 |
| 10/9/2009 | 68.1 | 63.0 | 47.9 | 42.0 | 36.3 |
| 10/10/2009 | 76.8 | 71.5 | 54.6 | 46.2 | 37.8 |
| 10/11/2009 | 78.5 | 73.3 | 53.7 | 44.6 | 37.3 |
| 10/12/2009 | 68.9 | 63.7 | 48.1 | 42.4 | 36.5 |
| 10/13/2009 | 53.8 | 48.8 | 33.9 | 29.2 | 28.0 |
| 10/14/2009 | 46.5 | 42.9 | 28.1 | 26.0 | 27.3 |
| 10/15/2009 | 61.0 | 55.6 | 38.9 | 33.7 | 30.4 |
| 10/16/2009 | 66.2 | 61.2 | 46.1 | 40.6 | 35.5 |
| 10/17/2009 | 68.6 | 63.4 | 48.3 | 42.7 | 37.1 |
| 10/18/2009 | 65.7 | 60.7 | 46.6 | 41.5 | 35.9 |
| 10/19/2009 | 71.8 | 66.6 | 50.8 | 45.0 | 38.7 |
| 10/20/2009 | 75.7 | 70.4 | 53.7 | 46.7 | 39.2 |
| 10/21/2009 | 76.7 | 71.2 | 54.0 | 46.6 | 38.5 |
| 10/22/2009 | 77.6 | 72.0 | 54.0 | 45.9 | 37.9 |
| 10/23/2009 | 75.7 | 70.3 | 53.4 | 46.4 | 39.2 |
| 10/24/2009 | 69.8 | 64.5 | 48.5 | 42.5 | 36.8 |
| 10/25/2009 | 70.7 | 65.6 | 49.8 | 44.0 | 38.3 |
| 10/26/2009 | 63.4 | 58.6 | 43.5 | 38.4 | 33.9 |
| 10/27/2009 | 61.2 | 56.5 | 41.4 | 36.1 | 32.9 |
| 10/28/2009 | 63.3 | 58.4 | 43.4 | 38.0 | 33.9 |
| 10/29/2009 | 70.9 | 65.4 | 50.0 | 44.0 | 38.9 |
| 10/30/2009 | 72.4 | 67.1 | 51.2 | 45.1 | 39.6 |
| 10/31/2009 | 64.2 | 59.4 | 44.2 | 38.7 | 34.8 |
| 11/1/2009 | 70.0 | 65.1 | 49.5 | 43.6 | 38.5 |
| 11/2/2009 | 71.4 | 66.3 | 50.3 | 44.3 | 38.6 |
| 11/3/2009 | 75.9 | 70.5 | 53.5 | 46.3 | 39.0 |
| 11/4/2009 | 73.4 | 68.2 | 51.8 | 45.5 | 39.4 |
| 11/5/2009 | 77.6 | 72.1 | 53.8 | 45.8 | 38.7 |
| 11/6/2009 | 77.5 | 72.0 | 53.7 | 45.8 | 38.3 |
| 11/7/2009 | 75.4 | 70.0 | 53.0 | 46.5 | 39.9 |
| 11/8/2009 | 70.7 | 65.7 | 49.7 | 44.0 | 38.5 |
| 11/9/2009 | 73.7 | 68.2 | 51.9 | 45.7 | 39.5 |
| 11/10/2009 | 72.4 | 67.2 | 51.0 | 45.1 | 39.8 |
| 11/11/2009 | 77.0 | 71.2 | 53.0 | 46.3 | 39.5 |
| 11/12/2009 | 73.6 | 67.6 | 49.6 | 42.6 | 37.2 |
| 11/13/2009 | 75.0 | 69.6 | 52.3 | 45.2 | 38.9 |
| 11/14/2009 | 75.9 | 70.4 | 53.4 | 46.5 | 39.8 |
| 11/15/2009 | 77.7 | 71.9 | 53.5 | 45.7 | 39.0 |
| 11/16/2009 | 75.4 | 69.8 | 52.2 | 45.5 | 39.4 |
| 11/17/2009 | 66.2 | 61.4 | 45.7 | 39.9 | 36.9 |
| 11/18/2009 | 71.7 | 66.4 | 50.3 | 44.5 | 39.4 |
| 11/19/2009 | 75.4 | 69.8 | 51.0 | 44.5 | 37.9 |
| 11/20/2009 | 78.2 | 72.5 | 53.3 | 45.7 | 38.5 |
| 11/21/2009 | 76.4 | 70.6 | 53.3 | 46.4 | 39.9 |
| 11/22/2009 | 78.3 | 72.6 | 53.3 | 44.8 | 37.9 |
| 11/23/2009 | 77.2 | 71.6 | 53.4 | 46.1 | 38.9 |
| 11/24/2009 | 71.0 | 65.8 | 49.4 | 44.1 | 39.3 |
| 11/25/2009 | 67.0 | 60.9 | 45.7 | 40.0 | 37.7 |
| 11/26/2009 | 74.9 | 69.3 | 52.3 | 45.8 | 39.7 |
| 11/27/2009 | 74.9 | 69.3 | 52.6 | 46.0 | 39.9 |
| 11/28/2009 | 75.0 | 69.5 | 52.6 | 46.1 | 40.0 |
| 11/29/2009 | 72.0 | 66.2 | 50.0 | 43.8 | 38.5 |
| 11/30/2009 | 79.2 | 73.5 | 51.9 | 43.2 | 36.3 |
| 12/1/2009 | 78.6 | 72.7 | 52.8 | 44.3 | 37.0 |
| 12/2/2009 | 76.3 | 70.7 | 53.2 | 46.2 | 39.5 |
| 12/3/2009 | 74.7 | 69.3 | 52.2 | 45.5 | 39.4 |
| 12/4/2009 | 77.6 | 72.1 | 51.7 | 43.4 | 37.2 |
| 12/5/2009 | 80.1 | 74.8 | 57.8 | 55.5 | 54.0 |
| 12/6/2009 | 75.8 | 69.6 | 53.2 | 46.4 | 39.6 |
| 12/7/2009 | 78.8 | 72.9 | 53.8 | 45.5 | 38.7 |
| 12/8/2009 | 78.6 | 72.6 | 53.5 | 45.9 | 38.8 |
| 12/9/2009 | 72.6 | 66.4 | 50.5 | 44.9 | 39.1 |
| 12/10/2009 | 74.3 | 68.8 | 52.3 | 46.2 | 39.6 |
| 12/11/2009 | 70.0 | 63.1 | 47.7 | 42.0 | 37.0 |
| 12/12/2009 | 71.6 | 65.3 | 49.9 | 44.0 | 38.3 |
| 12/13/2009 | 72.2 | 66.7 | 50.6 | 44.8 | 39.0 |
| 12/14/2009 | 75.6 | 70.1 | 53.1 | 46.4 | 39.8 |
| 12/15/2009 | 73.7 | 67.9 | 51.7 | 45.5 | 40.0 |
| 12/16/2009 | 75.8 | 70.1 | 52.8 | 46.2 | 39.9 |
| 12/17/2009 | 70.3 | 64.3 | 48.7 | 42.9 | 38.1 |
| 12/18/2009 | 74.8 | 69.4 | 52.9 | 46.4 | 40.2 |
| 12/19/2009 | 77.8 | 72.0 | 54.6 | 46.9 | 39.1 |
| 12/20/2009 | 77.5 | 71.5 | 53.8 | 46.0 | 38.5 |
| 12/21/2009 | 75.9 | 70.3 | 52.5 | 45.6 | 39.2 |
| 12/22/2009 | 74.7 | 68.8 | 52.1 | 45.3 | 39.1 |
| 12/23/2009 | 69.4 | 63.0 | 47.3 | 40.9 | 35.9 |
| 12/24/2009 | 73.0 | 64.6 | 48.8 | 42.3 | 37.2 |
| 12/25/2009 | 76.8 | 70.1 | 53.2 | 46.4 | 39.4 |
| 12/26/2009 | 75.7 | 69.9 | 52.9 | 46.0 | 39.5 |
| 12/27/2009 | 75.8 | 70.3 | 53.2 | 46.2 | 39.6 |
| 12/28/2009 | 71.7 | 66.2 | 50.8 | 44.4 | 38.8 |
| 12/29/2009 | 74.9 | 69.6 | 53.0 | 46.3 | 39.7 |
| 12/30/2009 | 75.3 | 69.8 | 53.2 | 46.5 | 40.2 |
| 12/31/2009 | 72.5 | 67.2 | 51.4 | 44.8 | 38.9 |
| 1/1/2010 | 70.8 | 65.6 | 50.1 | 44.1 | 38.5 |
| 1/2/2010 | 75.9 | 70.4 | 53.3 | 46.2 | 39.4 |
| 1/3/2010 | 77.2 | 71.6 | 53.5 | 45.6 | 38.2 |
| 1/4/2010 | 78.9 | 73.1 | 52.6 | 43.2 | 36.3 |
| 1/5/2010 | 76.9 | 71.4 | 50.8 | 42.3 | 36.3 |
| 1/6/2010 | 73.3 | 67.7 | 51.7 | 44.9 | 38.4 |
| 1/7/2010 | 77.4 | 71.7 | 54.4 | 46.8 | 39.0 |
| 1/8/2010 | 78.2 | 72.5 | 53.4 | 44.9 | 37.4 |
| 1/9/2010 | 77.5 | 71.9 | 53.3 | 45.1 | 37.5 |
| 1/10/2010 | 78.6 | 72.7 | 53.7 | 46.5 | 40.1 |
| 1/11/2010 | 76.4 | 70.1 | 56.2 | 52.7 | 49.7 |
| 1/12/2010 | 75.9 | 69.6 | 56.5 | 52.8 | 50.1 |
| 1/13/2010 | 67.6 | 60.2 | 46.5 | 41.1 | 38.7 |
| 1/14/2010 | 75.5 | 67.6 | 51.2 | 44.8 | 41.5 |
| 1/15/2010 | 80.8 | 72.1 | 52.9 | 48.6 | 56.0 |
| 1/16/2010 | 78.9 | 70.5 | 52.1 | 44.6 | 40.8 |
| 1/17/2010 | 77.8 | 70.5 | 52.9 | 45.4 | 42.7 |
| 1/18/2010 | 76.4 | 69.0 | 52.3 | 45.8 | 40.1 |
| 1/19/2010 | 63.3 | 56.2 | 41.5 | 32.9 | 31.9 |
| 1/20/2010 | 66.0 | 54.9 | 38.8 | 28.1 | 29.5 |
| 1/21/2010 | 66.9 | 56.9 | 39.3 | 31.2 | 32.6 |
| 1/22/2010 | 80.2 | 70.9 | 58.1 | 55.2 | 52.5 |
| 1/23/2010 | 74.7 | 67.2 | 55.7 | 52.5 | 51.3 |
| 1/24/2010 | 69.4 | 62.0 | 53.1 | 51.0 | 48.3 |
| 1/25/2010 | 67.2 | 57.8 | 48.9 | 46.5 | 43.2 |
| 1/26/2010 | 67.7 | 61.2 | 46.1 | 43.3 | 41.0 |
| 1/27/2010 | 69.9 | 58.4 | 40.9 | 35.8 | 32.8 |
| 1/28/2010 | 67.1 | 57.3 | 40.6 | 34.4 | 32.9 |
| 1/29/2010 | 71.7 | 58.6 | 36.1 | 28.6 | 29.6 |
| 1/30/2010 | 67.4 | 57.7 | 35.3 | 29.0 | 29.2 |
| 1/31/2010 | 66.1 | 53.6 | 33.3 | 29.9 | 31.7 |
| 2/1/2010 | 69.9 | 57.3 | 35.1 | 31.9 | 31.3 |
| 2/2/2010 | 76.8 | 64.8 | 36.9 | 30.2 | 30.2 |
| 2/3/2010 | 66.6 | 58.5 | 30.6 | 26.1 | 28.6 |
| 2/4/2010 | 73.4 | 59.5 | 39.4 | 37.7 | 36.0 |
| 2/5/2010 | 60.1 | 50.5 | 34.9 | 29.9 | 30.0 |
| 2/6/2010 | 61.6 | 50.6 | 33.3 | 27.8 | 28.4 |
| 2/7/2010 | 74.4 | 57.2 | 34.6 | 32.7 | 32.4 |
| 2/8/2010 | 68.0 | 55.5 | 54.0 | 43.6 | 34.6 |
| 2/9/2010 | 62.6 | 57.3 | 49.8 | 41.3 | 31.8 |
| 2/10/2010 | 66.1 | 54.8 | 37.9 | 32.0 | 29.6 |
| 2/11/2010 | 62.9 | 55.2 | 40.9 | 36.8 | 34.8 |
| 2/12/2010 | 64.0 | 55.1 | 39.9 | 35.0 | 34.3 |
| 2/13/2010 | 66.3 | 53.4 | 31.8 | 28.6 | 34.6 |
| 2/14/2010 | 73.5 | 59.5 | 31.1 | 33.1 | 29.2 |
| 2/15/2010 | 61.0 | 48.2 | 29.1 | 24.7 | 28.5 |
| 2/16/2010 | 76.3 | 55.4 | 31.1 | 29.5 | 37.0 |
| 2/17/2010 | 70.1 | 56.2 | 34.3 | 32.9 | 37.2 |
| 2/18/2010 | 70.5 | 58.1 | 41.3 | 38.7 | 37.7 |
| 2/19/2010 | 70.7 | 61.1 | 49.4 | 47.2 | 44.8 |
| 2/20/2010 | 67.6 | 57.6 | 31.5 | 28.1 | 29.9 |
| 2/21/2010 | 73.9 | 63.5 | 34.7 | 31.0 | 40.9 |
| 2/22/2010 | 68.2 | 56.8 | 31.0 | 28.9 | 33.9 |
| 2/23/2010 | 66.5 | 52.7 | 31.0 | 28.0 | 30.1 |
| 2/24/2010 | 72.1 | 61.7 | 35.8 | 30.3 | 34.8 |
| 2/25/2010 | 75.0 | 60.0 | 35.0 | 30.3 | 29.9 |
| 2/26/2010 | 66.4 | 54.8 | 29.4 | 26.2 | 28.6 |
| 2/27/2010 | 75.9 | 64.7 | 47.7 | 36.2 | 31.7 |
| 2/28/2010 | 73.0 | 60.4 | 34.1 | 27.9 | 29.6 |
| 3/1/2010 | 68.2 | 54.4 | 30.0 | 27.1 | 28.8 |
| 3/2/2010 | 70.4 | 61.0 | 43.3 | 38.9 | 45.2 |
| 3/3/2010 | 62.8 | 56.3 | 35.5 | 29.2 | 28.7 |
| 3/4/2010 | 74.1 | 54.5 | 33.1 | 29.1 | 30.1 |
| 3/5/2010 | 60.8 | 57.8 | 40.1 | 34.7 | 31.2 |
| 3/6/2010 | 65.6 | 54.1 | 36.3 | 26.6 | 28.5 |
| 3/7/2010 | 65.4 | 56.2 | 42.8 | 37.6 | 34.9 |
| 3/8/2010 | 65.5 | 56.9 | 32.4 | 32.3 | 30.4 |
| 3/9/2010 | 60.0 | 52.9 | 31.6 | 26.4 | 28.3 |
| 3/10/2010 | 69.3 | 55.6 | 33.3 | 29.2 | 31.3 |
| 3/11/2010 | 70.9 | 61.3 | 37.1 | 30.8 | 36.8 |
| 3/12/2010 | 65.5 | 55.8 | 36.3 | 30.1 | 28.6 |
| 3/13/2010 | 69.9 | 61.3 | 41.2 | 35.4 | 29.8 |
| 3/14/2010 | 71.0 | 64.3 | 34.9 | 29.8 | 29.6 |
| 3/15/2010 | 71.2 | 61.8 | 35.7 | 32.4 | 29.7 |
| 3/16/2010 | 72.2 | 63.4 | 35.7 | 31.2 | 31.4 |
| 3/17/2010 | 70.6 | 60.7 | 29.9 | 27.0 | 28.3 |
| 3/18/2010 | 70.4 | 59.7 | 37.8 | 33.4 | 32.6 |
| 3/19/2010 | 80.2 | 69.2 | 54.3 | 51.0 | 47.8 |
| 3/20/2010 | 62.3 | 52.4 | 25.9 | 27.7 | 28.2 |
| 3/21/2010 | 66.7 | 57.7 | 27.1 | 26.8 | 28.4 |
| 3/22/2010 | 66.9 | 57.2 | 26.8 | 31.2 | 28.3 |
| 3/23/2010 | 70.7 | 59.9 | 30.2 | 27.3 | 29.1 |
| 3/24/2010 | 69.4 | 59.1 | 38.7 | 36.5 | 33.5 |
| 3/25/2010 | 70.7 | 62.5 | 31.4 | 32.2 | 32.6 |
| 3/26/2010 | 72.9 | 62.3 | 28.8 | 28.2 | 28.9 |
| 3/27/2010 | 72.8 | 64.1 | 41.6 | 33.4 | 29.4 |
| 3/28/2010 | 71.3 | 61.7 | 49.4 | 41.2 | 33.3 |
| 3/29/2010 | 74.4 | 63.8 | 39.3 | 35.5 | 31.6 |
| 3/30/2010 | 73.0 | 63.5 | 34.4 | 33.1 | 32.2 |
| 3/31/2010 | 71.5 | 61.1 | 42.9 | 34.5 | 31.7 |
| 4/1/2010 | 71.7 | 62.0 | 29.3 | 26.4 | 29.5 |
| 4/2/2010 | 80.2 | 69.3 | 47.4 | 35.8 | 31.3 |
| 4/3/2010 | 71.1 | 61.2 | 38.8 | 36.9 | 29.2 |
| 4/4/2010 | 73.0 | 64.7 | 35.4 | 36.6 | 36.1 |
| 4/5/2010 | 71.3 | 62.7 | 30.7 | 29.1 | 30.9 |
| 4/6/2010 | 71.2 | 61.9 | 41.1 | 34.5 | 31.7 |
| 4/7/2010 | 70.8 | 62.7 | 32.5 | 28.9 | 31.0 |
| 4/8/2010 | 74.5 | 64.0 | 29.7 | 28.0 | 30.2 |
| 4/9/2010 | 71.6 | 63.1 | 54.4 | 45.8 | 35.2 |
| 4/10/2010 | 72.1 | 60.6 | 40.5 | 36.2 | 33.9 |
| 4/11/2010 | 73.5 | 66.0 | 55.2 | 51.2 | 47.6 |
| 4/12/2010 | 72.8 | 64.1 | 33.4 | 30.1 | 30.7 |
| 4/13/2010 | 73.1 | 65.4 | 46.4 | 43.6 | 41.0 |
| 4/14/2010 | 73.9 | 64.6 | 37.9 | 35.2 | 33.3 |
| 4/15/2010 | 72.1 | 61.5 | 33.2 | 30.7 | 32.9 |
| 4/16/2010 | 73.0 | 67.5 | 35.3 | 32.6 | 32.0 |
| 4/17/2010 | 72.7 | 64.3 | 37.4 | 34.7 | 34.2 |
| 4/18/2010 | 74.1 | 64.4 | 34.9 | 32.6 | 31.9 |
| 4/19/2010 | 73.4 | 62.3 | 32.5 | 27.6 | 29.9 |
| 4/20/2010 | 74.3 | 63.3 | 30.5 | 30.3 | 30.2 |
| 4/21/2010 | 78.4 | 72.6 | 36.8 | 33.3 | 33.8 |
| 4/22/2010 | 74.1 | 68.1 | 38.0 | 33.7 | 32.1 |
| 4/23/2010 | 73.6 | 61.2 | 28.9 | 27.9 | 29.4 |
| 4/24/2010 | 74.5 | 61.9 | 28.8 | 28.8 | 30.0 |
| 4/25/2010 | 75.5 | 64.8 | 39.1 | 38.5 | 37.5 |
| 4/26/2010 | 75.3 | 65.1 | 32.9 | 31.5 | 33.2 |
| 4/27/2010 | 74.1 | 65.3 | 38.4 | 33.3 | 32.2 |
| 4/28/2010 | 75.4 | 68.4 | 46.0 | 41.4 | 38.0 |
| 4/29/2010 | 73.6 | 64.8 | 41.0 | 38.0 | 35.6 |
| 4/30/2010 | 74.5 | 66.1 | 31.6 | 29.4 | 31.2 |
| 5/1/2010 | 74.7 | 63.2 | 32.1 | 32.0 | 33.2 |
| 5/2/2010 | 73.1 | 62.1 | 30.2 | 29.0 | 30.3 |
| 5/3/2010 | 74.4 | 63.3 | 41.8 | 40.4 | 38.3 |
| 5/4/2010 | 73.2 | 62.7 | 47.8 | 46.3 | 44.4 |
| 5/5/2010 | 72.2 | 64.4 | 42.6 | 41.4 | 39.9 |
| 5/6/2010 | 76.3 | 63.6 | 39.5 | 38.4 | 36.5 |
| 5/7/2010 | 74.3 | 63.3 | 40.1 | 37.1 | 35.4 |
| 5/8/2010 | 75.6 | 63.8 | 39.3 | 37.9 | 35.4 |
| 5/9/2010 | 76.0 | 67.4 | 39.8 | 37.3 | 35.3 |
| 5/10/2010 | 77.5 | 66.3 | 41.5 | 40.1 | 39.3 |
| 5/11/2010 | 78.3 | 70.2 | 57.0 | 52.5 | 48.5 |
| 5/12/2010 | 78.2 | 71.9 | 57.2 | 53.1 | 48.2 |
| 5/13/2010 | 74.1 | 65.2 | 47.7 | 41.5 | 36.3 |
| 5/14/2010 | 73.1 | 64.3 | 48.5 | 43.6 | 39.7 |
| 5/15/2010 | 73.9 | 63.5 | 47.1 | 42.8 | 39.4 |
| 5/16/2010 | 70.1 | 62.3 | 44.9 | 40.0 | 36.6 |
| 5/17/2010 | 73.8 | 67.2 | 50.3 | 45.5 | 41.4 |
| 5/18/2010 | 71.7 | 66.2 | 50.7 | 44.8 | 39.8 |
| 5/19/2010 | 67.2 | 60.7 | 44.8 | 38.9 | 34.5 |
| 5/20/2010 | 73.0 | 71.9 | 47.7 | 41.9 | 37.0 |
| 5/21/2010 | 70.8 | 65.2 | 49.8 | 43.5 | 37.8 |
| 5/22/2010 | 65.6 | 59.3 | 44.5 | 39.0 | 35.5 |
| 5/23/2010 | 63.8 | 57.9 | 42.6 | 37.6 | 34.6 |
| 5/24/2010 | 58.3 | 50.8 | 34.9 | 30.8 | 30.0 |
| 5/25/2010 | 61.5 | 56.6 | 38.1 | 32.7 | 31.2 |
| 5/26/2010 | 69.8 | 63.9 | 49.5 | 45.2 | 40.2 |
| 5/27/2010 | 72.2 | 66.7 | 51.1 | 44.6 | 38.3 |
| 5/28/2010 | 65.6 | 60.8 | 45.2 | 39.9 | 36.3 |
| 5/29/2010 | 60.2 | 55.4 | 39.6 | 35.0 | 32.7 |
| 5/30/2010 | 64.0 | 59.0 | 43.5 | 37.5 | 33.3 |
| 5/31/2010 | 67.8 | 62.9 | 47.6 | 41.5 | 36.1 |
| 6/1/2010 | 72.4 | 67.5 | 51.9 | 45.5 | 39.8 |
| 6/2/2010 | 62.0 | 57.9 | 42.6 | 37.4 | 34.2 |
| 6/3/2010 | 62.1 | 57.5 | 43.2 | 40.1 | 37.6 |
| 6/4/2010 | 69.9 | 63.9 | 48.6 | 44.0 | 39.3 |
| 6/5/2010 | 54.7 | 49.3 | 34.4 | 28.8 | 28.5 |
| 6/6/2010 | 50.9 | 44.4 | 30.8 | 27.1 | 27.8 |
| 6/7/2010 | 63.1 | 58.4 | 43.5 | 38.2 | 33.3 |
| 6/8/2010 | 66.5 | 61.3 | 46.3 | 39.9 | 34.9 |
| 6/9/2010 | 65.8 | 50.9 | 34.5 | 25.2 | 28.0 |
| 6/10/2010 | 63.0 | 57.7 | 42.6 | 36.9 | 32.3 |
| 6/11/2010 | 63.1 | 57.8 | 42.9 | 37.8 | 33.2 |
| 6/12/2010 | 61.0 | 56.4 | 41.7 | 37.1 | 32.5 |
| 6/13/2010 | 72.8 | 67.5 | 52.9 | 46.6 | 38.8 |
| 6/14/2010 | 68.8 | 63.2 | 48.0 | 42.5 | 36.8 |
| 6/15/2010 | 63.6 | 59.0 | 43.8 | 42.3 | 35.5 |
| 6/16/2010 | 66.2 | 61.1 | 46.1 | 43.0 | 37.0 |
| 6/17/2010 | 69.7 | 64.3 | 49.0 | 44.5 | 38.0 |
| 6/18/2010 | 65.5 | 60.6 | 46.4 | 45.7 | 38.9 |
| 6/19/2010 | 66.1 | 60.8 | 46.1 | 41.4 | 35.6 |
| 6/20/2010 | 69.3 | 64.1 | 48.8 | 43.3 | 37.3 |
| 6/21/2010 | 74.0 | 68.4 | 52.7 | 45.4 | 38.1 |
| 6/22/2010 | 68.6 | 63.3 | 48.4 | 42.0 | 36.3 |
| 6/23/2010 | 62.4 | 57.2 | 42.0 | 35.8 | 31.8 |
| 6/24/2010 | 54.2 | 48.1 | 29.6 | 25.9 | 28.4 |
| 6/25/2010 | 66.9 | 61.4 | 46.6 | 40.2 | 34.8 |
| 6/26/2010 | 71.3 | 65.8 | 50.6 | 44.0 | 37.4 |
| 6/27/2010 | 68.1 | 62.7 | 47.8 | 41.5 | 35.8 |
| 6/28/2010 | 70.7 | 65.0 | 50.1 | 44.0 | 37.8 |
| 6/29/2010 | 64.1 | 58.9 | 43.7 | 38.0 | 32.6 |
| 6/30/2010 | 70.0 | 55.4 | 38.3 | 36.0 | 35.7 |
| 7/1/2010 | 66.8 | 61.6 | 46.8 | 42.6 | 36.8 |
| 7/2/2010 | 63.5 | 55.7 | 42.2 | 46.8 | 38.9 |
| 7/3/2010 | 60.9 | 56.2 | 41.7 | 35.7 | 32.0 |
| 7/4/2010 | 65.6 | 60.0 | 45.5 | 52.6 | 51.6 |
| 7/5/2010 | 64.8 | 59.6 | 46.3 | 45.7 | 37.7 |
| 7/6/2010 | 68.2 | 62.5 | 47.5 | 41.2 | 35.8 |
| 7/7/2010 | 64.6 | 59.1 | 44.6 | 38.2 | 33.4 |
| 7/8/2010 | 64.8 | 59.0 | 44.9 | 40.5 | 35.0 |
| 7/9/2010 | 69.0 | 63.5 | 48.4 | 42.0 | 36.2 |
| 7/10/2010 | 68.2 | 62.7 | 47.8 | 41.3 | 35.9 |
| 7/11/2010 | 54.3 | 50.0 | 35.6 | 30.4 | 29.4 |
| 7/12/2010 | 58.8 | 54.0 | 39.9 | 33.9 | 31.0 |
| 7/13/2010 | 66.1 | 60.7 | 46.1 | 39.6 | 34.4 |
| 7/14/2010 | 66.8 | 61.3 | 46.6 | 40.3 | 35.1 |
| 7/15/2010 | 55.4 | 51.2 | 37.1 | 31.0 | 29.7 |
| 7/16/2010 | 60.9 | 55.8 | 41.3 | 35.4 | 31.7 |
| 7/17/2010 | 65.7 | 60.4 | 45.7 | 39.5 | 34.6 |
| 7/18/2010 | 59.7 | 54.6 | 40.0 | 34.0 | 30.9 |
| 7/19/2010 | 63.9 | 58.8 | 44.5 | 41.6 | 35.9 |
| 7/20/2010 | 56.3 | 53.9 | 44.7 | 44.9 | 37.1 |
| 7/21/2010 | 55.0 | 51.2 | 36.2 | 31.2 | 30.0 |
| 7/22/2010 | 70.1 | 64.4 | 49.4 | 42.2 | 35.6 |
| 7/23/2010 | 67.7 | 62.2 | 47.2 | 40.8 | 34.8 |
| 7/24/2010 | 62.4 | 56.9 | 42.6 | 36.6 | 32.3 |
| 7/25/2010 | 64.6 | 59.3 | 44.8 | 38.6 | 34.1 |
| 7/26/2010 | 60.6 | 55.4 | 41.1 | 37.5 | 32.1 |
| 7/27/2010 | 67.0 | 61.5 | 46.9 | 40.9 | 35.6 |
| 7/28/2010 | 65.4 | 60.0 | 45.4 | 39.1 | 34.2 |
| 7/29/2010 | 68.1 | 62.5 | 47.7 | 41.8 | 36.0 |
| 7/30/2010 | 67.0 | 61.6 | 47.0 | 40.7 | 35.4 |
| 7/31/2010 | 68.1 | 62.7 | 48.0 | 41.8 | 36.1 |
| 8/1/2010 | 57.2 | 52.5 | 38.2 | 32.4 | 29.6 |
| 8/2/2010 | 67.3 | 61.9 | 47.2 | 40.9 | 35.5 |
| 8/3/2010 | 68.8 | 63.3 | 48.3 | 42.0 | 36.2 |
| 8/4/2010 | 68.7 | 63.2 | 48.4 | 42.1 | 36.4 |
| 8/5/2010 | 69.7 | 64.0 | 49.1 | 42.8 | 36.7 |
| 8/6/2010 | 69.2 | 63.5 | 48.8 | 42.7 | 36.7 |
| 8/7/2010 | 64.6 | 59.3 | 44.8 | 39.3 | 34.0 |
| 8/8/2010 | 72.2 | 66.7 | 51.2 | 44.2 | 37.0 |
| 8/9/2010 | 59.5 | 54.3 | 41.3 | 43.6 | 35.7 |
| 8/10/2010 | 60.4 | 55.2 | 41.4 | 35.2 | 31.3 |
| 8/11/2010 | 55.7 | 49.6 | 39.5 | 32.1 | 29.7 |
| 8/12/2010 | 66.0 | 60.3 | 47.0 | 40.0 | 34.8 |
| 8/13/2010 | 68.6 | 61.2 | 46.6 | 39.5 | 34.4 |
| 8/14/2010 | 73.3 | 67.6 | 51.8 | 41.7 | 34.5 |
| 8/15/2010 | 73.7 | 67.9 | 52.1 | 44.3 | 36.7 |
| 8/16/2010 | 70.1 | 64.6 | 49.2 | 42.3 | 35.7 |
| 8/17/2010 | 70.1 | 64.6 | 49.7 | 43.1 | 37.1 |
| 8/18/2010 | 65.4 | 60.0 | 45.6 | 42.6 | 36.2 |
| 8/19/2010 | 61.4 | 55.4 | 41.4 | 34.7 | 31.3 |
| 8/20/2010 | 72.1 | 66.6 | 52.0 | 46.4 | 39.6 |
| 8/21/2010 | 70.2 | 64.0 | 49.9 | 43.6 | 37.6 |
| 8/22/2010 | 66.5 | 61.0 | 46.4 | 40.1 | 34.9 |
| 8/23/2010 | 58.9 | 54.0 | 40.4 | 34.1 | 31.2 |
| 8/24/2010 | 70.8 | 65.2 | 50.1 | 43.5 | 37.4 |
| 8/25/2010 | 71.2 | 65.4 | 50.5 | 44.2 | 37.8 |
| 8/26/2010 | 71.2 | 65.6 | 50.8 | 44.5 | 37.9 |
| 8/27/2010 | 67.4 | 61.8 | 47.0 | 41.3 | 35.7 |
| 8/28/2010 | 69.3 | 62.2 | 47.3 | 41.0 | 35.6 |
| 8/29/2010 | 72.8 | 61.3 | 39.3 | 33.0 | 30.5 |
| 8/30/2010 | 65.1 | 59.9 | 45.5 | 39.1 | 34.4 |
| 8/31/2010 | 63.0 | 57.2 | 43.1 | 36.9 | 32.9 |
| 9/1/2010 | 69.3 | 63.7 | 48.7 | 42.3 | 36.6 |
| 9/2/2010 | 73.2 | 66.7 | 51.5 | 45.3 | 38.5 |
| 9/3/2010 | 71.5 | 65.8 | 50.9 | 44.8 | 38.3 |
| 9/4/2010 | 67.9 | 62.7 | 48.6 | 44.6 | 38.2 |
| 9/5/2010 | 68.5 | 62.9 | 48.2 | 41.9 | 36.3 |
| 9/6/2010 | 74.3 | 68.4 | 52.6 | 45.3 | 38.0 |
| 9/7/2010 | 74.2 | 68.3 | 53.6 | 45.5 | 38.6 |
| 9/8/2010 | 72.4 | 66.9 | 51.6 | 44.9 | 38.4 |
| 9/9/2010 | 66.7 | 61.1 | 46.8 | 40.3 | 35.3 |
| 9/10/2010 | 68.4 | 62.9 | 48.9 | 47.8 | 40.9 |
| 9/11/2010 | 64.3 | 58.7 | 44.7 | 38.3 | 34.2 |
| 9/12/2010 | 75.1 | 69.5 | 53.3 | 45.5 | 37.9 |
| 9/13/2010 | 74.7 | 68.9 | 53.0 | 47.2 | 39.5 |
| 9/14/2010 | 55.4 | 50.4 | 39.0 | 30.9 | 30.0 |
| 9/15/2010 | 56.4 | 52.3 | 38.9 | 32.7 | 30.8 |
| 9/16/2010 | 67.2 | 62.2 | 47.4 | 41.2 | 36.0 |
| 9/17/2010 | 67.3 | 62.3 | 47.6 | 41.6 | 36.5 |
| 9/18/2010 | 69.7 | 64.5 | 49.6 | 44.2 | 38.2 |
| 9/19/2010 | 72.9 | 67.3 | 51.9 | 45.4 | 38.2 |
| 9/20/2010 | 72.2 | 66.4 | 51.1 | 45.3 | 39.3 |
| 9/21/2010 | 72.6 | 67.0 | 51.5 | 44.9 | 38.1 |
| 9/22/2010 | 68.8 | 63.2 | 48.3 | 42.2 | 36.4 |
| 9/23/2010 | 66.4 | 61.0 | 47.1 | 44.4 | 37.8 |
| 9/24/2010 | 69.1 | 63.8 | 48.9 | 42.9 | 37.2 |
| 9/25/2010 | 73.5 | 67.7 | 52.0 | 45.3 | 38.2 |
| 9/26/2010 | 74.7 | 62.1 | 42.0 | 24.7 | 29.8 |
| 9/27/2010 | 88.5 | 72.0 | 52.6 | 61.7 | 40.7 |
| 9/28/2010 | 76.4 | 71.1 | 52.9 | 44.2 | 37.2 |
| 9/29/2010 | 74.4 | 68.8 | 52.4 | 45.2 | 38.7 |
| 9/30/2010 | 72.5 | 67.1 | 51.0 | 44.2 | 38.0 |
| 10/1/2010 | 70.9 | 66.0 | 50.0 | 43.5 | 37.8 |
| 10/2/2010 | 75.7 | 70.5 | 53.0 | 45.0 | 37.8 |
| 10/3/2010 | 72.4 | 67.0 | 50.6 | 44.2 | 38.3 |
| 10/4/2010 | 68.9 | 63.8 | 48.0 | 42.3 | 37.4 |
| 10/5/2010 | 67.6 | 62.4 | 46.8 | 41.1 | 35.5 |
| 10/6/2010 | 68.3 | 63.1 | 47.5 | 42.2 | 36.9 |
| 10/7/2010 | 68.6 | 63.3 | 47.7 | 42.0 | 36.7 |
| 10/8/2010 | 63.1 | 58.1 | 42.9 | 39.4 | 34.6 |
| 10/9/2010 | 67.5 | 62.4 | 47.0 | 41.5 | 36.2 |
| 10/10/2010 | 72.0 | 66.6 | 50.2 | 43.8 | 38.1 |
| 10/11/2010 | 73.2 | 68.1 | 51.3 | 44.6 | 38.3 |
| 10/12/2010 | 68.5 | 63.4 | 47.9 | 42.8 | 37.2 |
| 10/13/2010 | 65.7 | 60.7 | 45.9 | 43.9 | 39.1 |
| 10/14/2010 | 66.5 | 61.2 | 45.9 | 43.1 | 37.5 |
| 10/15/2010 | 67.6 | 62.6 | 46.7 | 41.7 | 36.7 |
| 10/16/2010 | 74.0 | 68.6 | 51.8 | 45.0 | 38.2 |
| 10/17/2010 | 75.6 | 70.1 | 52.5 | 44.6 | 38.1 |
| 10/18/2010 | 74.6 | 69.1 | 52.2 | 44.7 | 38.6 |
| 10/19/2010 | 69.9 | 64.5 | 48.5 | 42.1 | 36.8 |
| 10/20/2010 | 69.5 | 63.6 | 48.2 | 42.3 | 37.3 |
| 10/21/2010 | 75.0 | 69.8 | 51.9 | 44.0 | 37.4 |
| 10/22/2010 | 73.4 | 68.0 | 50.2 | 43.4 | 37.8 |
| 10/23/2010 | 69.8 | 64.8 | 49.2 | 43.1 | 37.1 |
| 10/24/2010 | 68.8 | 63.7 | 48.7 | 43.5 | 37.8 |
| 10/25/2010 | 71.8 | 66.6 | 50.9 | 44.2 | 38.2 |
| 10/26/2010 | 69.1 | 64.0 | 48.6 | 41.9 | 36.4 |
| 10/27/2010 | 76.1 | 71.1 | 54.4 | 50.4 | 48.0 |
| 10/28/2010 | 75.4 | 71.8 | 55.0 | 50.3 | 48.1 |
| 10/29/2010 | 73.2 | 67.8 | 51.3 | 44.6 | 39.2 |
| 10/30/2010 | 69.0 | 63.5 | 47.3 | 40.8 | 37.5 |
| 10/31/2010 | 73.2 | 68.1 | 50.6 | 43.3 | 38.2 |
| 11/1/2010 | 73.0 | 67.7 | 51.0 | 44.0 | 39.0 |
| 11/2/2010 | 75.7 | 70.4 | 52.2 | 44.3 | 38.8 |
| 11/3/2010 | 72.1 | 67.1 | 50.7 | 45.2 | 39.9 |
| 11/4/2010 | 69.9 | 64.6 | 49.0 | 42.8 | 38.0 |
| 11/5/2010 | 72.8 | 67.6 | 51.2 | 44.5 | 38.9 |
| 11/6/2010 | 74.0 | 68.6 | 51.6 | 44.6 | 38.9 |
| 11/7/2010 | 69.4 | 64.5 | 48.6 | 42.4 | 37.2 |
| 11/8/2010 | 69.9 | 65.0 | 49.0 | 43.4 | 37.8 |
| 11/9/2010 | 73.9 | 68.6 | 51.7 | 44.6 | 38.4 |
| 11/10/2010 | 75.0 | 69.5 | 52.3 | 45.2 | 38.6 |
| 11/11/2010 | 74.9 | 69.7 | 52.0 | 44.6 | 37.7 |
| 11/12/2010 | 76.0 | 70.4 | 52.2 | 44.4 | 37.6 |
| 11/13/2010 | 74.9 | 69.3 | 51.8 | 44.6 | 38.6 |
| 11/14/2010 | 69.2 | 63.7 | 47.9 | 42.1 | 37.0 |
| 11/15/2010 | 68.4 | 58.3 | 42.8 | 37.4 | 33.2 |
| 11/16/2010 | 71.1 | 65.2 | 48.9 | 42.6 | 37.1 |
| 11/17/2010 | 74.9 | 69.3 | 52.2 | 44.9 | 38.7 |
| 11/18/2010 | 73.2 | 67.4 | 51.4 | 44.6 | 38.9 |
| 11/19/2010 | 74.0 | 68.2 | 52.0 | 45.4 | 39.1 |
| 11/20/2010 | 74.6 | 67.6 | 52.0 | 45.1 | 38.7 |
| 11/21/2010 | 71.2 | 65.3 | 50.7 | 48.5 | 41.6 |
| 11/22/2010 | 69.0 | 63.1 | 48.3 | 45.1 | 38.8 |
| 11/23/2010 | 75.5 | 68.9 | 51.8 | 43.8 | 37.5 |
| 11/24/2010 | 74.4 | 68.3 | 50.4 | 42.6 | 37.0 |
| 11/25/2010 | 70.8 | 65.4 | 49.0 | 43.0 | 38.2 |
| 11/26/2010 | 70.5 | 65.1 | 49.5 | 44.6 | 39.1 |
| 11/27/2010 | 72.8 | 67.0 | 51.4 | 54.0 | 45.4 |
| 11/28/2010 | 63.0 | 57.7 | 43.0 | 43.1 | 34.6 |
| 11/29/2010 | 74.7 | 69.1 | 51.8 | 44.2 | 37.9 |
| 11/30/2010 | 73.2 | 67.4 | 50.9 | 45.7 | 38.5 |
| 12/1/2010 | 68.1 | 62.7 | 47.6 | 44.8 | 37.5 |
| 12/2/2010 | 74.2 | 69.1 | 53.1 | 54.8 | 46.7 |
| 12/3/2010 | 72.8 | 67.0 | 50.3 | 43.9 | 38.9 |
| 12/4/2010 | 78.3 | 72.8 | 51.1 | 41.6 | 35.5 |
| 12/5/2010 | 76.0 | 70.3 | 51.7 | 43.7 | 37.7 |
| 12/6/2010 | 65.6 | 60.0 | 44.7 | 38.4 | 34.8 |
| 12/7/2010 | 69.4 | 63.9 | 48.1 | 42.4 | 37.5 |
| 12/8/2010 | 74.0 | 68.5 | 51.6 | 44.5 | 38.7 |
| 12/9/2010 | 70.9 | 65.3 | 49.6 | 43.3 | 38.5 |
| 12/10/2010 | 67.0 | 61.6 | 46.8 | 40.4 | 36.4 |
| 12/11/2010 | 68.0 | 62.8 | 47.5 | 41.1 | 37.0 |
| 12/12/2010 | 63.1 | 57.6 | 42.8 | 37.7 | 34.2 |
| 12/13/2010 | 71.3 | 65.7 | 50.1 | 43.6 | 38.3 |
| 12/14/2010 | 67.1 | 62.0 | 47.0 | 40.7 | 36.9 |
| 12/15/2010 | 69.2 | 63.7 | 48.2 | 41.7 | 37.5 |
| 12/16/2010 | 65.4 | 59.9 | 45.2 | 38.9 | 35.3 |
| 12/17/2010 | 66.6 | 61.1 | 46.2 | 40.6 | 36.0 |
| 12/18/2010 | 72.0 | 66.4 | 50.4 | 43.7 | 38.3 |
| 12/19/2010 | 74.1 | 68.6 | 51.6 | 44.7 | 38.6 |
| 12/20/2010 | 71.5 | 66.0 | 49.9 | 43.2 | 38.7 |
| 12/21/2010 | 74.6 | 69.0 | 51.7 | 44.6 | 38.6 |
| 12/22/2010 | 70.2 | 64.4 | 48.3 | 41.4 | 36.5 |
| 12/23/2010 | 72.9 | 67.4 | 51.1 | 44.6 | 39.0 |
| 12/24/2010 | 75.6 | 69.8 | 51.6 | 43.8 | 37.7 |
| 12/25/2010 | 76.7 | 71.1 | 51.8 | 43.3 | 36.6 |
| 12/26/2010 | 77.8 | 71.9 | 50.9 | 41.4 | 35.8 |
| 12/27/2010 | 76.3 | 70.4 | 51.6 | 43.6 | 37.3 |
| 12/28/2010 | 73.9 | 68.4 | 51.4 | 44.3 | 39.0 |
| 12/29/2010 | 76.4 | 70.7 | 52.5 | 44.3 | 38.0 |
| 12/30/2010 | 77.4 | 71.5 | 53.2 | 44.5 | 37.4 |
| 12/31/2010 | 70.2 | 64.0 | 48.1 | 41.5 | 37.2 |
| 1/1/2011 | 77.0 | 71.0 | 51.3 | 42.9 | 36.9 |
| 1/2/2011 | 76.0 | 70.1 | 51.4 | 43.9 | 38.8 |
| 1/3/2011 | 72.0 | 65.8 | 49.0 | 42.0 | 37.6 |
| 1/4/2011 | 76.7 | 71.0 | 52.2 | 44.3 | 39.3 |
| 1/5/2011 | 68.5 | 62.3 | 47.5 | 42.2 | 39.6 |
| 1/6/2011 | 73.0 | 67.1 | 52.1 | 47.9 | 45.1 |
| 1/7/2011 | 77.5 | 71.5 | 52.4 | 43.9 | 37.5 |
| 1/8/2011 | 77.2 | 71.1 | 54.7 | 51.2 | 47.2 |
| 1/9/2011 | 76.3 | 69.9 | 52.7 | 44.9 | 38.3 |
| 1/10/2011 | 74.9 | 68.8 | 52.2 | 45.0 | 38.7 |
| 1/11/2011 | 74.3 | 68.5 | 51.7 | 44.8 | 38.9 |
| 1/12/2011 | 73.0 | 67.2 | 50.6 | 43.7 | 38.7 |
| 1/13/2011 | 75.2 | 69.0 | 51.4 | 44.6 | 39.0 |
| 1/14/2011 | 80.5 | 72.4 | 56.6 | 51.3 | 48.0 |
| 1/15/2011 | 78.0 | 69.7 | 52.6 | 46.6 | 42.5 |
| 1/16/2011 | 75.3 | 67.3 | 51.7 | 46.3 | 44.7 |
| 1/17/2011 | 71.7 | 62.2 | 47.5 | 42.6 | 41.2 |
| 1/18/2011 | 78.0 | 70.5 | 53.9 | 48.3 | 46.4 |
| 1/19/2011 | 80.0 | 72.7 | 54.6 | 48.5 | 46.1 |
| 1/20/2011 | 80.9 | 72.2 | 53.2 | 47.7 | 44.5 |
| 1/21/2011 | 73.3 | 62.9 | 45.5 | 40.5 | 38.1 |
| 1/22/2011 | 70.3 | 59.8 | 44.1 | 37.7 | 34.8 |
| 1/23/2011 | 78.2 | 67.2 | 51.0 | 45.3 | 44.8 |
| 1/24/2011 | 67.9 | 60.6 | 43.5 | 38.4 | 34.5 |
| 1/25/2011 | 61.6 | 49.4 | 46.5 | 42.4 | 36.5 |
| 1/26/2011 | 65.4 | 48.1 | 32.5 | 34.5 | 32.5 |
| 1/27/2011 | 73.3 | 65.6 | 43.3 | 34.3 | 33.7 |
| 1/28/2011 | 72.7 | 64.1 | 51.1 | 48.3 | 46.2 |
| 1/29/2011 | 72.9 | 57.8 | 45.4 | 42.5 | 40.5 |
| 1/30/2011 | 80.3 | 63.0 | 47.0 | 43.3 | 41.1 |
| 1/31/2011 | 76.9 | 57.6 | 43.6 | 43.2 | 44.1 |
| 2/1/2011 | 73.5 | 60.6 | 47.0 | 48.0 | 46.5 |
| 2/2/2011 | 72.4 | 58.0 | 46.5 | 45.2 | 42.7 |
| 2/3/2011 | 71.6 | 61.7 | 52.8 | 50.7 | 47.9 |
| 2/4/2011 | 75.0 | 64.2 | 47.0 | 44.2 | 41.4 |
| 2/5/2011 | 72.2 | 61.7 | 49.9 | 46.6 | 43.8 |
| 2/6/2011 | 71.1 | 57.9 | 41.5 | 39.0 | 38.1 |
| 2/7/2011 | 69.1 | 52.4 | 31.3 | 30.0 | 34.0 |
| 2/8/2011 | 66.0 | 48.5 | 32.6 | 29.5 | 33.1 |
| 2/9/2011 | 64.7 | 52.2 | 40.3 | 40.4 | 46.7 |
| 2/10/2011 | 70.7 | 56.7 | 39.5 | 31.1 | 33.5 |
| 2/11/2011 | 72.8 | 58.0 | 41.3 | 38.7 | 38.7 |
| 2/12/2011 | 68.1 | 58.6 | 44.6 | 41.6 | 40.9 |
| 2/13/2011 | 70.7 | 61.8 | 44.2 | 41.6 | 39.8 |
| 2/14/2011 | 67.9 | 57.4 | 36.3 | 33.1 | 34.3 |
| 2/15/2011 | 67.5 | 57.1 | 42.5 | 41.3 | 39.1 |
| 2/16/2011 | 73.4 | 67.0 | 57.0 | 54.7 | 51.8 |
| 2/17/2011 | 75.8 | 68.6 | 64.8 | 64.2 | 62.4 |
| 2/18/2011 | 76.8 | 68.4 | 60.8 | 57.9 | 54.9 |
| 2/19/2011 | 80.0 | 70.5 | 51.6 | 48.8 | 48.4 |
| 2/20/2011 | 79.2 | 71.2 | 70.3 | 68.2 | 65.9 |
| 2/21/2011 | 79.9 | 67.6 | 64.9 | 61.6 | 58.8 |
| 2/22/2011 | 76.3 | 68.1 | 59.5 | 54.2 | 51.8 |
| 2/23/2011 | 79.2 | 71.4 | 59.6 | 53.7 | 50.3 |
| 2/24/2011 | 78.4 | 72.9 | 60.4 | 57.6 | 57.3 |
| 2/25/2011 | 75.6 | 69.9 | 52.7 | 47.7 | 44.8 |
| 2/26/2011 | 75.2 | 57.2 | 43.0 | 43.3 | 45.2 |
| 2/27/2011 | 72.7 | 57.1 | 44.7 | 46.2 | 48.1 |
| 2/28/2011 | 74.4 | 58.7 | 48.7 | 49.0 | 49.5 |
| 3/1/2011 | 76.0 | 61.5 | 49.2 | 49.6 | 49.3 |
| 3/2/2011 | 75.0 | 61.6 | 42.4 | 44.9 | 46.6 |
| 3/3/2011 | 73.7 | 59.6 | 47.1 | 48.3 | 47.6 |
| 3/4/2011 | 72.9 | 61.0 | 43.0 | 43.5 | 43.9 |
| 3/5/2011 | 73.3 | 59.2 | 43.1 | 42.2 | 43.1 |
| 3/6/2011 | 76.9 | 60.4 | 44.6 | 43.2 | 43.9 |
| 3/7/2011 | 72.0 | 57.0 | 40.8 | 39.6 | 36.7 |
| 3/8/2011 | 71.8 | 55.2 | 37.1 | 37.5 | 38.7 |
| 3/9/2011 | 75.0 | 60.2 | 35.3 | 33.8 | 31.4 |
| 3/10/2011 | 74.6 | 61.3 | 42.1 | 39.8 | 40.0 |
| 3/11/2011 | 77.5 | 67.1 | 50.8 | 49.3 | 48.5 |
| 3/12/2011 | 77.4 | 63.6 | 45.6 | 43.5 | 42.5 |
| 3/13/2011 | 74.4 | 61.7 | 42.8 | 41.5 | 41.6 |
| 3/14/2011 | 72.2 | 63.5 | 41.8 | 40.9 | 40.2 |
| 3/15/2011 | 73.8 | 59.6 | 32.9 | 28.3 | 29.1 |
| 3/16/2011 | 74.1 | 60.7 | 33.1 | 33.8 | 31.4 |
| 3/17/2011 | 71.9 | 61.8 | 42.1 | 38.2 | 38.8 |
| 3/18/2011 | 74.4 | 66.0 | 53.1 | 49.6 | 48.3 |
| 3/19/2011 | 72.5 | 65.8 | 53.1 | 50.8 | 50.0 |
| 3/20/2011 | 68.9 | 61.7 | 47.2 | 43.4 | 41.7 |
| 3/21/2011 | 68.3 | 62.0 | 48.2 | 44.8 | 43.2 |
| 3/22/2011 | 68.2 | 59.0 | 42.6 | 40.7 | 39.1 |
| 3/23/2011 | 72.0 | 60.7 | 44.8 | 43.0 | 41.9 |
| 3/24/2011 | 67.6 | 59.6 | 42.7 | 39.4 | 38.6 |
| 3/25/2011 | 65.9 | 56.9 | 42.9 | 41.5 | 41.6 |
| 3/26/2011 | 66.8 | 58.0 | 37.5 | 34.9 | 36.7 |
| 3/27/2011 | 67.6 | 58.7 | 46.9 | 45.2 | 44.3 |
| 3/28/2011 | 68.3 | 62.2 | 42.5 | 42.7 | 44.0 |
| 3/29/2011 | 68.7 | 59.7 | 42.0 | 41.0 | 42.4 |
| 3/30/2011 | 66.6 | 56.2 | 37.7 | 36.2 | 38.0 |
| 3/31/2011 | 67.0 | 56.8 | 44.1 | 45.8 | 45.3 |
| 4/1/2011 | 67.4 | 56.4 | 36.7 | 37.6 | 39.5 |
| 4/2/2011 | 68.3 | 57.3 | 34.7 | 32.8 | 35.9 |
| 4/3/2011 | 69.0 | 60.6 | 46.6 | 42.3 | 39.8 |
| 4/4/2011 | 69.5 | 60.5 | 32.9 | 29.8 | 34.2 |
| 4/5/2011 | 68.0 | 57.5 | 44.6 | 41.9 | 40.2 |
| 4/6/2011 | 68.9 | 57.1 | 44.0 | 40.4 | 39.4 |
| 4/7/2011 | 70.6 | 62.8 | 49.6 | 47.3 | 46.9 |
| 4/8/2011 | 71.7 | 65.9 | 49.4 | 44.3 | 40.8 |
| 4/9/2011 | 69.8 | 60.3 | 43.7 | 39.0 | 37.0 |
| 4/10/2011 | 69.7 | 57.5 | 37.4 | 36.2 | 38.2 |
| 4/11/2011 | 71.6 | 60.5 | 42.1 | 39.8 | 39.4 |
| 4/12/2011 | 70.0 | 60.4 | 29.0 | 26.5 | 32.8 |
| 4/13/2011 | 72.2 | 62.2 | 33.6 | 34.6 | 35.1 |
| 4/14/2011 | 69.5 | 58.0 | 28.9 | 32.5 | 33.0 |
| 4/15/2011 | 71.8 | 59.6 | 31.9 | 31.0 | 37.4 |
| 4/16/2011 | 72.1 | 61.7 | 33.6 | 36.7 | 35.6 |
| 4/17/2011 | 70.1 | 59.2 | 31.9 | 32.4 | 34.9 |
| 4/18/2011 | 72.2 | 66.2 | 32.4 | 33.5 | 35.1 |
| 4/19/2011 | 72.3 | 64.7 | 31.8 | 31.2 | 34.6 |
| 4/20/2011 | 72.7 | 60.3 | 34.0 | 33.5 | 35.8 |
| 4/21/2011 | 70.1 | 55.6 | 31.2 | 30.9 | 34.3 |
| 4/22/2011 | 70.1 | 60.5 | 37.4 | 33.9 | 35.5 |
| 4/23/2011 | 72.1 | 62.6 | 45.4 | 40.6 | 38.1 |
| 4/24/2011 | 71.7 | 62.6 | 32.9 | 35.7 | 35.0 |
| 4/25/2011 | 73.5 | 63.0 | 31.6 | 30.1 | 34.4 |
| 4/26/2011 | 72.7 | 62.1 | 36.4 | 31.3 | 34.3 |
| 4/27/2011 | 72.7 | 62.6 | 35.6 | 33.6 | 36.6 |
| 4/28/2011 | 72.3 | 61.5 | 42.8 | 37.8 | 37.0 |
| 4/29/2011 | 72.4 | 61.8 | 39.8 | 37.4 | 36.5 |
| 4/30/2011 | 72.3 | 62.1 | 36.5 | 33.0 | 35.1 |
| 5/1/2011 | 73.1 | 61.0 | 30.6 | 31.1 | 33.8 |
| 5/2/2011 | 72.6 | 60.6 | 32.4 | 30.3 | 34.2 |
| 5/3/2011 | 72.8 | 61.9 | 43.8 | 41.4 | 40.0 |
| 5/4/2011 | 73.1 | 61.5 | 32.3 | 29.2 | 33.8 |
| 5/5/2011 | 74.5 | 64.1 | 32.9 | 31.0 | 35.2 |
| 5/6/2011 | 74.3 | 63.2 | 39.5 | 38.1 | 38.4 |
| 5/7/2011 | 74.2 | 63.2 | 32.7 | 32.7 | 34.9 |
| 5/8/2011 | 74.1 | 63.6 | 33.9 | 31.7 | 35.8 |
| 5/9/2011 | 73.2 | 63.0 | 32.0 | 34.6 | 34.8 |
| 5/10/2011 | 72.8 | 62.5 | 31.6 | 36.8 | 35.6 |
| 5/11/2011 | 73.6 | 64.5 | 46.8 | 45.0 | 43.8 |
| 5/12/2011 | 74.7 | 67.5 | 56.6 | 54.4 | 52.3 |
| 5/13/2011 | 74.0 | 67.2 | 55.9 | 54.3 | 52.0 |
| 5/14/2011 | 68.8 | 61.0 | 47.2 | 45.1 | 43.1 |
| 5/15/2011 | 68.9 | 63.7 | 47.3 | 44.7 | 43.0 |
| 5/16/2011 | 67.3 | 60.7 | 44.4 | 43.0 | 41.3 |
| 5/17/2011 | 64.0 | 55.0 | 37.1 | 35.3 | 33.5 |
| 5/18/2011 | 63.1 | 54.0 | 34.8 | 39.2 | 34.7 |
| 5/19/2011 | 67.5 | 61.3 | 44.9 | 35.0 | 32.7 |
| 5/20/2011 | 76.0 | 72.8 | 60.2 | 58.5 | 63.8 |
